# Reciprocal interaction between SIRT6 and APC/C regulates genomic stability

**DOI:** 10.1038/s41598-021-93684-w

**Published:** 2021-07-09

**Authors:** Helin Wang, Kangze Feng, Qingtao Wang, Haiteng Deng

**Affiliations:** 1 MOE Key Laboratory of Bioinformatics, Center for Synthetic and Systematic Biology, School of Life Sciences, Tsinghua University, Beijing, 100084 China; 2 Beijing Chaoyang Hospital Affiliated to Capital Medical University, Beijing, 100043 China

**Keywords:** Acetylation, Ubiquitin ligases

## Abstract

SIRT6 is an NAD^+^-dependent deacetylase that plays an important role in mitosis fidelity and genome stability. In the present study, we found that SIRT6 overexpression leads to mitosis defects and aneuploidy. We identified SIRT6 as a novel substrate of anaphase-promoting complex/cyclosome (APC/C), which is a master regulator of mitosis. Both CDH1 and CDC20, co-activators of APC/C, mediated SIRT6 degradation via the ubiquitination-proteasome pathway. Reciprocally, SIRT6 also deacetylated CDH1 at lysine K135 and promoted its degradation, resulting in an increase in APC/C-CDH1-targeted substrates, dysfunction in centrosome amplification, and chromosome instability. Our findings demonstrate the importance of SIRT6 for genome integrity during mitotic progression and reveal how SIRT6 and APC/C cooperate to drive mitosis.

## Introduction

The sirtuin family has seven members, which are NAD^+^-dependent protein deacetylases and/or mono-ADP-ribosyl transferases. While studies have found that SIRT6 plays important roles in aging, DNA repair, and metabolism^1^, it is unclear whether SIRT6 is an oncogene or a tumor suppressor. In liver, lung, pancreas, colon, and ovarian cancers, SIRT6 is downregulated in tumors, and/or the higher SIRT6 expression is associated with longer overall survival. In multiple myeloma, acute myeloid leukemia, skin cancer, papillary thyroid carcinoma, and head and neck squamous cell carcinoma, the opposite results have been reported. For some types of cancer, such as breast cancer, the results are conflicting^2^. Thus, the relationship between SIRT6 and cancer development remains to be elucidated.

A well-studied cause of cancer is aneuploidy induced by imbalanced mitosis^3^, and studies have indicated that SIRT6 is an important participant in mitosis. The earliest study reported that SIRT6 expression fluctuated during mitosis and that SIRT6 was co-localized with mitotic spindles, indicating that SIRT6 is regulated during this process and there are non-histone substrates of SIRT6^4^. Then, two groups reported histone H4K16ac and H3K18ac as mitotic substrates of SIRT6^5,6^. However, the regulatory mechanism of SIRT6 and its non-histone substrates during mitosis remains elusive.

As an E3 ubiquitin ligase that plays a pivotal role in cell cycle progression, the anaphase-promoting complex/cyclosome (APC/C) is a candidate SIRT6 regulator. APC/C is a large multi-subunit member of the RING-type ubiquitin ligases, which comprises 15 proteins and a total of 19 subunits, including scaffolding and catalytic components, and the two co-activators CDC20 and CDH1, which function as substrate adapters^7^. The APC/C initiates the separation of sister-chromatin and mitotic exit by targeting key regulators for proteasomal degradation, such as securin and cyclin B^8^. The proper destruction of these regulators is crucial for accurate chromosome segregation and successful progression through cell division. Disruption of APC/C regulation may cause serious mitotic deficiency and lead to genomic instability, which has been associated with the initiation of different types of cancer. Therefore, the activity of APC/C is strictly regulated by a number of mechanisms, such as degradation of its subunits, binding of inhibitor and activators, and post-transcriptional modifications, such as acetylation and phosphorylation^9,10^. Among all levels of regulation, the regulation of subunit levels is less clear than others, while our study sheds light on this process.

Our findings demonstrate that there is reciprocal regulation between SIRT6 and APC/C. SIRT6 is ubiquitinated by APC/C-CDC20/CDH1 and degraded. Intriguingly, SIRT6 also deacetylates CDH1 at K135, which promotes degradation of CDH1, thus reducing APC/C-CDH1 E3 ligase activity. Through this process, overexpression of SIRT6 leads to upregulation of a number of APC/C substrates, resulting in mitotic defects and genome instability.

## Results

### SIRT6 over-expression leads to impaired mitotic function and slows cell proliferation

**Figure 1:**
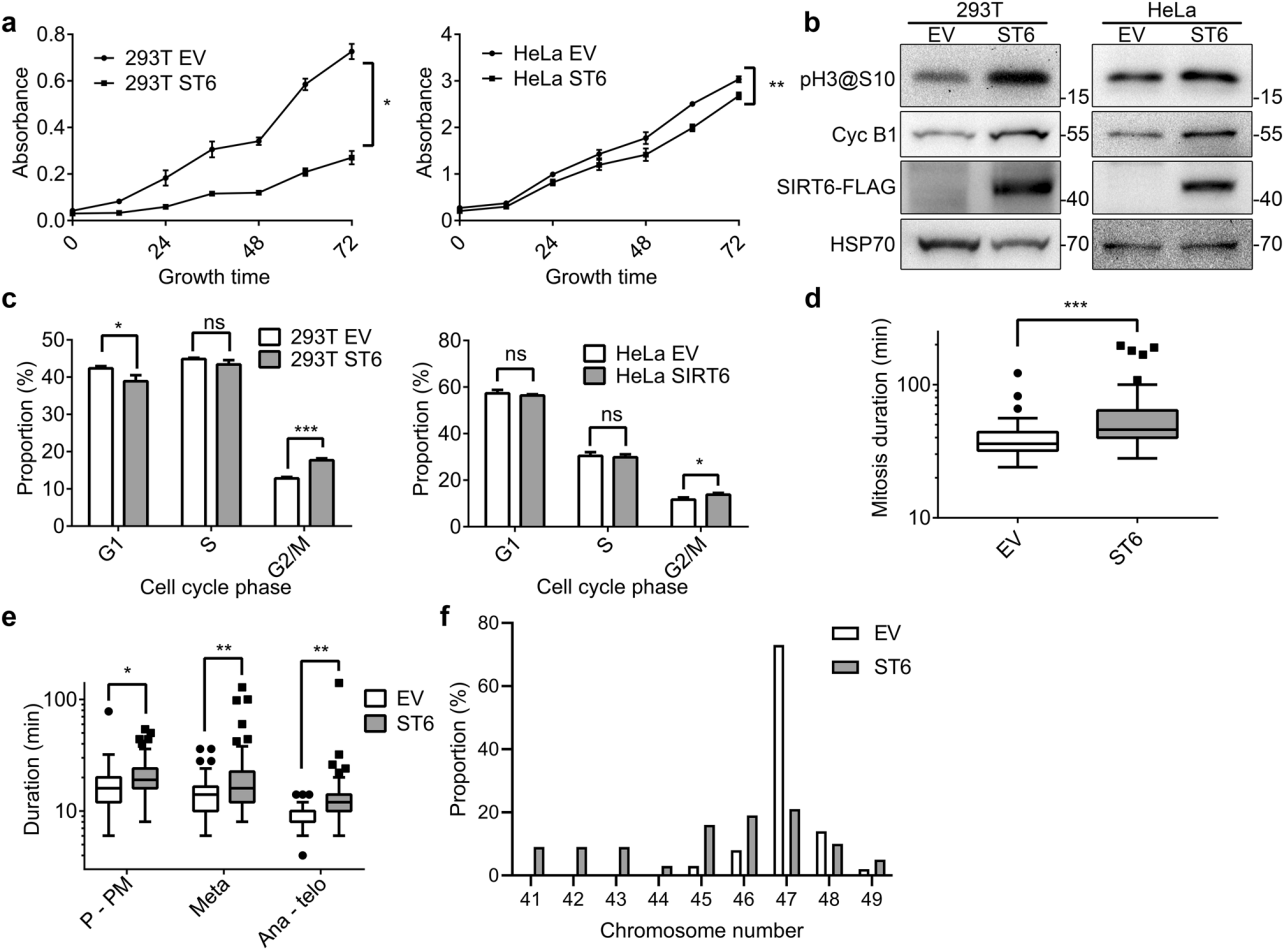
SIRT6 overexpression leads to abnormal mitosis. (**a**) Growth curves of control and SIRT6 overexpressing 293 T and HeLa cells. (**b**) Validation of SIRT6-FLAG by anti-FLAG blotting. Phosphorylation of histone H3 at Ser10 and cyclin B1 were also detected. HSP70 was used as a loading control. (**c**) Cell cycle distribution of control and SIRT6 overexpression in 293 T and HeLa cells. (**d**) Box-and-whisker plot showing the duration from NEB to completion of cytokinesis in 293 T stably expressing H2B.1-mCherry, which were transfected with empty vehicle (EV, n = 62) or SIRT6 overexpressing (ST6, n = 62) lentivirus. (**e**) Box-and-whisker plot showing the duration from NEB to chromosome alignment completion (P-PM), chromosome alignment maintenance (Meta), and anaphase onset to cytokinesis completion (Ana-telo) of cells described in d. (**f**) MCF10A cells were infected with SIRT6 overexpressing (n = 58) or empty vehicle (n = 63) lentivirus. Chromosomes of metaphase spreads were counted within 50 generations.

To validate effect of SIRT6 overexpression on cell morphology, we first explored whether SIRT6 overexpression affects cell proliferation. Cell proliferation rates were measured using CCK-8 assay in empty vehicle-transfected (EV) and SIRT6 overexpressing (ST6) 293 T and HeLa cells. SIRT6 overexpression in both 293 T and HeLa cells significantly inhibited cell proliferation (Fig. 1a). Although SIRT6 overexpression was reported to induce apoptosis and cell death^11^, we also noticed that there were more mitotic cells in SIRT6 overexpressing cells, indicating that SIRT6 overexpression caused cell cycle arrest. To further examine this phenomenon, western blotting was carried out and showed that SIRT6 overexpression led to the elevation of G2/M marker cyclin B1 and phosphorylation of histone H3 at Ser10, a marker of chromosome condensation during mitosis, indicating arrest in the M phase in SIRT6 overexpressing cells (Fig. 1b). Fluorescence-activated cell sorting (FACS) analysis also showed an increase in G2/M proportion in SIRT6 overexpressing 293 T and HeLa cells (Fig. 1c).

To further investigate the effects of SIRT6 on mitosis, we monitored mitotic progression by time-lapse imaging of 293 T cells stably expressing mCherry-tagged histone H2B.1, and measured durations from prophase to prometaphase, metaphase and anaphase to telophase, along with durations of whole mitosis. We found that SIRT6 overexpression (ST6) caused a significant increase in mitosis duration, compared with control cells (EV) (Fig. 1d, Supplementary Fig. S1). The average duration in mitosis increased from 40.1 ± 1.9 min in control cells (n = 62) to 59.0 ± 4.7 min in SIRT6 overexpressing cells (n = 62; Fig. 1d). We further found that although the durations of all phases increased (Fig. 1e), the progression of anaphase and telophase were mostly affected (Table 1). This indicates that SIRT6 may affect regulators of mitosis, especially during anaphase and telophase.

**Table 1:** Ratios of mean duration of totasl M phase and different sub-phases in SIRT6 overexpressing/control cells.

M phase	1.47
Prophase & prometaphase	1.26
Metaphase	1.57
Anaphase & telophase	1.71

As mitosis abnormalities could lead to chromosome missegregation and aneuploidy, we next analyzed whether SIRT6 overexpression led to aneuploidy in MCF10A cells. Chromosomes of metaphase spreads in control (EV) and SIRT6 overexpressing (ST6) MCF10A cells were counted, and proportions of cells with different numbers of chromosomes were plotted. We found that overexpression of SIRT6 (western blotting verification shown in Fig. S2) increased the incidence of cells with different numbers of chromosomes (Fig. 1f). This result indicated that overexpression of SIRT6 induced chromosomal instability.

### SIRT6 interacts with APC/C

As mitosis and chromosome segregation were affected by SIRT6 overexpression, we further investigated SIRT6 function during mitosis, with an AP-MS experiment in SIRT6-FLAG overexpressing and control 293 T cells, treated with paclitaxel to synchronize the cells to M phase. SIRT6-FLAG and its interacting proteins were immune-precipitated and detected with liquid chromatography-tandem mass spectrometry (LC–MS/MS). The experiment identified 440 candidate interacting proteins (Supplementary Table S1). The identified proteins included the known SIRT6-interacting protein G3BP1, validating our approach. The candidate interacting proteins were mapped according to physical interactions using the STRING interaction database (http://string-db.org/)^12^ (Fig. S3). The results showed that five of APC/C subunits were identified as possible SIRT6-interacting proteins.

**Figure 2:**
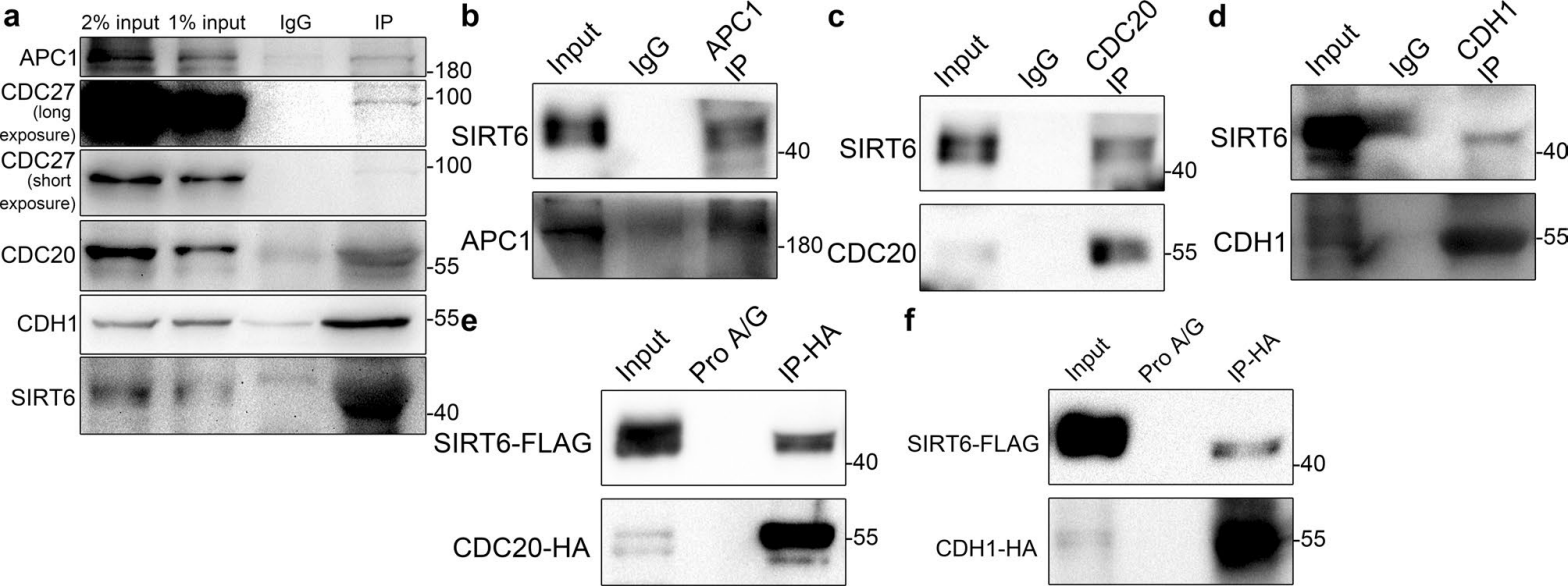
SIRT6 interacts with APC/C. (**a**) Lysates of asynchronous HeLa cells were immunoprecipitated (IP) with an anti-SIRT6 antibody or normal IgG, and then the immunoprecipitates were analyzed by immunoblotting. (**b–d**) Lysates of asynchronous HeLa cells were immunoprecipitated with anti-APC1 (b), anti-CDC20 (c), anti-CDH1 (d) antibody, or normal IgG, and then the immunoprecipitates were analyzed by immunoblotting. (**e**, **f**) Lysates of HeLa cells stably expressing CDC20-HA/CDH1-HA along with SIRT6-FLAG were immunoprecipitated with anti-HA antibody, and then the immunoprecipitates were analyzed by immunoblotting. Protein A/G was used as an unspecific control. Images of CDC20-HA, SIRT6-FLAG and CDH1-HA with the short exposure time are shown in the original image section in the supplementary file.

Next, we performed a series of co-immunoprecipitation experiments to verify the interaction between SIRT6 and APC/C. Figure 2a shows that APC1, Cdc27, CDH1, and CDC20 were detected in the SIRT6 immunoprecipitates and, conversely, SIRT6 was detected in APC1, CDH1 or CDC20 immunoprecipitates (Fig. 2b–d). Similar results were obtained for co-immunoprecipitation experiments using ectopically expressed proteins (Fig. 2e,f). These results prove that SIRT6 interacts with APC/C.

### APC/C enhances degradation of SIRT6 through D-box-dependent ubiquitination

**Figure 3:**
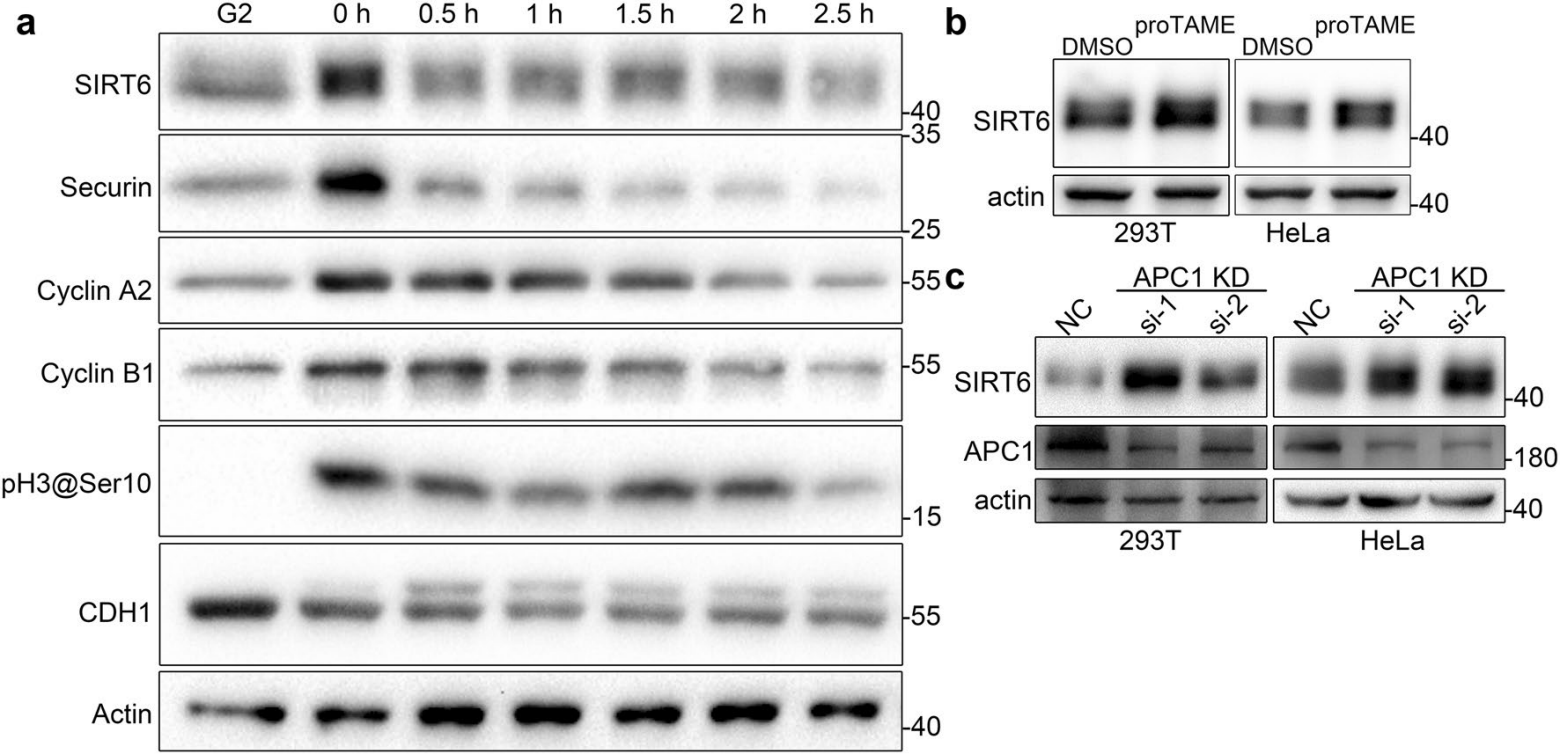
APC/C leads to SIRT6 decrease. (**a**) HeLa cells were synchronized to the G2 phase and samples were collected with the procedure shown in Supplementary Fig. S4. G2 samples were collected without release from RO-3306 and other cells were released. The time when most cells entered mitosis was designated as 0 h when mitotic cells were physically detached, and detached cells were cultured separately and sampled at 0.5 h intervals from 0 to 2.5 h. Changes in the indicated proteins were detected by immunoblotting. Actin was used as a loading control. (**b**) 293 T and HeLa cells were treated with 10 μM proTAME or DMSO for 12 h. SIRT6 levels were examined by immunoblotting. Actin was used as a loading control. (**c**) 293 T and HeLa cells were transfected with non-specific control (NC) or APC1 knockdown siRNAs. SIRT6 levels were examined by immunoblotting. Actin was used as a loading control.

To examine whether SIRT6 is a substrate of APC/C, wild-type HeLa cells were synchronized to the G2 phase using a double-thymidine and RO-3306 block protocol, with procedure illustrated in Supplementary Fig. S4 and described in methods section ^13^, and G2 cells were released to normal media, allowing cells to proceed through mitosis, and harvested at 0.5 h intervals for 2.5 h (Fig. 3a). Consistent with a previous report^4^, our results showed that the level of SIRT6 increases at the beginning of mitosis, and decreases as cells exit mitosis, especially at 0.5 h and 2.5 h, similar to known APC/C substrates securin, cyclin A2, and cyclin B1. Levels of SIRT6 mRNA were also measured during this procedure, which especially showed no significant difference between 0–0.5 h and 2–2.5 h, confirming that the decrease of SIRT6 was not induced by its mRNA transcription (Supplementary Fig. S5). Treatment of cells with proTAME, a cell-permeable APC/C inhibitor, led to an increase in SIRT6 (Fig. 3b). Knockdown of the key APC/C subunit APC1 led to SIRT6 upregulation (Fig. 3c). Collectively, these results suggest that APC/C down-regulates SIRT6.

**Figure 4:**
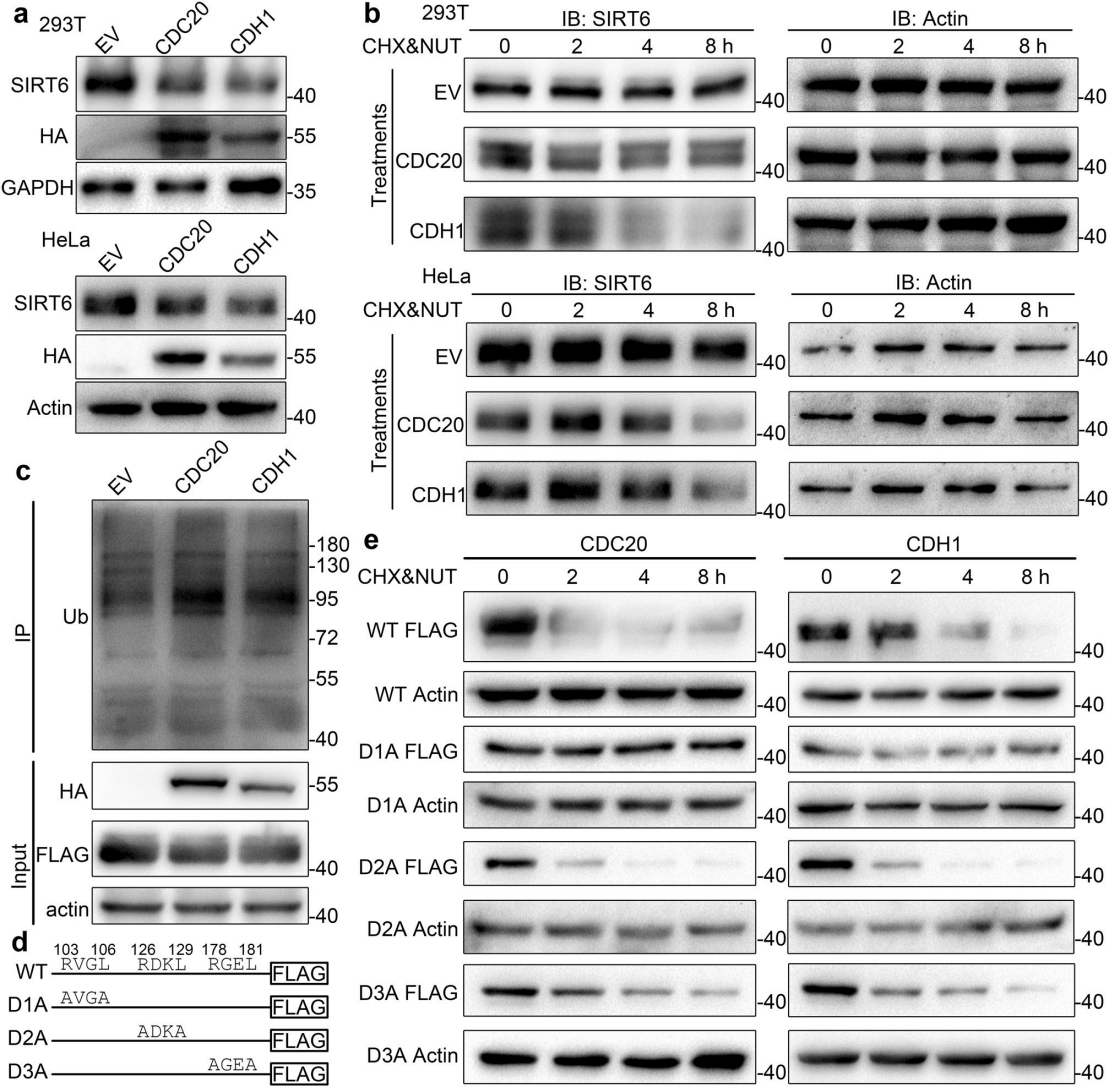
The APC/C leads to SIRT6 degradation through D-box-dependent ubiquitination. (**a**) 293 T and HeLa cells were transfected with empty vehicle and CDC20/CDH1 overexpressing plasmids, respectively. SIRT6 levels were examined by immunoblotting. Actin was used as a loading control. (**b**) 293 T and HeLa cells transiently transfected with empty vehicle (EV) or CDC20/CDH1 overexpressing plasmids were treated with 5 μg/mL cycloheximide (CHX) and 10 μM nutlin-3 (NUT) simultaneously for 0, 2, 4, and 8 h. SIRT6 levels were examined by immunoblotting. Actin was used as a loading control. (**c**) HeLa cells expressing SIRT6-FLAG and myc-Ub were transfected with EV, CDC20-HA, or CDH1-HA overexpressing plasmids, respectively. Cell lysates were subjected to immunoprecipitation with an anti-FLAG antibody and immunoblotted with an anti-ubiquitination antibody. Whole cell lysates were probed with anti-HA and anti-FLAG antibodies. Actin was used as a loading control. This experiment was repeated four times. (**d**) Schematic of wild-type SIRT6 protein showing the positions of the three D-boxes, as well as the D1A (R103A, L106A), D2A (R126A, L129A) and D3A (R178A, L181A) mutants. (**e**) HeLa cells stably expressing FLAG-tagged wild-type or three D-box mutants were transfected with CDC20-HA or CDH1-HA overexpressing plasmids, respectively. Cell were then treated with 5 μg/mL cycloheximide (CHX) and 10 μM nutlin-3 (NUT) simultaneously for 0, 2, 4, and 8 h. Wild-type and mutated SIRT6 protein levels were monitored with anti-FLAG immunoblotting. Actin was used as loading control.

As activity of APC/C is regulated by two co-activators, CDC20 and CDH1, we next investigated which of them regulates the degradation of SIRT6. Overexpression of both CDC20 and CDH1 led to a decrease in SIRT6 (Fig. 4a).To confirm whether CDC20 and CDH1 lead to SIRT6 degradation, 293 T and HeLa cells were transfected with an empty vehicle (EV), or CDC20- or CDH1-expressing plasmids, treated with cycloheximide and nutlin-3 (to inhibit MDM2, a known E3 of SIRT6^14^) for 0, 2, 4, and 8 h, and SIRT6 levels were tested using western blotting. The results showed that SIRT6 was degraded at a faster rate in the presence of CDC20 and CDH1, compared with empty vehicles (Fig. 4b). These results indicate that both CDC20 and CDH1 mediate the degradation of SIRT6.

Along with other components of APC/C, CDC20 and CDH1 interact with and lead to polyubiquitylation of their substrates^15,16^. Therefore, we assessed whether CDC20 and CDH1 could promote polyubiquitylation of SIRT6. HeLa cells stably expressing SIRT6-FLAG were transfected with myc-ubiquitin plasmids, combined with empty vehicle (EV), CDC20-HA, or CDH1-HA-expressing plasmids. Twenty-four hours after transfection, cells were treated with MG-132 for another 12 h and harvested for immunoprecipitation with an anti-FLAG antibody. Immunoprecipitation samples were separated by SDS-PAGE and detected with an anti-ubiquitination antibody. The results showed that CDC20 and CDH1 overexpression led to an increase in SIRT6 ubiquitination compared with empty vehicle (Fig. 4c, quantification of Ub shown in Supplementary Fig. S6), demonstrating that CDC20 and CDH1 overexpression enhances the ubiquitination of SIRT6.

Both CDC20 and CDH1 interact with their substrates through a motif known as D-box, which contains a simple core sequence of RxxL^17^. Analysis of the SIRT6 protein sequence revealed three conserved putative D-box motifs (Fig. 4d). D-box-dependent substrate degradation can be ablated by mutating the arginine and leucine residues within the RxxL motif to alanine^18^, thus we performed site-directed mutagenesis to generate mutants in which Arg and Leu of the D-boxes were mutated to Ala, respectively, and designated them as D1A, D2A and D3A (Fig. 4d). CHX chase experiment (treatment of CHX and nutlin-3 same as in Fig. 4b) demonstrated that half-life of the D1A mutation was substantially increased compared with wild-type SIRT6 and other mutations upon CDC20 and CDH1 overexpression. The experiment indicates that the first D-box motif of SIRT6 is necessary for its degradation by CDC20 and CDH1 (Fig. 4e).

### SIRT6 deacetylates CDH1 and enhances its degradation

**Figure 5:**
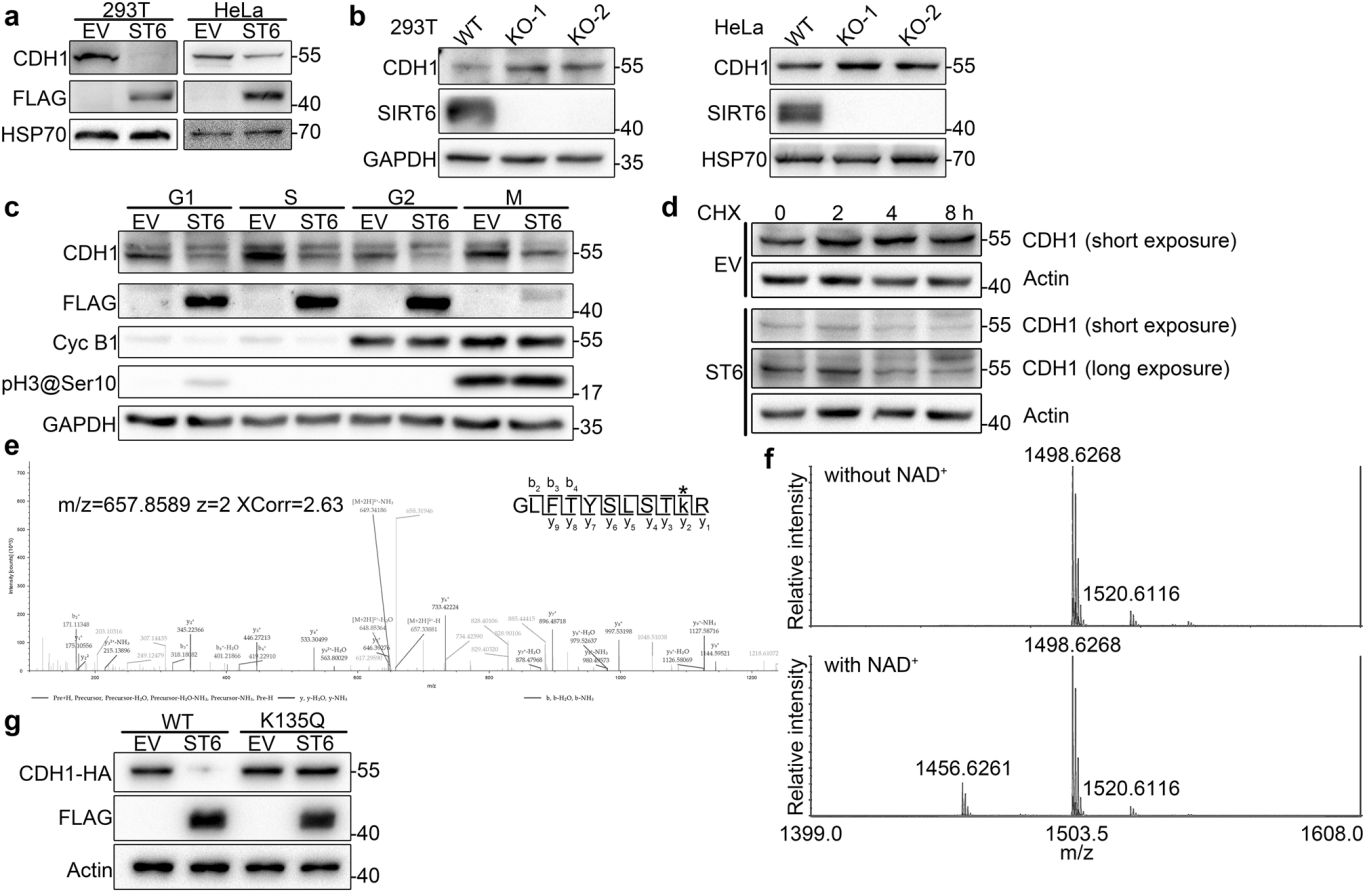
SIRT6 deacetylates CDH1, a co-activator of APC/C, and promotes its degradation. (**a**) Control and SIRT6-FLAG overexpressing 293 T and HeLa cells were subjected to western blotting with the indicated antibodies. HSP70 was used as a loading control. (**b**) Level of CDH1 protein was detected by western blotting in wild-type and SIRT6 knockout 293 T and HeLa cells. GAPDH and HSP70 were used as loading controls. (**c**) Control (EV) and SIRT6-overexpressing (ST6) HeLa cells were synchronized to G1, S, G2 and M phases, respectively. Levels of CDH1, FLAG, cyclin B1 and phosphorylation of histone H3 at Ser10 (pH3@Ser10) were detected with corresponding antibodies. GAPDH was used as loading control. (**d**) Control (EV) and SIRT6-overexpressing (ST6) cells were treated with 5 μg/mL cycloheximide (CHX) for 0, 2, 4, and 8 h. Levels of CDH1 were detected by western blotting. Actin was used as loading control. (**e**) Tandem mass spectrometry spectrum of the acetylated peptide for identification of Lys135 modifications in CDH1. An asterisk in the peptide sequence indicates the modified lysine residue. The image was generated with Xcalibur 3.0 (Thermo Fisher Scientific Inc.). (**f**) Synthesized peptide around CDH1 K135 with acetylation was incubated with SIRT6 without/with NAD^+^. Samples were analyzed with MALDI-TOF/TOF with m/z range 1400–1600 (z = 1). (**g**) 293 T stably expressing wild-type or K135Q CDH1-HA were transfected with empty vehicle (EV) or SIRT6-FLAG expressing vector (ST6), respectively. CDH1-HA levels were detected using anti-HA antibody. Expression of SIRT6-FLAG was detected using an anti-FLAG antibody. Actin was used as a loading control.

Considering that SIRT6 interacted with CDH1 far more than other tested APC/C subunits (Fig. 2a), and level of CDH1 decreased as level of SIRT6 increased at 0 h in Fig. 3a, we investigated whether changes in SIRT6 levels would affect CDH1. Surprisingly, the level of CDH1 decreased in both 293 T and HeLa cells with overexpression of SIRT6 and increased in cells with knockout of *Sirt6*, especially in 293 T cells (Fig. 5a,b). Levels of CDC20 were also examined in SIRT6 overexpressing and knockout cells, showing no significant changes (Supplementary Fig. S7 & S8). The off-target effect of sgRNAs used for knockout and deactivated SIRT6 did not affect CDH1 level, as confirmed by rescue experiments (Supplementary Fig. S9). As the level of CDH1 is regulated during cell cycle^19,20^, and SIRT6 overexpression leads to change in cell cycle distribution, it is necessary to confirm if SIRT6 affects CDH1 through its modulation of cell cycle distribution. We found that downregulation of CDH1 by SIRT6 is independent of mitosis arrest, as SIRT6 overexpression leads to the CDH1 decrease in synchronized cells (Fig. 5c).

Next, we explored whether SIRT6 leads to downregulation of CDH1 through mRNA transcription, protein synthesis, or protein degradation. *Cdh1* mRNA levels did not change significantly in SIRT6 overexpressing (Supplementary Fig. S10) or knock-out cells (Supplementary Fig. S11). In 293 T cells ectopically expressing CDH1-HA under the CMV-F promoter, SIRT6-FLAG was transiently overexpressed, leading to a decrease in CDH1-HA (Supplementary Fig. S12). These results indicate that SIRT6 overexpression/knock-out affect CDH1 at the protein level but not through the regulation of *Cdh1* mRNA transcription. In SIRT6-overexpressing cells treated with cycloheximide whose protein synthesis was inhibited, the level of CDH1 decreased as treatment was elongated, indicating that CDH1 degradation was enhanced upon SIRT6 overexpression (Fig. 5d).

To further investigate the mechanisms underlying SIRT6-enhanced CDH1 degradation, we characterized changes in post-translational modifications (PTM) of CDH1 upon *Sirt6* knockout. CDH1-HA in wild-type and SIRT6 knock-out cells was immune-precipitated, and PTM changes of CDH1-HA were analyzed with LC–MS/MS. This experiment demonstrated that acetylation of the Lys135 residue increased upon *Sirt6* knockout, indicating that it was a SIRT6 deacetylation site. Spectrum of the peptide containing acetylated K135 was shown in Fig. 5e, and corresponding areas and normalized ratios of the peptide were shown in Table 2. To directly prove that CDH1 is a SIRT6 substrate, an in vitro deacetylation assay was performed with synthesized CDH1 fragment peptide containing acetylated K135 and purified SIRT6 protein. The result showed that acetylation of CDH1 K135 can be deacetylated by SIRT6 (Fig. 5f). To further confirm the involvement of Lys135 in CDH1 degradation, Lys135 of CDH1 was mutated to glutamine, mimicking function of acetylated lysine. 293 T cells stably expressing wild-type and K135Q CDH1-HA proteins were transfected with plasmids expressing SIRT6. Cells were harvested 48 h after transfection, and the levels of CDH1-HA proteins were detected using anti-HA antibody in western blot analyses. The results showed that mutation of Lys135 to glutamine abolished SIRT6-mediated CDH1 degradation, demonstrating that the Lys135 residue is essential for this process (Fig. 5g). These results indicate that SIRT6 promotes degradation of CDH1 protein through deacetylation of CDH1 K135.

**Table 2:** Areas and normalized ratios of acetylated CDH1 K135 peptide in control and SIRT6-knockout 293 T cells.

Sample	Knock-out 1	Knock-out 2	Knock-out 3
Peptide area	1.43e6	1.55e6	1.7e6
Protein area	2.46e10	2.51e10	3.33e10
Sample	Control 1	Control 2	Control 3
Peptide area	5.29e5	Not found	5.76e5
Protein area	2.13e10	1.7e10	2.21e10
Normalized KO/Ctrl ratio	2.34	N/A	1.96

### SIRT6 overexpression leads to centrosome hyper-amplification associated with increased levels of Aurora kinase A

**Figure 6:**
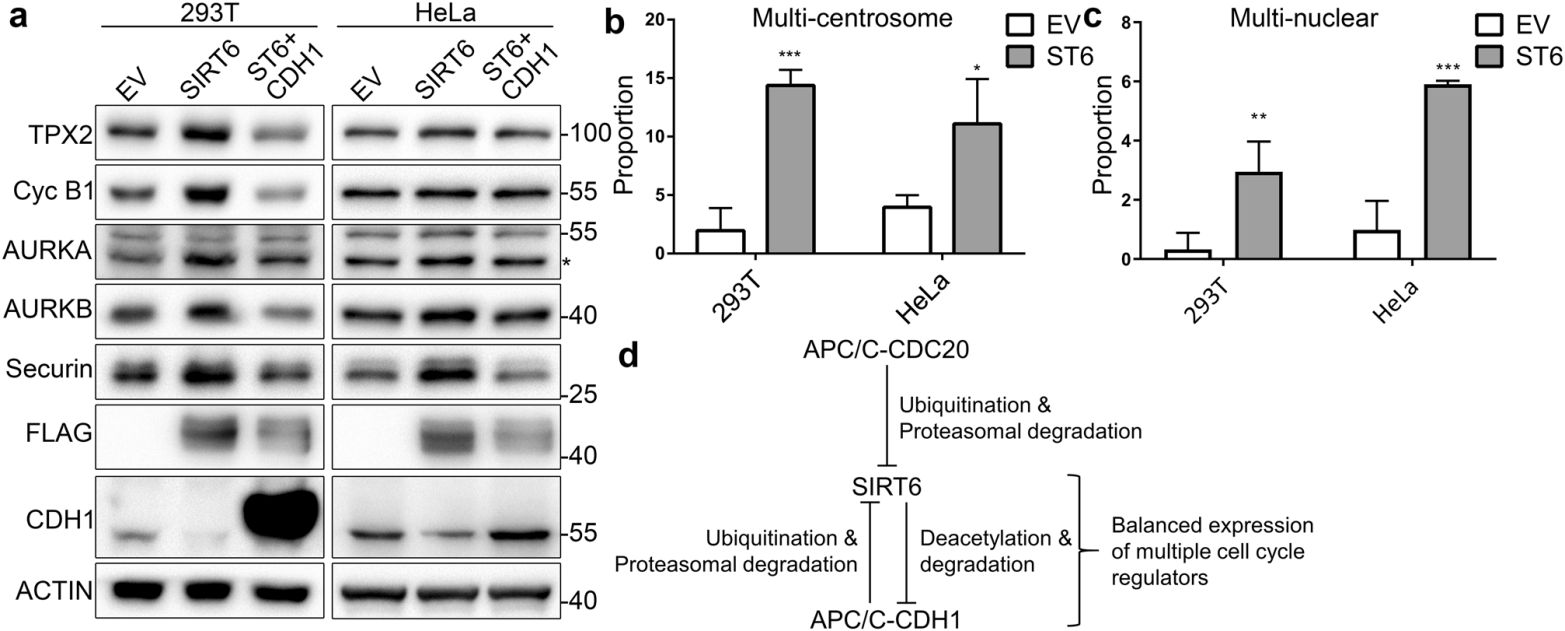
Overexpression of SIRT6 leads to up-regulation of various APC/C substrates and defective mitosis. (**a**) 293 T and HeLa cells stably expressing empty vehicle (EV), SIRT6-FLAG (SIRT6), SIRT6-FLAG, and CDH1-HA simultaneously (ST6 + CDH1) were treated with thymidine block and subjected to western blotting. Levels of TPX2, cyclin B1, AURKA, AURKB, securin, FLAG, and CDH1 were detected using the corresponding antibodies. Actin was used as a loading control. Short exposures of 293 T CDH1 are shown in the original image section of the supplementary file. (**b**) Proportions of cells with three centrosomes or more were counted in control and SIRT6 overexpressing 293 T and HeLa cells (n = 100). (**c**) Proportions of cells with two or more nuclears were counted in control and SIRT6 overexpressing 293 T and HeLa cells (n = 100). (**d**) Mechanistic illustration of the current study.

The presence of aneuploid cells with SIRT6 overexpression (Fig. 1f) suggests that the integrity of chromosome segregation during mitosis is compromised. As a number of mitosis regulators are substrates of APC/C-CDH1, we investigated whether these proteins change upon SIRT6 overexpression. The results showed that TPX2, cyclin B1, Aurora kinase A, Aurora kinase B, and securin increased as CDH1 decreased in SIRT6 overexpressing cells, and decreased when CDH1 was ectopically replenished (Fig. 6a). As overexpression of Aurora kinase A was reported to induce centrosome amplification and aneuploidy^21^, we compared the centrosome numbers of SIRT6 overexpressing and control 293 T and HeLa cells. Our data showed that approximately 14% of overexpressing 293 T and 11% of overexpressing HeLa cells had three or more centrosomes, compared with 2% and 4% in control 293 T and HeLa cells (Fig. 6b). Also, proportion of cells with two or more nuclears also increased in SIRT6 overexpressing 293 T and HeLa cells (Fig. 6c). These results indicate that overexpression of SIRT6 induces chromosomal instability by upregulating APC/C-CDH1 substrates, which play a pivotal role during mitosis.

## Discussion

The mechanisms investigated in our study are summarized in Fig. 6d. In short, we identified SIRT6 as a novel substrate of APC/C, a key mitosis regulator, and SIRT6 inhibits APC/C activity by deacetylating its coactivator CDH1 and enhancing the degradation of CDH1. As a result of APC/C inhibition, cells overexpressing SIRT6 have altered mitosis duration, hyper-amplified centrosomes, and reduced chromosome stability, which has been well studied to induce carcinogenesis^3,22^.

Previous reports have identified the participation of SIRT6 in mitosis, although almost all regarded SIRT6 as a mitosis guardian. The first cell cycle-specific study of SIRT6 by Fengyi Liang discovered its co-localization with mitotic spindles and fluctuated levels during mitosis, indicating the participation and regulation of SIRT6 during mitosis^4^. A subsequent study reported an increase in histone H4K16 acetylation, meiotic spindle deficiency, and aneuploidy in SIRT6-depleted oocytes^6^. The latest study identified pericentric histone H3K18ac as a substrate of SIRT6, and this activity maintained pericentric chromatin silencing and prevented aberrant accumulation of satellite transcriptions, which further obviated mitosis defects such as multipolar mitoses and supernumerary centrosomes^5^. However, the mechanism of SIRT6 level regulation and centrosome amplification caused by SIRT6 aberration remain to be elucidated, while our study has shed light on these questions.

Cells overexpressing SIRT6 display genetic instability and abnormal mitosis. Our analysis indicates that deacetylation of CDH1 by SIRT6 induces its degradation, which decreases APC/C activity. In contrast, SIRT6 knockout enhances stability of CDH1, leading to activation of APC/C. These data indicate that SIRT6 is a negative regulator of APC/C-CDH1 activity. Interestingly, previous reports on SIRT6 knockdown/knock-out cells also described mitotic defects and genomic instability. This indicates that the maintenance of mitotic fidelity and genomic stability by SIRT6 may be dose-dependent, as aberrantly high or low levels of SIRT6 lead to abnormal cellular functions. This is consistent with studies of AURKA functions, as both overexpression and depletion of AURKA lead to abnormal centrosome and mitotic spindle function, resulting in aneuploidy^23^. Furthermore, in our study, SIRT6 was also found to be a substrate of APC/C-CDC20/CDH1. Along with other regulation pathways, this reciprocal downregulation between SIRT6 and APC/C may form a bi-stable switch through which cells maintain the mitotic state while the SIRT6 level is high enough to inhibit APC/C, and exit mitosis when SIRT6 is degraded by APC/C.

As the levels of several APC/C target proteins in human cells increase after SIRT6 overexpression, including some key mitosis regulators such as TPX2, AURKA, AURKB, cyclin B1, and securin, genomic instability caused by SIRT6 overexpression can be a combined effect of these regulators. Although it is impossible to investigate the actual influence of SIRT6 on each of these regulators, or their impact on the SIRT6-dependent phenotypes observed in this study, we chose to present AURKA as a mechanistic example. To date, a considerable number of reports have indicated AURKA in genomic instability and cancer. Overexpression of AURKA leads to centrosome hyper-amplification and tumor formation. AURKA overexpression also activates the Akt-mTOR pathway, which is involved in a transformed phenotype^23^. Overexpression of AURKA is frequently detected in different human cancers. In addition, high AURKA expression was reported to be associated with decreased survival or poor prognosis^24–26^.

In summary, we demonstrated reciprocal regulation between SIRT6 and APC/C. While APC/C-CDC20/CDH1 enhances SIRT6 degradation through ubiquitination, SIRT6 regulates APC/C activity by deacetylating its coactivator, CDH1, and enhances its degradation (Fig. 6d). In addition, SIRT6 overexpression leads to centrosome hyper-amplification and genomic instability via AURKA, which is stabilized when CDH1 is downregulated. Thus, these results indicate SIRT6 as a possible oncogene and uncover an essential role for SIRT6 in maintaining the integrity of mitosis by negatively regulating APC/C activity, dysfunction of which leads to genetic instability.

## Methods

### Cell culture

Human embryonic kidney 293 T, human adenocarcinoma HeLa, and human mammary epithelial MCF10A cell lines were obtained from the cell bank of the Chinese Academy of Sciences (Shanghai, China). 293 T and HeLa cells were grown in DMEM medium (#317-005-CL; Wisent, Montreal, QC, Canada) supplemented with 10% fetal bovine serum (#ST30-3302; PAN, Aidenbach, Germany) and 1% streptomycin/penicillin (#15140122; Gibco, Waltham, MA, USA). MCF10A cells were grown in DMEM/F12 medium (#319-075-CL, Wisent) supplemented with 5% horse serum (#16050130; Life Technologies, Grand Island, NY, USA), 20 ng/mL epidermal growth factor (EGF), 0.5 mg/mL hydrocortisone, 10 μg/mL insulin, and 1% streptomycin/penicillin. All cells were cultured at 37 °C in a humidified incubator with 5% CO2. For plasmid and siRNA transfection, cells were transfected with Lipofectamine 3000 (Life Technologies) according to the manufacturer’s instructions. The siRNA sequences used in the study are listed in Supplementary Table S2.

### Establishment of stable SIRT6 overexpression cell lines

Human SIRT6 cDNA was synthesized from the total RNA of the 293 T cell line. The SIRT6 coding region with a FLAG-tag sequence was cloned into the pLVX-IRES-ZsGreen1 vector. A blank pLVX-IRES-ZsGreen1 vector was used as a control. Production of lentiviral particles of recombinant SIRT6 was carried out based on the protocol of Tiscornia et al.^27^. Briefly, the main vectors pLVX-SIRT6-FLAG-IRES-ZsGreen1 and pLVX-IRES-ZsGreen1, with packaging and envelope vectors, were transfected into 293 T cells when they reached 70–90% confluence. After 48 h of culture, the cell culture supernatant was collected and concentrated with PEG6000. The precipitated lentiviral particles were resuspended in PBS. The lentiviral particles were then used to infect 293 T and HeLa cells in the presence of 6 μg/mL Polybrene for 12 h. Cells were then cultured in fresh medium for more than 96 h, and cells with ZsGreen1 fluorescence were sorted by flow cytometry.

### Establishment of SIRT6 knockout cell lines

Genome engineering for the creation of SIRT6 knockout cell lines was performed as previously described^28^. We designed sgRNAs according to the GeCKOv2 library^29^ and the target sequences are listed in Table S2.

### Cell proliferation assay with CCK-8

Cells were seeded in 96-well plates (3,000 cells per well). Cell proliferation rate was determined using the CCK-8 assay (#CK04; Dojindo Laboratories, Kumamoto, Japan) according to the manufacturer’s instructions. Optical density (OD) was measured 2 h after CCK-8 addition at 450 nm using a microplate reader when cells had grown for 0, 12, 24, 36, 48, 60, and 72 h.

### Quantitative real-time PCR (qPCR)

Total RNA was extracted with TRNzol universal reagent (#DP424; TIANGEN, Beijing, China) and reverse-transcribed to cDNA using a SuperRT one step RT-PCR kit (#CW0742S; CWBIO, Jiangsu, China) according to the manufacturer’s instructions. Real-time PCR was performed using a LightCycler 96 System (Roche, Basel, Switzerland) with UltraSYBR mixture (#CW0957H; CWBIO) according to the manufacturer’s instructions. *ACTB* was used as an internal control. Relative expression levels were calculated using double delta Ct analysis. Primer sequences used for qPCR are listed in Supplementary Table S2.

### Cell cycle synchronization

HeLa cells were synchronized to the G1, S, G2, and M phases according to procedures previously described^13^. Briefly, for G1 samples, HeLa cells were treated with 20 μM lovastatin for 24 h. For S samples, HeLa cells were treated with 100 mM thymidine for 14 h, followed by normal media for 9 h, then 100 mM thymidine for 14 h (double-thymidine block), and then cultured in normal media for 4 h. For G2 samples, cells were synchronized with double-thymidine block followed by normal media for 2 h, then treated with 10 μM RO-3306 for 10 h. For M samples, cells were treated with 10 μM paclitaxel for 12 h, and then the mitotic cells were physically detached and collected. To prepare samples for Fig. 3a, HeLa cells were synchronized by double-thymidine block and RO-3306 treatment for 10 h, and a dish of cells was collected as the G2 sample. The other cells were washed twice with normal medium and cultured in normal medium for about 45 min, when most cells entered mitosis. Mitotic cells were physically detached and cultured in normal medium for 0, 0.5, 1, 1.5, 2, and 2.5 h and collected for western blotting.

### Western blot analysis

For cell lysate preparation, ~ 9 × 10^6^ HeLa or 293 T cells were washed twice with ice-cold PBS and lysed in 300 μL RIPA lysis buffer (#R0020; Solarbio, Beijing, China), supplemented with 1% protease inhibitor cocktail (#B14002; Bimake, Shanghai, China) and 1% phosphatase inhibitor cocktail (#15,002; Bimake). Cells were sonicated and debris was then cleared by centrifugation at 12,000 rpm for 10 min at 4 °C. Supernatants were transferred to fresh tubes. Protein concentrations were determined using a BCA protein assay kit (Solarbio). Equal amounts of proteins were separated in a 10% SDS-PAGE gel and then transferred onto a PVDF membrane. Western blot analysis was carried out using the standard procedure.

### Immunoprecipitation

For immunoprecipitation, ~ 9 × 10^6^ cells were lysed in 1 mL IP lysis buffer (#P0013; Beyotime), supplemented with 1% protease inhibitor cocktail and 1% phosphatase inhibitor cocktail. Lysates were treated as described above, except for sonication. To precipitate endogenous proteins, lysates were incubated with rabbit or mouse anti-SIRT6/APC1/CDC20/CDH1 antibodies (as indicated in figure legends), respectively with recommended dilution at 4 °C overnight with end over end rotation, and protein A (for rabbit antibody, #22810; Pierce, Thermo Fisher Scientific Inc. Waltham, MA, USA) or protein G (for mouse antibody, #22851) agarose beads were added to precipitate antibody-target conjugates. Samples were incubated at 4 °C for a further 3 h. To precipitate FLAG or HA-tagged proteins, anti-FLAG or HA beads were incubated with cell lysates overnight at 4 °C. Beads were then rinsed with 1 mL IP lysis buffer four times and re-suspended in 1 × SDS-PAGE sample buffer and prepared for SDS-PAGE, followed by western blotting or in-gel digestion.

### Antibodies and reagents

Rabbit anti-SIRT6 (12486), rabbit anti-FLAG tag (2368), rabbit anti-cyclin B1 (12231), rabbit anti-phospho-histone H3 (Ser10), mouse anti-cyclin A2 (4656), rabbit anti-AURKB (3094) and cycloheximide (2112) were purchased from Cell Signaling Technology (Danvers, MA, USA). Rabbit anti-HSP70 (ab194360), rabbit anti-ubiquitin (ab19247), rabbit anti-γ-tubulin (ab179503), mouse anti-CDH1 (ab89535), rabbit anti-securin (ab79546), rabbit anti-AURKA (ab108353), and rabbit anti-securin (ab79546) were purchased from Abcam (Cambridge, MA, USA). Mouse anti-SIRT6 (sc-517196), mouse anti-Cdc27 (sc-9972), and mouse anti-CDC20 (sc-13162) were purchased from Santa Cruz Biotechnology, Inc. (Santa Cruz, CA, USA). Rabbit anti-APC1 (21748-1-AP) and rabbit anti-TPX2 (11741-1-AP) were purchased from ProteinTech (Chicago, IL, USA). Mouse anti-GAPDH (CW0100), mouse anti-HA tag (CW0092) and mouse anti-β-actin (CW0096) were purchased from Beijing Cowinbioscience Co. Ltd. (Beijing, China). Mouse anti-ubiquitin (05-944) was purchased from Merck Millipore (KGaA, Darmstadt, Germany). Mouse anti-α-tubulin (T5168) was purchased from Sigma-Aldrich (KGaA). Nutlin-3 (S1061), paclitaxel (S1150), colchicine (S2284) and MG-132 (S2619) were purchased from Selleck Chemicals (Houston, TX, USA).

### Immuno-fluorescence imaging

For centrosome morphology analysis, cells were cultured in a glass bottom cell culture dish and fixed with 4% paraformaldehyde in PBS for 10 min at room temperature. For α- and γ-tubulin double staining, cells were permeabilized with 0.5% saponin in PBS for 10 min, followed by blocking with 5% normal goat serum and 0.1% saponin in PBS at room temperature. Mouse anti-α-tubulin monoclonal antibody (1:2000), rabbit anti-γ-tubulin (1:500), and DAPI (Beyotime) were used. Fluorescence was detected using an UltraView VoX Spinning Disk imaging system equipped with a 60 × objective. Images of α- and γ-tubulin were acquired at 0.2 μm steps and collected using identical imaging systems. Images were processed using Imaris 9 (Bitplane Inc., Zurich, Switzerland).

### Chromosome aneuploidy and spread analyses

MCF10A cells were treated with 50 ng/mL colchicine for 6 h. Cells were collected and hypotonically swollen in 75 mM KCl for 45 min at 37 °C. Cells were fixed in freshly made Carnoys fixative solution (75% methanol and 25% acetic acid) with several changes of the fixative. Cells were dropped onto cooled glass slides and dried at room temperature. Chromosomes were stained with DAPI for 10 min, rinsed with PBS, air-dried, mounted and imaged. Chromosomes of each individual cell in control (EV) and SIRT6 overexpressing (ST6) cells were counted, and proportions of cells with different numbers of chromosomes in each group of cells were plotted.

### Time-lapse imaging and measurement of mitosis duration

293 T H2B-mCherry cell lines stably expressing SIRT6-FLAG and empty vehicle as control were seeded in a glass-bottom cell culture dish and cultured in DMEM with 10% FBS and antibiotics in a humidified culture chamber (5% CO_2_ at 37 °C). Differential interference contrast (DIC) and fluorescence images were collected every 2 min using the DeltaVision Elite imaging system (GE Healthcare, Barrington, IL, USA) with a 60 × NA 1.40 Plan Apochromat oil DIC objective, mCherry filter, and 0.05 s exposure for 10 h. Image sequences were viewed using ImageJ 1.53 software^30^, and cell behavior was analyzed manually. The time of landmark events were determined as previously reported^31^. Briefly, nuclear envelope breakdown (NEB) in each mitotic cell was set as time = 0, and the completion of chromosome alignment on the spindle equator was marked metaphase (M), the onset of chromosome-to-pole movement was marked anaphase onset (A), and the completion of cytokinesis was marked the end of mitosis (C). Three sections were recorded respectively for each individual cell: the time from NEB to M was recorded as prophase to prometaphase (P—PM), time from M to A as metaphase (Meta), and time from A to C as anaphase to telophase (Ana—telo). Box plots were generated according to durations of whole mitosis and three sections described above and outliers were defined with the Tukey method.

### In vitro deacetylation assay

Peptide around acetylated K135 of CDH1, with sequence TYSLSTK(Ac)RSSPDD was synthesized. For in vitro deacetylation assay, the peptide was incubated with commercially available recombinant SIRT6 enzyme, with/without NAD^+^ as cofactor. After incubation at 37 °C for 2 h, the samples were analyzed with MALDI.

### Mass spectrometry analysis

For LC–MS/MS analysis, the digestion product was separated by a 120 min gradient elution at a flow rate of 0.250 μL/min with an EASY-nLCII integrated nano-HPLC system (Proxeon, Odense, Denmark), which was directly interfaced to a Thermo Q-Exactive mass spectrometer (Thermo Fisher Scientific Inc.). The analytical column was a homemade fused silica capillary column (75 μH ID, 150 mm length; Upchurch, Oak Harbor, WA, USA) packed with C-18 resin (300 Å, 5 μm; Varian, Lexington, MA, USA). Mobile phase A consisted of 0.1% formic acid, and mobile phase B consisted of 100% acetonitrile and 0.1% formic acid. The mass spectrometer was operated in the data-dependent acquisition mode using Xcalibur 3.0 software (Thermo Fisher Scientific Inc.), and there was a single full-scan mass spectrum in the orbitrap (400–1800 m/z, 30 000 resolution) followed by 20 data-dependent MS/MS scans in the ion trap at 35% normalized collision energy (CID).

### Affinity purification—mass spectrometry (AP-MS) analysis

Samples were prepared as described in the section “Immunoprecipitation”, and separated by SDS-PAGE. The samples were in-gel digested and subjected to LC–MS/MS analysis. The MS/MS spectra from each LC–MS/MS run were searched against the UniProt human database^32^ (version 10 January 2015, 89,105 sequences) using an in-house Sequest HT algorithm in Proteome Discoverer 2.1 software (Thermo Fisher Scientific Inc.). The search criteria were as follows: full tryptic specificity was required; one missed cleavage was allowed; oxidation (M) was set as dynamic modification; carbamidomethylation (C) was set as fixed modification; precursor ion mass tolerances were set at 10 ppm for all MS acquired in an Orbitrap mass analyzer; and the fragment ion mass tolerance was set at 0.02 Da for all MS2 spectra acquired in the linear ion trap. The searched data were further processed with the Percolator function in Proteome Discoverer to filter with a 1% peptide false discovery rate (FDR). The SAINT algorithm^33^ (http://sourceforge.net/projects/saint-apms) was used to evaluate the MS data. Proteins with at least 2 unique peptides, SEQUEST score ≥ 40 in the overexpression sample and SAINT score ≥ 0.85 were considered as candidate interaction proteins. Candidate interactions were visualized using the STRING database (https://string-db.org/)12 with a threshold of 0.7.

### Identification of acetylation sites of CDH1

293 T WT and 293 T SIRT6 KO cells stably expressing CDH1-HA were lysed and subjected to anti-HA immunoprecipitation. IP samples were separated by SDS-PAGE and bands of CDH1-HA were sliced, in-gel digested, and subjected to LC–MS/MS analysis. Acquired spectra were searched using PEAKS Studio 8.5 (Bioinformatics Solutions Inc., Waterloo, ON, Canada) against the CDH1-HA protein sequence with the following criteria: full tryptic specificity was required; one missed cleavage was allowed; oxidation (M), carbamidomethylation (C), and acetylation (K) were set as dynamic modifications; precursor ion mass tolerances were set at 10 ppm for all MS acquired in an Orbitrap mass analyzer; and the fragment ion mass tolerance was set at 0.02 Da for all MS2 spectra acquired in the linear ion trap. Ratios of acetylated peptide were quantified with peak area and normalized to the total peak area of the whole protein.

### Data analysis

All immunoblots, co-immunoprecipitation, FACS data, and qPCR results were replicated three times, unless stated otherwise in the figure legends. GraphPad Prism version 7.00 software (GraphPad, San Diego, CA, USA, https://www.graphpad.com) was used for statistical analysis. All data are shown as mean ± SEM, and significant differences were determined by the Student’s t-test (*p < 0.05; **p < 0.01; ***p < 0.001). *p* values < 0.05 were considered significant.

## Supplementary Information

Supplementary Information.

## Data Availability

The mass spectrometry data have been deposited to the ProteomeXhange Consortium (http://proteomecntral.proteomexchange.org) via the PRIDE partner repository^34^ with the dataset identifier PXD022402 (Reviewer account: reviewer_pxd022402@ebi.ac.uk Password: tY4tbzy0) and PXD022500 (Reviewer account: reviewer_pxd022500@ebi.ac.uk Password: U58mgflK).
