# Supplementary material for: Reciprocal interaction between SIRT6 and APC/C regulates genomic stability
Source: Sci Rep. 2021 Jul 9;11:14253. doi: 10.1038/s41598-021-93684-w (PMC8270898; doi:10.1038/s41598-021-93684-w)

Supplementary Materials

For the article entitled:

Reciprocal interaction between SIRT6 and APC/C regulates genomic stability

Helin Wang[1], Kangze Feng[1], Qingtao Wang[2], Haiteng Deng[1, *]

[1]MOE Key Laboratory of Bioinformatics, Center for Synthetic and Systematic Biology, School of Life Sciences, Tsinghua University
[2]Beijing Chaoyang Hospital Affiliated to Capital Medical University, Beijing, 100043, China;

Contact:
Helin wang: whl0818@live.cn
Kangze Feng: fkz_2008@126.com
Qingtao Wang: wqt36@163.com
Haiteng Deng: dht@mail.tsinghua.edu.cn

* Correspondence: Haiteng Deng

Supplementary figures

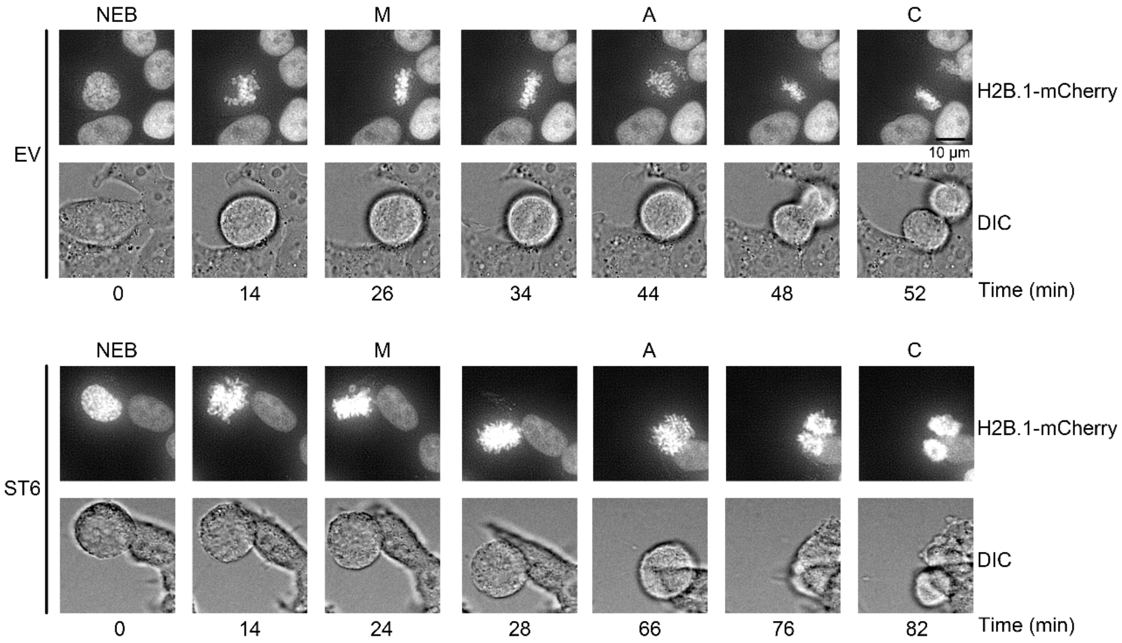

Figure S1. Selected frames from time-lapse movies of representative 293T H2B.1-mCherry transfected with SIRT6 overexpression lentivirus or empty vehicle. NEB, nuclear envelope breakdown; M, metaphase; A, anaphase. Images were viewed with ImageJ.[30]

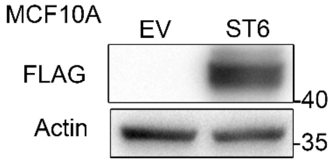

Figure S2. Western blot validation of SIRT6 overexpressing MCF10A cell line. Anti-FLAG antibody was used to blot expression of SIRT6-FLAG protein. Actin was used as a loading control.

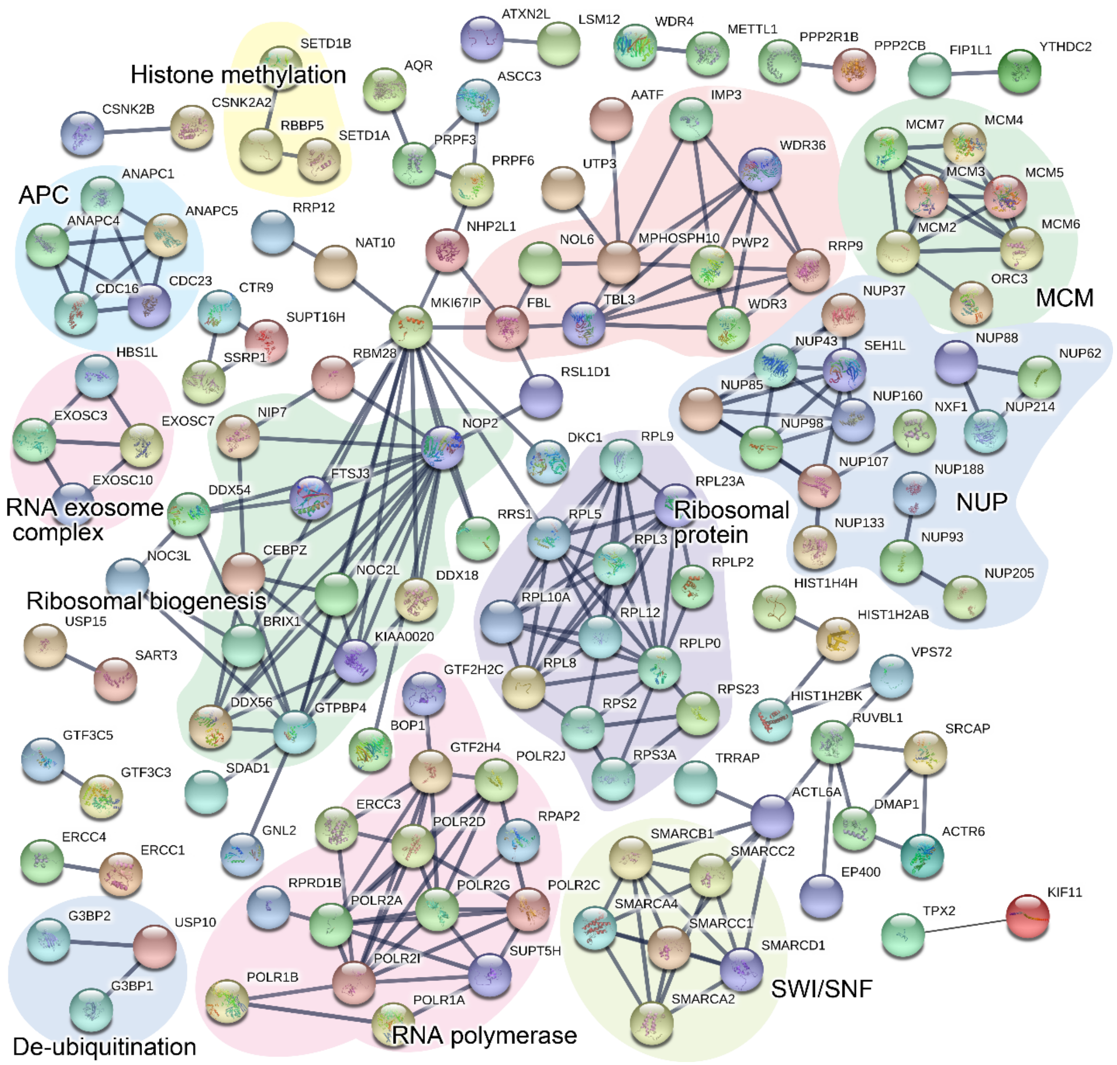

Figure S3. AP-MS analysis of SIRT6 interactions during mitosis. SIRT6-FLAG and interacting proteins were enriched from 293T cells synchronized to mitosis using paclitaxel and subjected to mass spectrometry analysis. Identified protein complexes were reconstructed using the STRING database. Shading indicates proteins belonging to a common cellular process or a complex. This experiment was conducted once.

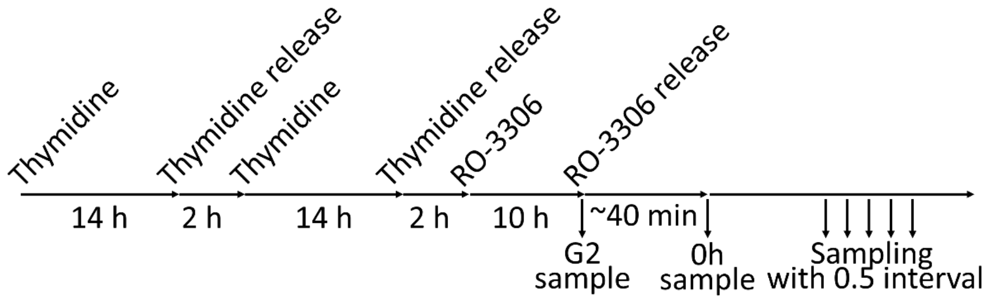

Figure S4. Procedure of cell treatments for preparing samples of figure 4A and S5. Detailed method was described in materials and methods.

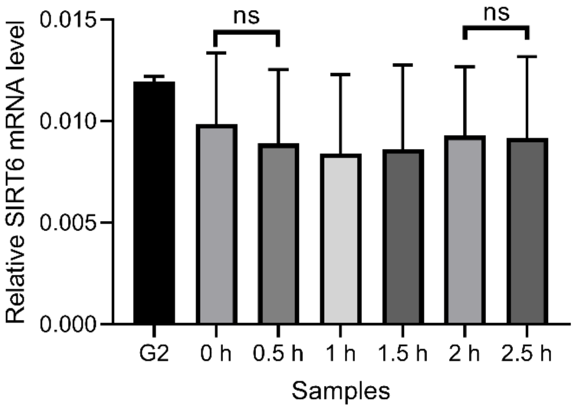

Figure S5. Quantitative real-time PCR quantifying the mRNA levels of SIRT6 in cells collected following the procedure shown in Figure S4.

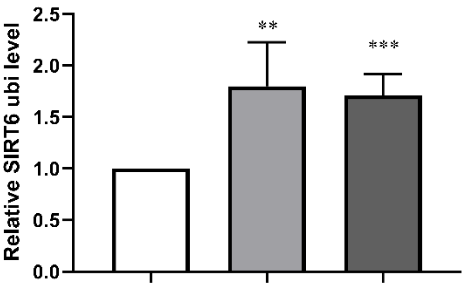

Figure S6. Quantification analysis of relative SIRT6 ubiquitination level in Figure 4c. Grayscale band volume was measured with Image Lab 5.2.1 (Bio-Rad Laboratories, Inc.).

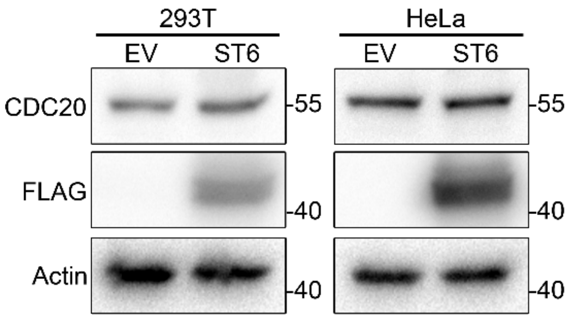

Figure S7. Control and SIRT6-FLAG overexpressing 293T and HeLa cells were subjected to western blotting with the indicated antibodies. Actin was used as a loading control.

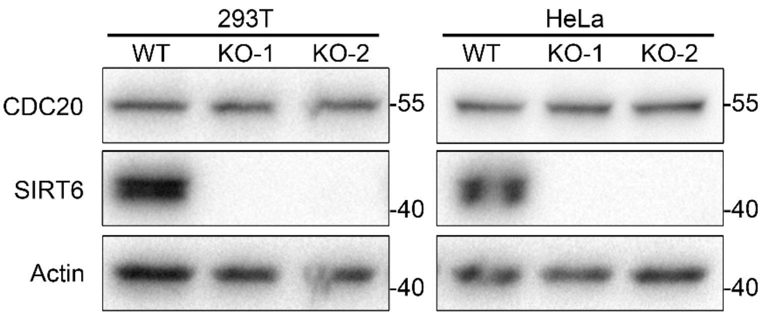

Figure S8. Level of CDC20 protein was detected by western blotting in wild-type and SIRT6 knockout 293T and HeLa cells. Actin was used as a loading control.

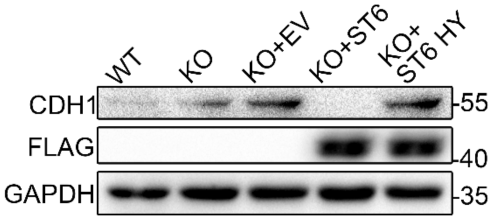

Figure S9. Wild-type 293T cells, 293T SIRT6 knock-out cells, and knock-out cells transfected with empty vehicle (KO+EV), wild-type SIRT6 (KO+ST6), or inactive mutation SIRT6 H133Y (KO+ST6 HY) were subjected to western blotting using anti-CDH1 antibody. Anti-FLAG antibody was used to detect SIRT6-FLAG expression. GAPDH was used as a loading control.

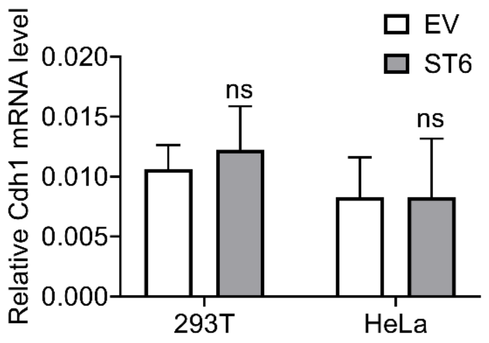

Figure S10. Quantitative real-time PCR quantifying the mRNA levels of Cdh1 in 293T and HeLa cells transfected with SIRT6 overexpressing lentivirus (ST6) or empty vehicle (EV).

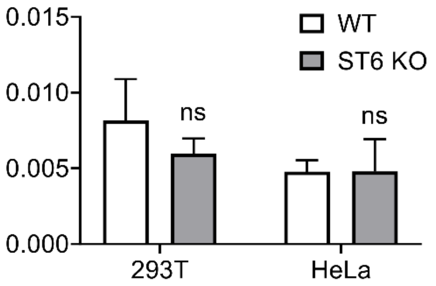

Figure S11. Quantitative real-time PCR quantifying the mRNA levels of Cdh1 in wild-type 293T and HeLa cells (WT) or SIRT6 knockout cells (KO).

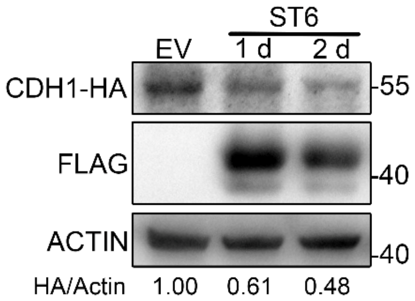

Figure S12. 293T cells stably expressing ectopic CDH1-HA under the CMV promoter were transfected with empty vehicle (EV) or SIRT6 overexpressing plasmid (ST6). CDH1-HA levels were detected using anti-HA antibody. Anti-FLAG antibody was used to detect the expression of SIRT6-FLAG, and actin was used as a loading control.

Supplementary Tables

Table S1. Candidate interacting proteins identified in 293T SIRT6-FLAG AP-MS experiment

Accession	Gene name	ΣCoverage	Score ST6	Coverage ST6	Unique peptide	# AAs	MW [kDa]	SAINT score
C9J4Z3	RPL37A	44.12	61.24	44.12	2	68	7.62	1
O15027-5	SEC16A	34.39	284.67	33.71	2	2201	235.61	1
O60814	HIST1H2BK	26.98	42.29	26.98	2	126	13.88	0.98
P04908	HIST1H2AB	36.15	74.44	36.15	2	130	14.13	1
P62266	RPS23	37.06	149.23	37.06	2	143	15.80	1
Q5T1V6-2	DDX59	7.24	399.47	7.24	2	580	64.53	1
Q8WWM7	ATXN2L	32.19	343.31	32.19	2	1075	113.30	1
Q96KK5	HIST1H2AH	36.72	142.84	36.72	2	128	13.90	1
Q96RR5	HCA90	51.47	345.61	51.47	2	783	89.34	1
Q9NYF8-2	BCLAF1	25.82	75.99	14.16	2	918	105.88	1
Q9UN86-2	G3BP2	49.89	372.43	49.89	2	449	50.79	1
P63167	DYNLL1	65.17	48.77	65.17	3	89	10.36	0.99
Q3MHD2	LSM12	26.15	40.89	26.15	3	195	21.69	1
Q8IUE6	HIST2H2AB	35.38	51.75	35.38	3	130	13.99	1
A0A075B7F8	POM121C	24.98	276.74	24.98	4	1229	125.02	1
E9PQ01	PCF11	44.94	553.58	44.94	4	999	112.24	1
O94808	GFPT2	15.98	40.84	15.98	4	682	76.88	1
P30154-4	PPP2R1B	26.08	60.05	26.08	4	556	61.27	1
J3KNL6	SEC16A	34.92	306.75	34.28	5	2357	251.74	1
P36954	POLR2I	56.8	60.29	56.8	5	125	14.51	1
P49790	NUP153	57.76	1167.52	57.76	5	1475	153.84	1
P67870	CSNK2B	26.98	72.31	26.98	5	215	24.93	1
Q9Y221	NIP7	51.11	54.35	51.11	5	180	20.45	1
E9PCY5	TOP2B	14.43	68.12	14.43	6	1150	130.40	1
O14497-2	ARID1A	6	41.94	4.74	6	2068	218.20	1
P35637-2	FUS	19.05	139.90	19.05	6	525	53.32	1
P52435	POLR2J	40.17	46.32	40.17	6	117	13.29	1
Q13838	DDX39B	36.21	61.14	32.94	6	428	48.96	1
Q8NFH5-2	NUP35	25.57	42.71	25.57	6	309	33.10	1
Q96HA1	POM121	24.34	290.32	24.34	6	1249	127.64	1
Q9BRR8	GPATCH1	13.1	63.73	13.1	6	931	103.28	1
Q9H2G4	TSPYL2	9.96	47.86	9.96	6	693	79.39	0.92
D6RIY6	EXOSC9	25.53	64.81	25.53	7	423	46.95	0.98
O15514	POLR2D	41.55	42.09	41.55	7	142	16.30	1
O43159	RRP8	21.49	53.26	21.49	7	456	50.68	1
P15927	RPA2	40.37	74.72	37.41	7	270	29.23	1
P27348	YWHAQ	43.27	40.87	43.27	7	245	27.75	1
P30291-2	WEE1	27.31	47.09	27.31	7	432	49.04	1

Table S1 continued

Accession	Gene name	ΣCoverage	Score ST6	Coverage ST6	Unique peptide	# AAs	MW [kDa]	SAINT score
P52294	KPNA1	34.76	98.89	34.57	7	538	60.18	1
P55795	HNRNPH2	40.98	199.61	40.98	7	449	49.23	0.99
Q16637-4	SMN1	33.2	58.39	33.2	7	250	27.12	1
Q5MIZ7-2	SMEK2	23.13	83.53	23.13	7	817	93.66	1
Q5QJE6	DNTTIP2	14.81	70.74	14.81	7	756	84.42	1
Q96FX7	TRMT61A	41.87	42.70	41.87	7	289	31.36	1
Q9BUF5	TUBB6	48.88	219.44	39.24	7	446	49.83	1
Q9BYG3	NIFK	30.03	45.74	30.03	7	293	34.20	0.99
Q9NQG5	RPRD1B	47.24	134.43	47.24	7	326	36.88	1
F5GWD3	GTF2H3	41.86	49.08	41.86	8	258	28.89	1
J3KPF0	HECTD4	5.5	42.05	5.5	8	4272	468.36	1
M0R0F0	RPS5	31	266.18	31	8	200	22.38	1
O43818	RRP9	30.95	43.39	27.79	8	475	51.81	1
P07992	ERCC1	37.37	48.32	37.37	8	297	32.54	1
P55769	NHP2L1	51.56	52.20	51.56	8	128	14.16	0.98
P62487	POLR2G	40.7	45.83	40.7	8	172	19.28	1
Q13185	CBX3	50.82	56.28	50.82	8	183	20.80	1
Q14643-4	ITPR1	8.65	70.64	8.65	8	2695	306.58	1
Q15906	VPS72	40.93	53.50	38.74	8	364	40.57	1
Q6P1K8	GTF2H2C	33.16	41.04	30.89	8	395	44.42	1
Q6P2C8	MED27	32.8	44.31	32.8	8	311	35.41	1
Q8NFH3	NUP43	32.63	80.89	32.63	8	380	42.12	1
Q8NFH4	NUP37	33.74	66.39	33.74	8	326	36.68	1
Q9BYN8	MRPS26	22.44	47.38	22.44	8	205	24.20	1
Q9GZN1	ACTR6	34.85	42.01	34.85	8	396	45.78	1
Q9NY61	AATF	23.93	51.09	23.93	8	560	63.09	1
Q9ULX6-2	AKAP8L	21.54	82.96	21.54	8	585	65.19	1
E9PEI0	CDCA2	18.65	67.04	18.65	9	1008	110.94	1
O00139-2	KIF2A	14.55	48.93	14.55	9	660	75.00	1
O60684	KPNA6	45.34	75.13	45.15	9	536	59.99	1
P31943	HNRNPH1	48.11	1000.28	48.11	9	449	49.20	1
P37108	SRP14	41.91	60.50	41.91	9	136	14.56	0.99
Q5T5Y3	CAMSAP1	11.17	43.40	11.17	9	1602	177.86	1
Q86WP2-3	GPBP1	34.41	72.20	34.41	9	465	52.41	1
Q8TF76	GSG2	20.3	45.00	20.3	9	798	88.44	1
Q96CT7	CCDC124	34.98	87.23	34.98	9	223	25.82	1
Q96P16	RPRD1A	50	146.83	50	9	312	35.70	1
Q9HB71-3	CACYBP	60	95.17	60	9	185	21.21	1
Q9NPI1	BRD7	20.89	51.64	20.89	9	651	74.09	1
Q9NQT5	EXOSC3	48	42.26	48	9	275	29.55	1

Table S1 continued

Accession	Gene name	ΣCoverage	Score ST6	Coverage ST6	Unique peptide	# AAs	MW [kDa]	SAINT score
Q9NW75	GPATCH2	24.43	52.67	24.43	9	528	58.91	1
Q9UNF1	MAGED2	20.3	45.43	16.34	9	606	64.91	1
A0A075B6T1	AMBRA1	16.28	47.45	16.28	10	1179	129.52	1
A0A087X1A5	STAU1	29.01	50.23	29.01	10	493	54.91	1
G3V203	RPL18	44.51	181.71	44.51	10	164	18.74	1
G5E9A6	USP11	18.48	41.68	18.48	10	920	104.96	1
J3KMX2	SMARCD2	32.68	73.24	32.68	10	456	52.21	1
J3KNZ9	FBRS	17.65	46.10	17.65	10	980	103.71	1
O96019-2	ACTL6A	43.41	103.33	43.41	10	387	43.21	1
P30050	RPL12	66.06	283.54	66.06	10	165	17.81	1
P78362	SRPK2	26.16	56.58	26.16	10	688	77.48	1
Q14498-2	RBM39	23.66	72.94	23.66	10	524	58.62	1
Q15050	RRS1	38.08	78.27	38.08	10	365	41.17	1
Q71RC2-3	LARP4	19.92	71.14	19.92	10	723	80.42	1
Q8IWU2	LMTK2	13.77	43.73	13.77	10	1503	164.80	1
Q8TAQ2-2	SMARCC2	20.62	154.39	19.91	10	1130	124.76	1
Q8WXX5	DNAJC9	45.38	66.42	45.38	10	260	29.89	0.92
Q92615	LARP4B	24.53	61.37	24.53	10	738	80.50	1
Q96DT7	ZBTB10	17.22	43.78	17.22	10	871	94.83	1
Q96PV6-2	LENG8	18.88	45.67	18.88	10	800	88.10	1
Q96SK2-2	TMEM209	36.96	59.84	36.96	10	560	62.75	1
Q9H0S4	DDX47	27.03	62.60	22.42	10	455	50.61	1
Q9H0W8	SMG9	25.77	54.62	25.77	10	520	57.61	1
Q9NWT1	PAK1IP1	25.77	47.40	23.47	10	392	43.94	1
Q9NZJ5	EIF2AK3	15.5	43.60	14.52	10	1116	125.14	0.98
Q9UJX4	ANAPC5	20.79	45.77	20.79	10	755	85.02	1
B4DGU4	CTNNB1	24.94	73.36	24.94	11	774	84.71	1
F8WAJ0	DDX31	21.85	55.92	21.85	11	746	83.46	1
H0Y714	IMP4	60.71	133.28	60.71	11	280	31.93	1
O14641	DVL2	25.82	85.35	25.82	11	736	78.90	1
O43422	PRKRIR	23.78	41.33	23.78	11	761	87.65	1
P05387	RPLP2	82.61	492.18	82.61	11	115	11.66	1
P49750-3	YLPM1	9.94	53.17	9.94	11	1862	209.35	1
P62805	HIST1H4A	61.17	120.30	61.17	11	103	11.36	1
P62917	RPL8	51.75	218.16	51.75	11	257	28.01	1
Q13042	CDC16	22.58	47.11	20.97	11	620	71.61	1
Q15024	EXOSC7	52.23	106.13	52.23	11	291	31.80	1
Q15047-3	SETDB1	12.33	44.94	12.33	11	1290	142.91	1
Q4VCS5	AMOT	16.88	67.07	15.96	11	1084	118.01	1
Q6WCQ1-3	MPRIP	16.9	46.34	15.8	11	1000	113.98	1

Table S1 continued

Accession	Gene name	ΣCoverage	Score ST6	Coverage ST6	Unique peptide	# AAs	MW [kDa]	SAINT score
Q8N1G0	ZNF687	17.46	51.05	17.46	11	1237	129.45	1
Q8TDN6	BRIX1	48.44	106.22	48.44	11	353	41.37	0.99
Q92759	GTF2H4	33.55	49.67	30.74	11	462	52.15	1
Q96EE3	SEH1L	38.89	66.83	38.89	11	360	39.62	1
Q96GM5	SMARCD1	31.07	46.65	27.18	11	515	58.20	1
Q9NQ55-2	PPAN	37.83	49.80	29.78	11	460	51.87	1
Q9NV31	IMP3	72.28	134.90	72.28	11	184	21.84	1
Q9P2K5-2	MYEF2	18.92	45.59	18.92	11	576	61.89	1
Q9Y265	RUVBL1	34.65	100.65	34.65	11	456	50.20	1
Q9Y2I1	NISCH	13.56	42.65	13.56	11	1504	166.52	1
Q9Y2X0-2	MED16	16.05	40.53	15.34	11	841	92.97	1
Q9Y3T9	NOC2L	17.09	42.24	17.09	11	749	84.87	1
H3BP71	RNF40	21.23	71.34	21.23	12	961	108.98	1
O15047	SETD1A	13.65	49.78	13.65	12	1707	185.92	1
O43823	AKAP8	20.23	83.81	20.23	12	692	76.06	1
O60347	TBC1D12	22.97	47.88	22.97	12	775	85.57	1
O60832	DKC1	33.07	62.96	27.82	12	514	57.64	0.98
O60942-4	RNGTT	28.47	57.10	28.47	12	541	61.67	1
P08621	SNRNP70	28.15	122.60	24.49	12	437	51.53	1
P29590-11	PML	26.02	56.85	26.02	12	834	92.51	1
P32969	RPL9	66.15	300.08	66.15	12	192	21.85	1
P46100-3	ATRX	7.28	57.43	7.28	12	2375	269.64	1
P62906	RPL10A	47.93	162.16	47.93	12	217	24.82	1
P78346	RPP30	51.12	64.38	51.12	12	268	29.30	1
P82979	SARNP	60.48	111.69	60.48	12	210	23.66	1
Q07021	C1QBP	52.48	356.36	52.48	12	282	31.34	1
Q13428-2	TCOF1	12.97	73.38	12.97	12	1411	144.23	1
Q15397	KIAA0020	23.46	64.96	23.46	12	648	73.54	1
Q5T6F2	UBAP2	27.97	306.21	27.97	12	1119	117.04	1
Q6UN15	FIP1L1	33.5	98.75	31.14	12	594	66.49	1
Q9GZS3	WDR61	55.74	99.76	55.74	12	305	33.56	1
Q9H089	LSG1	29.18	64.00	29.18	12	658	75.18	1
Q9H7U1-3	CCSER2	21.11	51.40	21.11	12	1047	116.84	1
Q9H9Y6	POLR1B	13.66	61.95	13.66	12	1135	128.15	1
Q9NY93-2	DDX56	28.99	49.26	26.63	12	507	57.24	1
Q9UBP6	METTL1	50.72	194.06	50.72	12	276	31.45	1
Q9UG63	ABCF2	20.55	51.53	20.55	12	623	71.24	0.98
Q9ULW3	ABT1	57.35	72.41	57.35	12	272	31.06	1
Q9UPS6-2	SETD1B	11.91	63.44	11.91	12	1923	208.60	1
Q9Y295	DRG1	45.78	55.20	45.78	12	367	40.52	0.86

Table S1 continued

Accession	Gene name	ΣCoverage	Score ST6	Coverage ST6	Unique peptide	# AAs	MW [kDa]	SAINT score
Q9Y2X7	GIT1	29.57	55.56	29.57	12	761	84.29	1
Q9Y450-4	HBS1L	29.91	82.20	27.88	12	642	70.58	1
A0A087WXM6	RPL17	53.25	282.96	53.25	13	169	19.57	1
E7EXA6	CHTF18	20.31	42.35	20.31	13	975	107.36	1
F5H8F7	ASH2L	38.04	71.34	38.04	13	489	55.29	1
O15381	NVL	22.43	49.10	22.43	13	856	94.99	1
P26196	DDX6	40.17	77.69	40.17	13	483	54.38	1
P37198	NUP62	23.95	96.03	23.95	13	522	53.22	1
P40938	RFC3	44.66	55.58	34.55	13	356	40.53	1
P62750	RPL23A	47.44	105.62	46.79	13	156	17.68	1
P78406	RAE1	55.16	101.00	52.72	13	368	40.94	1
Q13610	PWP1	38.12	90.07	38.12	13	501	55.79	1
Q15291	RBBP5	31.97	54.79	31.97	13	538	59.12	1
Q86YS7	C2CD5	17.8	59.83	17.8	13	1000	110.38	1
Q92610	ZNF592	15.47	86.56	15.47	13	1267	137.44	1
Q92989	CLP1	29.65	106.55	29.65	13	425	47.62	1
Q9BQ39	DDX50	30.12	82.85	27.14	13	737	82.51	0.95
Q9H5Z1-2	DHX35	29.76	60.96	28.42	13	672	75.39	1
Q9UJX5	ANAPC4	22.15	48.55	21.16	13	808	92.06	1
B7Z6D5	DDX27	18.69	43.95	17.65	14	765	86.55	1
H0YM23	ANKRD17	17.29	88.74	16.53	14	2487	263.20	1
H7BXH2	PPP6R3	24.06	61.47	24.06	14	827	92.35	1
O43663-4	PRC1	33	78.38	28.55	14	606	70.15	1
O60341	KDM1A	25	71.66	25	14	852	92.84	1
O60568	PLOD3	28.46	126.65	28.46	14	738	84.73	1
P19387	POLR2C	62.55	135.71	62.55	14	275	31.42	1
P60510	PPP4C	68.08	181.92	68.08	14	307	35.06	1
Q06787-8	FMR1	30.03	109.72	30.03	14	586	66.21	1
Q14137-2	BOP1	41.17	91.14	41.17	14	634	71.89	1
Q5JRA6-2	MIA3	11.15	41.68	9.74	14	1848	206.76	1
Q5SW79	CEP170	14.14	62.32	14.14	14	1584	175.19	1
Q8IXW5-2	RPAP2	31.51	62.13	31.51	14	584	66.43	1
Q8IZL8	PELP1	23.19	110.66	23.19	14	1130	119.62	1
Q8WTT2	NOC3L	23.88	83.59	23.88	14	800	92.49	1
Q96S38	RPS6KC1	23.73	57.11	23.73	14	1066	118.61	1
Q96S55-2	WRNIP1	24.38	52.31	24.38	14	640	69.42	1
Q9H6R4-4	NOL6	21.96	53.42	21.26	14	1143	127.24	1
Q9NVP1	DDX18	37.16	88.97	37.16	14	670	75.36	1
Q9Y5B9	SUPT16H	19.1	53.02	18.15	14	1047	119.84	0.99
A0A087WYU1	SNX9	50.84	129.18	50.84	15	594	66.43	1

Table S1 continued

Accession	Gene name	ΣCoverage	Score ST6	Coverage ST6	Unique peptide	# AAs	MW [kDa]	SAINT score
B4DXZ6	FXR1	45.72	127.76	45.72	15	608	68.29	1
O00469	PLOD2	28.36	64.56	28.36	15	737	84.63	1
O95625	ZBTB11	17.76	85.82	17.76	15	1053	119.31	1
P05455	SSB	42.89	76.69	42.89	15	408	46.81	1
P06748-2	NPM1	53.58	1039.59	53.58	15	265	29.45	1
P52597	HNRNPF	63.13	770.45	63.13	15	415	45.64	1
P57081	WDR4	46.12	176.52	46.12	15	412	45.46	1
Q03701	CEBPZ	19.17	92.02	19.17	15	1054	120.90	1
Q15269	PWP2	24.48	83.42	24.48	15	919	102.39	1
Q15642-2	TRIP10	38.35	239.05	38.35	15	545	62.55	1
Q15648	MED1	14.23	65.57	14.23	15	1581	168.37	1
Q7Z2W4	ZC3HAV1	25.94	73.47	25.94	15	902	101.37	1
Q8IXB1	DNAJC10	24.34	54.75	22.82	15	793	91.02	1
Q8ND04	SMG8	24.72	112.31	24.72	15	991	109.61	1
Q96JC1-2	VPS39	24.46	47.17	24.46	15	875	100.70	1
Q9H078-2	CLPB	30.43	47.45	17.73	15	677	75.40	1
Q9NRG9	AAAS	44.87	89.18	44.87	15	546	59.54	1
Q9NVU7	SDAD1	22.27	73.67	22.27	15	687	79.82	1
Q9NY27	PPP4R2	45.08	123.46	45.08	15	417	46.87	1
Q9UBD5	ORC3	34.88	79.87	32.63	15	711	82.20	1
Q9UER7	DAXX	26.35	95.78	26.35	15	740	81.32	1
Q9Y2K1	ZBTB1	28.47	71.51	28.47	15	713	81.96	1
A0A087WV66	MKI67	8.66	44.71	7.99	16	3255	358.41	1
E9PFT7	APC	17.55	61.42	17.55	16	1305	145.67	1
G5EA36	CDC27	38.64	74.47	38.64	16	823	91.68	1
O60306	AQR	16.23	49.60	16.23	16	1485	171.19	1
O95602	POLR1A	15.7	64.03	13.9	16	1720	194.69	1
P06493	CDK1	73.06	163.26	73.06	16	297	34.07	1
P22087	FBL	57.94	163.93	57.94	16	321	33.76	1
P31350	RRM2	54.76	191.44	54.76	16	389	44.85	1
P51531-2	SMARCA2	20.8	151.65	19.21	16	1572	179.17	1
Q5VUA4	ZNF318	11.5	92.87	10.05	16	2279	250.96	1
Q68CP9-3	ARID2	13.4	85.32	13.4	16	1783	191.30	1
Q70EL1-7	USP54	14.8	66.24	14.8	16	1534	170.48	1
Q7LG56	RRM2B	62.68	172.14	62.68	16	351	40.71	1
Q99959-2	PKP2	32.5	99.40	32.5	16	837	92.70	1
Q9H6R7	C2orf44	38.56	88.95	38.56	16	721	79.09	1
Q9NVC6	MED17	32.26	52.14	30.57	16	651	72.84	1
Q9NW13-2	RBM28	29.45	85.73	29.45	16	618	69.88	1
Q9NXC5	MIOS	23.31	46.47	21.14	16	875	98.52	1

Table S1 continued								
Accession	Gene name	ΣCoverage	Score ST6	Coverage ST6	Unique peptide	# AAs	MW [kDa]	SAINT score
Q9P2N5	RBM27	23.3	152.18	21.79	16	1060	118.64	1
Q9UJX2	CDC23	30.99	54.35	28.48	16	597	68.79	1
Q9UKJ3	GPATCH8	14.05	78.85	14.05	16	1502	164.10	1
P15880	RPS2	49.49	423.47	49.49	17	293	31.30	1
P46777	RPL5	58.25	383.75	55.89	17	297	34.34	1
Q06210-2	GFPT1	41.7	153.66	39.79	17	681	76.71	1
Q13627-2	DYRK1A	22.28	79.60	22.28	17	754	84.50	1
Q14160	SCRIB	20.31	78.83	20.31	17	1630	174.78	1
Q14669-4	TRIP12	16.9	65.02	16.2	17	1722	191.95	0.98
Q92922	SMARCC1	32.49	250.36	31.76	17	1105	122.79	1
Q96RF0-2	SNX18	37.02	91.30	37.02	17	624	68.96	1
Q96T76	MMS19	28.54	72.08	27.38	17	1030	113.22	0.99
Q99575	POP1	25.2	129.44	25.2	17	1024	114.64	1
Q9BYW2	SETD2	10.96	49.70	10.96	17	2564	287.42	1
A6NNK5	TP53BP1	17.33	82.35	17.33	18	1927	208.89	1
H0YNU5	BLM	20.84	65.55	20.22	18	1286	144.38	1
P05388	RPLP0	59.62	789.70	59.62	18	317	34.25	1
P30414	NKTR	18.13	132.21	18.13	18	1462	165.58	1
P47897	QARS	32.13	58.53	27.61	18	775	87.74	1
P50454	SERPINH1	57.18	225.11	57.18	18	418	46.41	0.96
P51116	FXR2	42.2	117.09	41.01	18	673	74.18	1
P52292	KPNA2	61.06	212.18	61.06	18	529	57.83	1
Q08945	SSRP1	30.32	68.13	30.32	18	709	81.02	1
Q14157-4	UBAP2L	40.98	330.47	40.98	18	976	103.11	1
Q14573	ITPR3	12.84	74.52	12.84	18	2671	303.91	1
Q14674	ESPL1	15.19	62.50	15.19	18	2120	233.03	1
Q14C86-5	GAPVD1	20.45	74.55	20.45	18	1452	162.13	1
Q16204	CCDC6	36.08	128.71	36.08	18	474	53.26	1
Q5VTR2	RNF20	27.59	82.44	26.15	18	975	113.59	1
Q6PD62	CTR9	21.74	139.84	21.74	18	1173	133.42	1
Q8NB90	SPATA5	33.03	105.54	31.69	18	893	97.84	0.9
Q8TDD1	DDX54	32.35	92.88	32.35	18	881	98.53	1
Q92974-3	ARHGEF2	26.2	68.34	25.05	18	958	108.17	1
O95714	HERC2	5.79	51.60	5.79	19	4834	526.90	1
P19784	CSNK2A2	63.14	151.33	60.57	19	350	41.19	1
P21127-6	CDK11B	28.18	46.90	28.18	19	738	86.01	1
P39023	RPL3	44.91	520.15	44.91	19	403	46.08	1
Q5VYS8	ZCCHC6	20.27	46.12	18.19	19	1495	171.12	1
Q7Z478	DHX29	23.89	68.98	22.35	19	1369	155.14	1
Q8IY92	SLX4	19.63	113.92	19.63	19	1834	199.89	1

Table S1 continued

Accession	Gene name	ΣCoverage	Score ST6	Coverage ST6	Unique peptide	# AAs	MW [kDa]	SAINT score
Q8N3X1	FNBP4	26.06	124.99	26.06	19	1017	110.20	1
Q8NHQ9	DDX55	35	78.97	35	19	600	68.50	1
Q96L91-3	EP400	11.63	67.51	11.08	19	3086	335.60	1
Q9H1I8	ASCC2	29.85	167.39	29.85	19	757	86.31	1
Q9NNW5	WDR6	28.28	121.49	28.28	19	1121	121.65	1
Q9NPF5	DMAP1	46.9	117.19	45.18	19	467	52.96	1
P52701	MSH6	19.85	73.72	19.85	20	1360	152.69	1
P61247	RPS3A	57.95	884.04	57.95	20	264	29.93	1
Q02241	KIF23	29.17	108.63	27.4	20	960	109.99	1
Q14247	CTTN	38.36	227.15	36.91	20	550	61.55	1
Q14684	RRP1B	35.49	108.76	35.49	20	758	84.38	1
Q5VUJ6-2	LRCH2	37.17	136.11	37.03	20	748	82.76	1
Q99567	NUP88	41.7	142.81	41.7	20	741	83.49	1
Q99832	CCT7	47.88	119.83	41.44	20	543	59.33	1
Q9H0W5	CCDC8	50.56	189.96	50.56	20	538	59.34	1
Q9H501	ESF1	26.79	134.42	26.79	20	851	98.73	1
Q9HCE1	MOV10	25.42	86.71	24.53	20	1003	113.60	1
Q9NQZ2	UTP3	44.05	326.26	44.05	20	479	54.53	1
Q9Y2U8	LEMD3	31.94	112.13	31.94	20	911	99.94	1
O00116	AGPS	50.76	310.77	49.54	21	658	72.87	1
O14654	IRS4	25.86	126.55	22.04	21	1257	133.68	1
O43395	PRPF3	33.38	151.70	33.38	21	683	77.48	1
O94913	PCF11	50.87	1197.24	50.87	21	1555	172.94	1
O95831	AIFM1	46	88.12	38.99	21	613	66.86	1
P19447	ERCC3	40.79	120.12	40.79	21	782	89.22	1
Q15020	SART3	31.36	184.57	31.36	21	963	109.87	1
Q1KMD3	HNRNPUL2	31.06	79.27	26.24	21	747	85.05	1
Q5VT52-3	RPRD2	28.43	107.12	26.06	21	1435	152.78	1
Q92889	ERCC4	36.03	174.29	36.03	21	916	104.42	1
Q9BUJ2-2	HNRNPUL1	35.7	206.76	34.7	21	804	90.24	1
Q9BW27	NUP85	42.38	140.01	40.4	21	656	74.97	1
Q9H6S0	YTHDC2	19.65	88.53	18.74	21	1430	160.15	1
Q9ULK4-4	MED23	22.22	89.44	22.22	21	1359	155.45	0.95
Q9UPY3-2	DICER1	18.43	80.86	17	21	1829	208.32	1
Q9Y4W2	LAS1L	45.5	118.42	45.5	21	734	83.01	1
O60244	MED14	24.42	137.74	24.42	22	1454	160.50	1
P17480-2	UBTF	26.13	183.75	26.13	22	727	84.88	1
P51532-5	SMARCA4	24.18	195.46	22.63	22	1613	181.15	1
P55265-5	ADAR	33.3	112.91	33.3	22	931	103.58	0.94
Q14444-2	CAPRIN1	60.52	821.80	60.52	22	694	76.81	1

Table S1 continued

Accession	Gene name	ΣCoverage	Score ST6	Coverage ST6	Unique peptide	# AAs	MW [kDa]	SAINT score
Q14566	MCM6	33.01	149.40	33.01	22	821	92.83	1
Q6R327	RICTOR	18.79	83.34	18.21	22	1708	192.10	1
Q7Z417	NUFIP2	46.33	375.91	46.33	22	695	76.07	1
Q96II8	LRCH3	46.85	199.90	46.85	22	777	86.03	1
Q9BVQ7	SPATA5L1	51.13	70.48	51.13	22	753	80.66	1
Q9BZE4	GTPBP4	35.02	139.79	35.02	22	634	73.92	1
Q9H2P0	ADNP	29.49	190.79	29.49	22	1102	123.49	1
Q9NXF1-2	TEX10	31.33	139.72	31.33	22	913	103.85	1
O95235	KIF20A	37.64	117.10	37.64	23	890	100.22	1
O95248	SBF1	19.71	103.18	19.71	23	1867	208.18	1
O95757	HSPA4L	41.24	151.11	41.24	23	839	94.45	1
P46087-2	NOP2	37.25	209.57	37.25	23	808	88.92	1
Q08J23	NSUN2	42.76	144.86	39.63	23	767	86.42	1
Q8N6T7-2	SIRT6	81.4	25027.22	81.4	23	328	36.04	1
Q8NI36	WDR36	36.8	132.27	36.8	23	951	105.26	1
Q9H1A4	ANAPC1	18.78	102.60	18.78	23	1944	216.36	1
Q9P2E9	RRBP1	21.7	41.92	8.58	23	1410	152.38	1
O00267-2	SUPT5H	28.99	195.25	28.99	24	1083	120.42	1
O60293	ZFC3H1	17.9	107.63	17.14	24	1989	226.21	1
P33993	MCM7	46.59	234.84	46.59	24	719	81.26	1
Q02809	PLOD1	41.82	238.54	41.82	24	727	83.50	1
Q13823	GNL2	31.19	98.79	30.1	24	731	83.60	1
Q15650	TRIP4	51.12	110.04	48.54	24	581	66.10	1
Q92576-2	PHF3	19.02	136.40	19.02	24	1951	219.67	1
O00566	MPHOSPH10	35.39	410.22	35.39	25	681	78.82	1
O94964-2	SOGA1	23.12	94.52	23.12	25	1661	183.74	1
P02545-3	LMNA	45.27	51.42	26.66	25	634	70.62	1
P33991	MCM4	36.96	133.95	36.96	25	863	96.50	1
Q12788	TBL3	45.92	167.20	44.55	25	808	88.98	1
Q5SRE5	NUP188	19.61	114.23	18.75	25	1749	195.92	1
P52272-2	HNRNPM	54.7	525.81	54.7	26	691	73.57	1
Q13263	TRIM28	53.05	357.24	53.05	26	835	88.49	1
Q5JSL3	DOCK11	19.68	104.68	19.68	26	2073	237.52	1
Q7Z5K2	WAPAL	32.94	190.74	32.94	26	1190	132.86	1
Q8IY81	FTSJ3	41.79	246.98	41.79	26	847	96.50	1
Q9HCK8	CHD8	18.25	99.01	18.25	26	2581	290.34	1
Q9UBU9	NXF1	53.47	221.63	53.47	26	619	70.14	1
O60264	SMARCA5	24.81	137.17	24.81	27	1052	121.83	1
O60716-13	CTNND1	43.57	186.97	43.57	27	879	97.99	1
P49736	MCM2	40.6	290.26	40.6	27	904	101.83	1

Table S1 continued

Accession	Gene name	ΣCoverage	Score ST6	Coverage ST6	Unique peptide	# AAs	MW [kDa]	SAINT score
Q13283	G3BP1	60.3	5199.88	60.3	27	466	52.13	1
Q7Z2T5	TRMT1L	58.66	167.73	56.62	27	733	81.70	1
Q8N1F7	NUP93	45.18	181.11	45.18	27	819	93.43	1
Q8NFD5-4	ARID1B	23.76	151.03	23.76	27	1486	162.37	1
Q9BXP5-4	SRRT	36.17	181.51	36.17	27	871	100.09	1
Q9UNX4	WDR3	44.86	169.81	44.86	27	943	106.03	1
O75694-2	NUP155	30.18	139.92	30.18	28	1332	148.92	1
O76021	RSL1D1	43.27	216.44	43.27	28	490	54.94	1
O95163	IKBKAP	31.08	106.73	31.08	28	1332	150.16	1
P33992	MCM5	47.28	239.42	47.28	28	734	82.23	1
Q14258	TRIM25	46.35	188.73	46.35	28	630	70.93	1
Q5JTH9	RRP12	31.61	119.28	29.45	28	1297	143.61	1
Q8NE71-2	ABCF1	48.08	232.14	46.72	28	807	91.62	1
J3KNB8	MAP3K4	26.32	116.12	26.32	29	1554	176.46	1
P34932	HSPA4	44.29	237.14	44.29	29	840	94.27	1
O15226	NKRF	44.2	215.52	42.46	30	690	77.62	0.99
P42285	SKIV2L2	37.72	175.40	36.66	30	1042	117.73	1
P78347-2	GTF2I	40.54	306.08	38.14	30	957	107.90	1
Q6IN85-2	SMEK1	53.29	377.08	53.29	30	820	93.84	1
Q8WUA4	GTF3C2	45.77	449.77	45.77	30	911	100.62	1
F5GWX5	CHD4	30.13	246.01	29.71	31	1905	216.97	1
Q8TEM1	NUP210	24.38	202.75	24.01	32	1887	204.98	1
Q92900-2	UPF1	38.01	210.12	37.03	32	1118	122.96	1
Q9ULT8	HECTD1	20.5	132.01	20.04	32	2610	289.20	1
P28288	ABCD3	48.86	309.74	48.86	33	659	75.43	1
Q12906	ILF3	55.93	440.92	55.15	33	894	95.28	1
Q14571	ITPR2	18.92	161.93	18.92	33	2701	307.87	1
Q6PKG0	LARP1	45.8	286.89	45.8	33	1096	123.43	1
P19338	NCL	38.03	2023.87	38.03	34	710	76.57	1
P57740	NUP107	51.78	241.55	51.78	34	925	106.31	1
Q14694	USP10	60.15	1102.20	60.15	35	798	87.08	1
O00411	POLRMT	46.99	249.96	46.99	36	1230	138.53	1
O94906	PRPF6	44.53	167.21	37.09	36	941	106.86	1
Q9BSJ8	ESYT1	45.11	423.45	45.11	36	1104	122.78	1
Q9Y5Q9	GTF3C3	48.76	483.78	46.84	36	886	101.21	1
O00571	DDX3X	59.37	714.31	59.37	38	662	73.20	1
P13010	XRCC5	69.26	358.12	69.26	38	732	82.65	1
Q562F6-2	SGOL2	42.19	315.24	42.19	38	1261	144.09	1
Q9H0D6	XRN2	54.53	298.67	54.53	38	950	108.51	1
P21359	NF1	18.53	152.48	18.53	39	2839	319.17	1

Table S1 continued

Accession	Gene name	ΣCoverage	Score ST6	Coverage ST6	Unique peptide	# AAs	MW [kDa]	SAINT score
Q01780	EXOSC10	54.12	242.49	52.66	39	885	100.77	1
Q9H0A0	NAT10	49.07	318.78	49.07	39	1025	115.66	1
Q9Y4E8	USP15	47.81	159.75	36.7	39	981	112.35	1
P25205	MCM3	56.68	442.45	56.68	40	808	90.92	1
P35658-2	NUP214	32.61	273.25	32.61	40	2079	212.44	1
P98175-2	RBM10	49.62	116.22	25.51	40	929	103.37	1
Q00610-2	CLTC	33.86	173.92	27.15	40	1639	187.77	1
Q12769	NUP160	37.74	302.74	37.74	40	1436	162.02	1
P49792	RANBP2	30.99	320.07	29.65	41	3224	357.97	1
P11388	TOP2A	40.24	320.08	40.24	42	1531	174.28	1
Q9Y4A5-2	TRRAP	16.01	152.73	14.65	42	3830	434.13	1
P78332	RBM6	42.83	407.17	42.83	43	1123	128.57	1
Q7Z406-6	MYH14	32.5	232.69	30.9	43	2003	228.53	1
C9J2Y9	POLR2B	45.76	362.71	45.76	44	1167	132.97	1
P52948-5	NUP98	36.28	345.39	36.28	44	1800	195.70	1
Q14980-2	NUMA1	30.03	248.08	30.03	44	2101	236.37	1
Q68CQ4	DIEXF	63.23	729.59	63.23	44	756	87.00	1
P09874	PARP1	54.64	500.74	54.64	45	1014	113.01	1
Q8TEQ6	GEMIN5	43.63	284.79	43.63	45	1508	168.48	1
Q9BQG0	MYBBP1A	43.6	387.74	41.87	45	1328	148.76	1
Q7KZ85	SUPT6H	35.05	235.54	35.05	46	1726	198.95	1
Q7KZF4	SND1	62.64	539.17	61.65	46	910	101.93	0.97
Q6ZRS2-2	SRCAP	21.84	317.39	21.84	47	3168	337.24	1
Q86U86-4	PBRM1	35.71	264.23	35.71	48	1582	180.97	1
Q8WUM0	NUP133	51.82	306.84	50.43	48	1156	128.90	1
Q14676	MDC1	42.89	511.61	42.89	50	2089	226.53	1
Q92621	NUP205	32.7	261.10	32.31	50	2012	227.78	1
Q96Q15	SMG1	23.41	230.04	23.11	52	3661	410.24	1
Q9UKN8	GTF3C4	78.22	542.56	78.22	52	822	91.92	1
Q08211	DHX9	55.12	789.48	55.12	53	1270	140.87	1
Q7L2E3-3	DHX30	58.61	481.86	58.61	53	1155	129.36	1
Q92620	DHX38	50.86	1168.85	50.86	53	1227	140.42	1
P24928	POLR2A	35.63	483.54	35.63	58	1970	217.04	1
O95071-2	UBR5	32.77	505.53	32.49	62	2798	309.03	1
Q93008-1	USP9X	33.44	310.54	32.77	63	2554	290.28	1
Q13085	ACACA	35.46	380.49	35.46	65	2346	265.38	1
O75592-2	MYCBP2	20.85	291.98	20.85	66	4637	509.38	1
F8VPD4	CAD	47.04	507.79	47.04	67	2162	235.87	1
Q7Z6Z7-3	HUWE1	25.57	357.26	25.43	67	4365	480.72	1
Q8N3C0	ASCC3	42.64	397.41	42.64	67	2202	251.30	1

Table S1 continued

Accession	Gene name	ΣCoverage	Score ST6	Coverage ST6	Unique peptide	# AAs	MW [kDa]	SAINT score
Q9BQS8	FYCO1	58.19	620.39	57.58	67	1478	166.88	1
Q12789	GTF3C1	49.69	952.54	49.69	83	2109	238.72	1

Table S2. DNA and RNA oligo sequences

siRNA sequences	
Gene	Sequence
ANAPC1 human-1 sense	CCAGAGAACCUUUACCUACUTT
ANAPC1 human-1 antisense	AGUAGGUAAAGGUUCUCUGGTT
ANAPC1 human-2 sense	GCUCCAGUUACGCUGUGUAATT
ANAPC1 human-2 sense	UUACACAGCGUAACUGGAGCTT
sgRNA targetting sequence	
Target	Sequence
SIRT6 human-1	GTACGTCCGAGACACAGTCG
SIRT6 human-2	CTTCCACAAACATGTTCCCG
Primes for real-time PCR	
Primer	Sequence
actin-qF60.8	CATGTACGTTGCTATCCAGGC
actin-qR60.2	CTCCTTAATGTCACGCACGAT
FZR1-qF62.7	GACCGCTGTATCCGCTTCTG
FZR1-qR62.9	GGTCAGGGAGGGGTACTTCC

Original images

Original images of western blots of pH3@S10 and Cyc B1 in figure 1b 293T

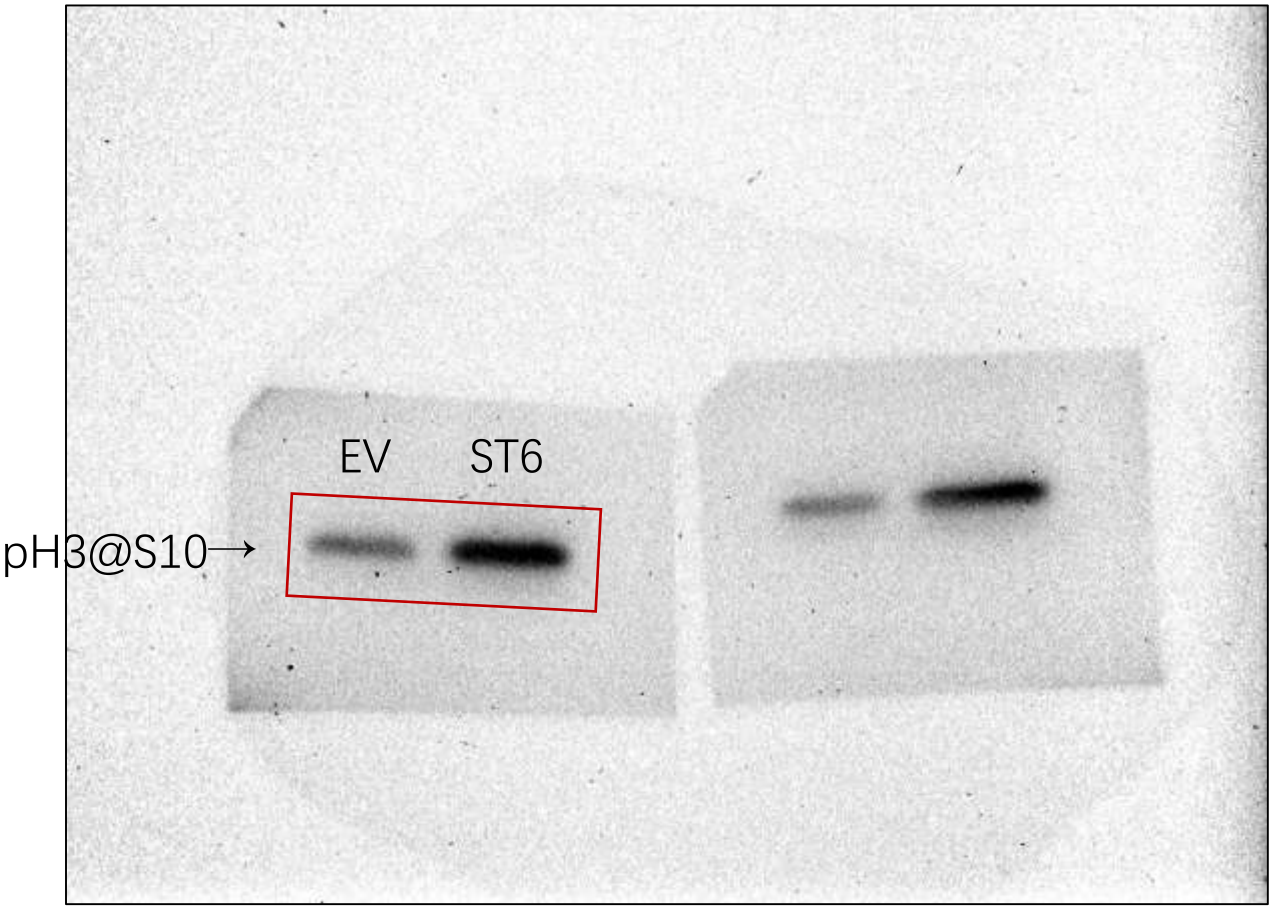

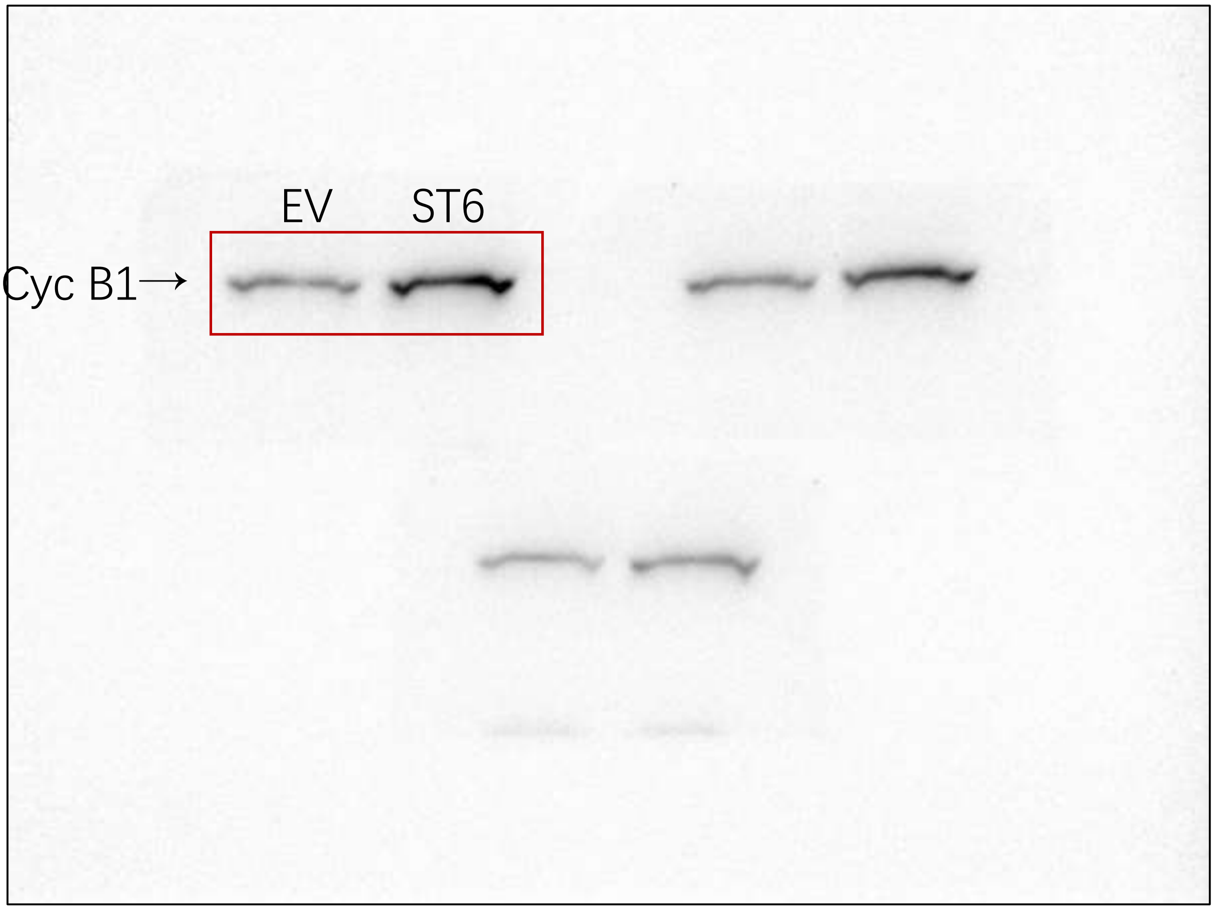

Original images of western blots of SIRT6-FLAG and HSP70 in figure 1b 293T

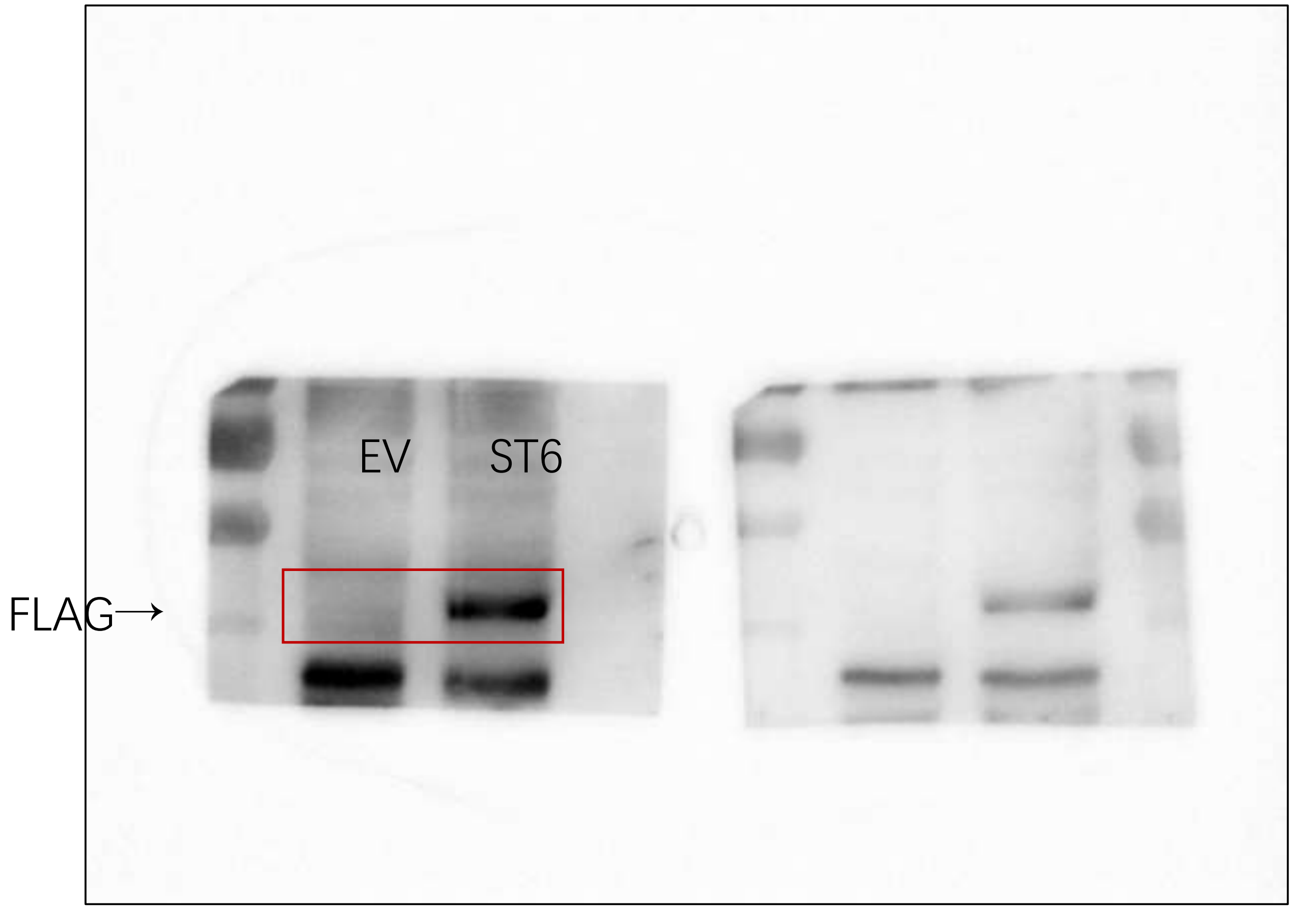

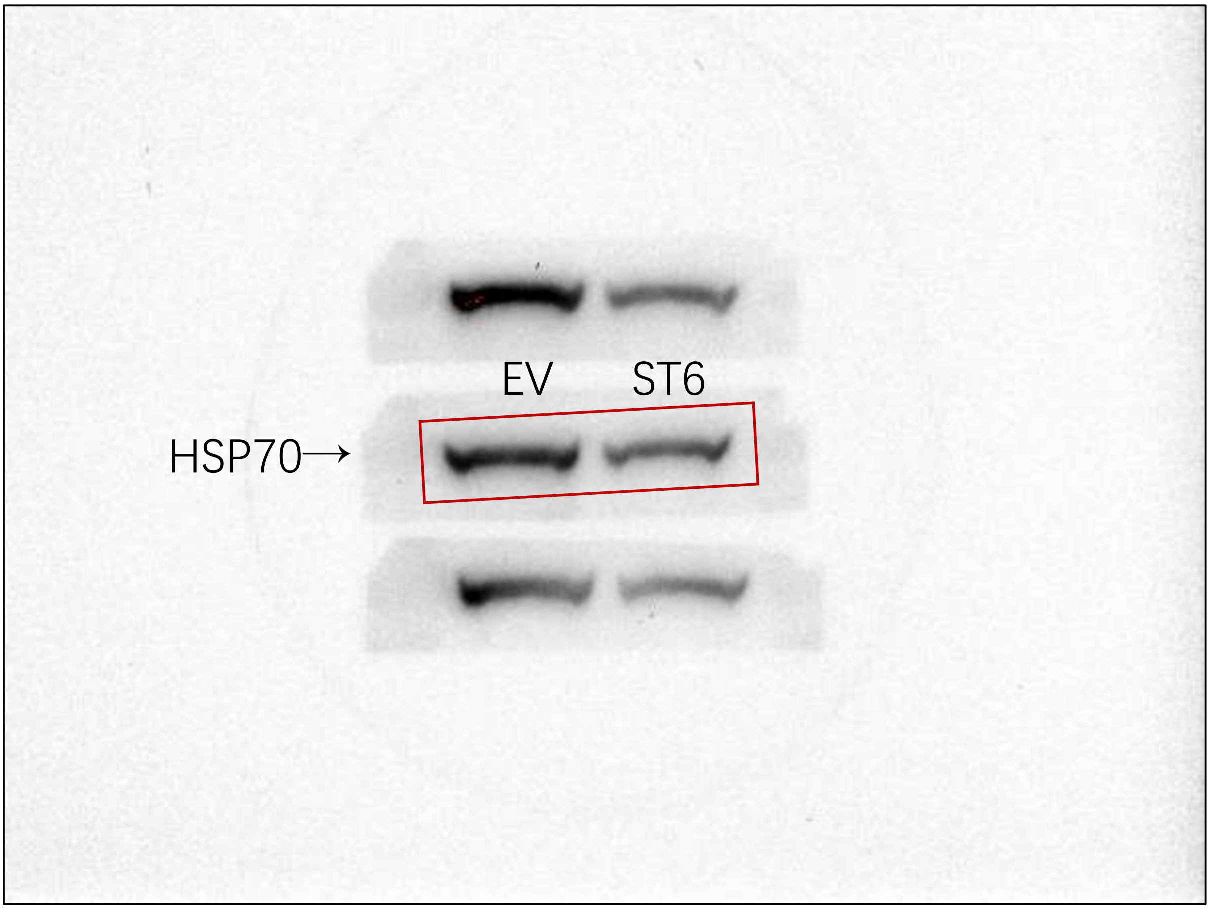

Original images of western blots of pH3@S10 and Cyc B1 in figure 1b HeLa

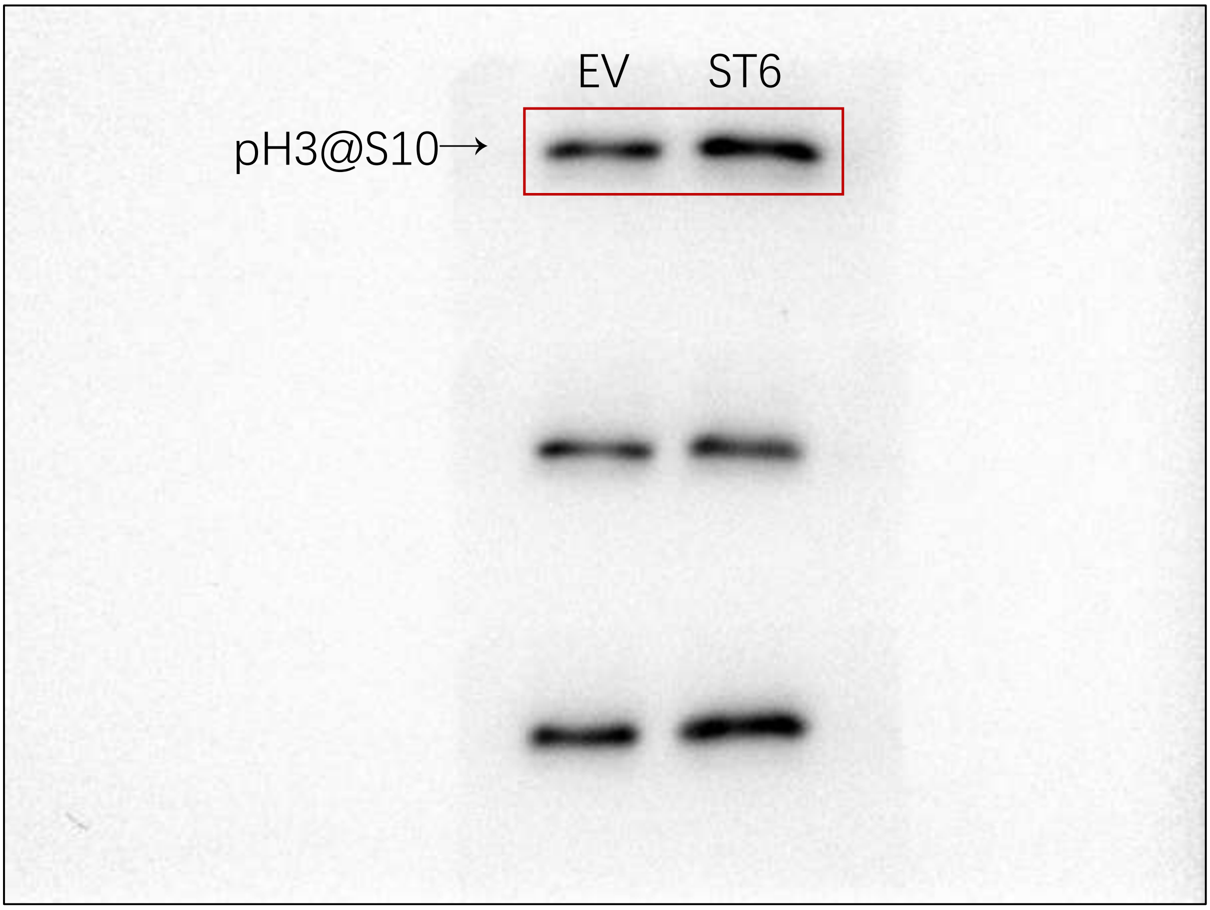

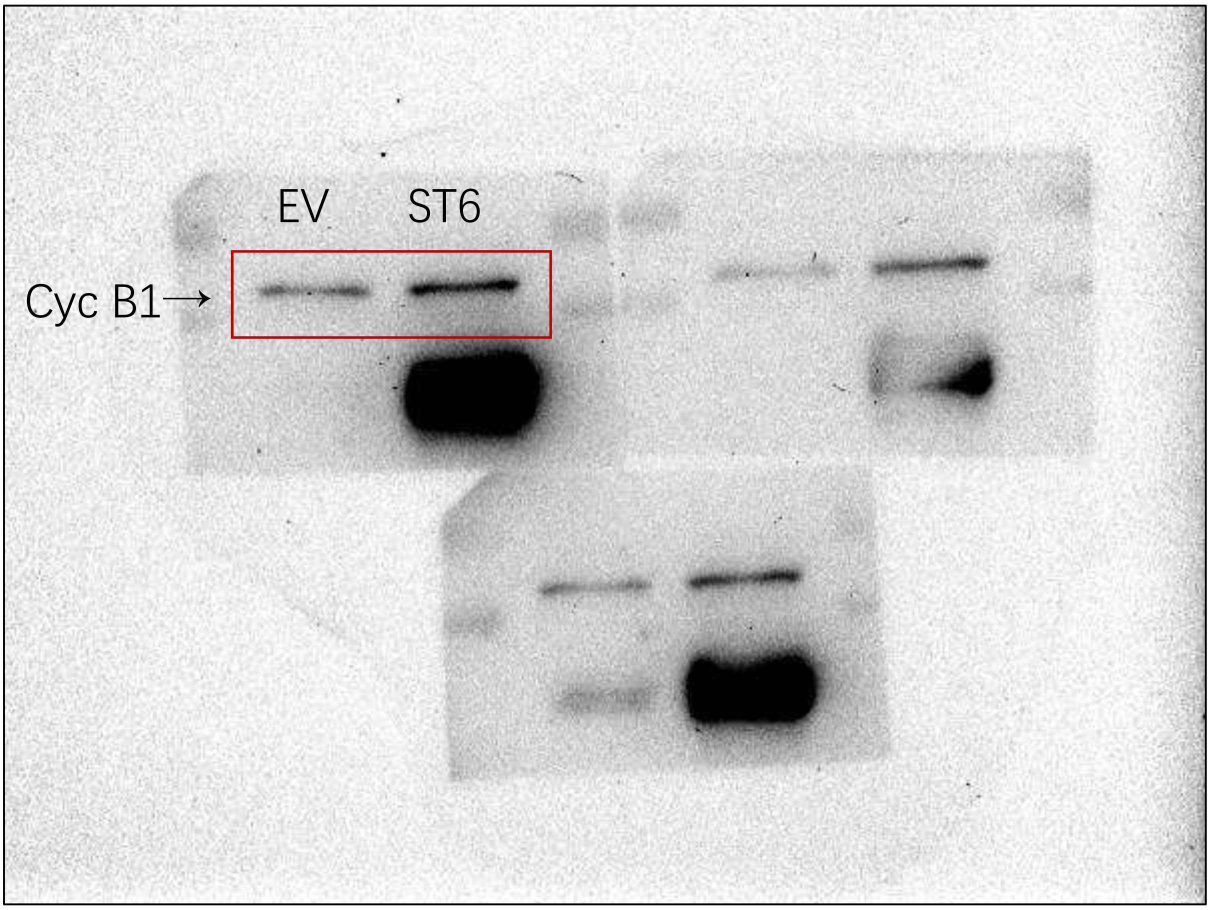

Original images of western blots of SIRT6-FLAG and HSP70 in figure 1b HeLa

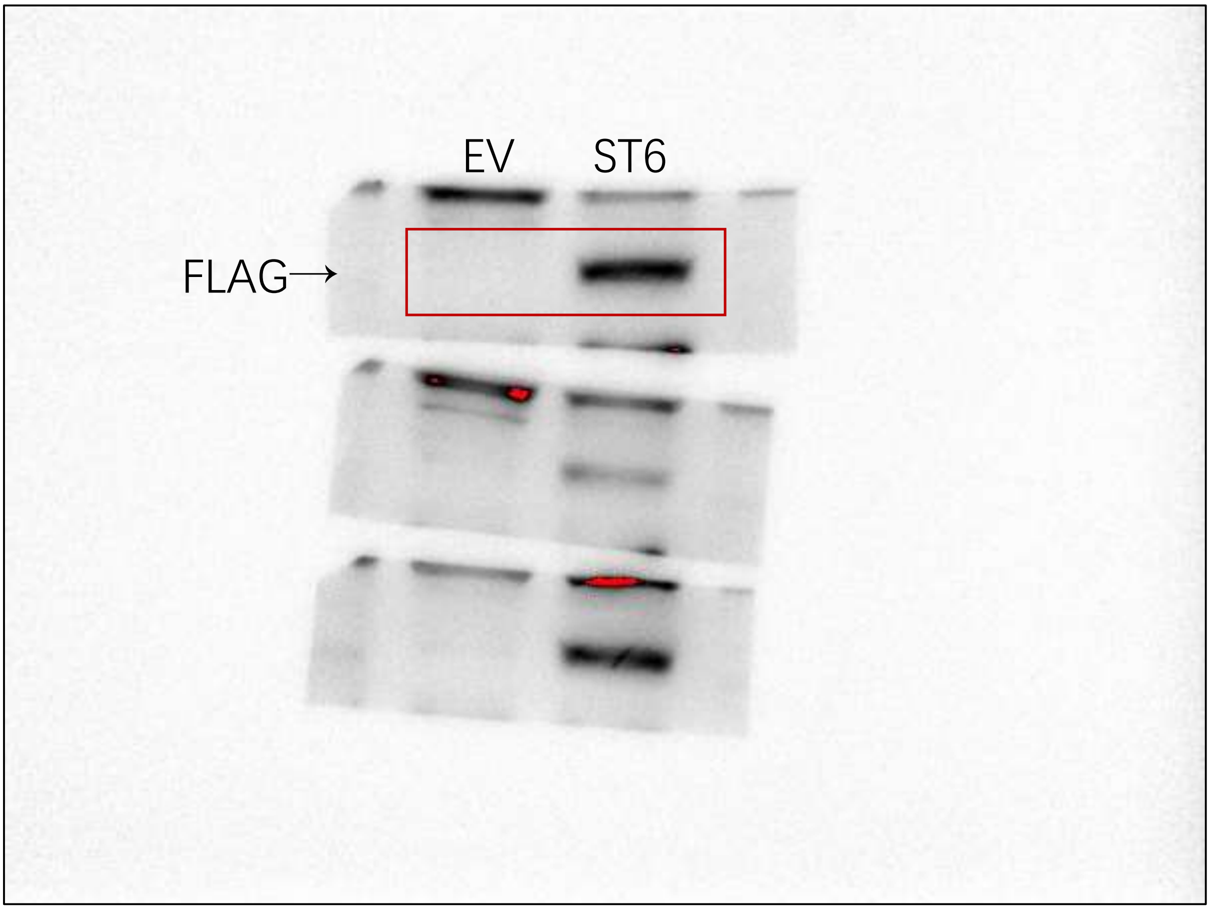

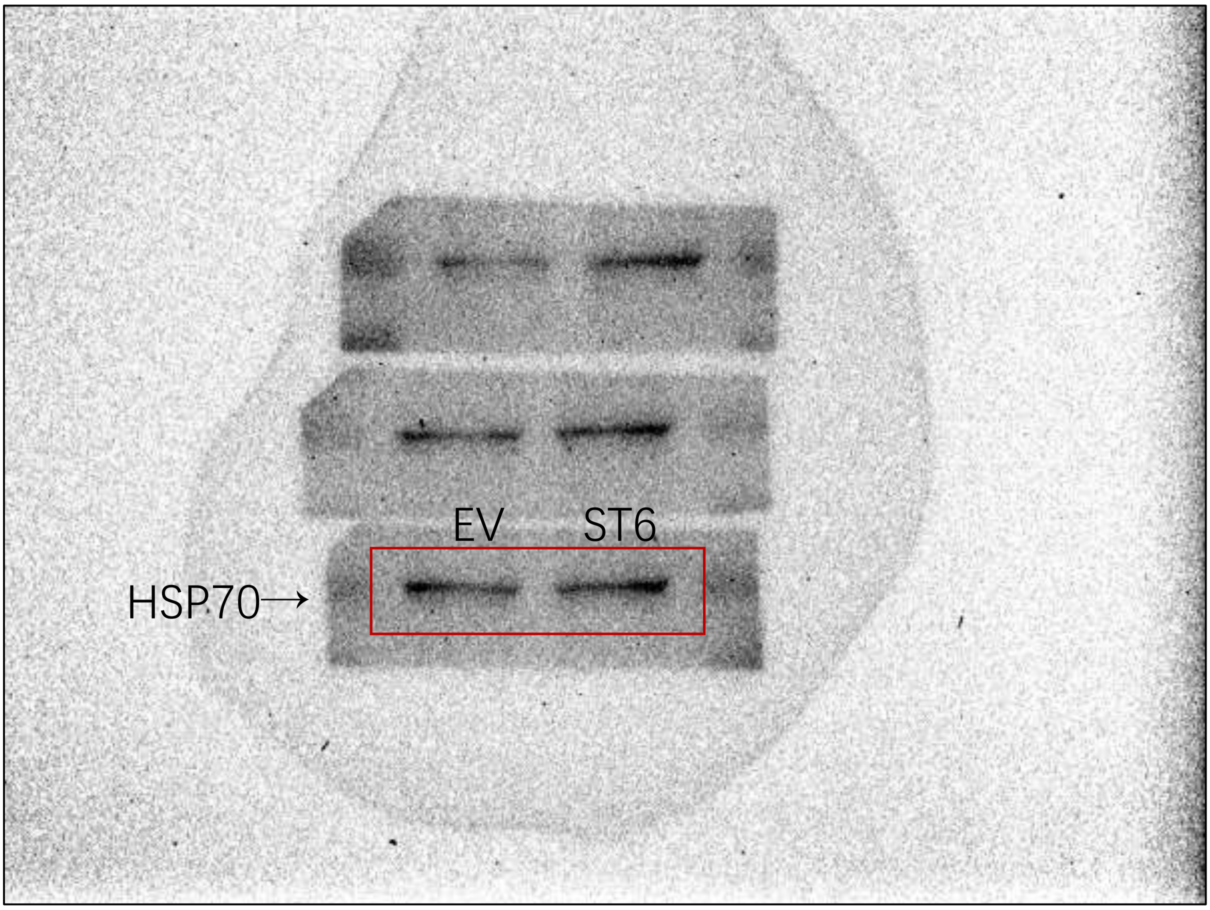

Original images of western blots of APC1 and CDC27 (long exposure) in figure 2a

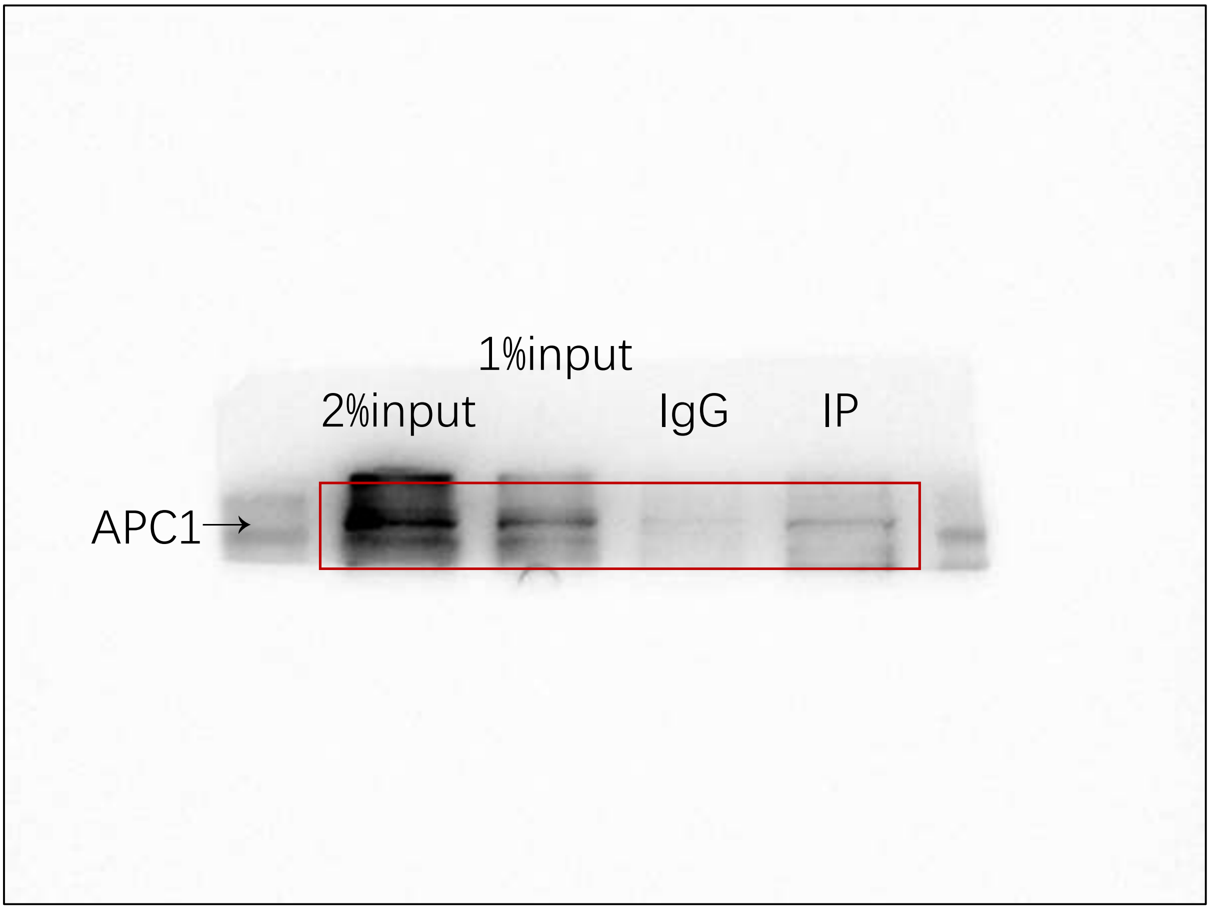

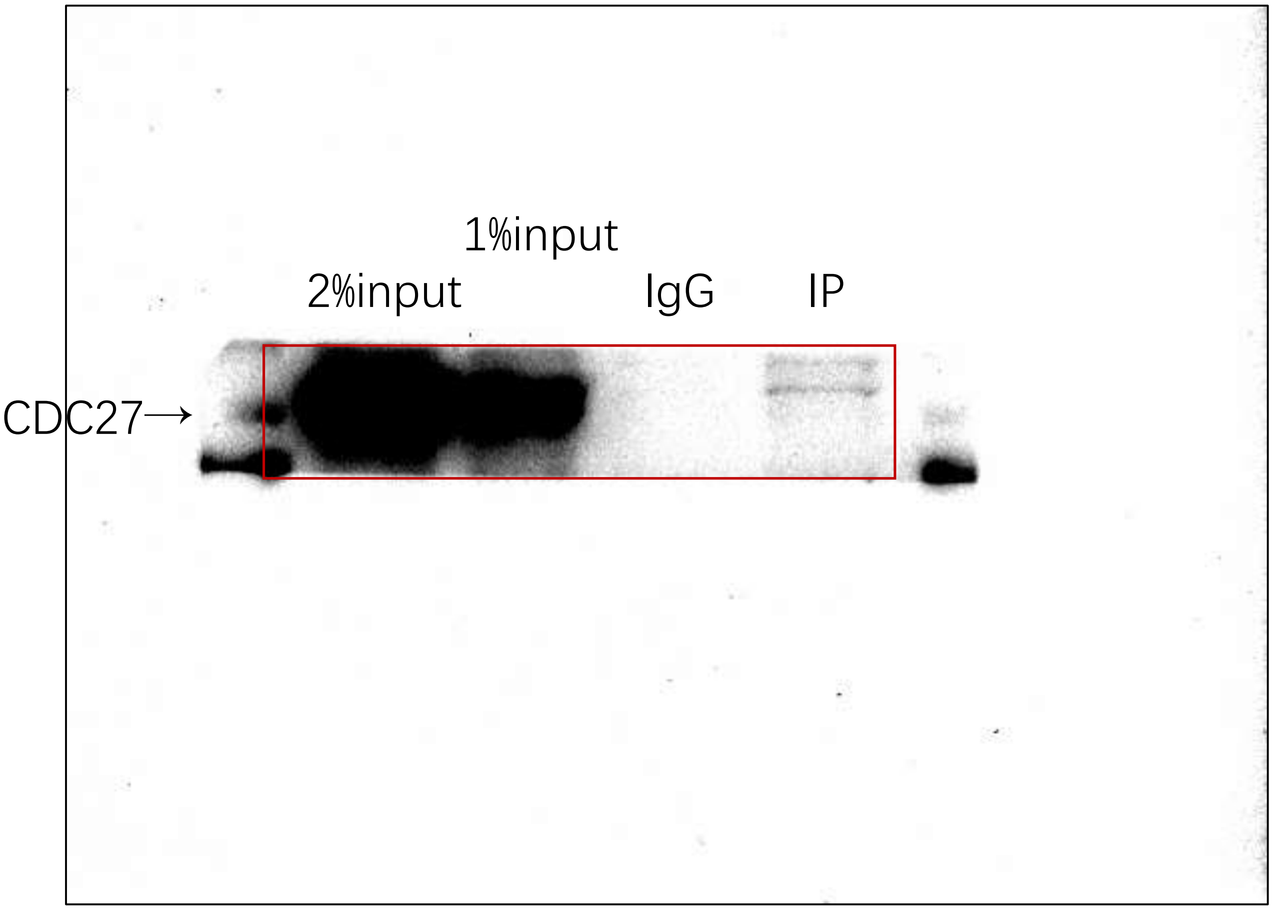

Original images of western blots of CDC27 (short exposure) in figure 2a

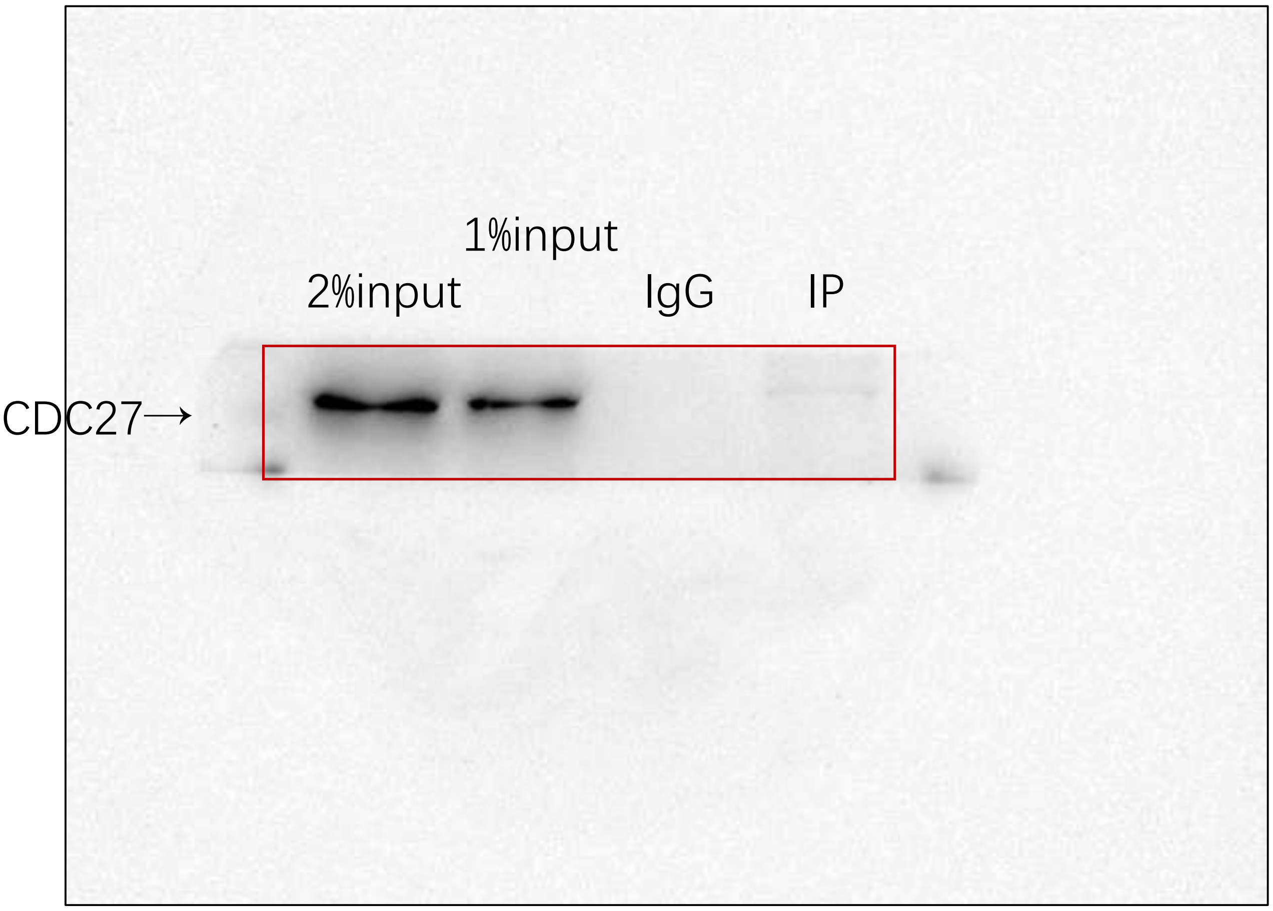

Original images of western blots of CDC20 and CDH1 in figure 2a

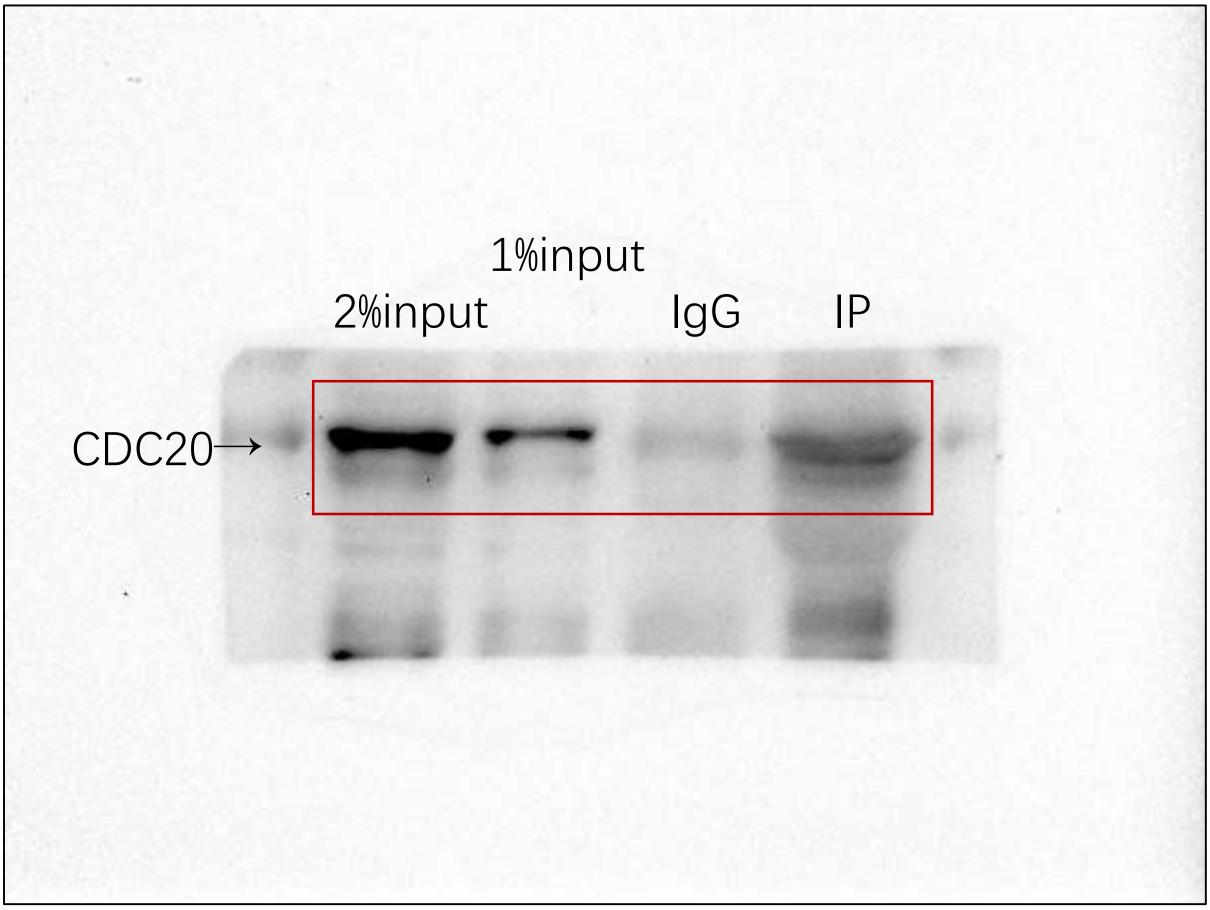

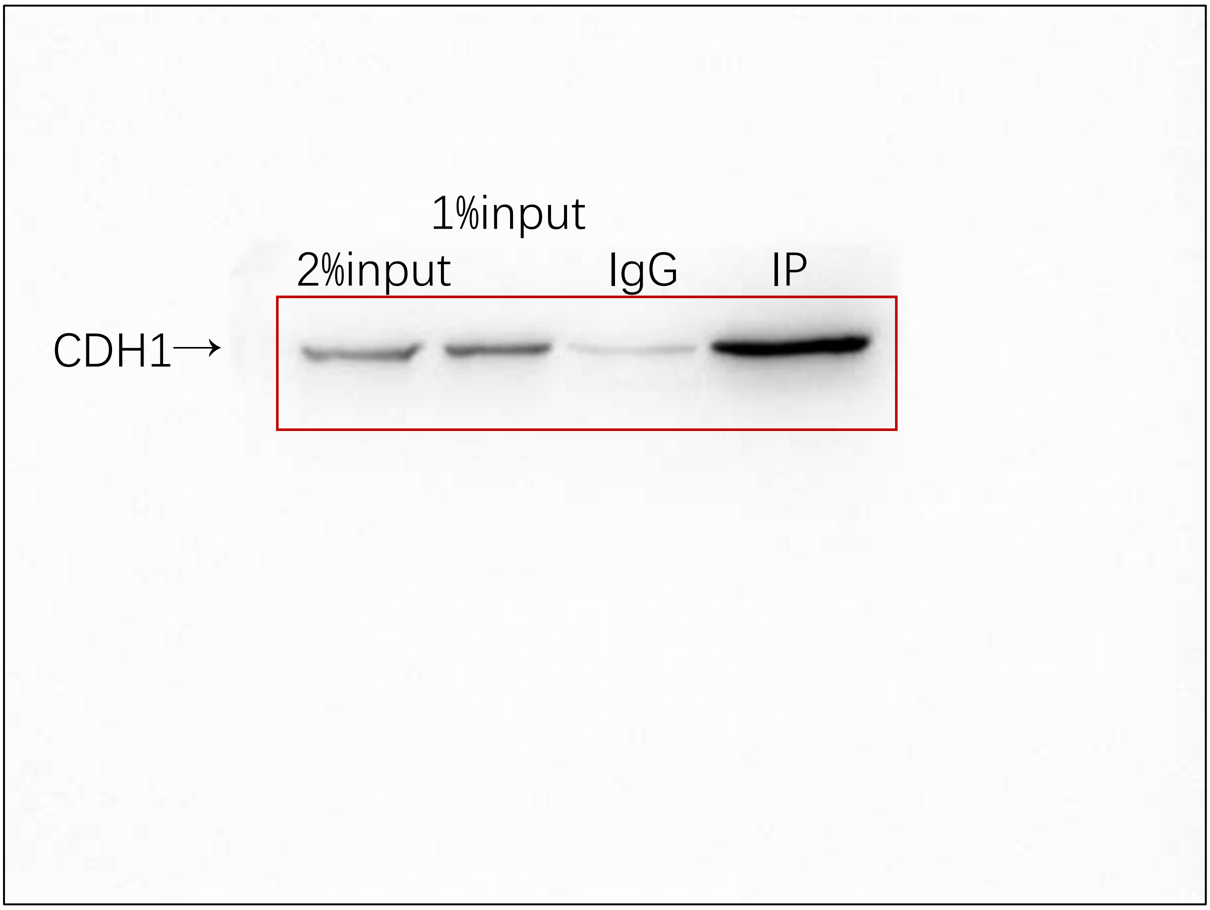

Original images of western blots of SIRT6 in figure 2a

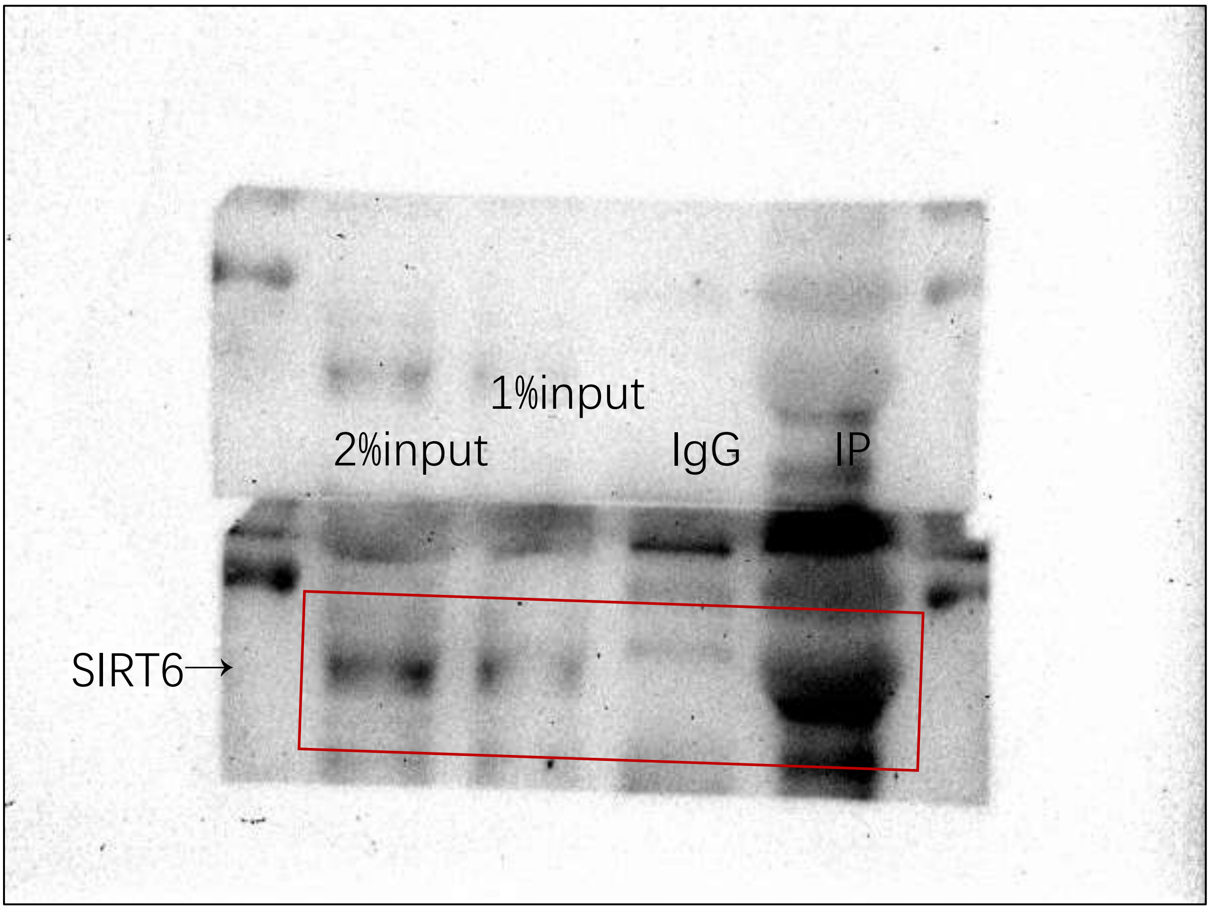

Original images of western blots of SIRT6 and APC1 in figure 2b

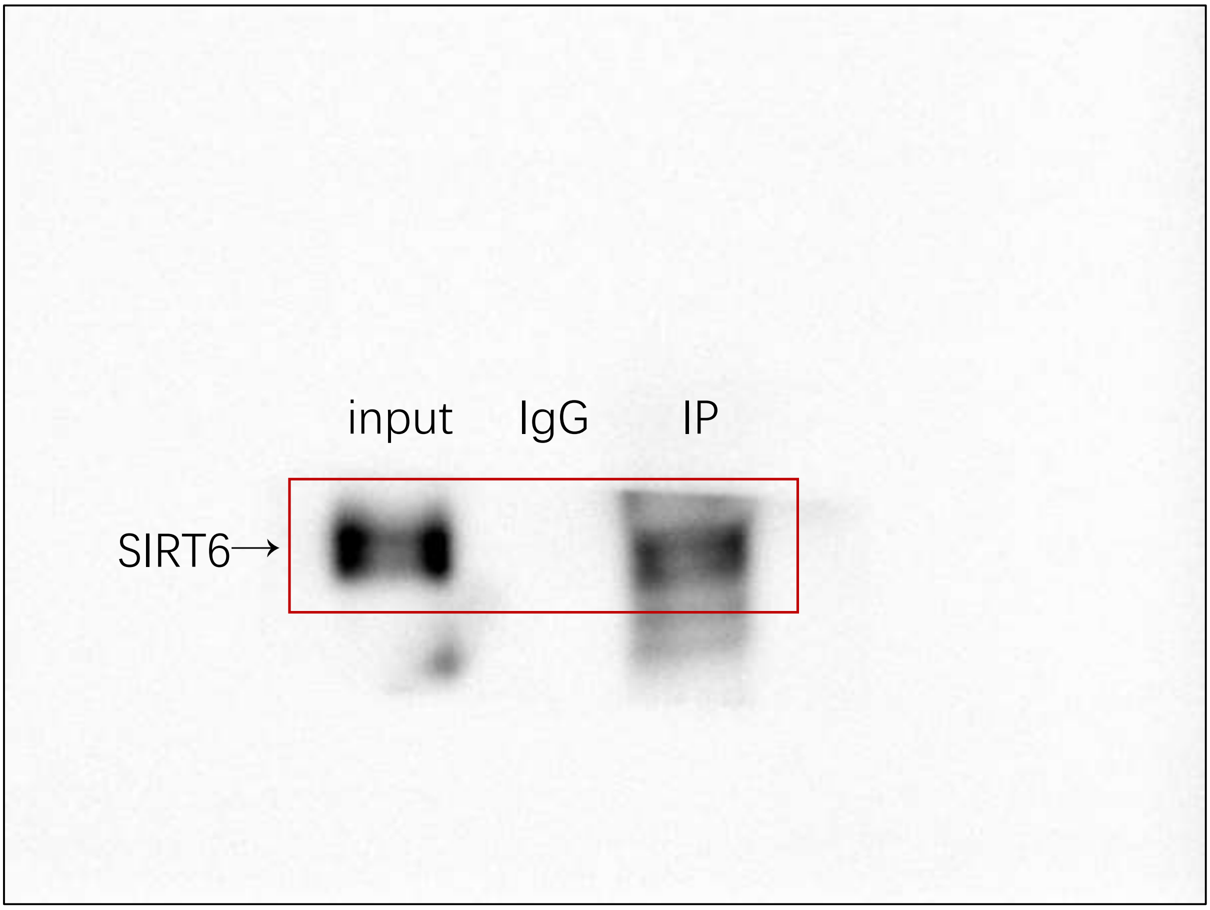

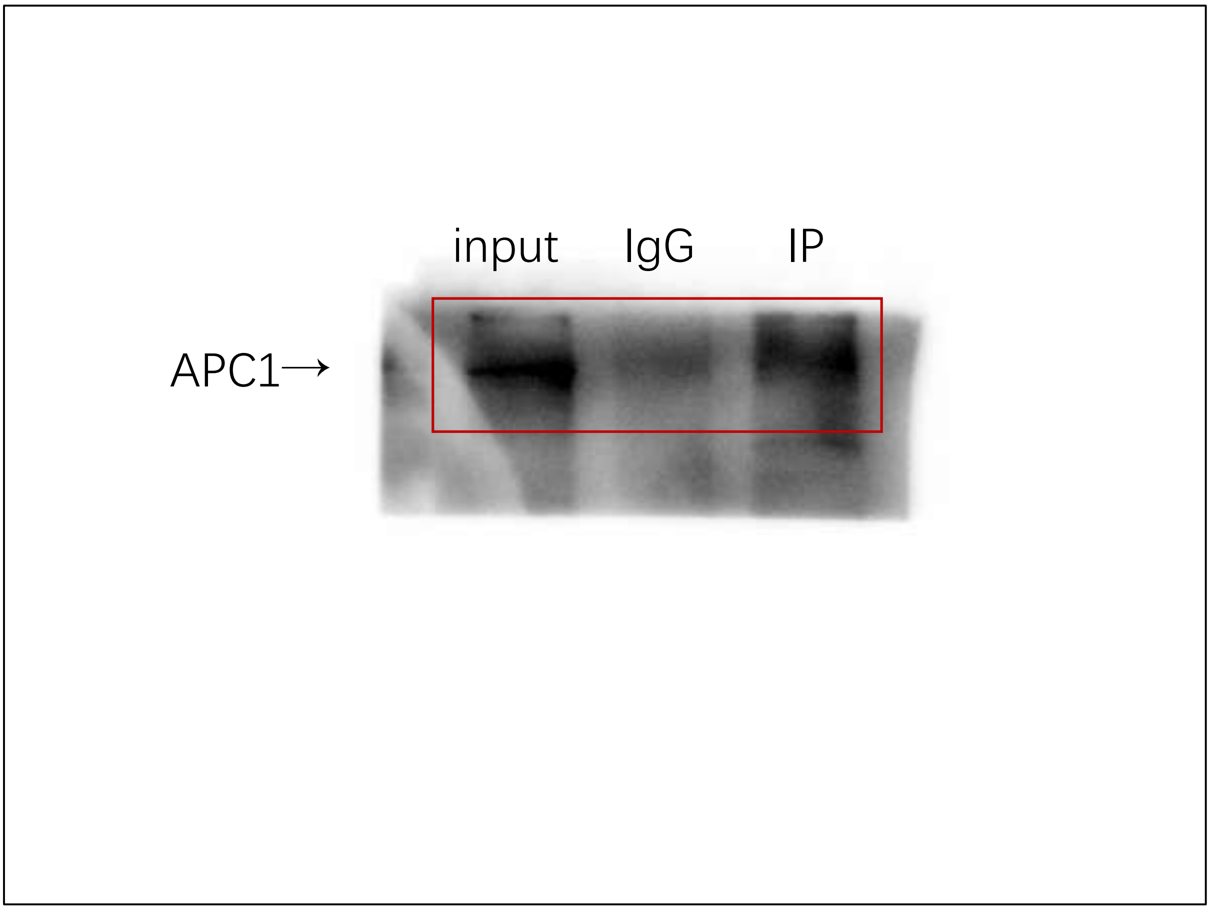

Original images of western blots of SIRT6 and CDC20 in figure 2c

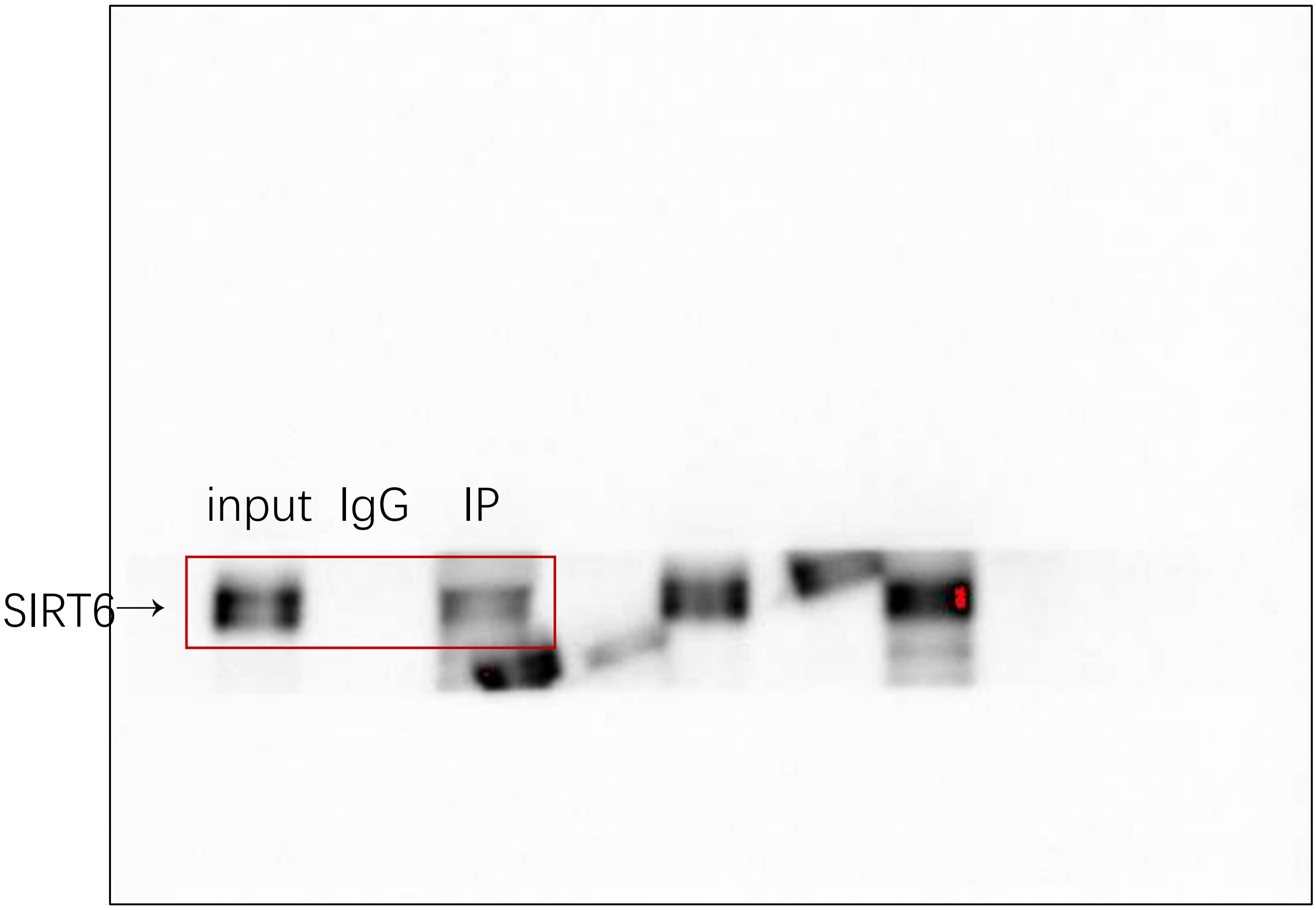

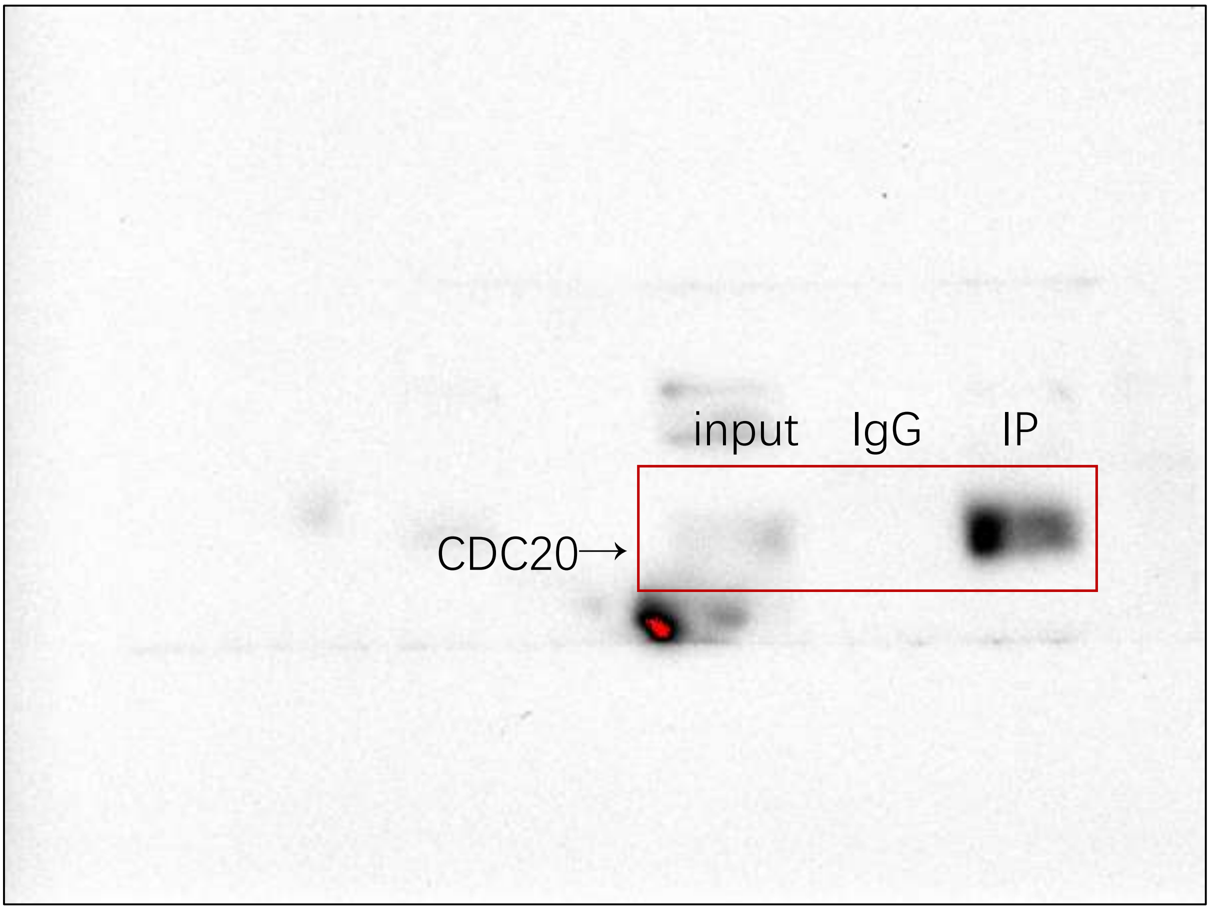

Original images of western blots of SIRT6 and CDH1 in figure 2d

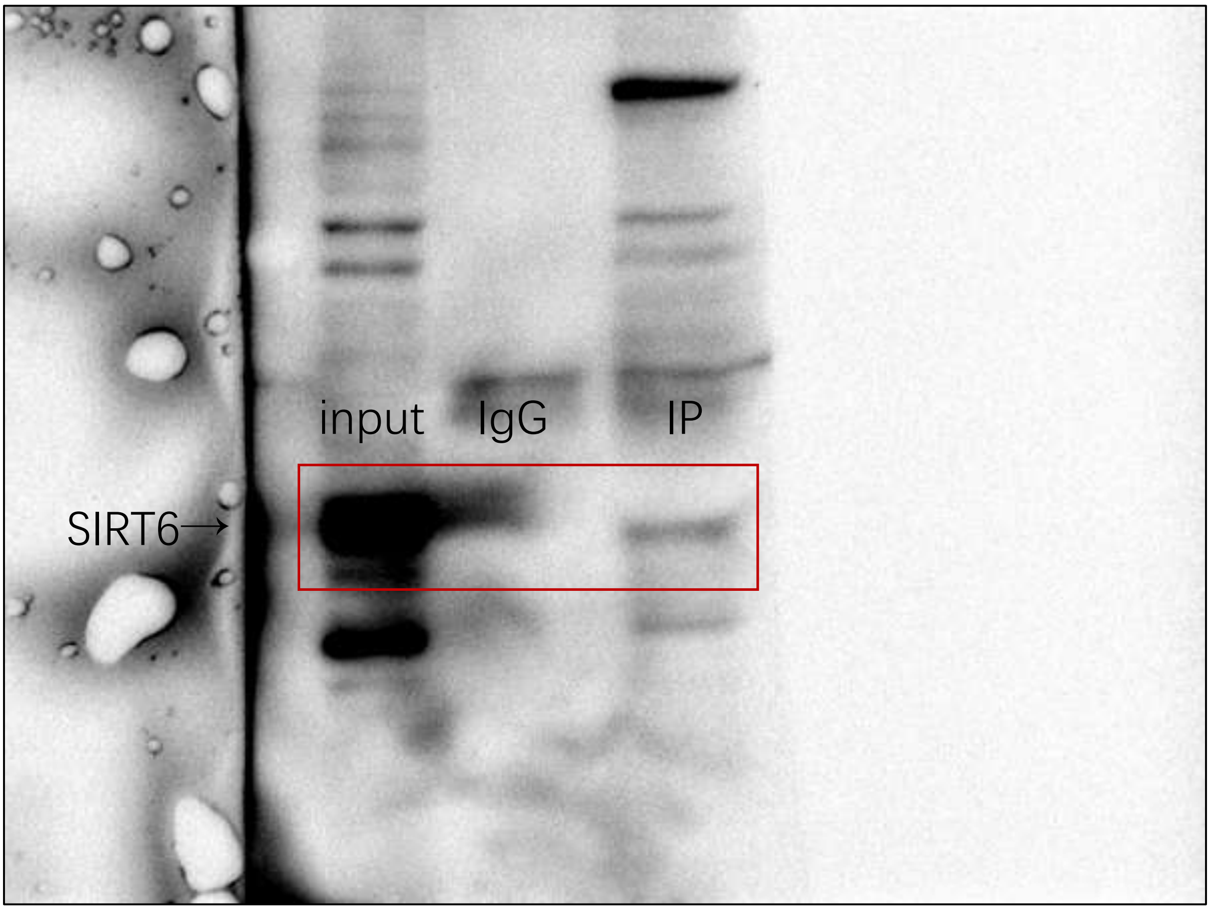

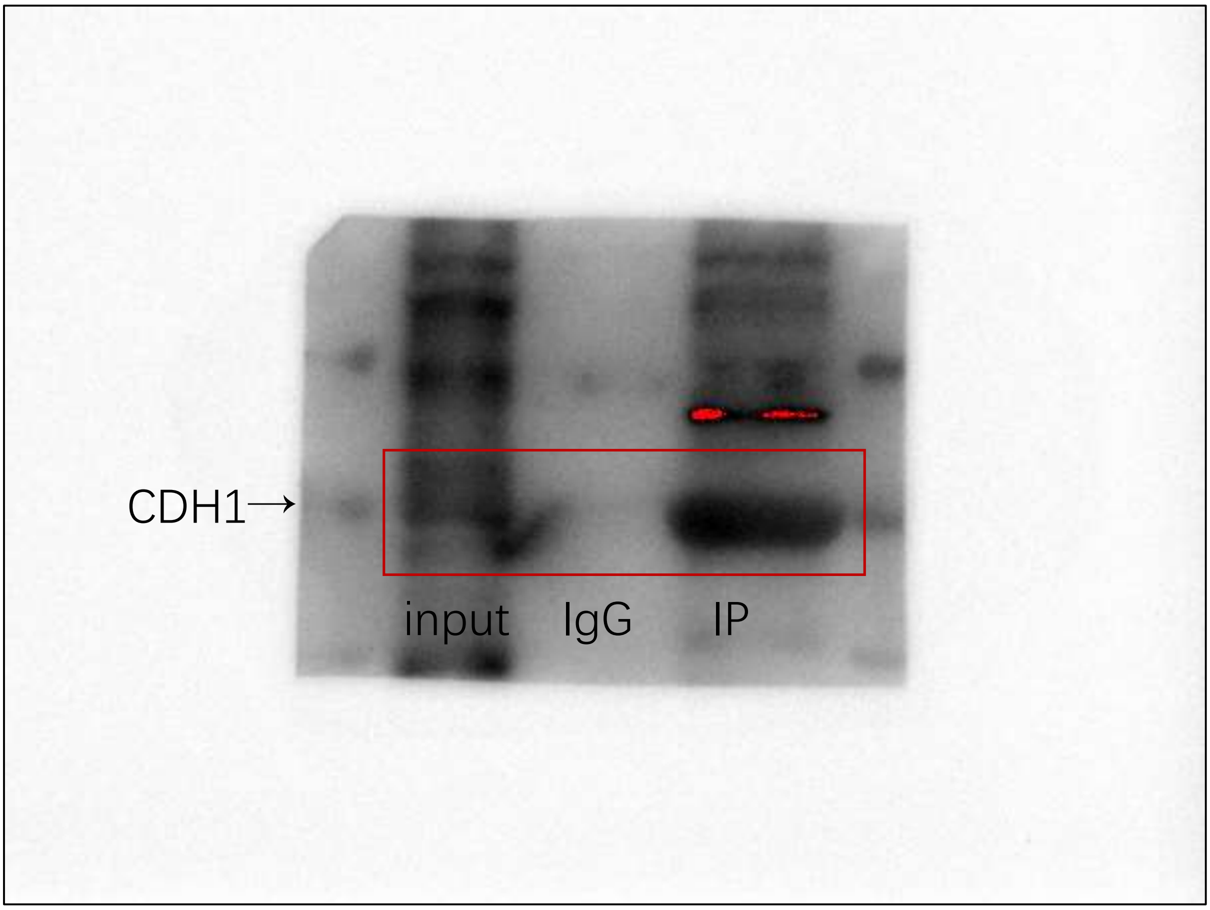

Original images of western blots of SIRT6-FLAG and CDC20 in figure 2e

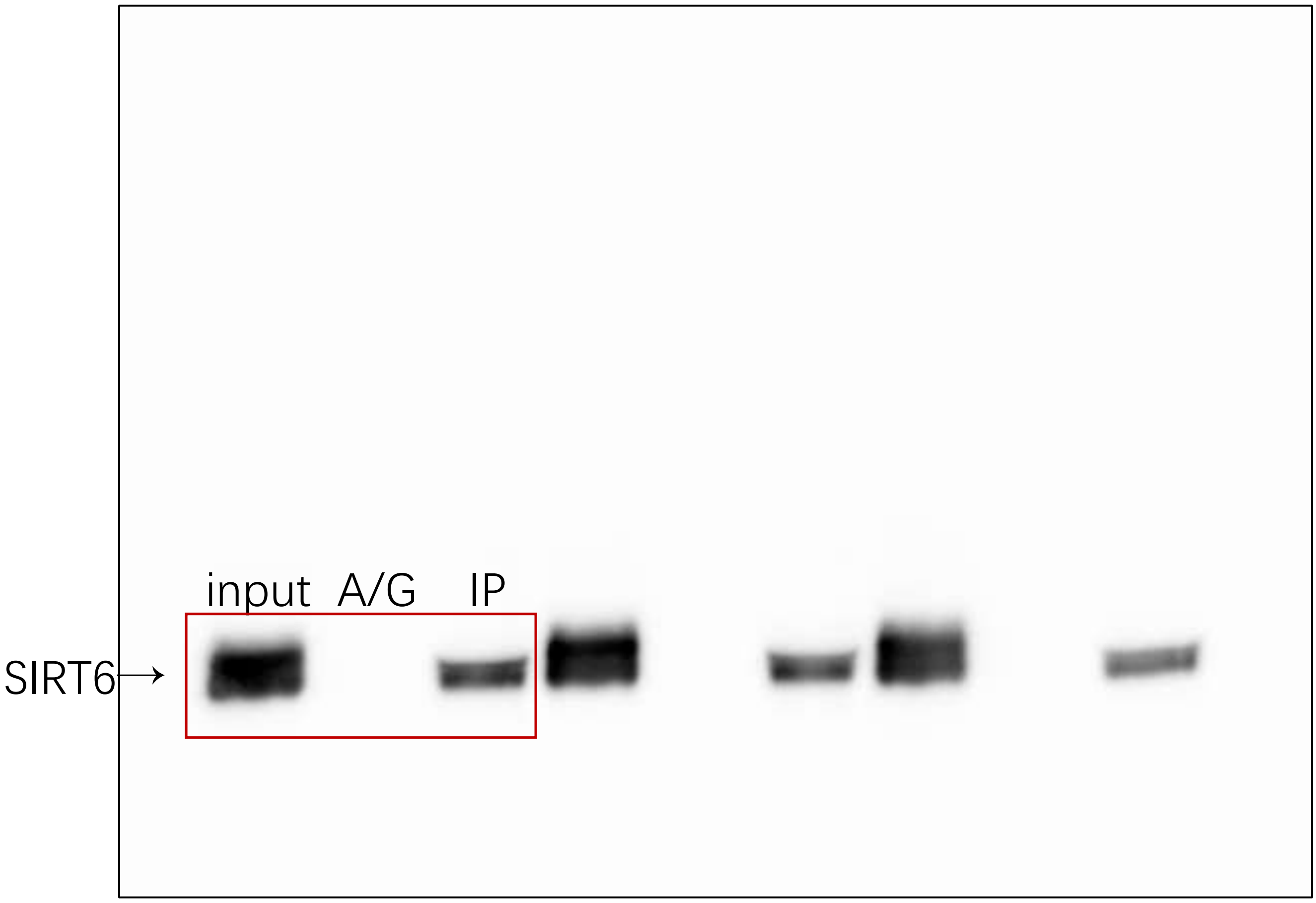

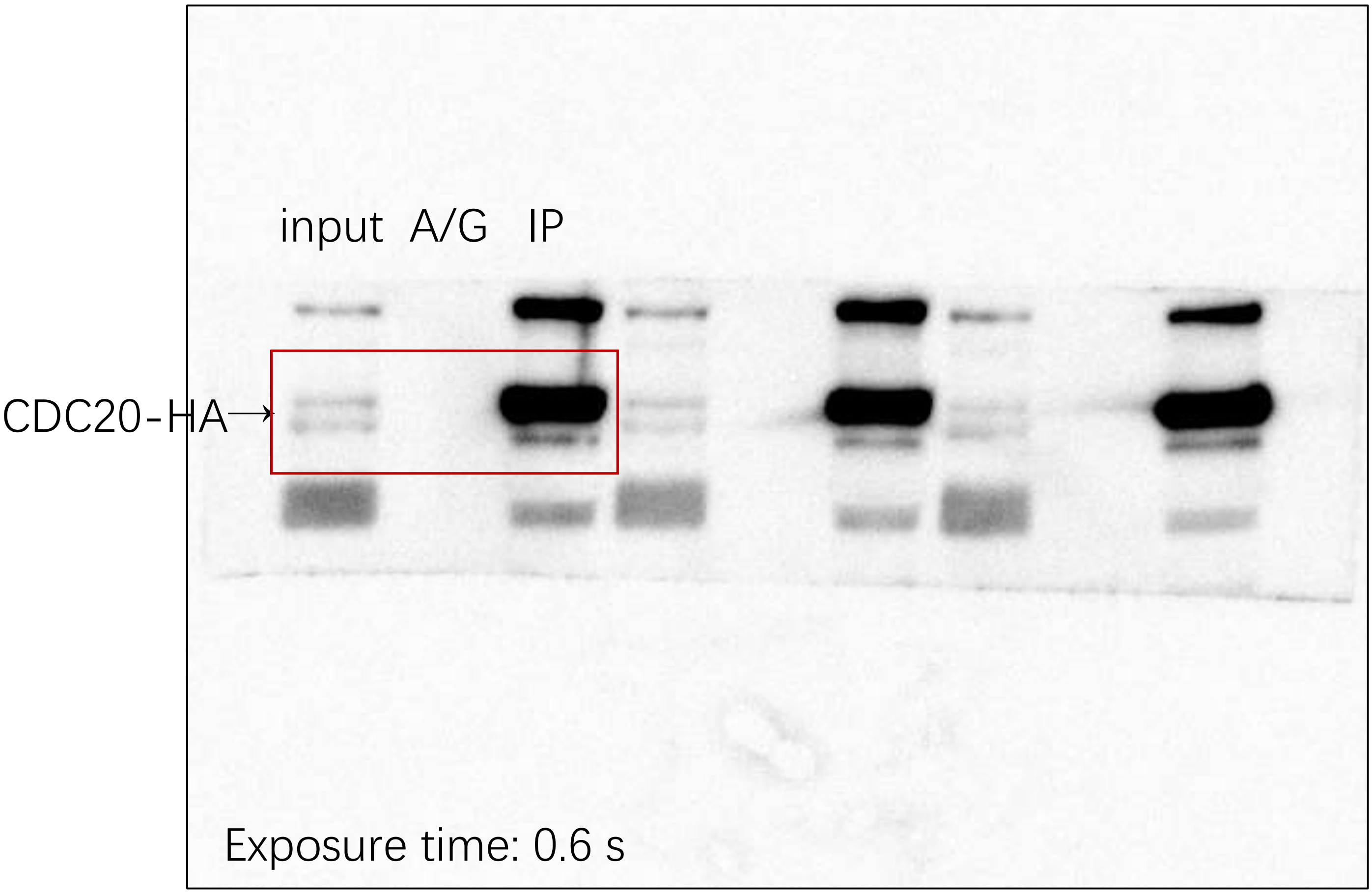

Original images of western blots of CDC20 with short exposure time in figure 2e

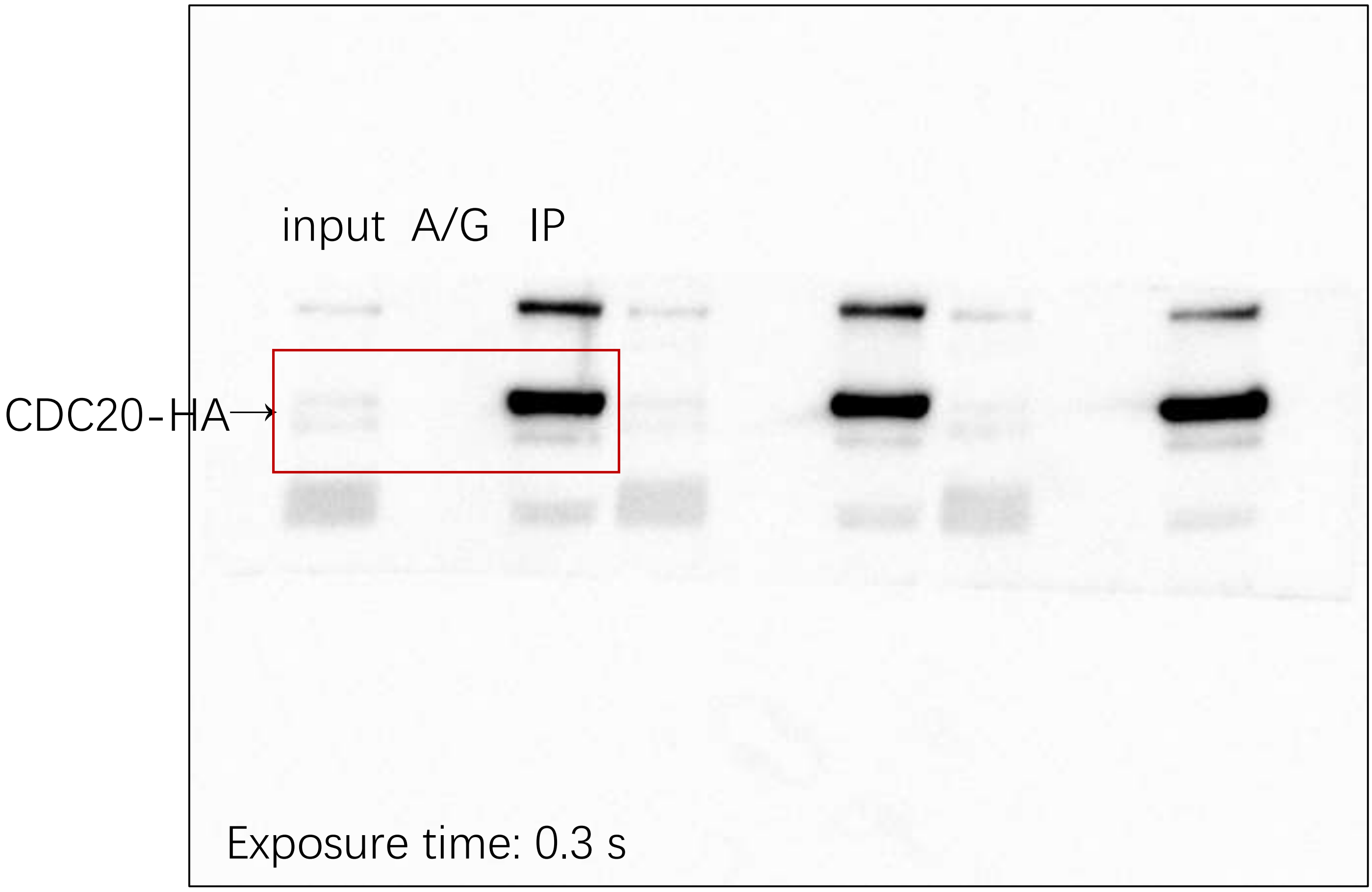

Original images of western blots of SIRT6-FLAG and CDH1 in figure 2f

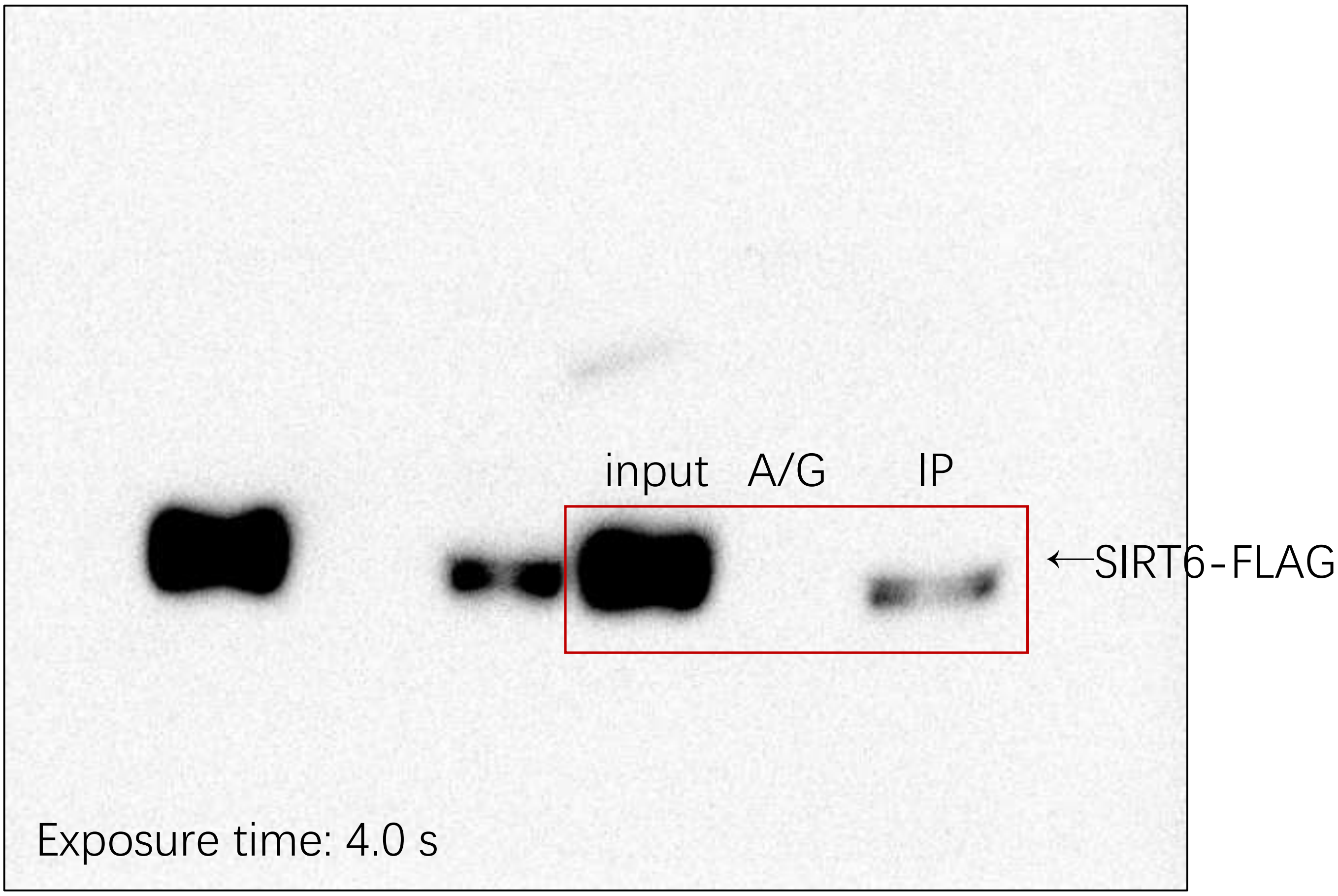

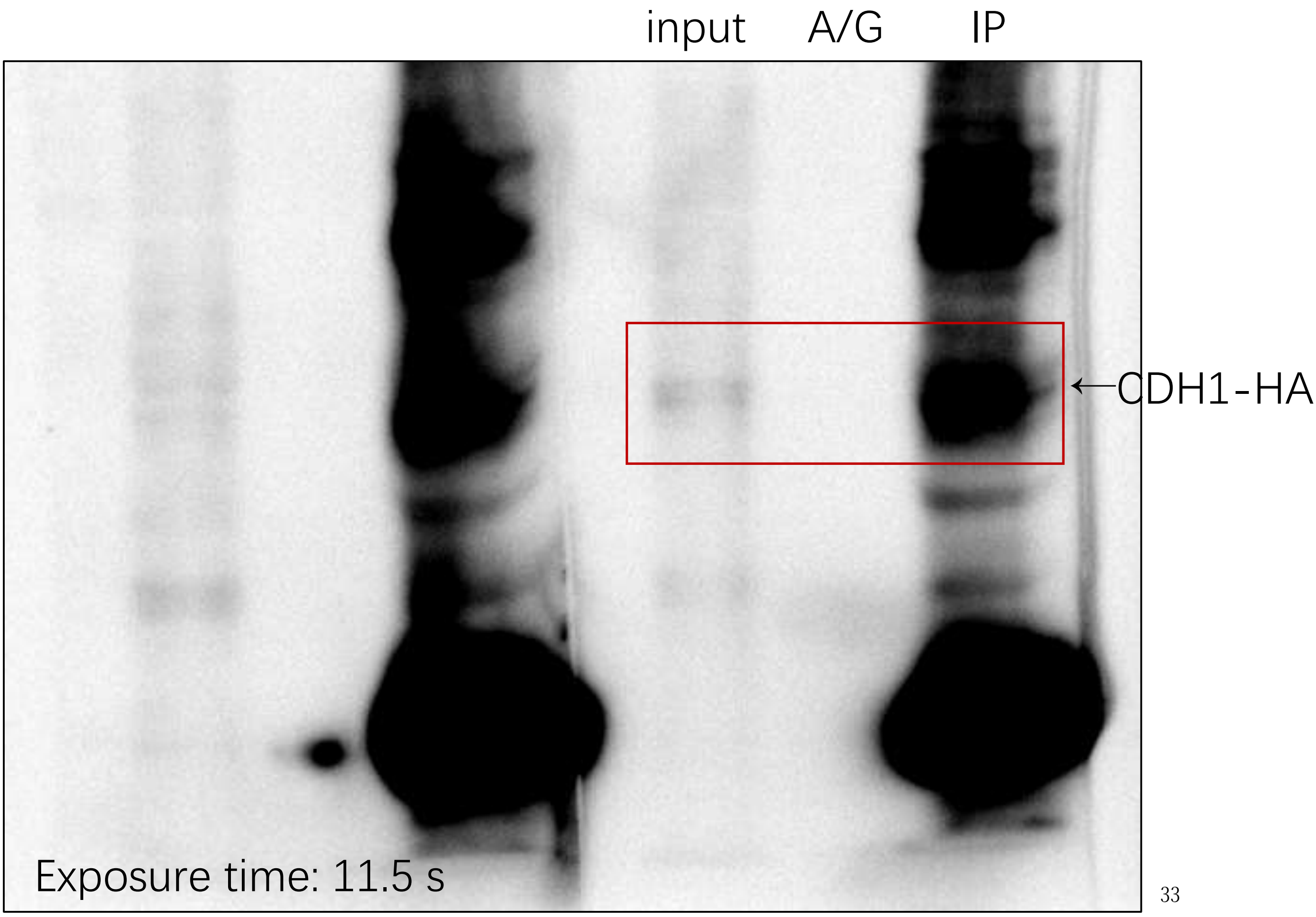

Original images of western blots of SIRT6-FLAG and CDH1 in figure 2f

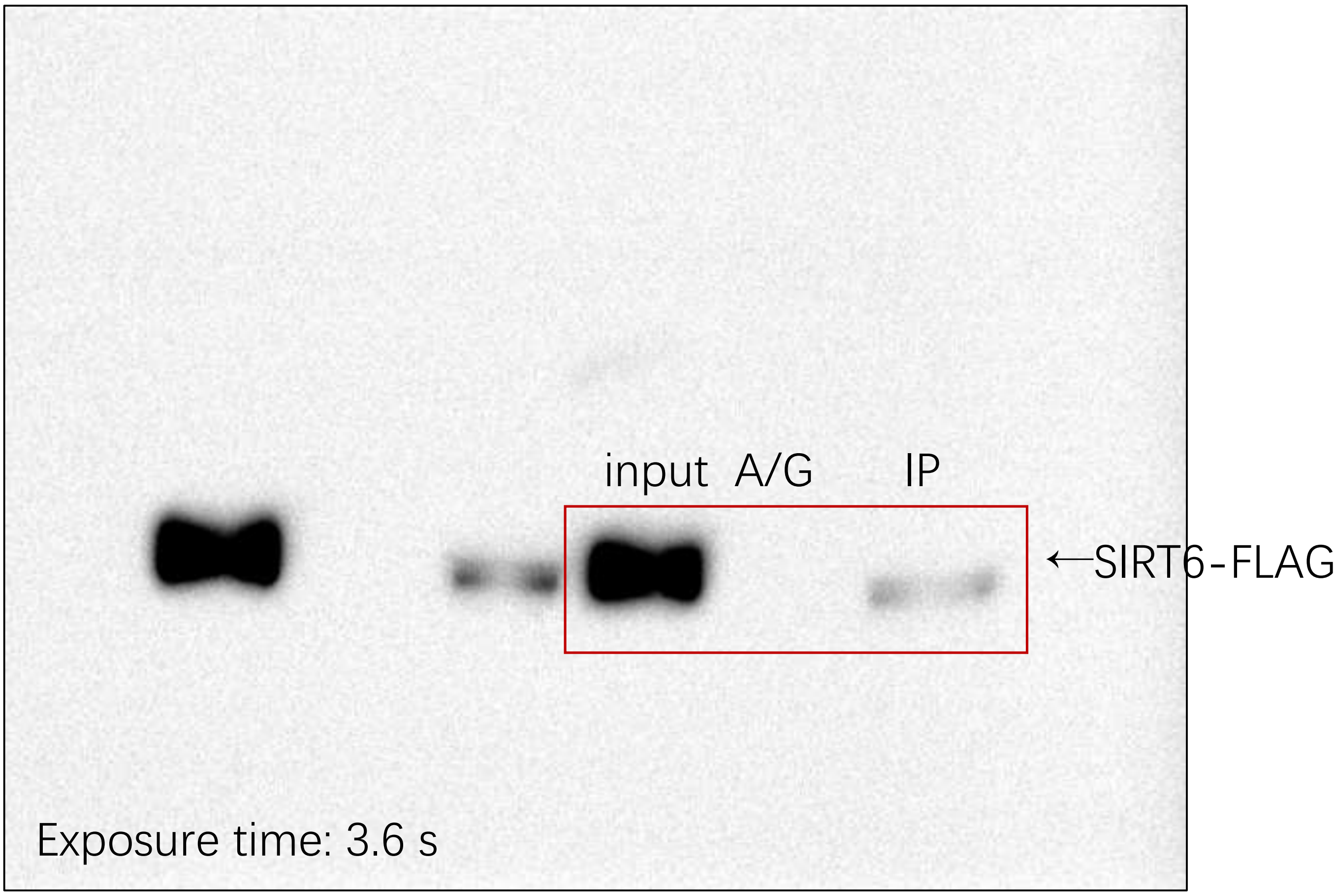

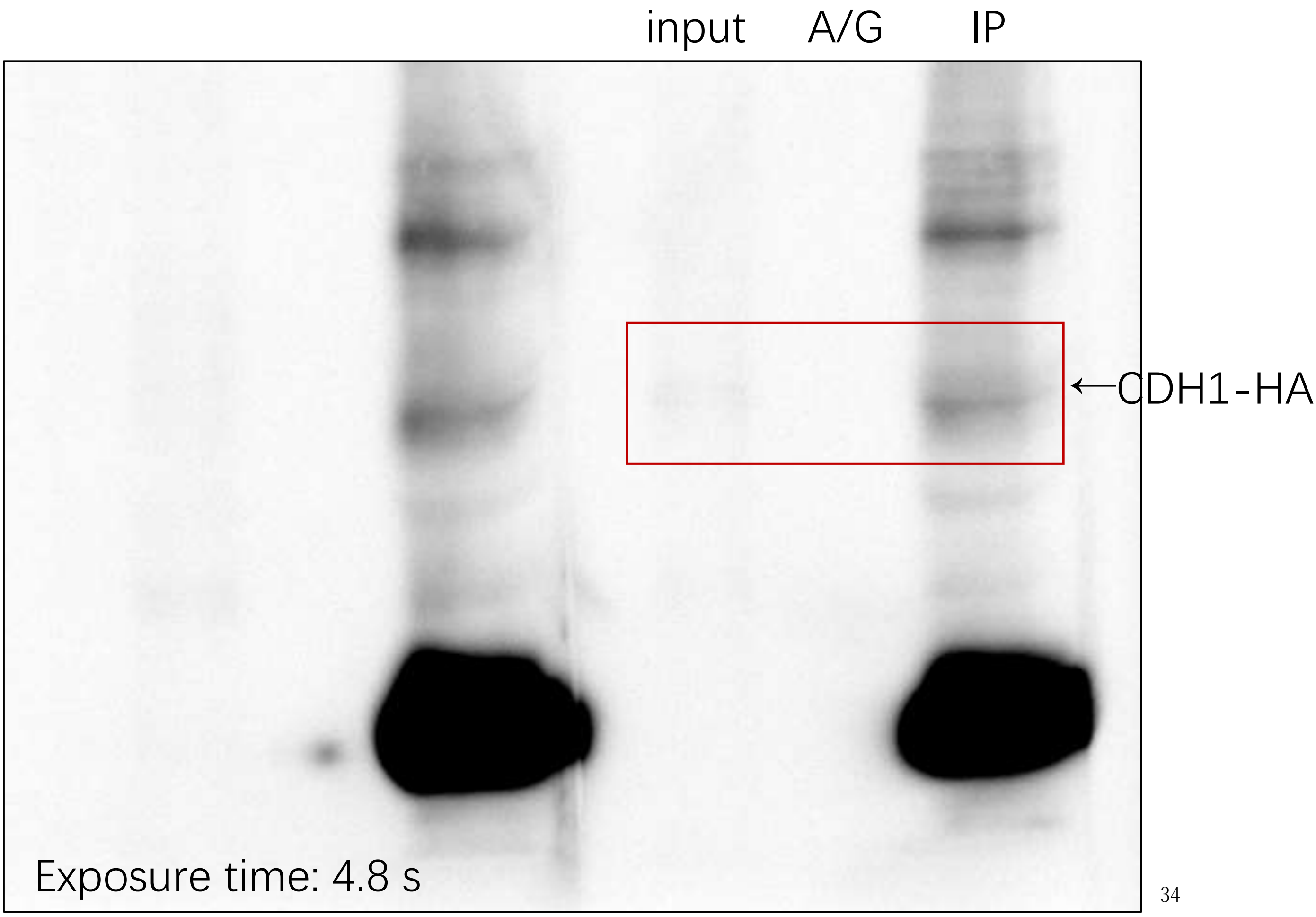

Original images of western blots of SIRT6 and securin in figure 3a

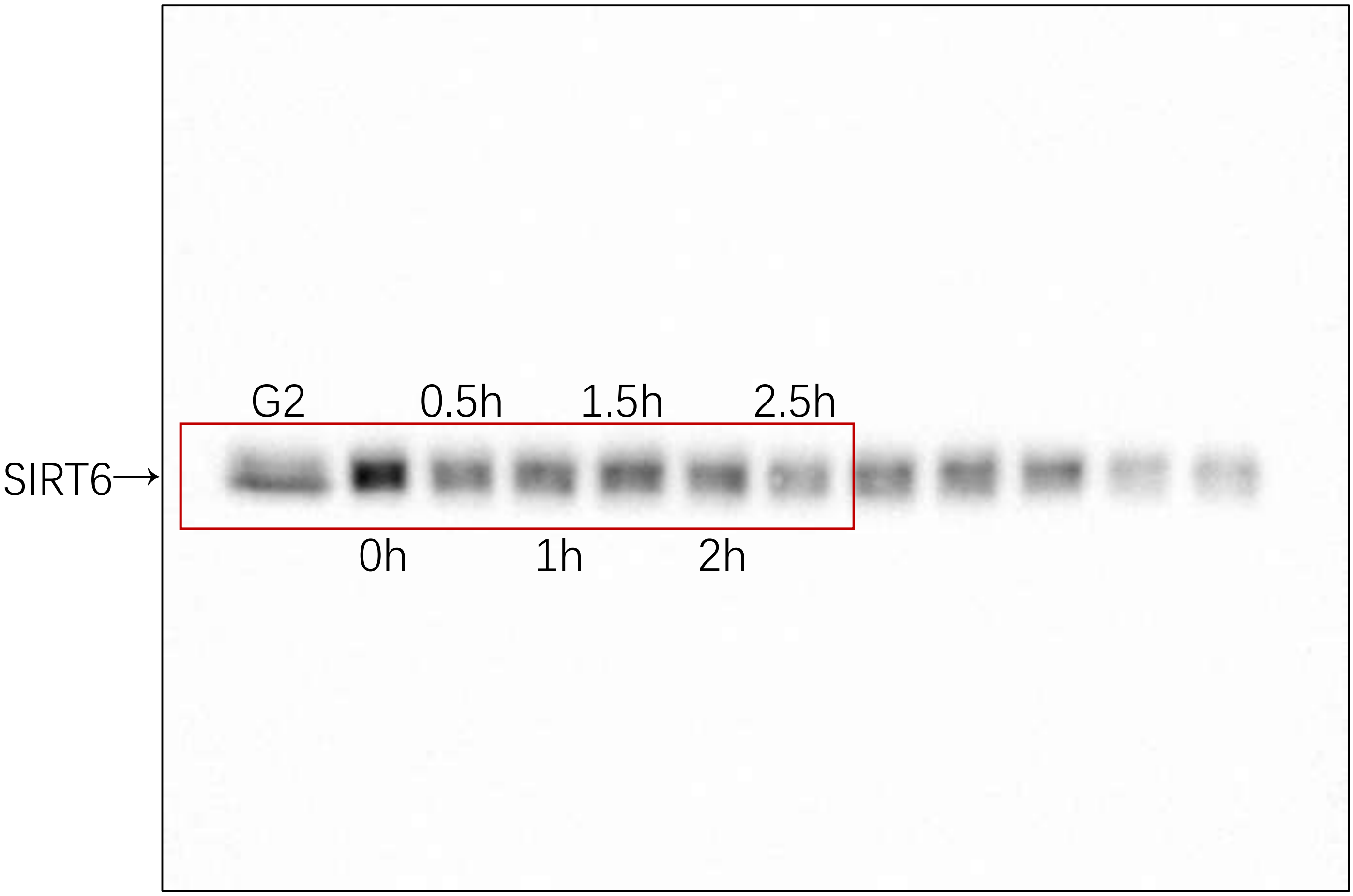

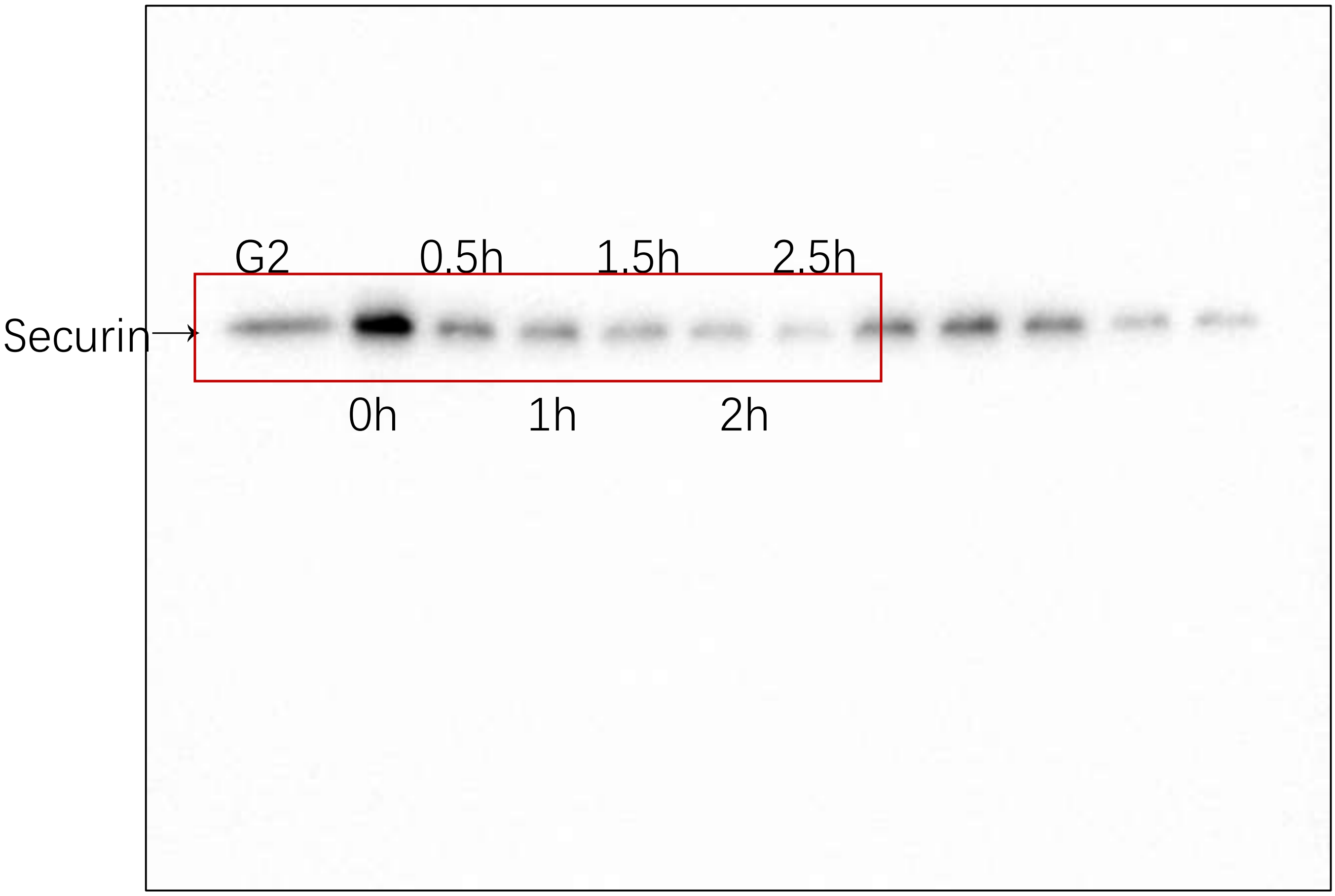

Original images of western blots of cyclin A2 and cyclin B1 in figure 3a

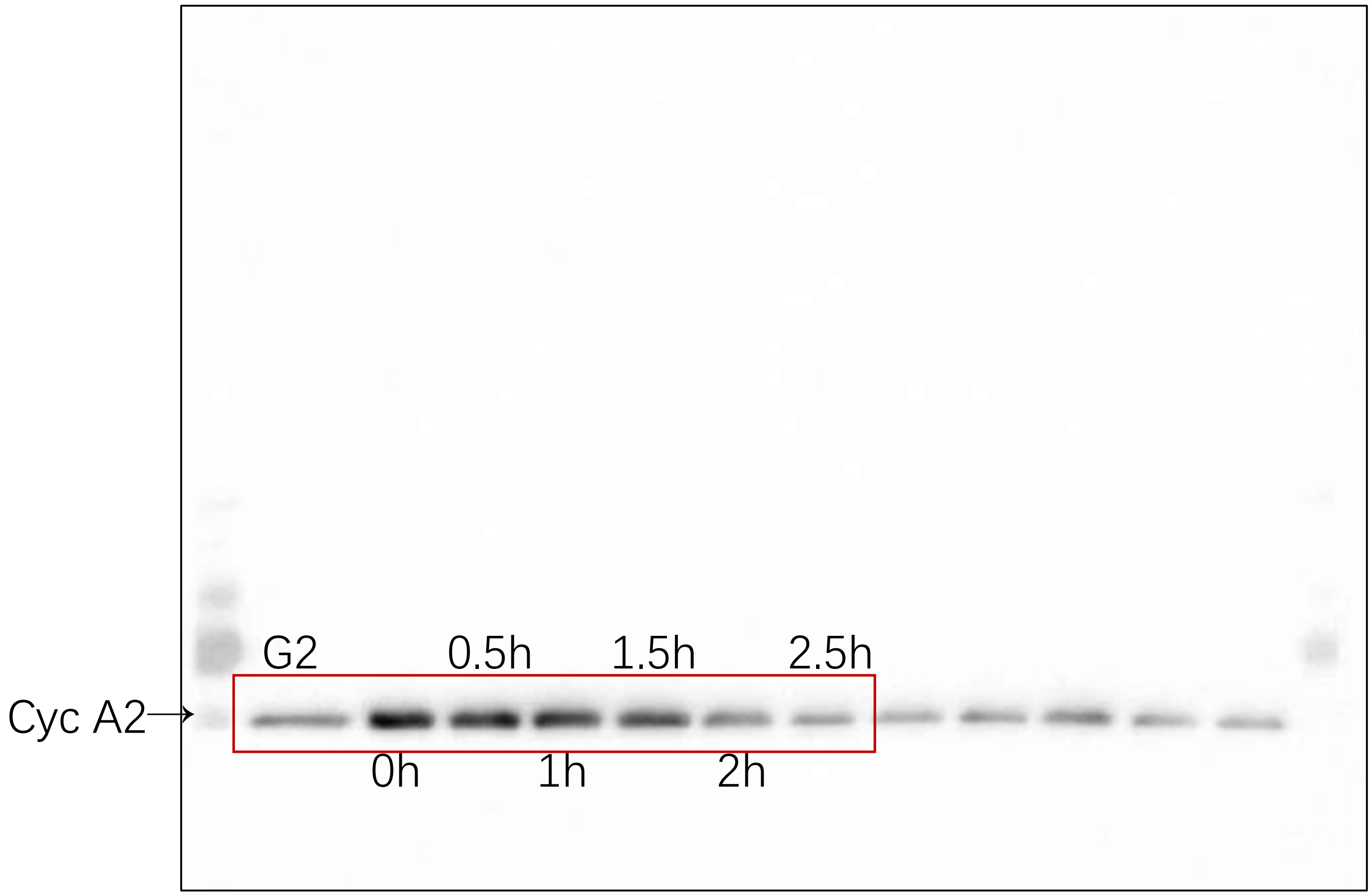

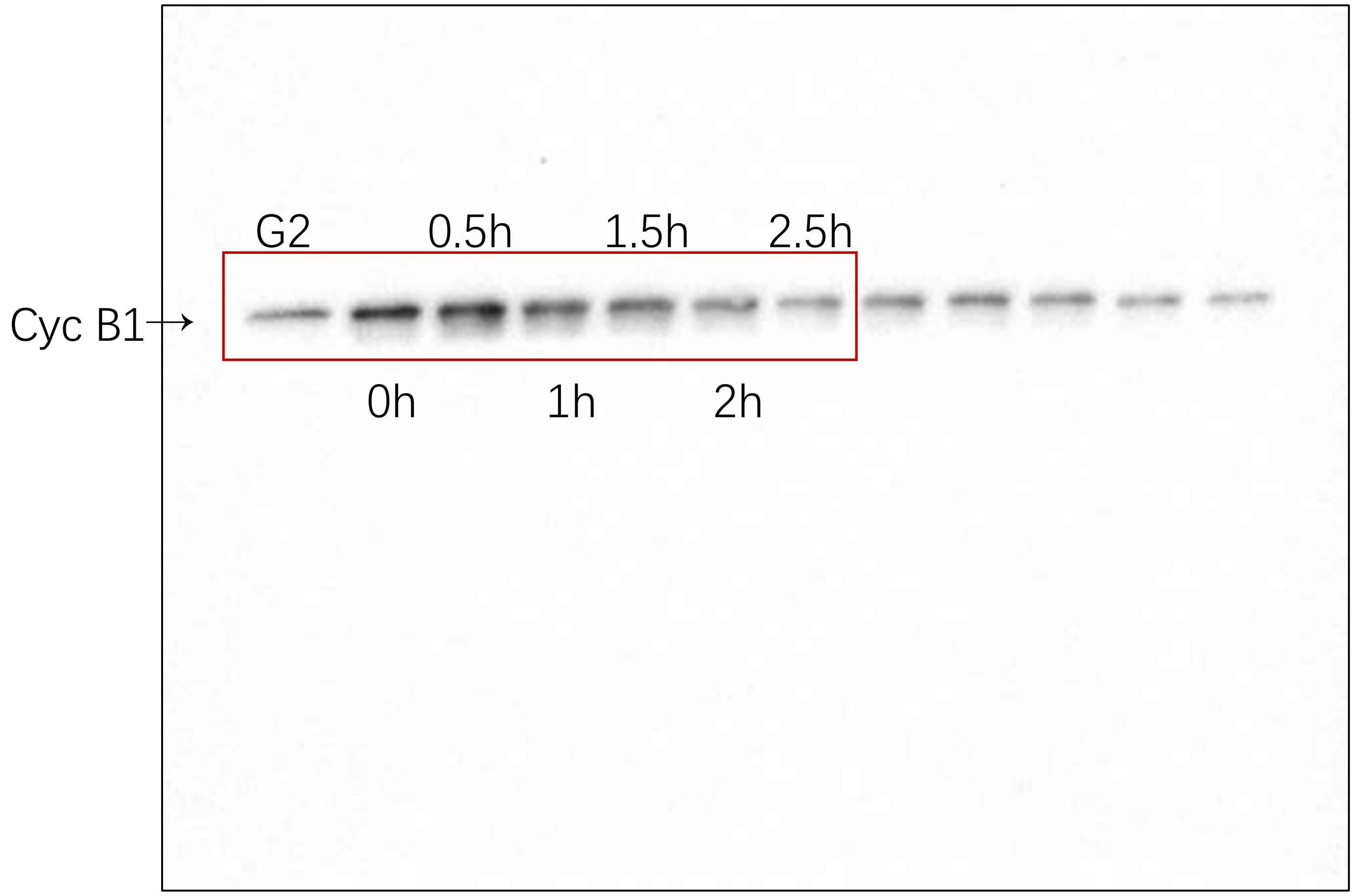

Original images of western blots of pH3@Ser10 and actin in figure 3a

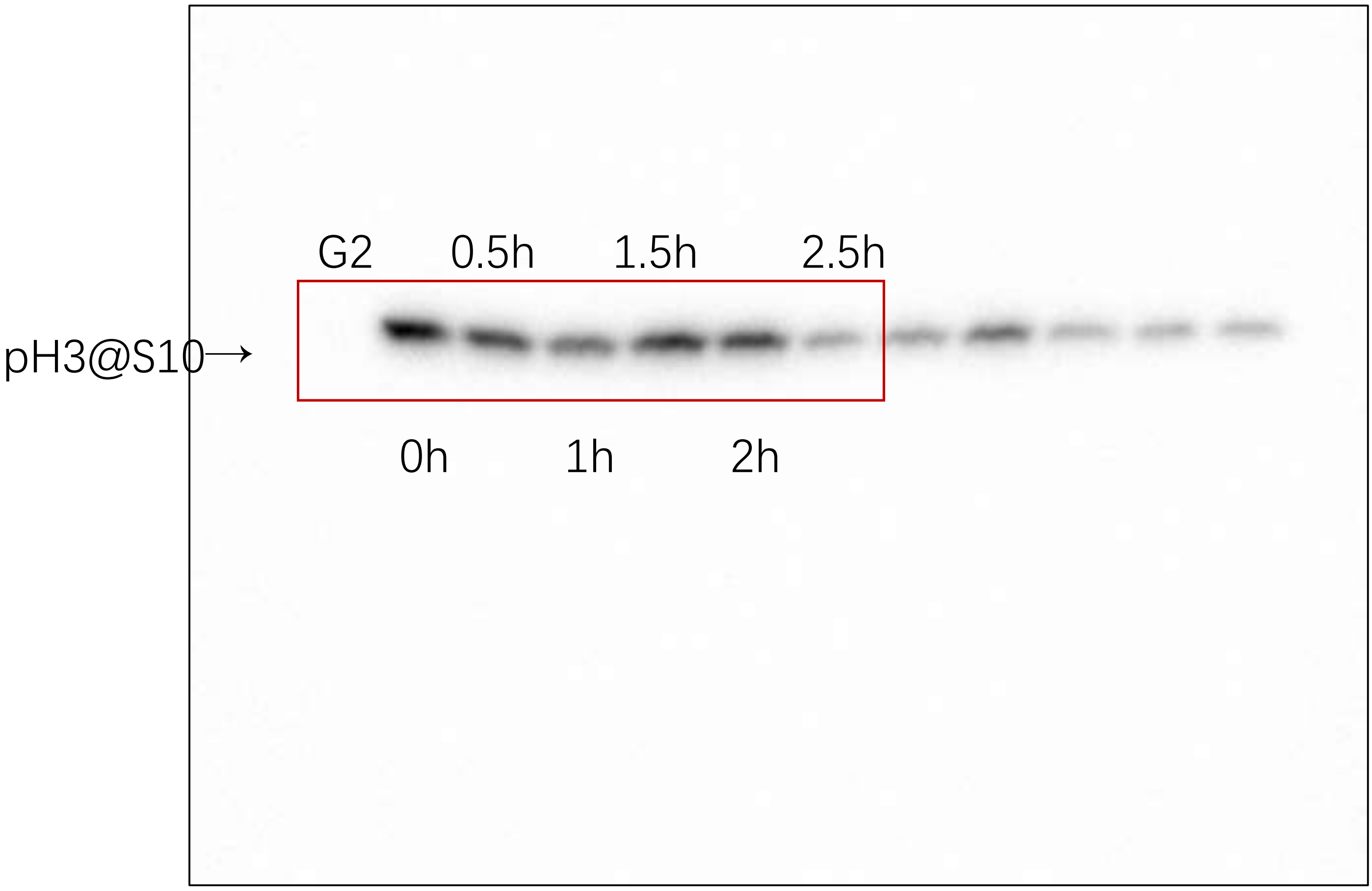

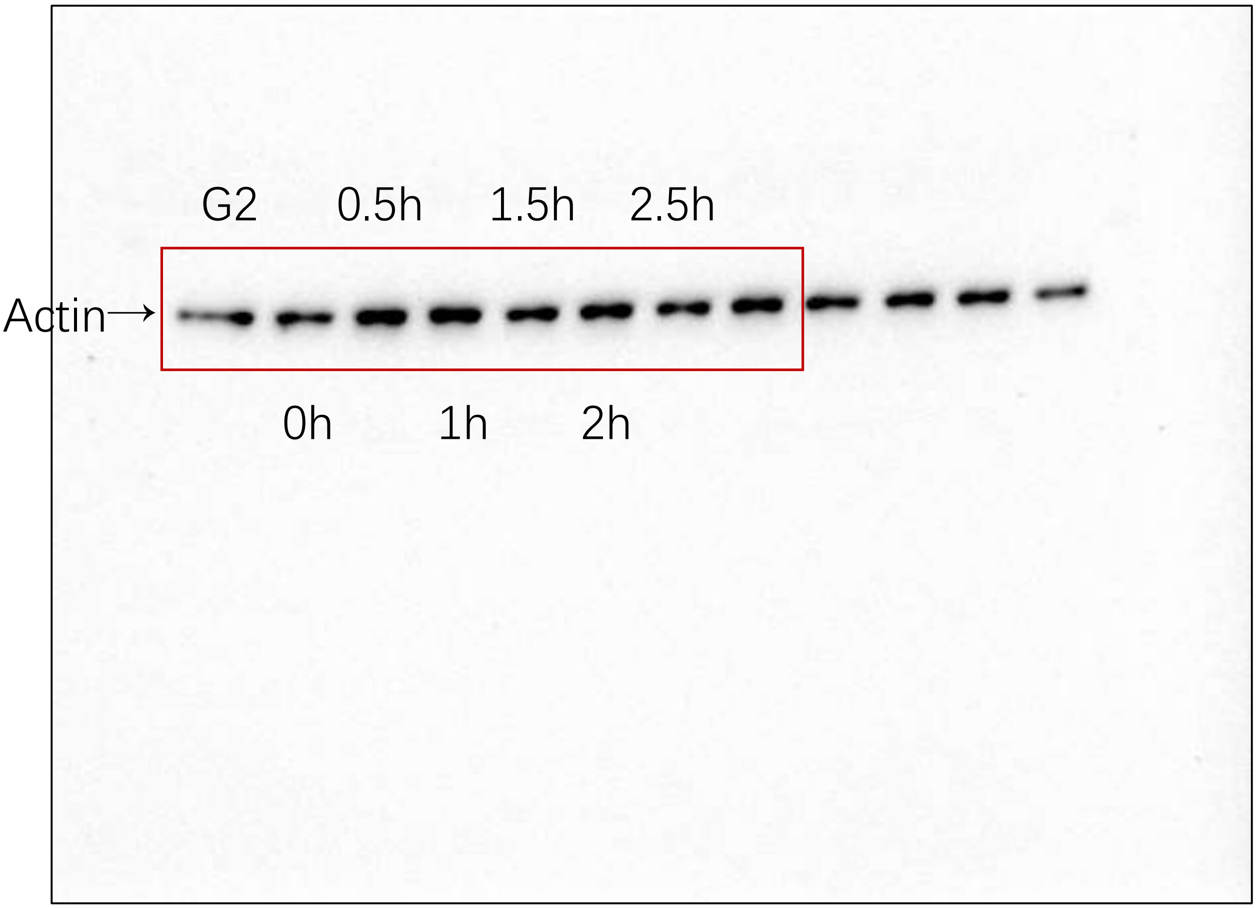

Original images of western blots of CDH1 in figure 3a

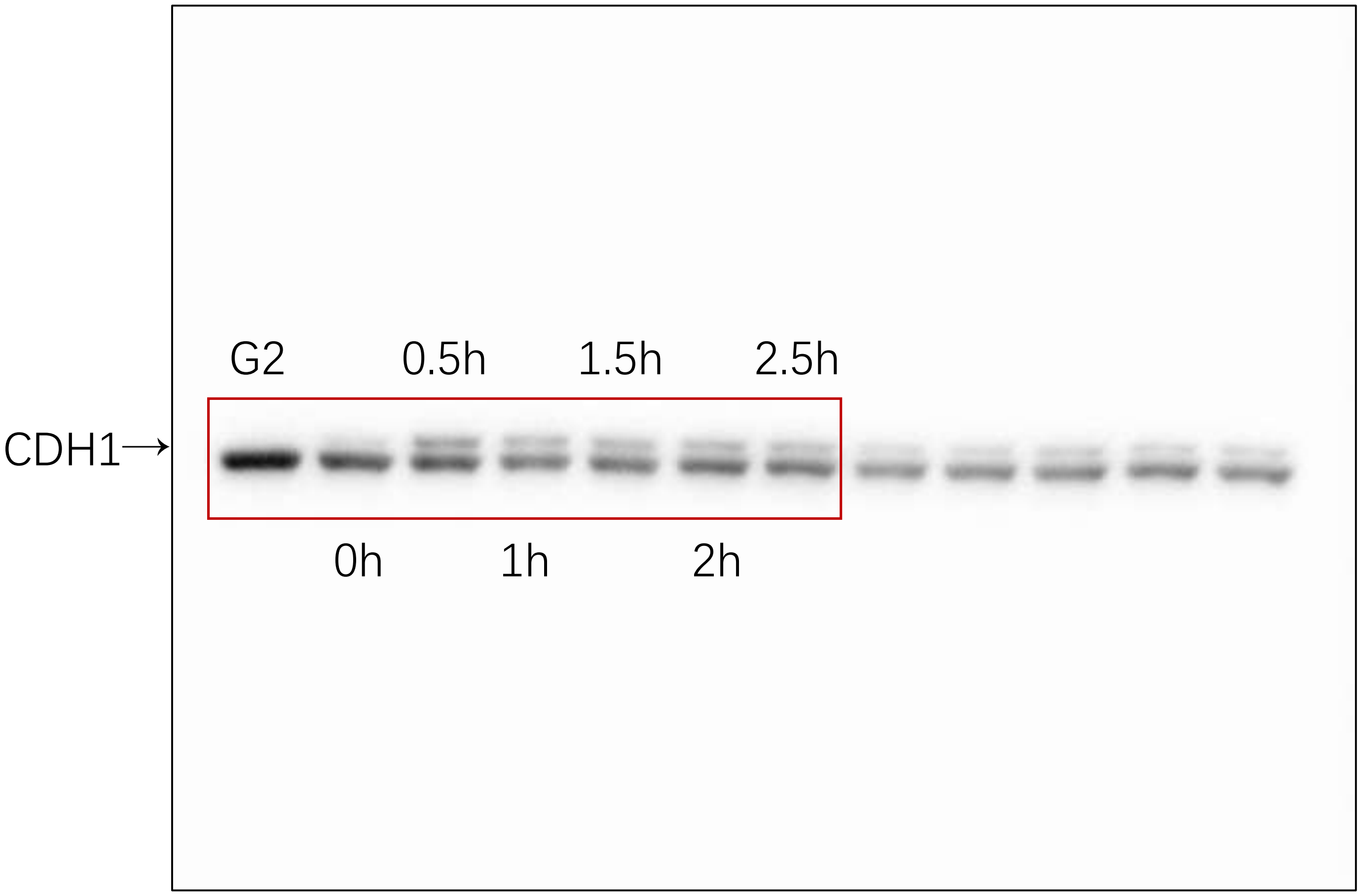

Original images of western blots of SIRT6 and actin in figure 3b 293T

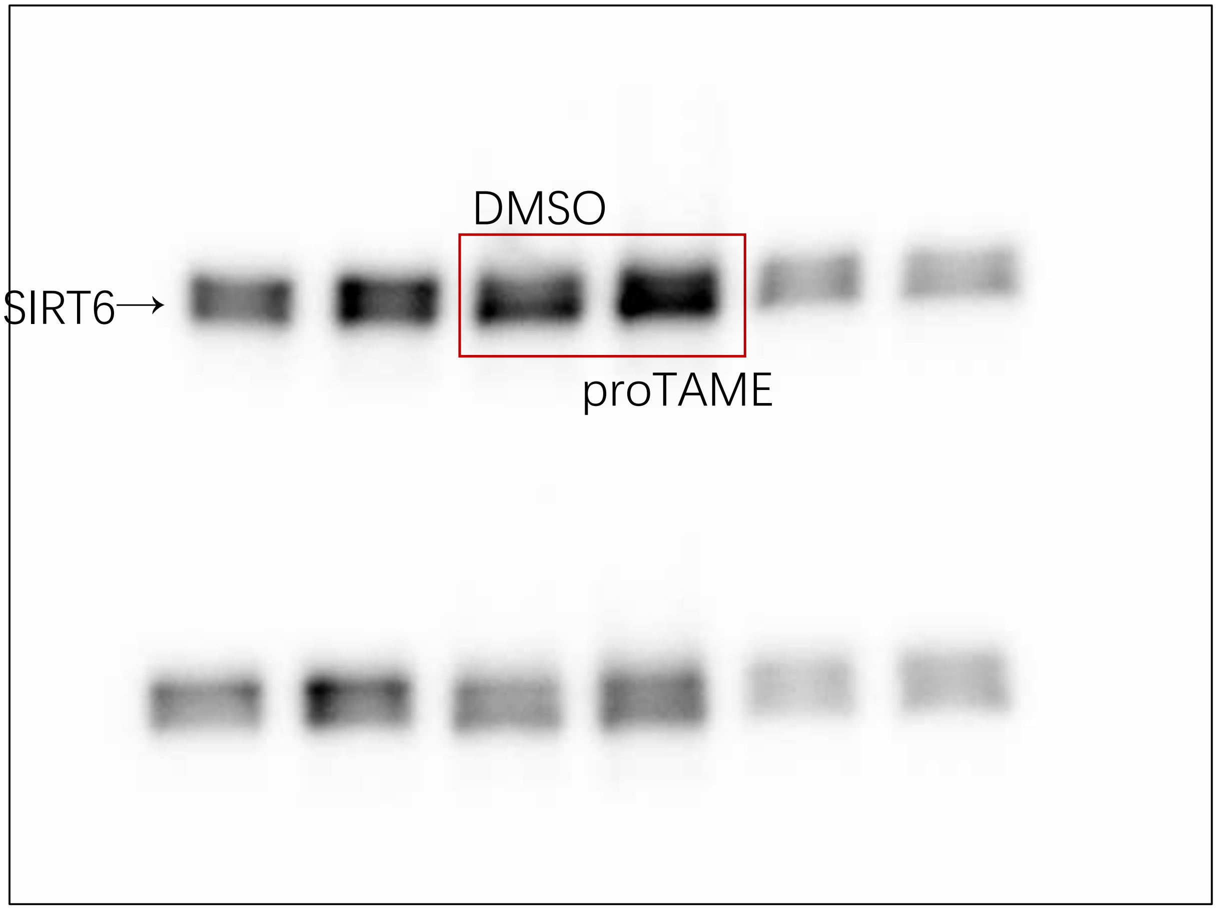

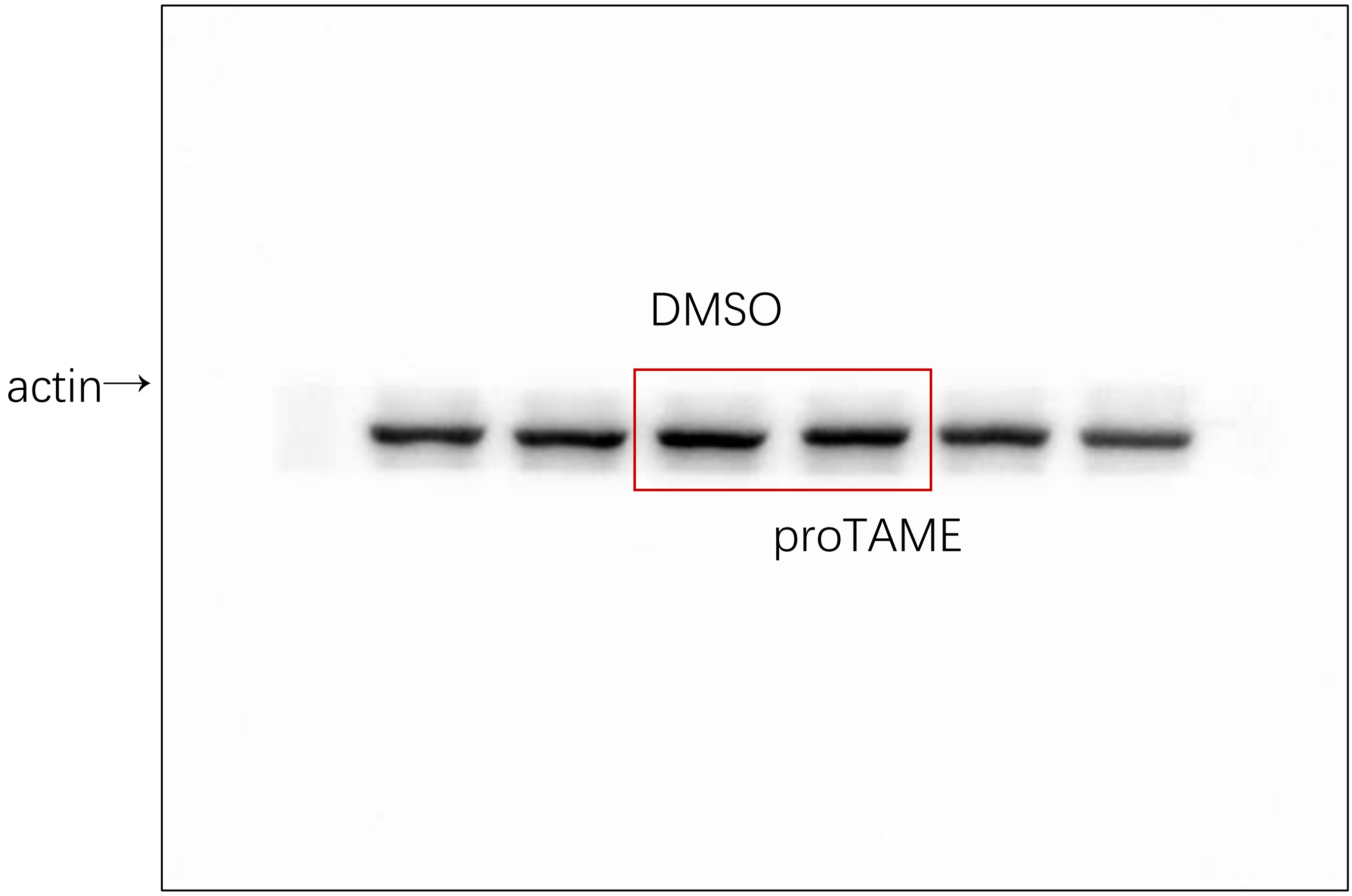

Original images of western blots of SIRT6 and actin in figure 3b HeLa

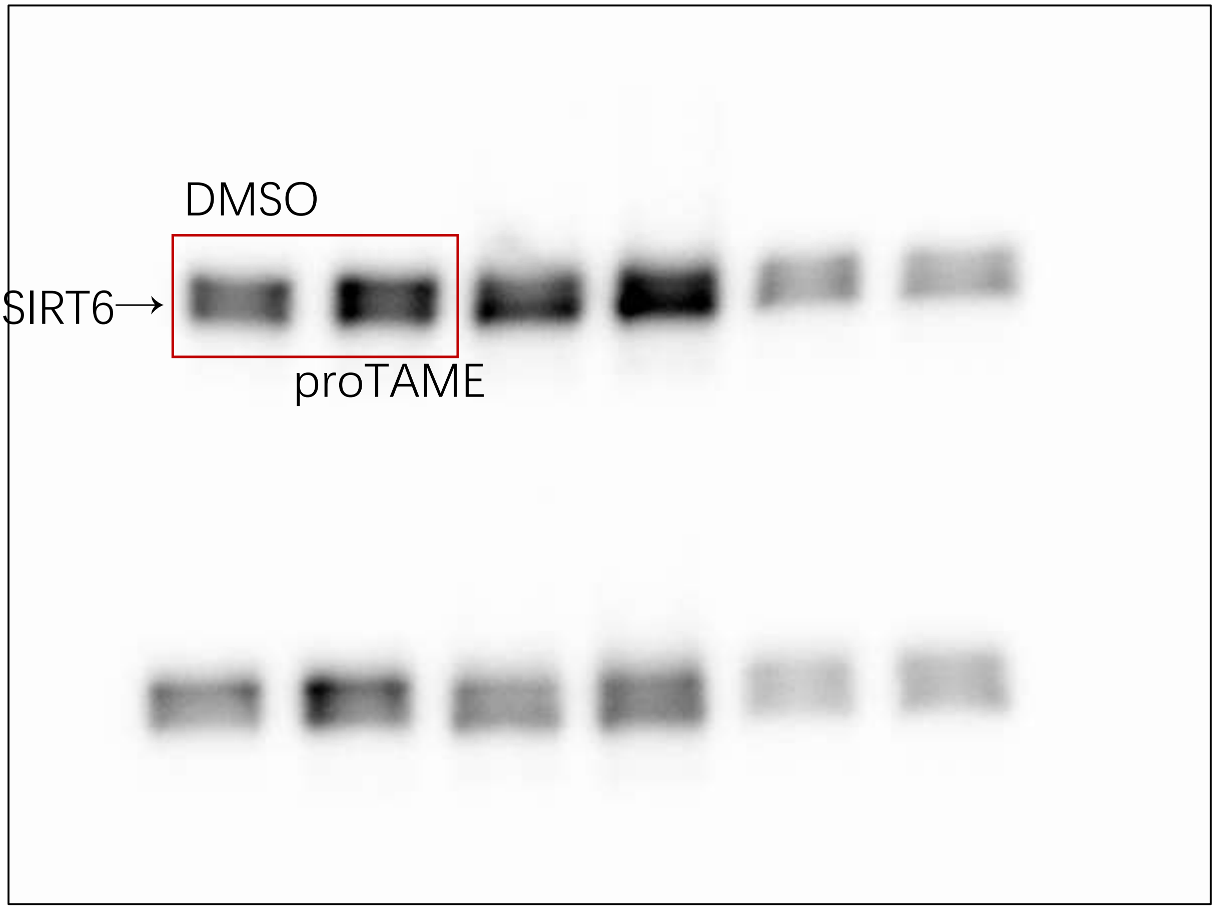

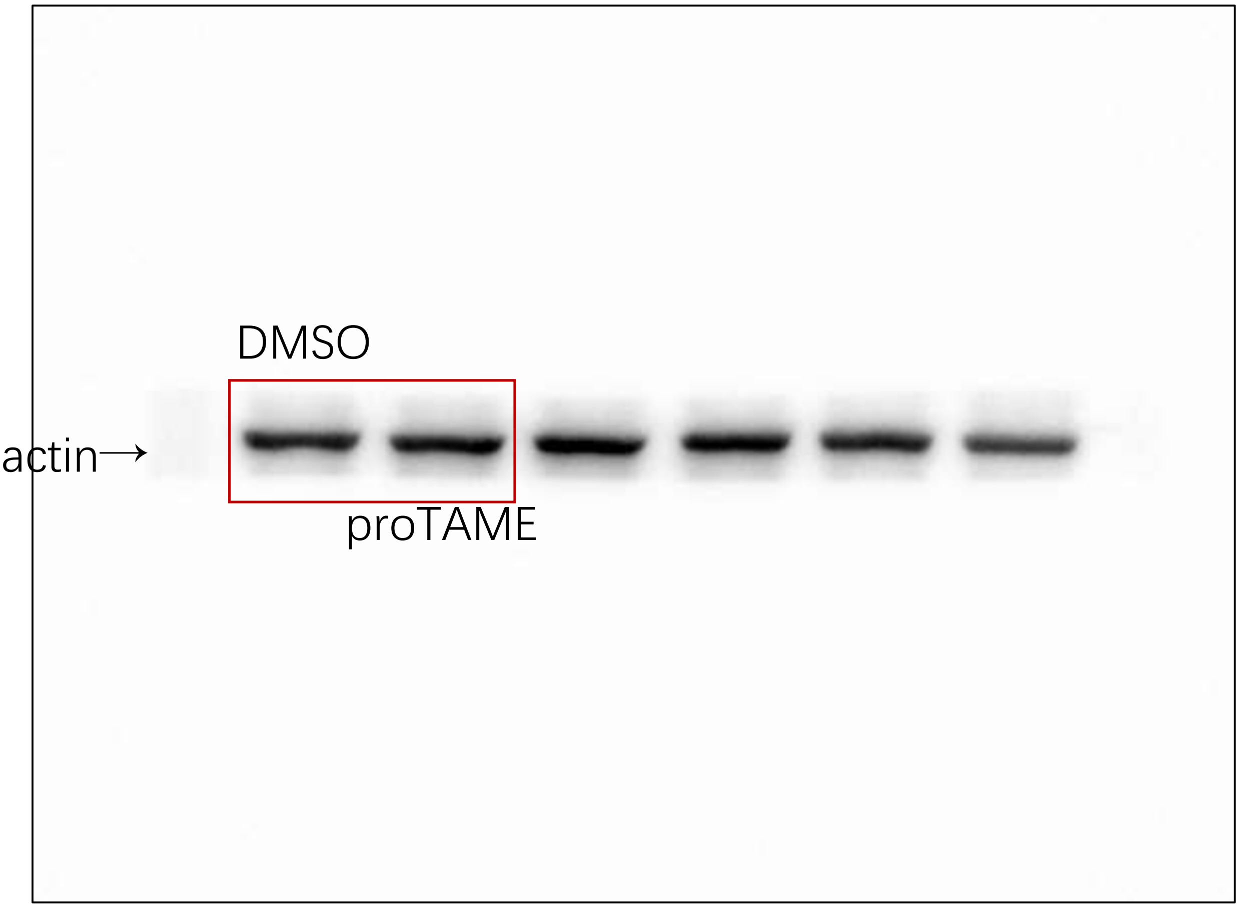

Original images of western blots of SIRT6 and APC1 in figure 3c 293T

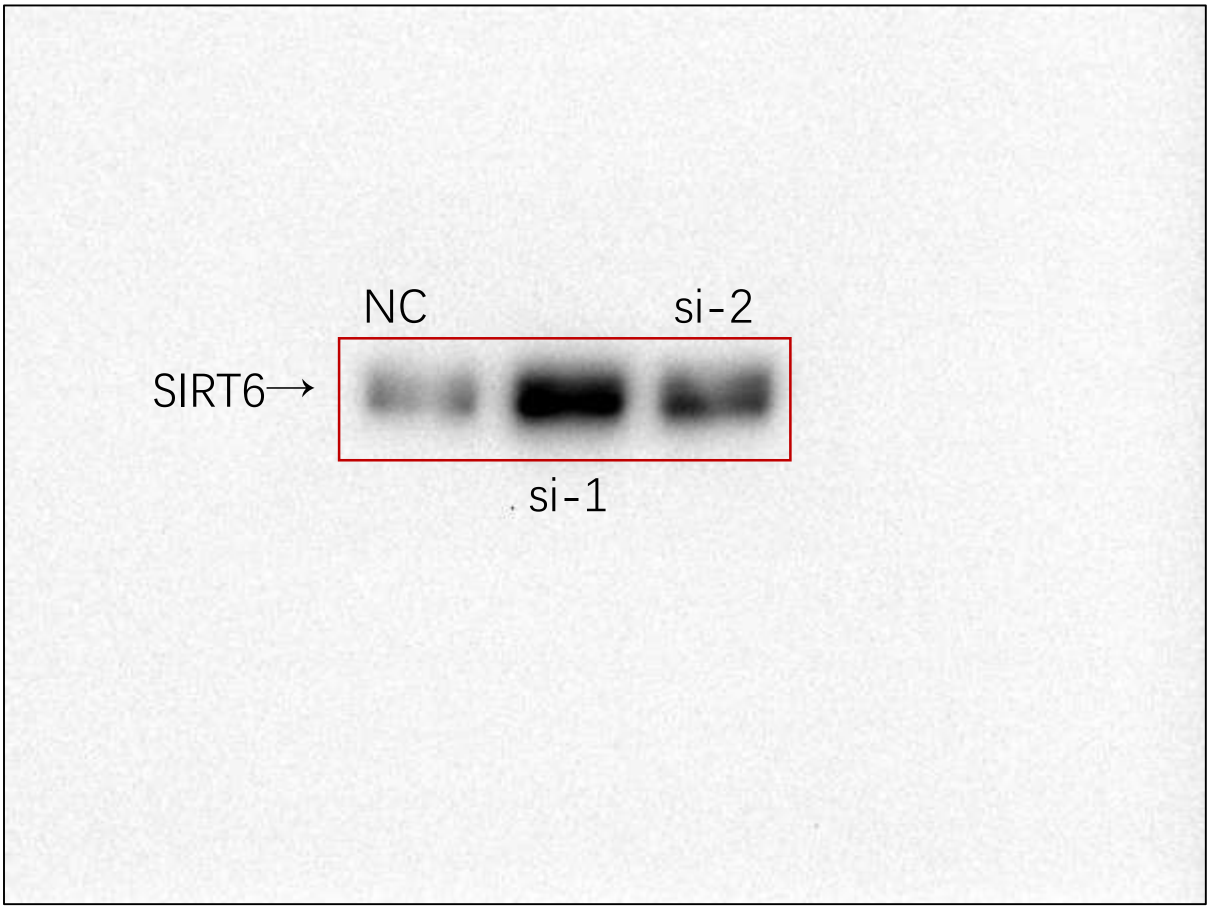

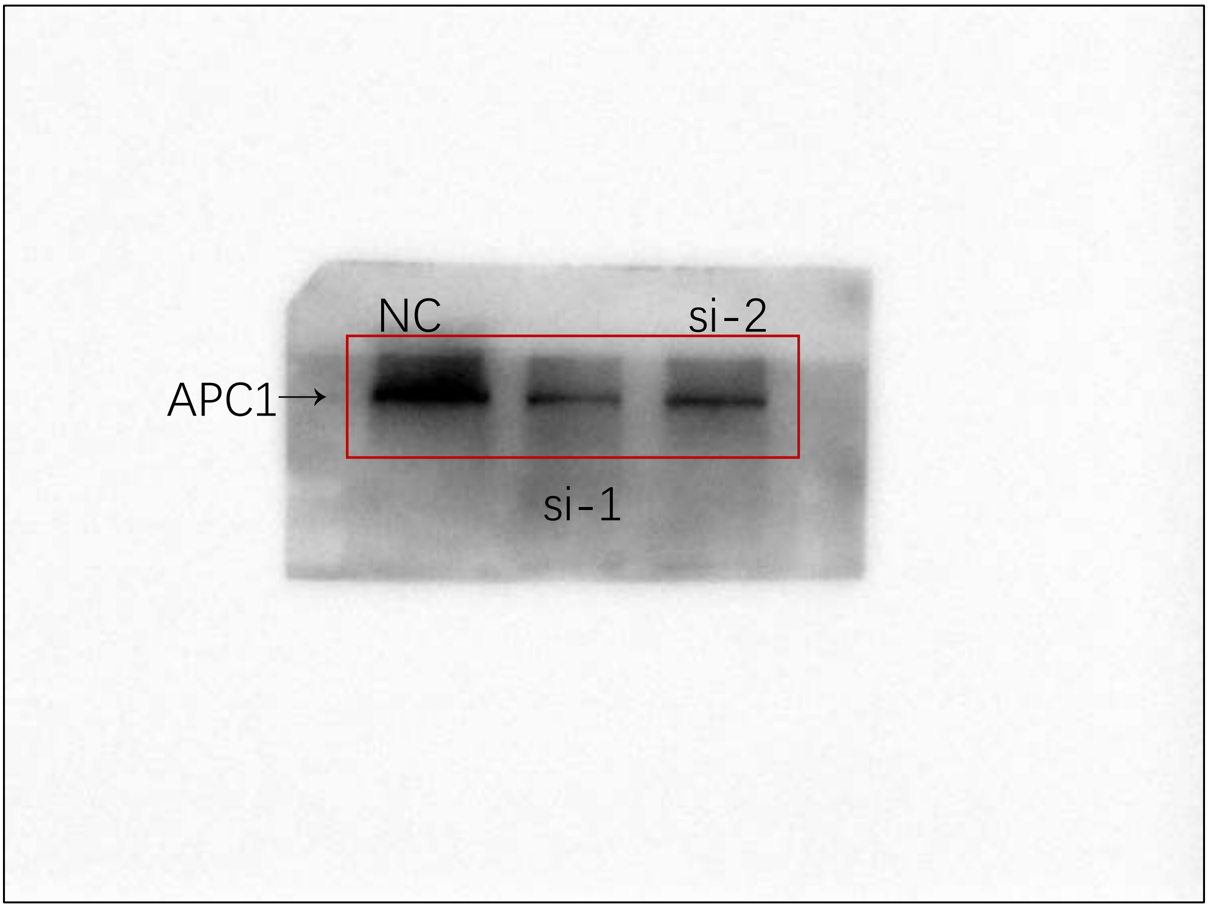

Original images of western blots of actin in figure 3c 293T

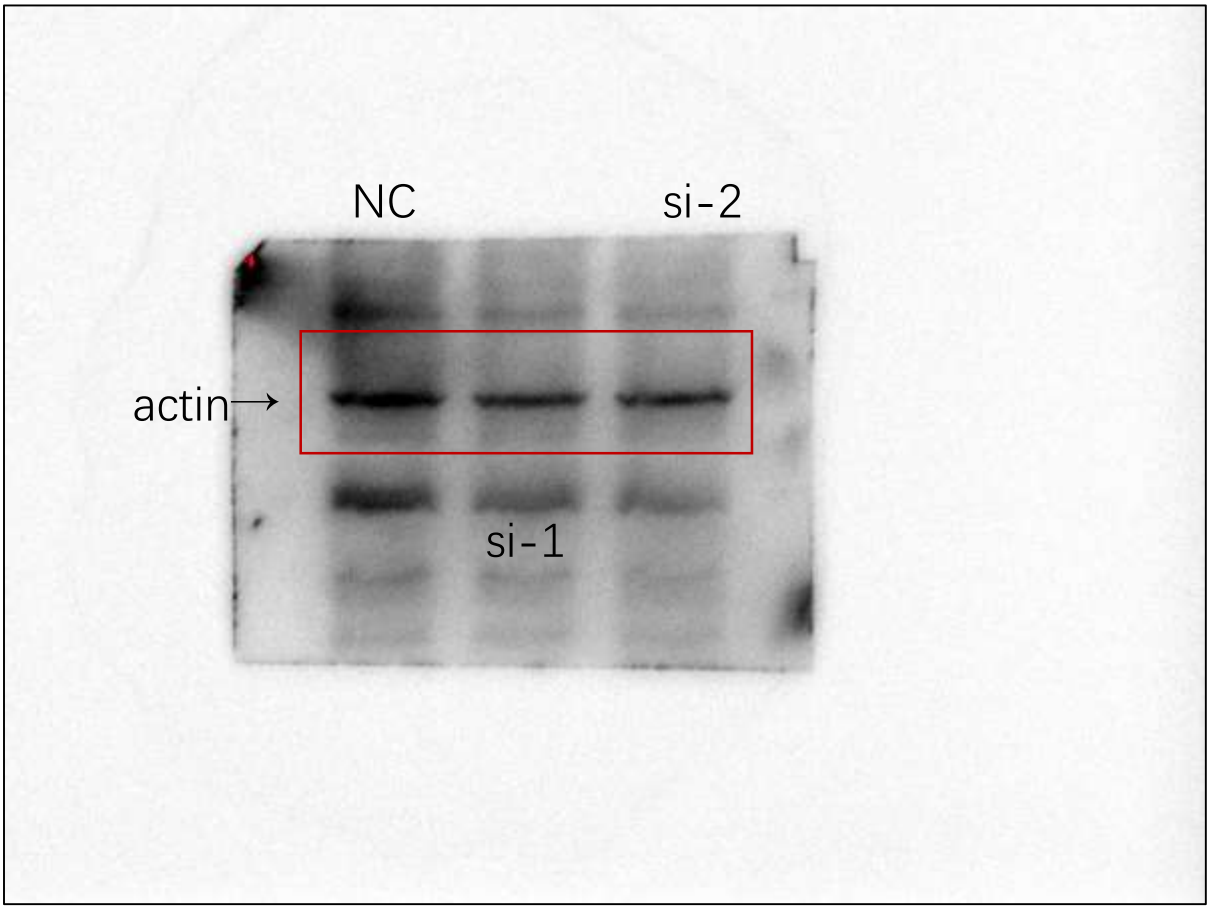

Original images of western blots of SIRT6 and APC1 in figure 3c HeLa

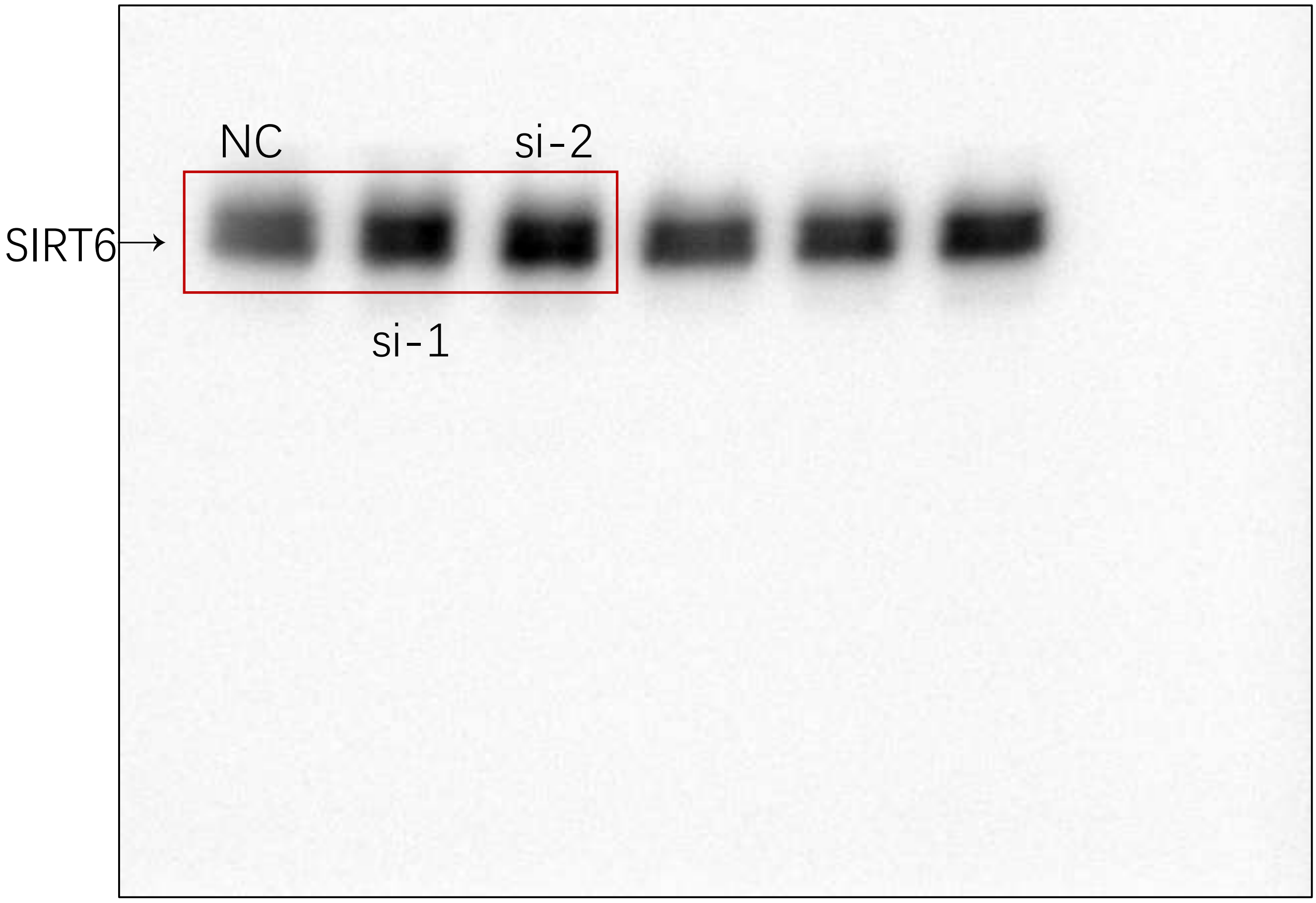

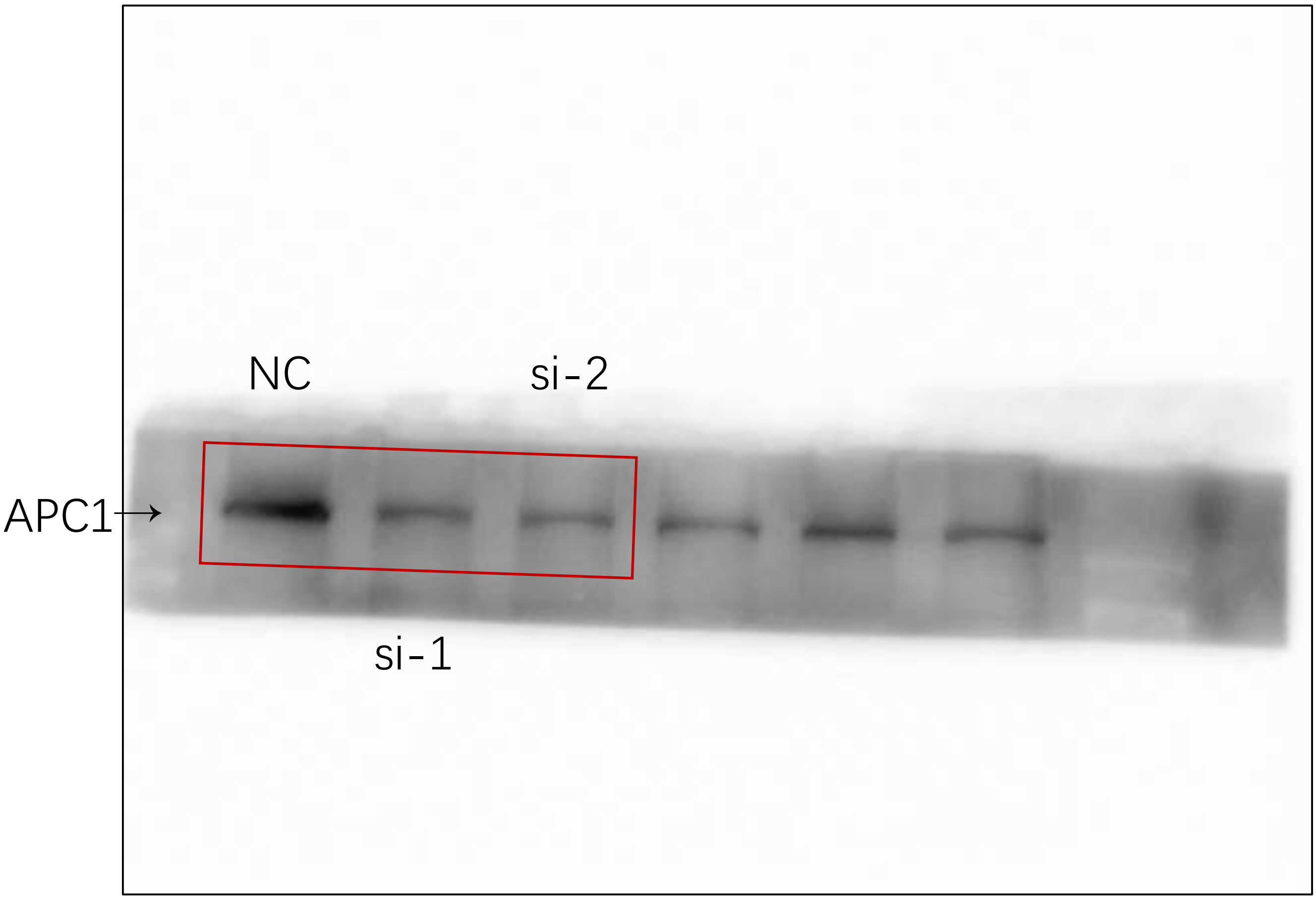

Original images of western blots of actin in figure 3c HeLa

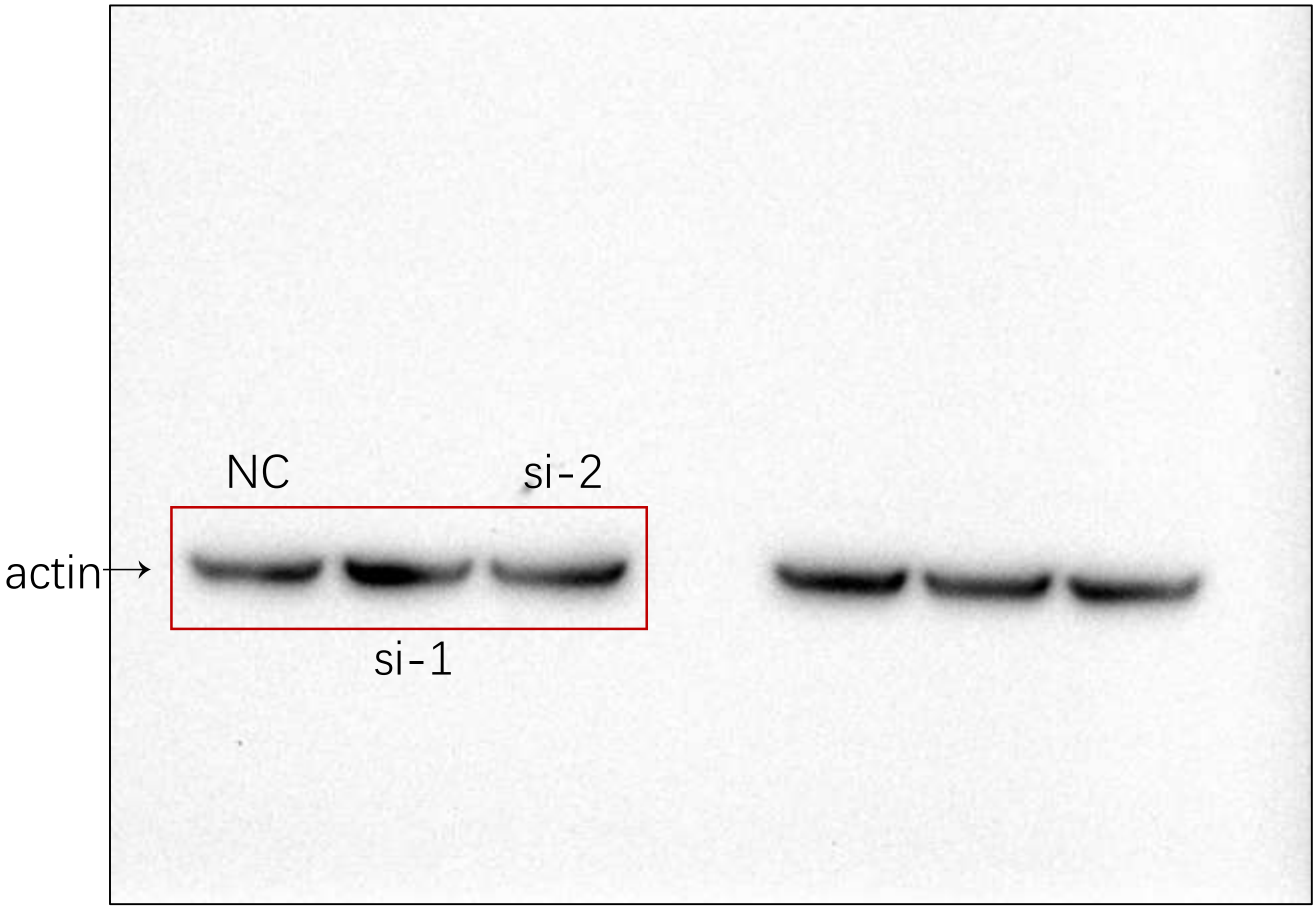

Original images of western blots of SIRT6 and HA in figure 4a 293T

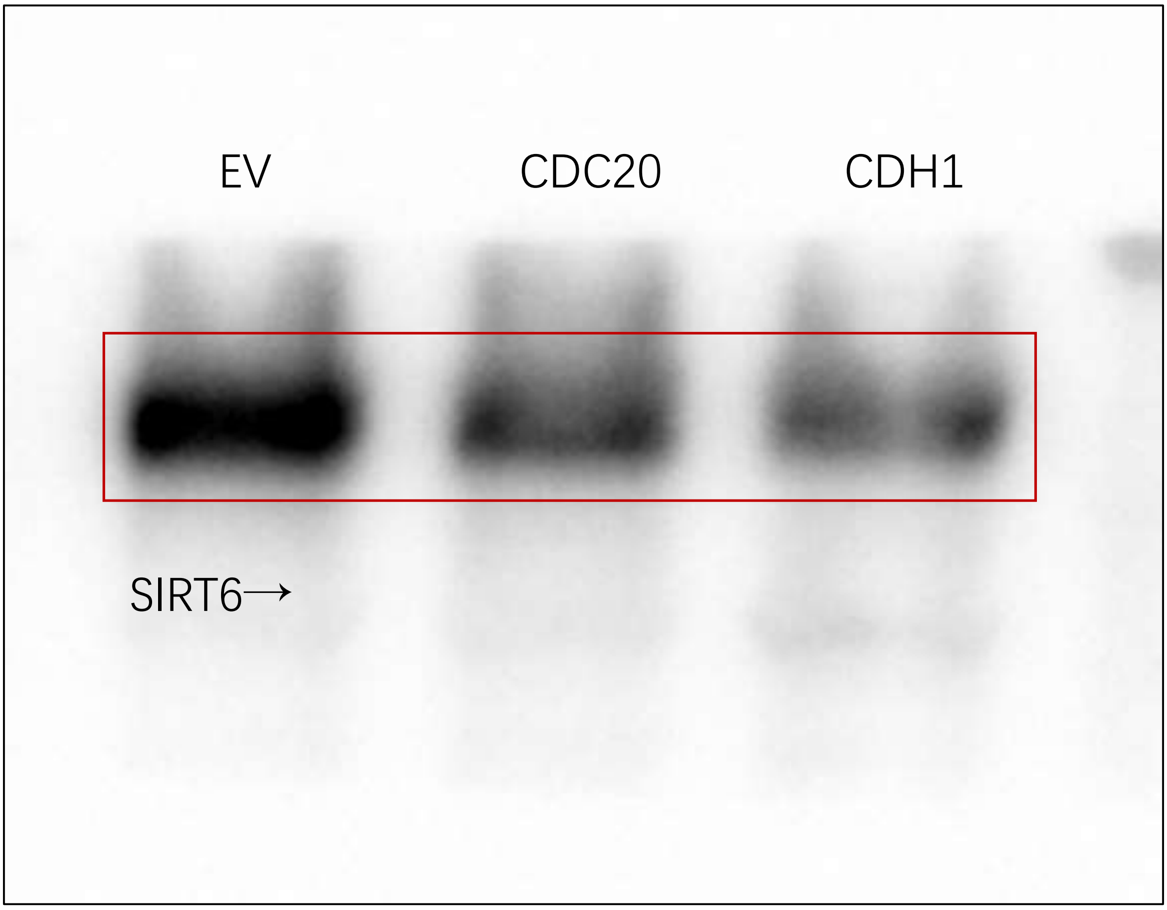

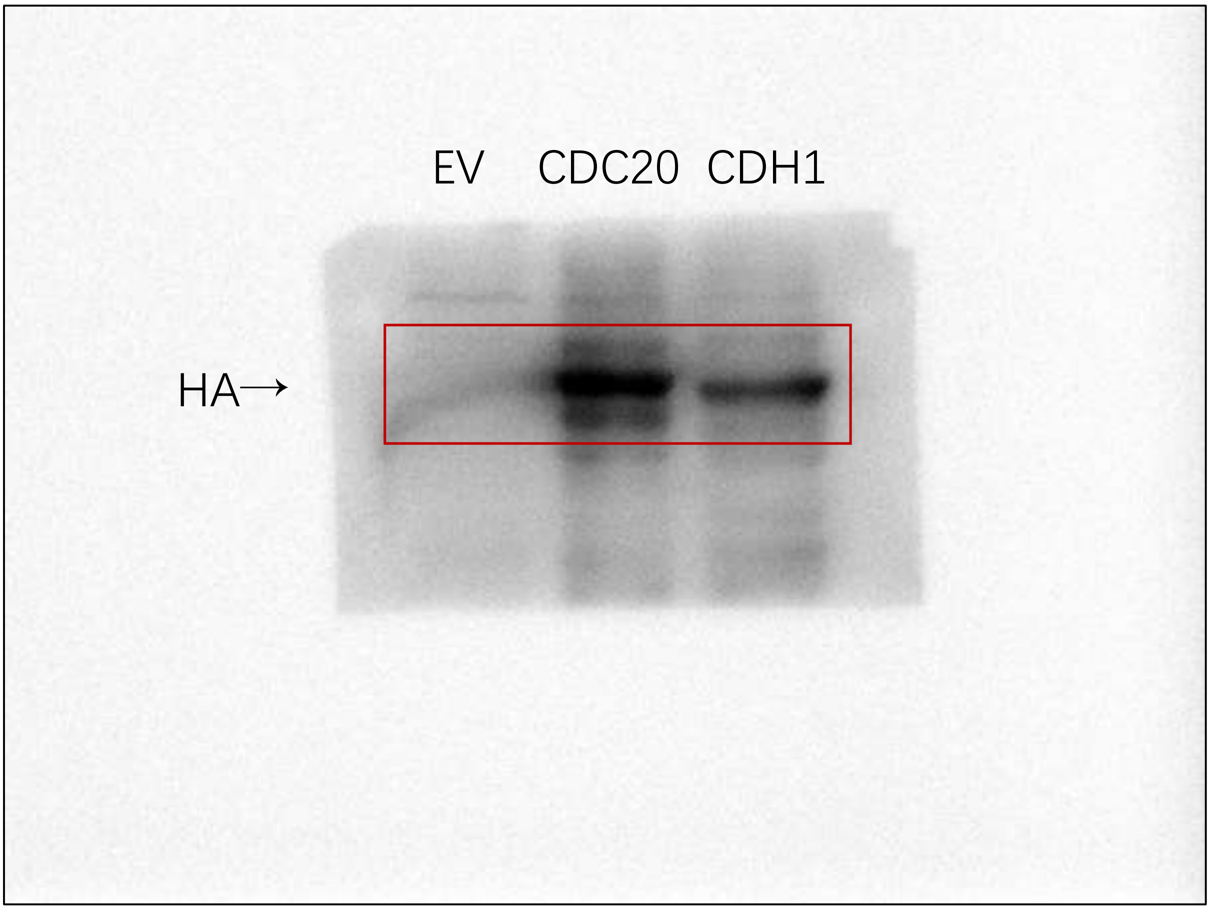

Original images of western blots of GAPDH in figure 4a 293T

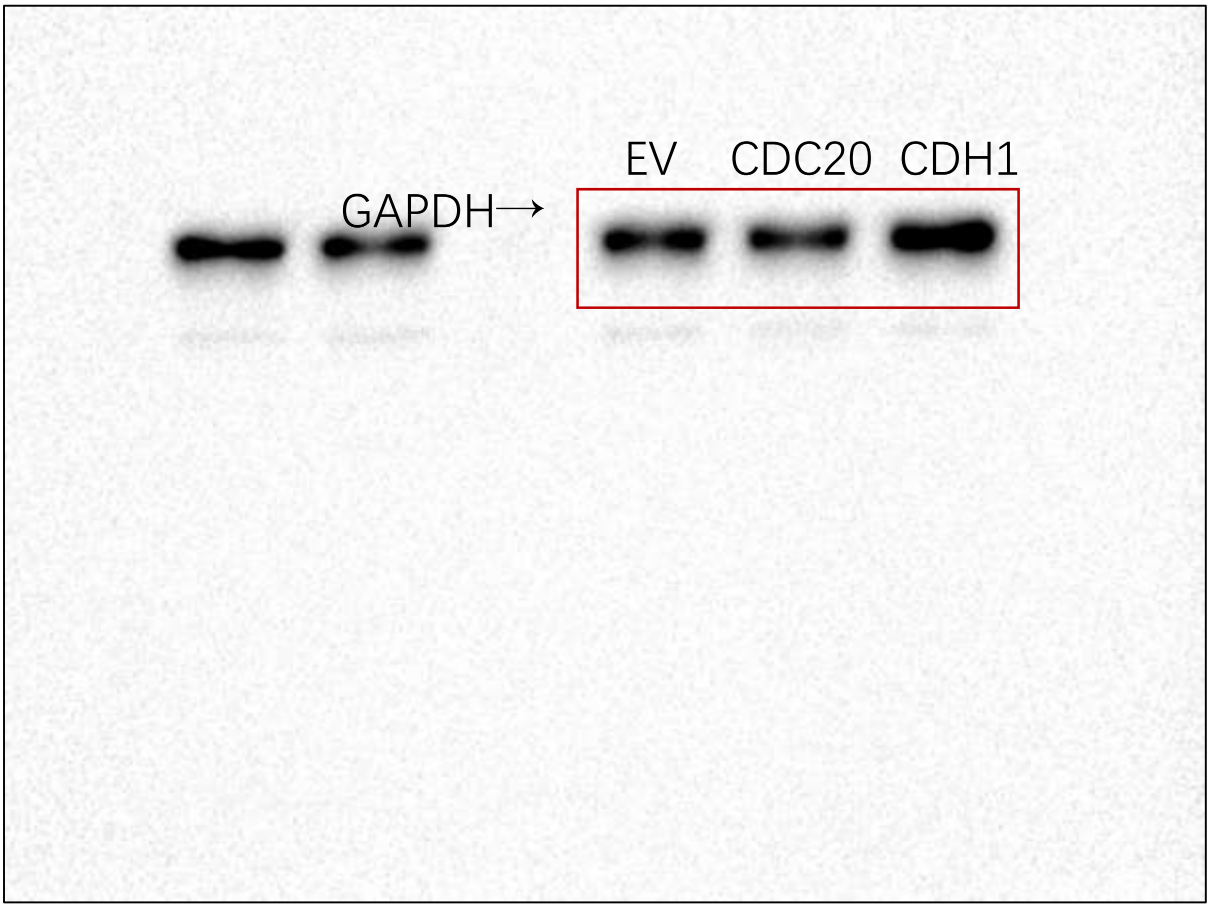

Original images of western blots of SIRT6 and HA in figure 4a HeLa

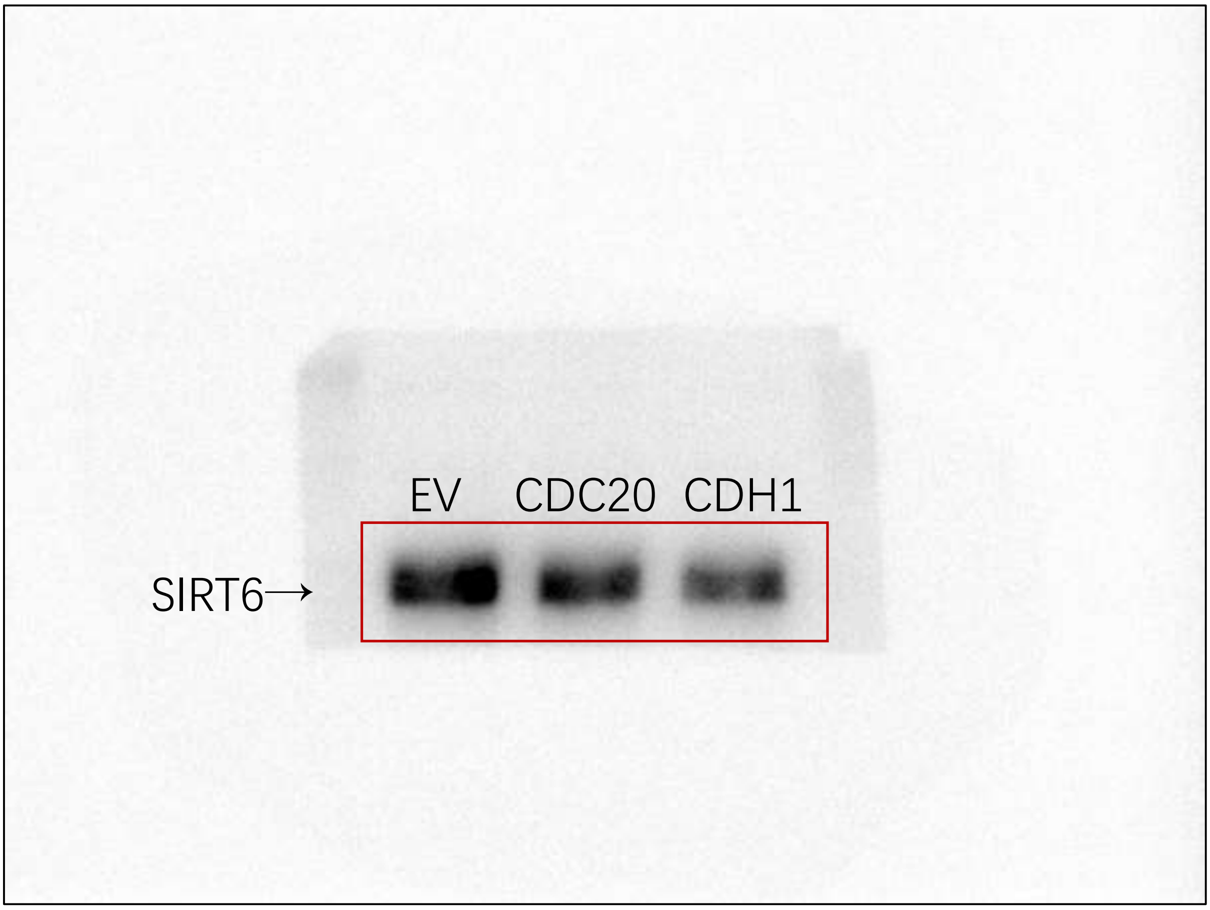

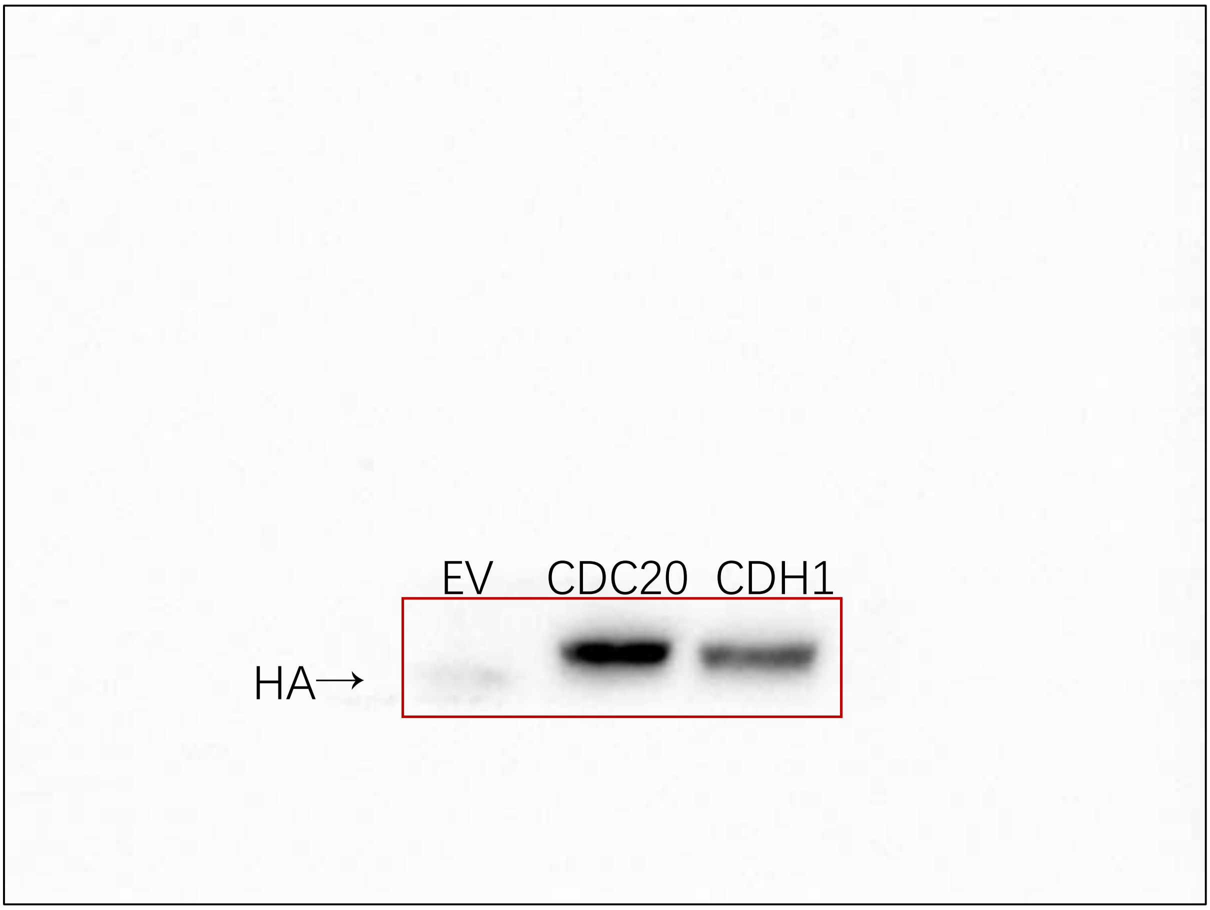

Original images of western blots of actin in figure 4a HeLa

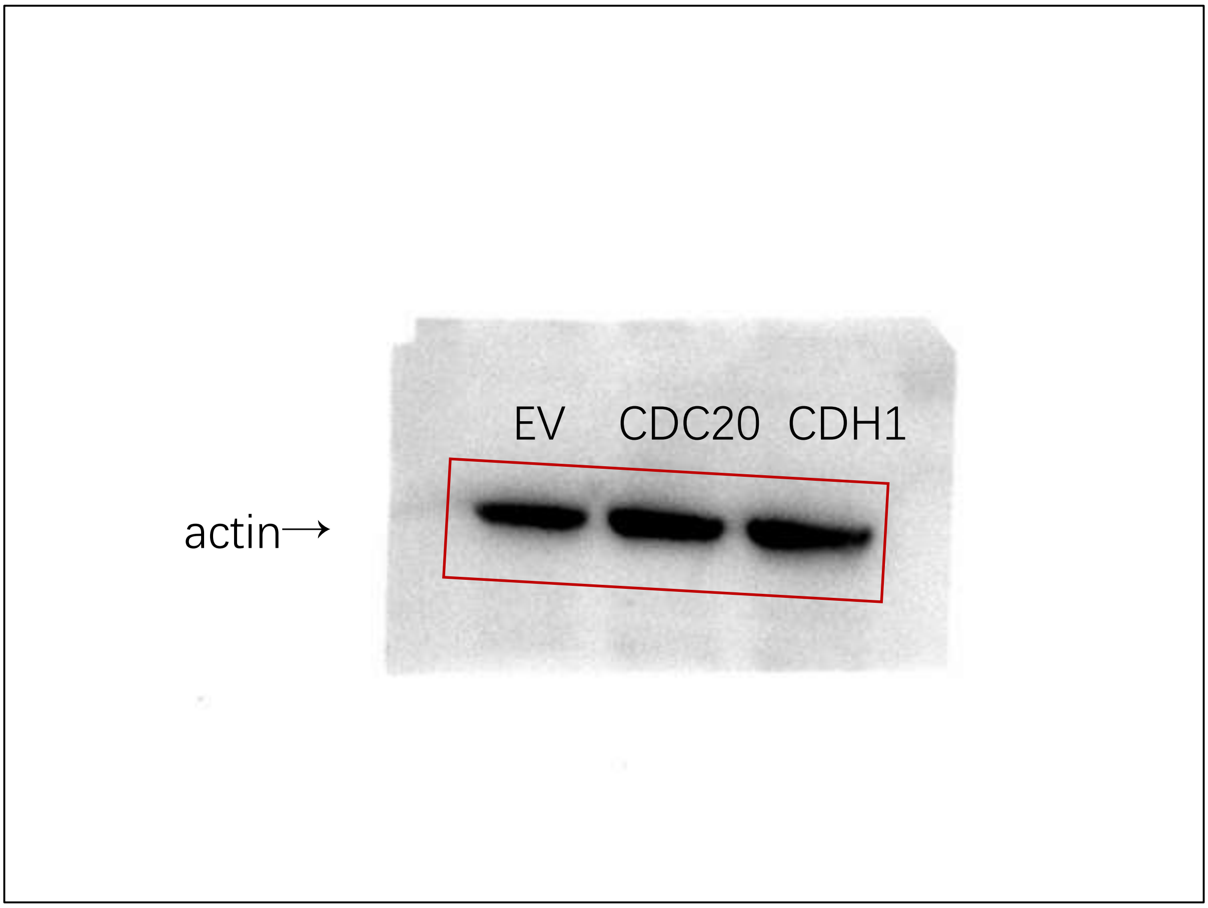

Original images of western blots of SIRT6 in figure 4b 293T EV & CDC20

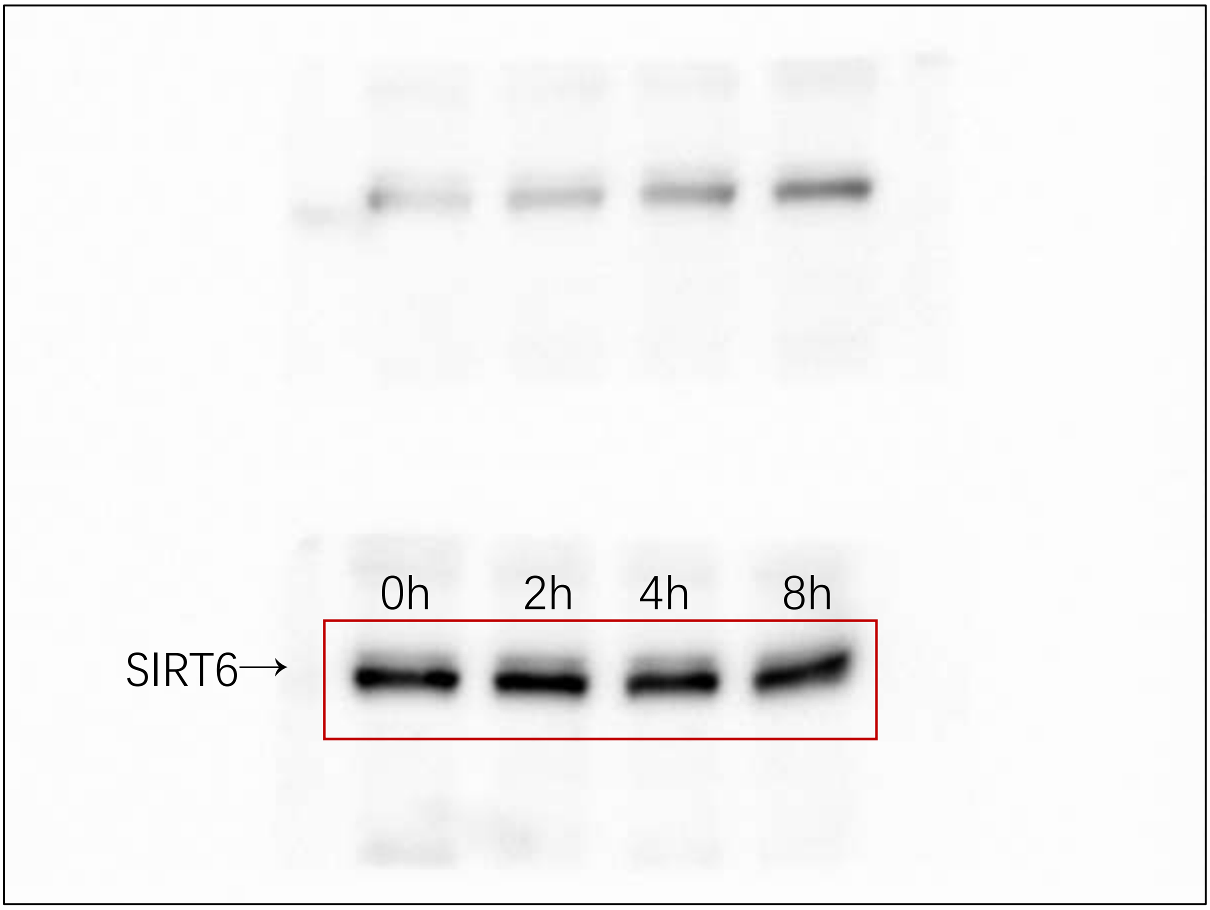

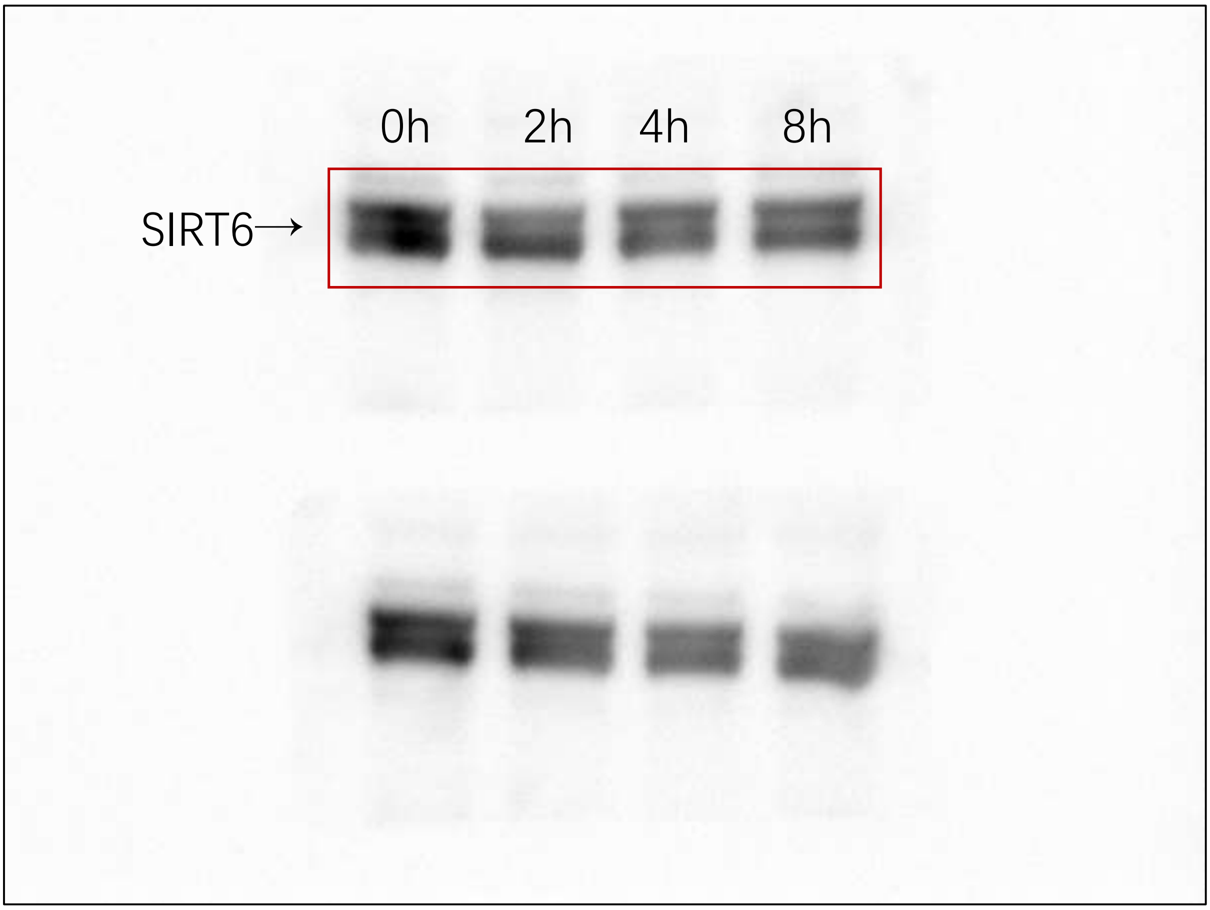

Original images of western blots of SIRT6 in figure 4b 293T CDH1

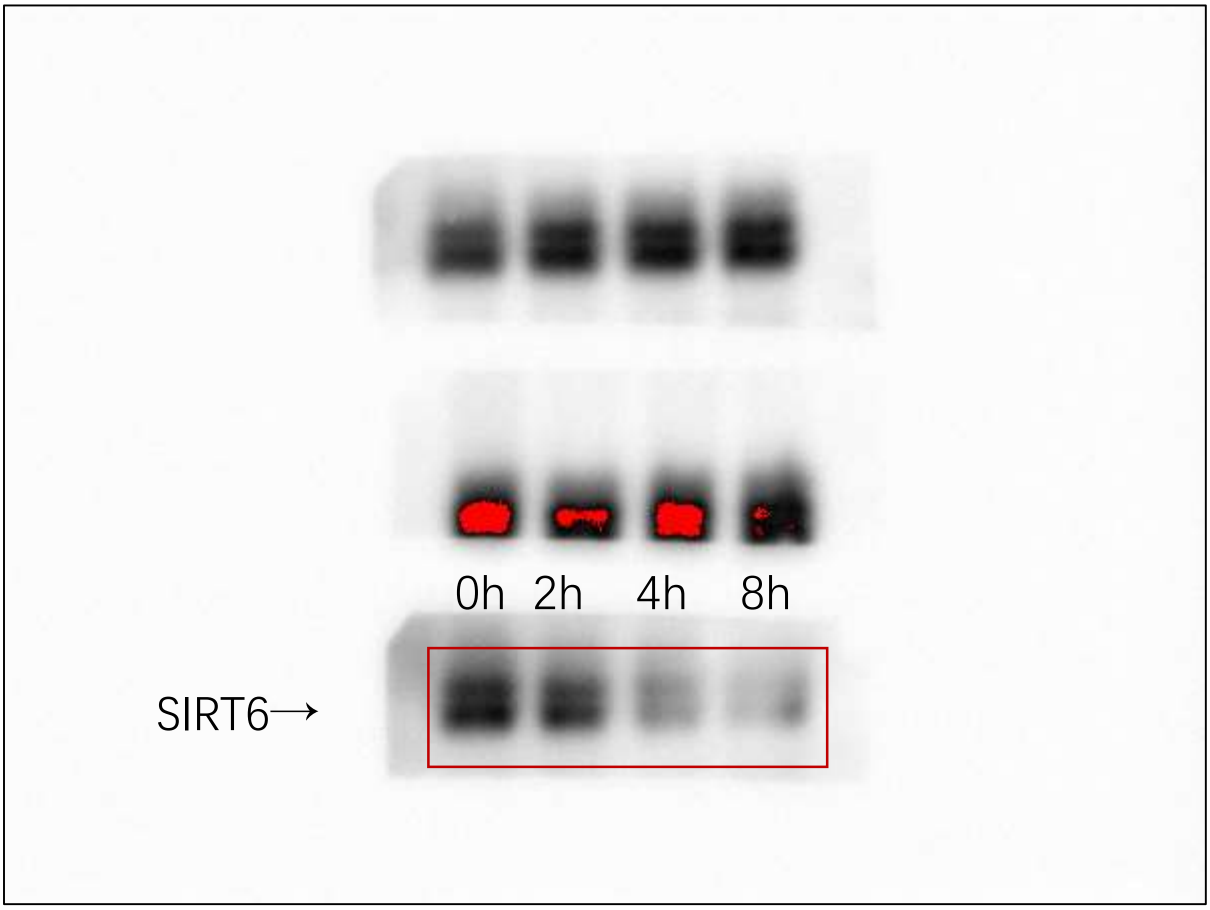

Original images of western blots of actin in figure 4b 293T EV & CDC20

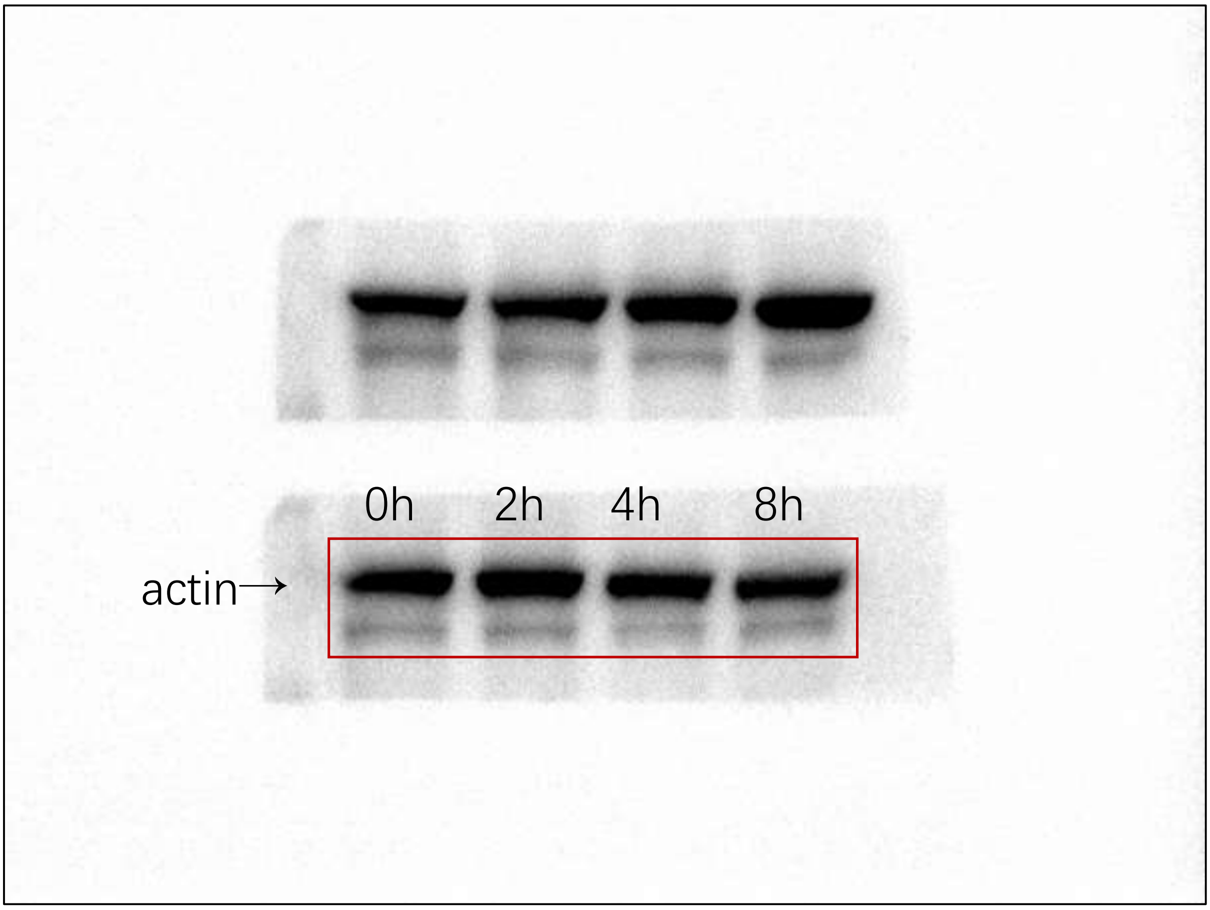

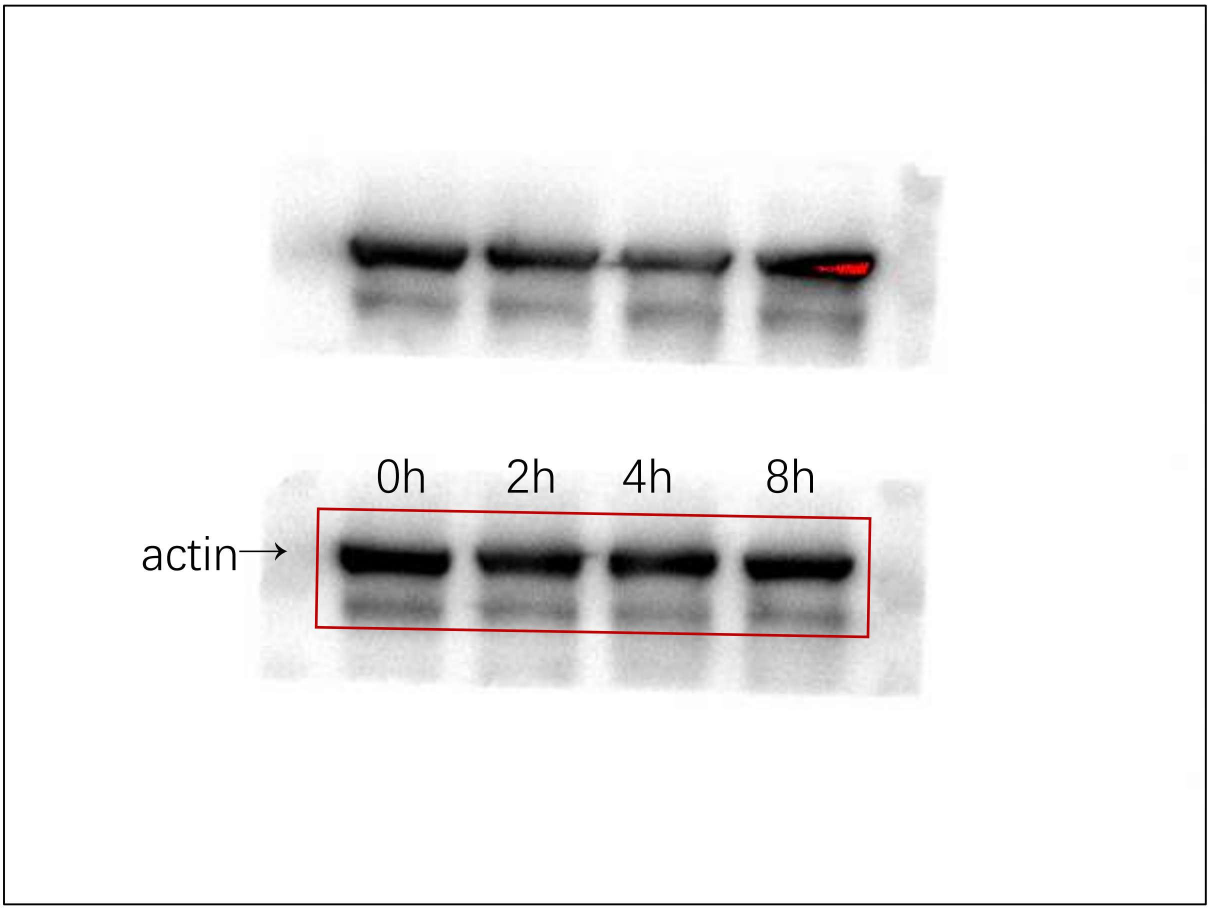

Original images of western blots of actin in figure 4b 293T CDH1

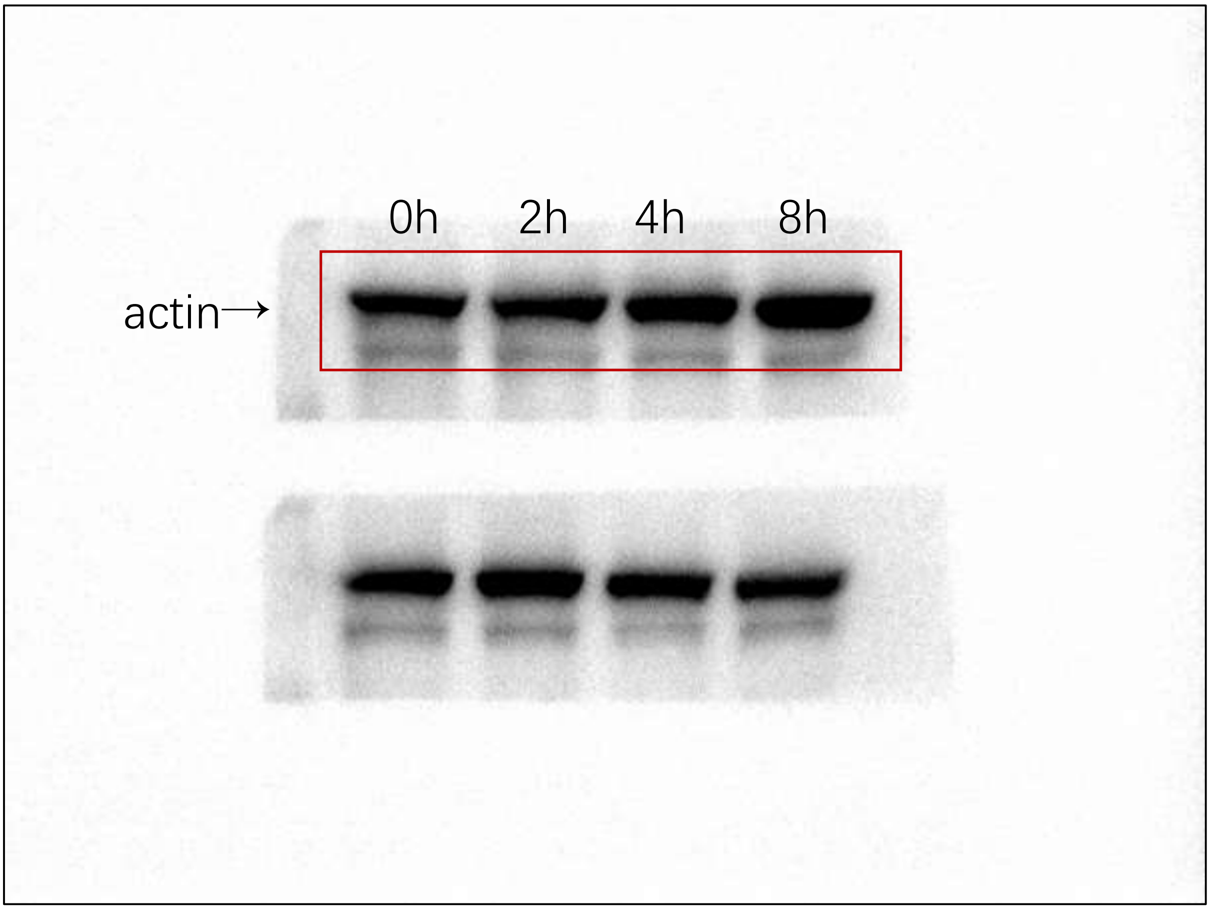

Original images of western blots of SIRT6 in figure 4b HeLa EV & CDC20

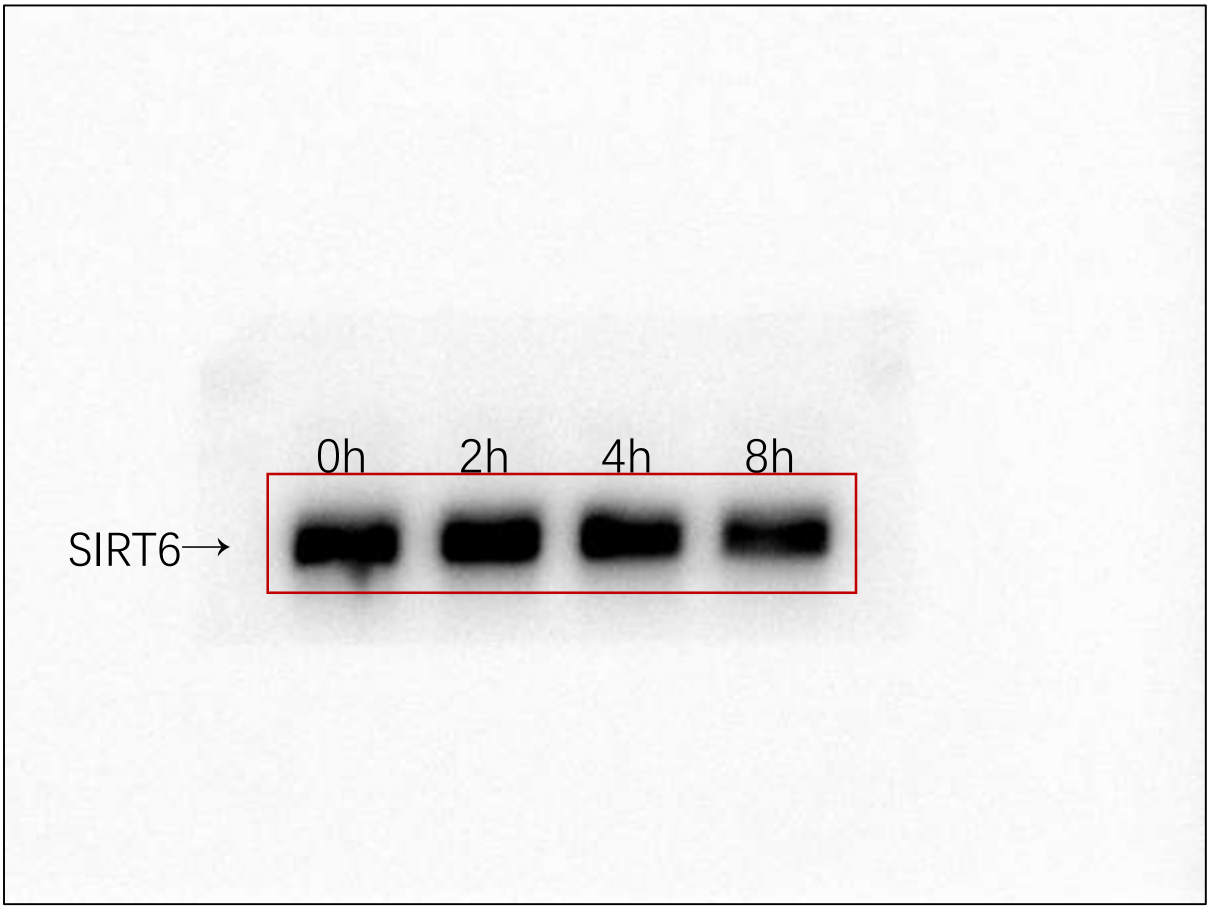

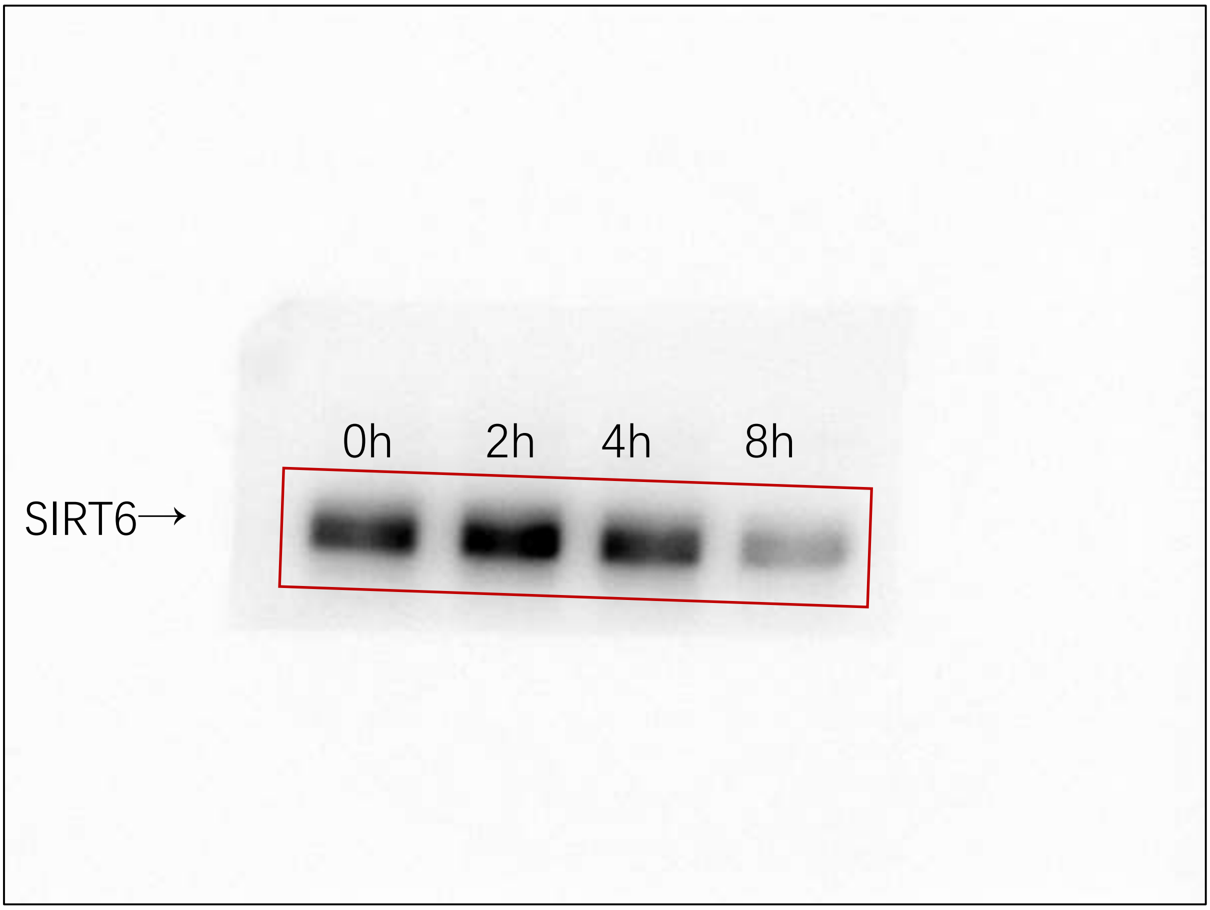

Original images of western blots of SIRT6 in figure 4b HeLa CDH1

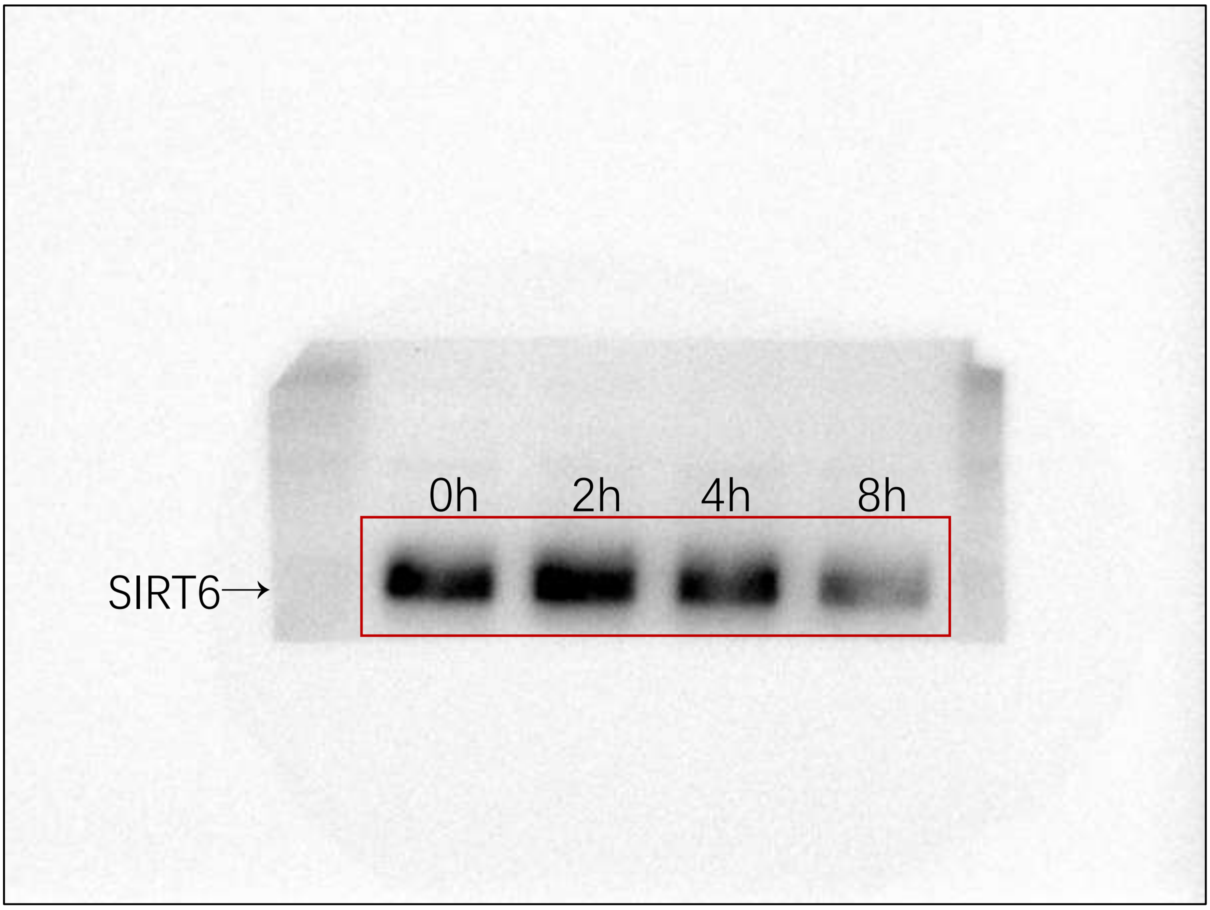

Original images of western blots of actin in figure 4b HeLa EV & CDC20

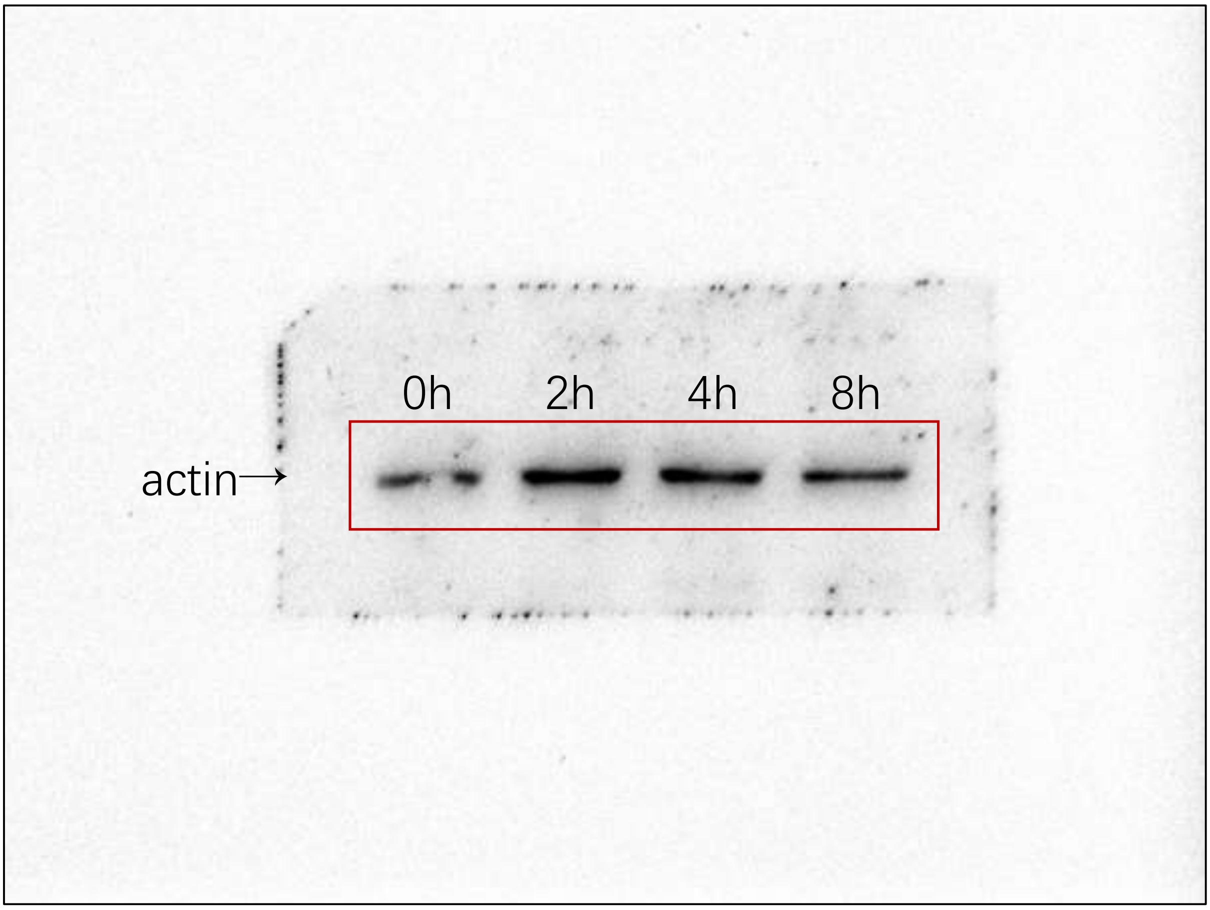

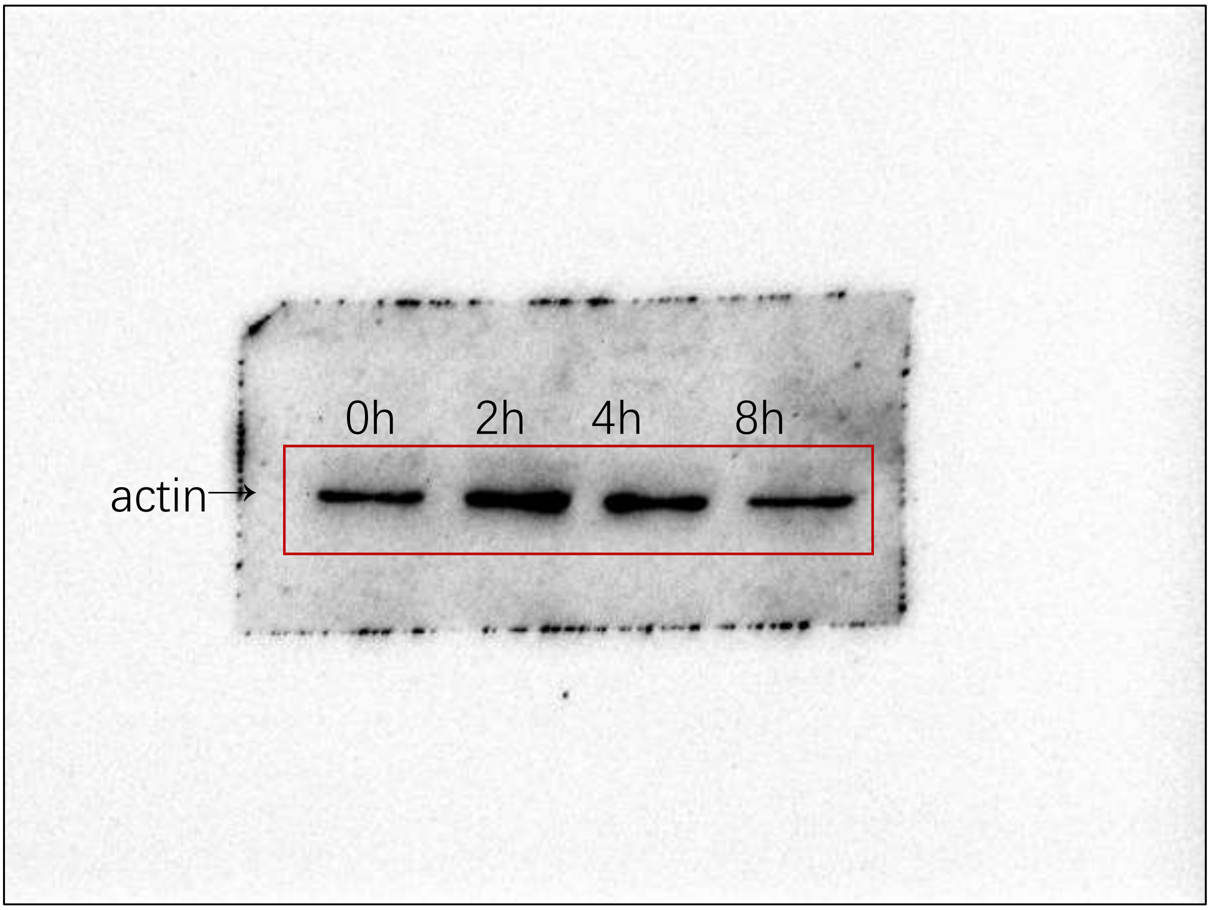

Original images of western blots of actin in figure 4b HeLa CDH1

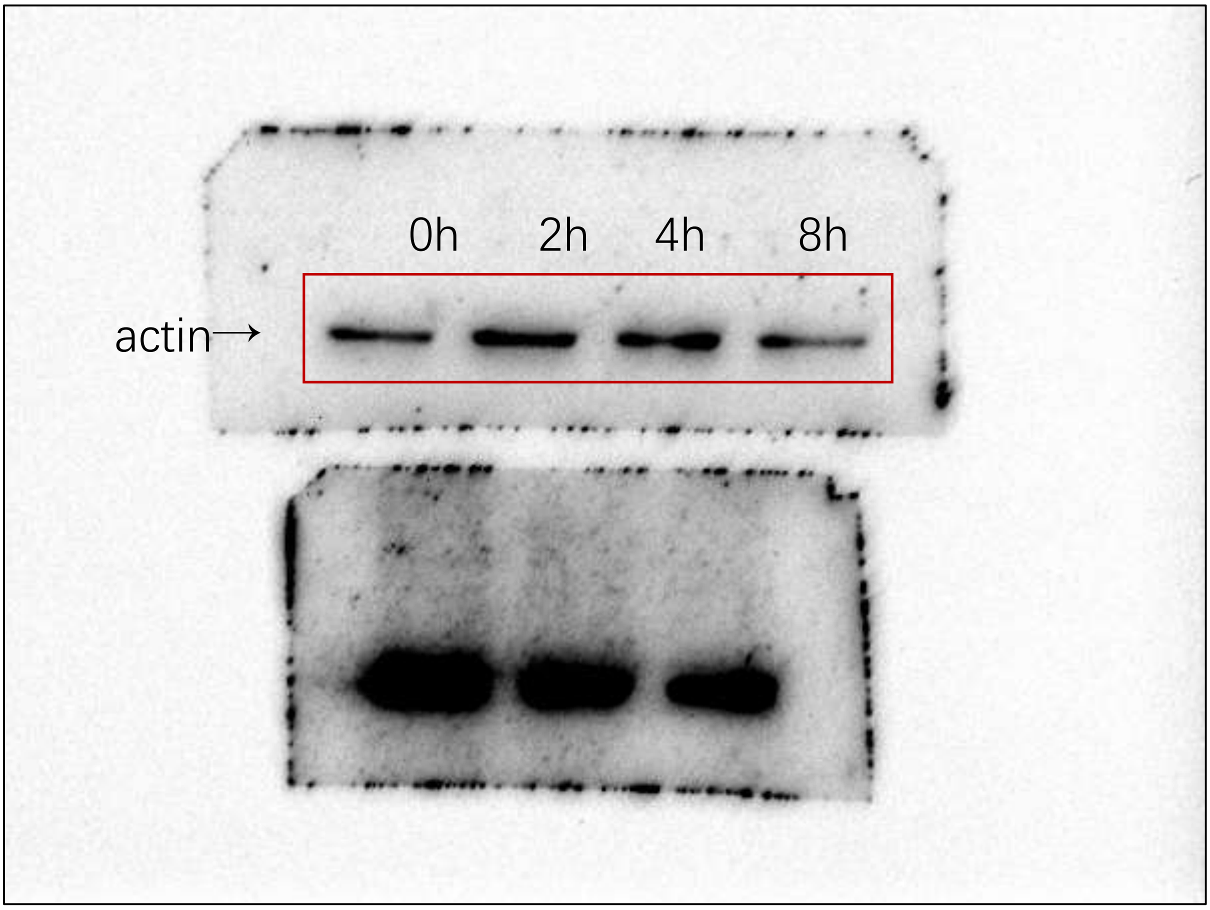

Original images of western blots of Ub & HA in figure 4c

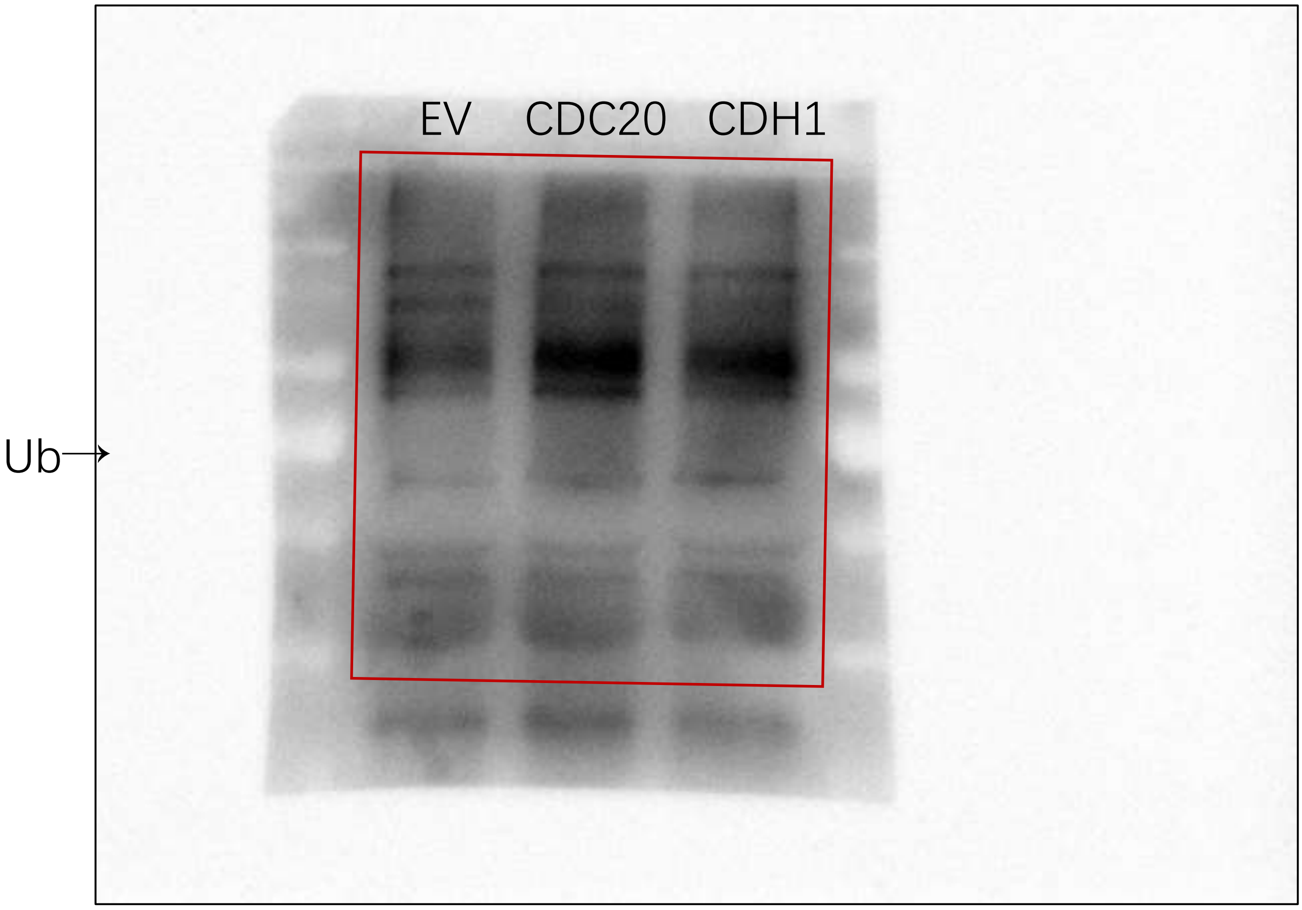

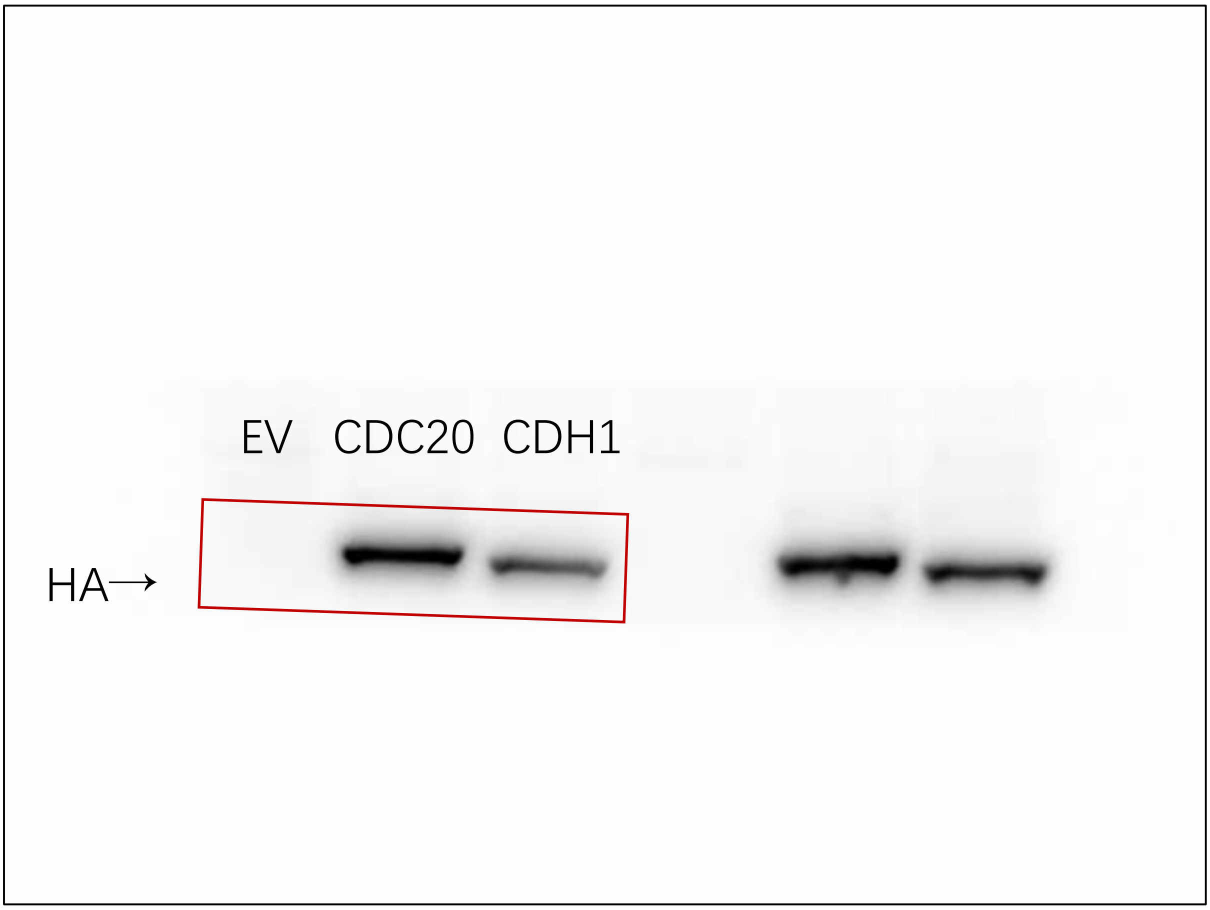

Original images of western blots of FLAG & actin in figure 4c

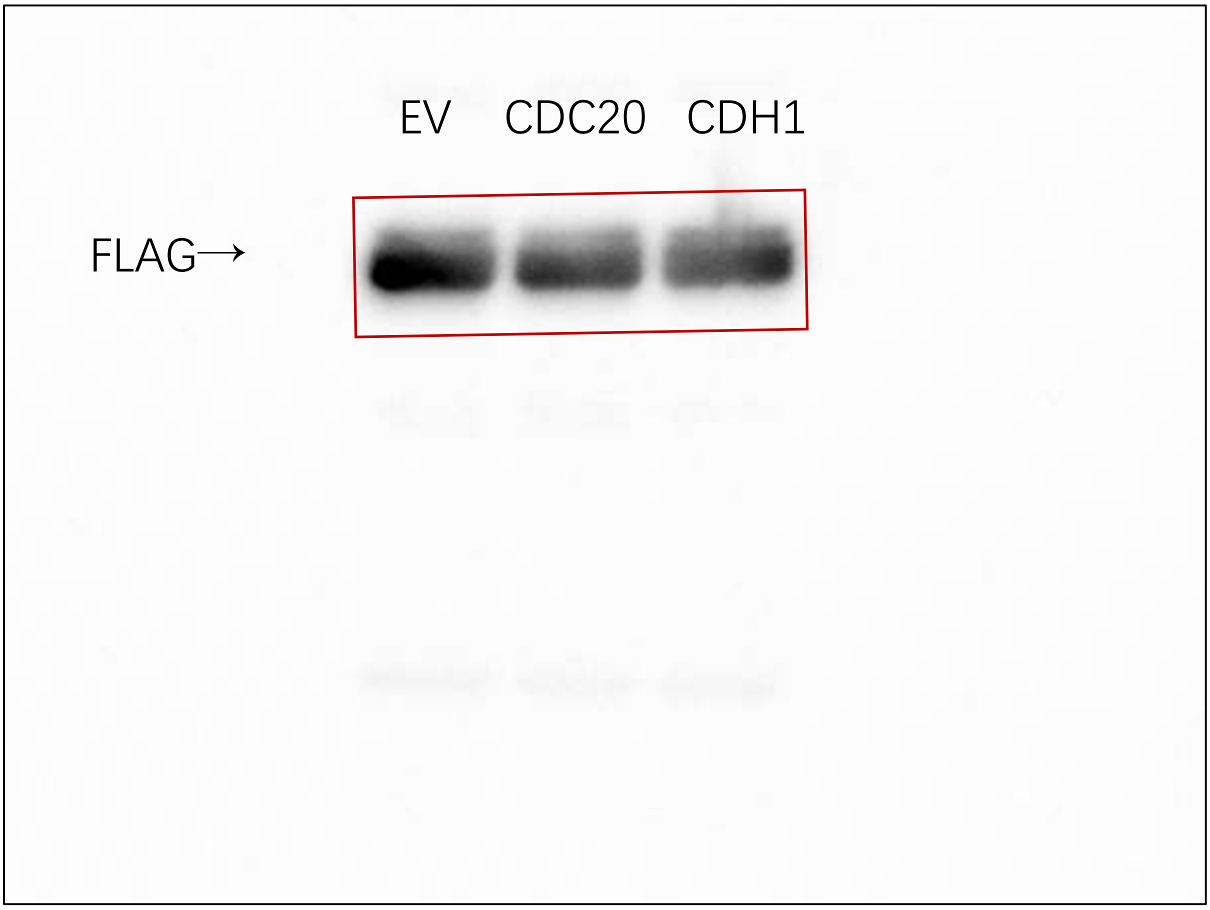

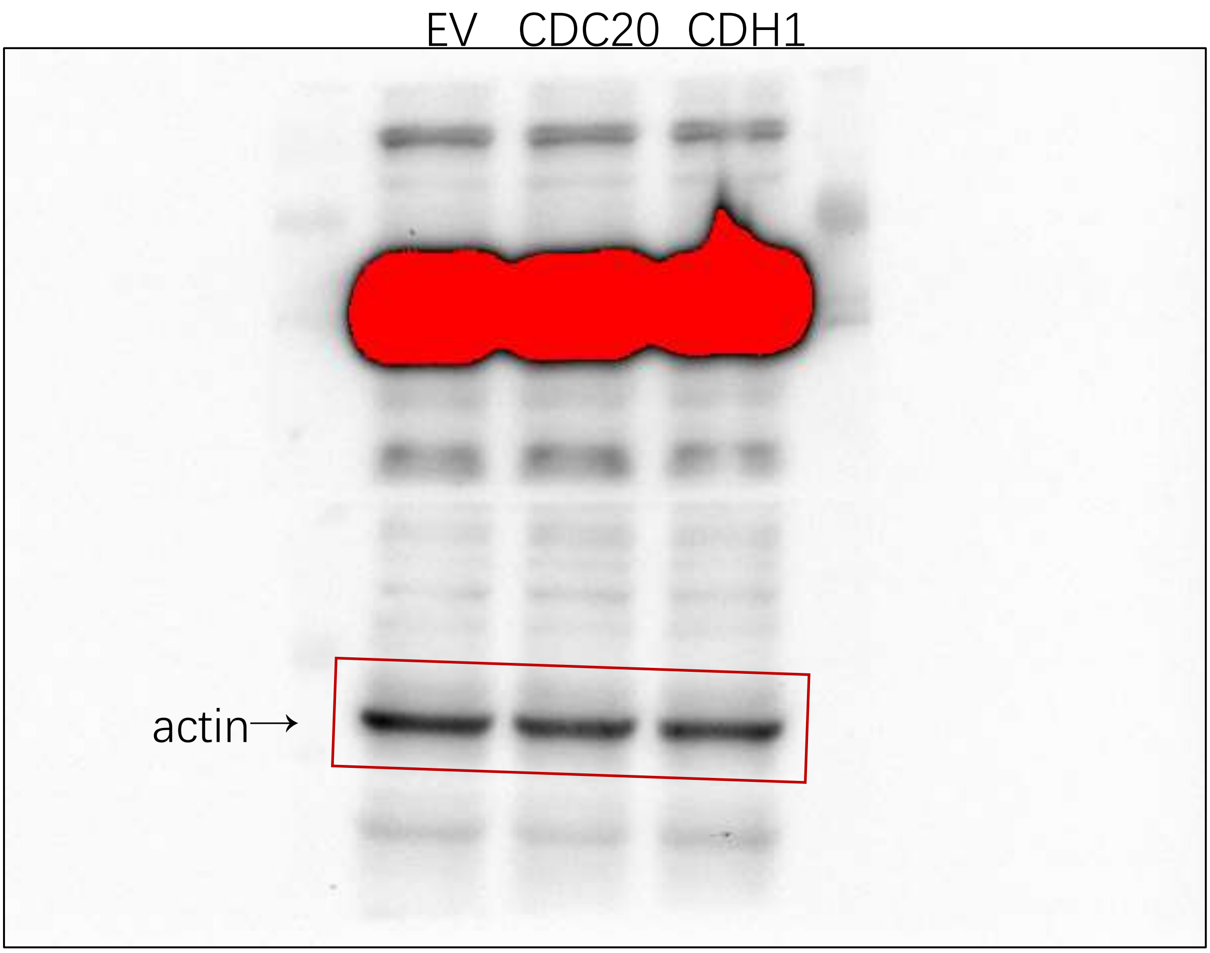

Original images of western blots of WT FLAG in figure 4e CDC20 & CDH1

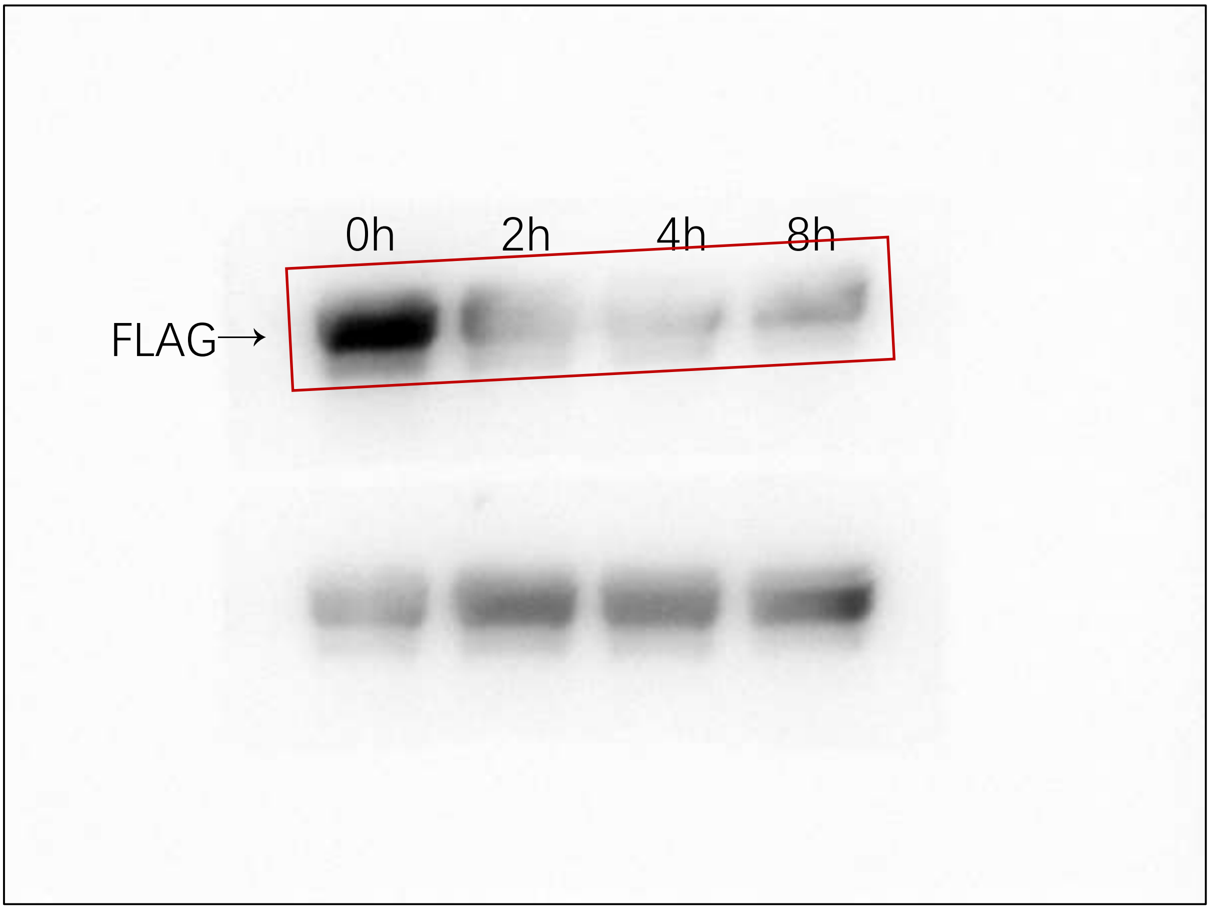

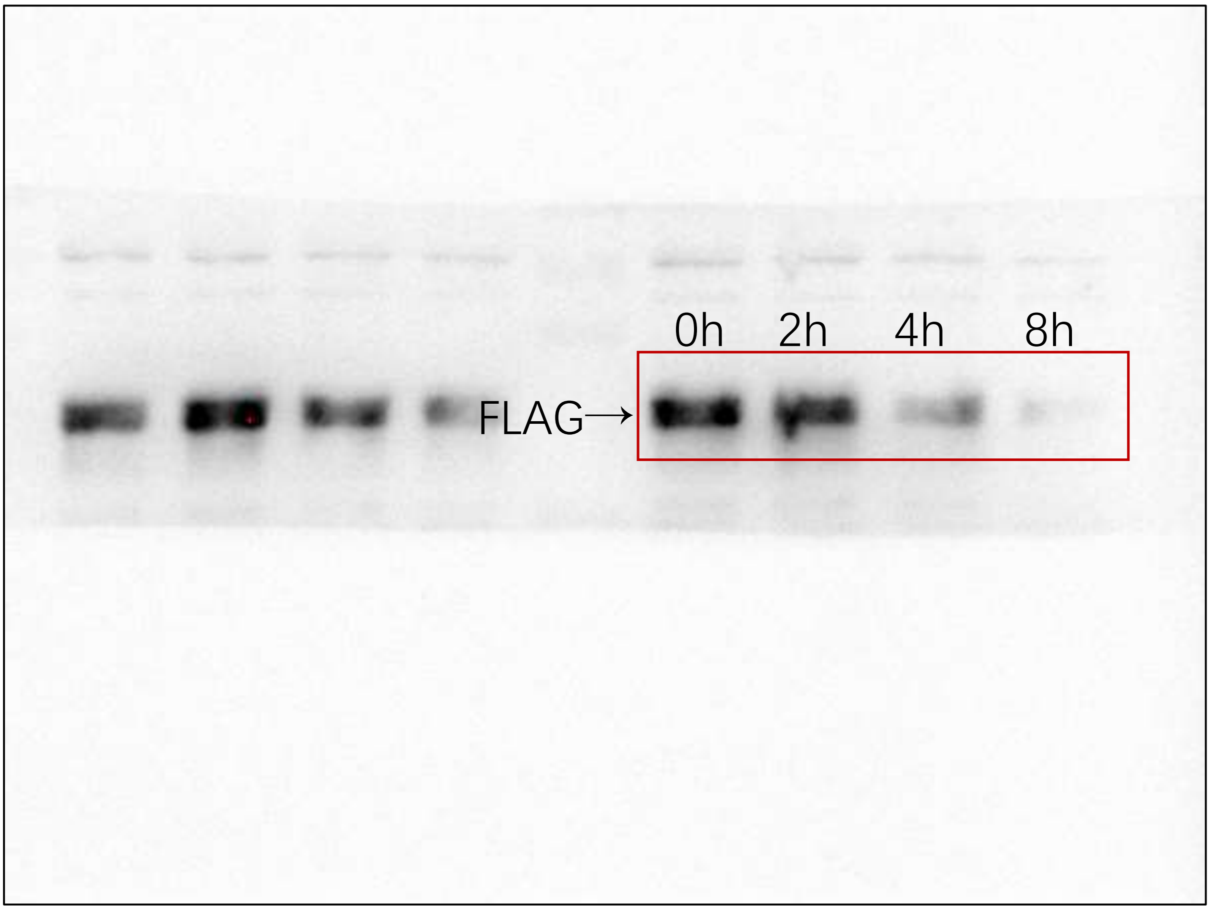

Original images of western blots of WT actin in figure 4e CDC20 & CDH1

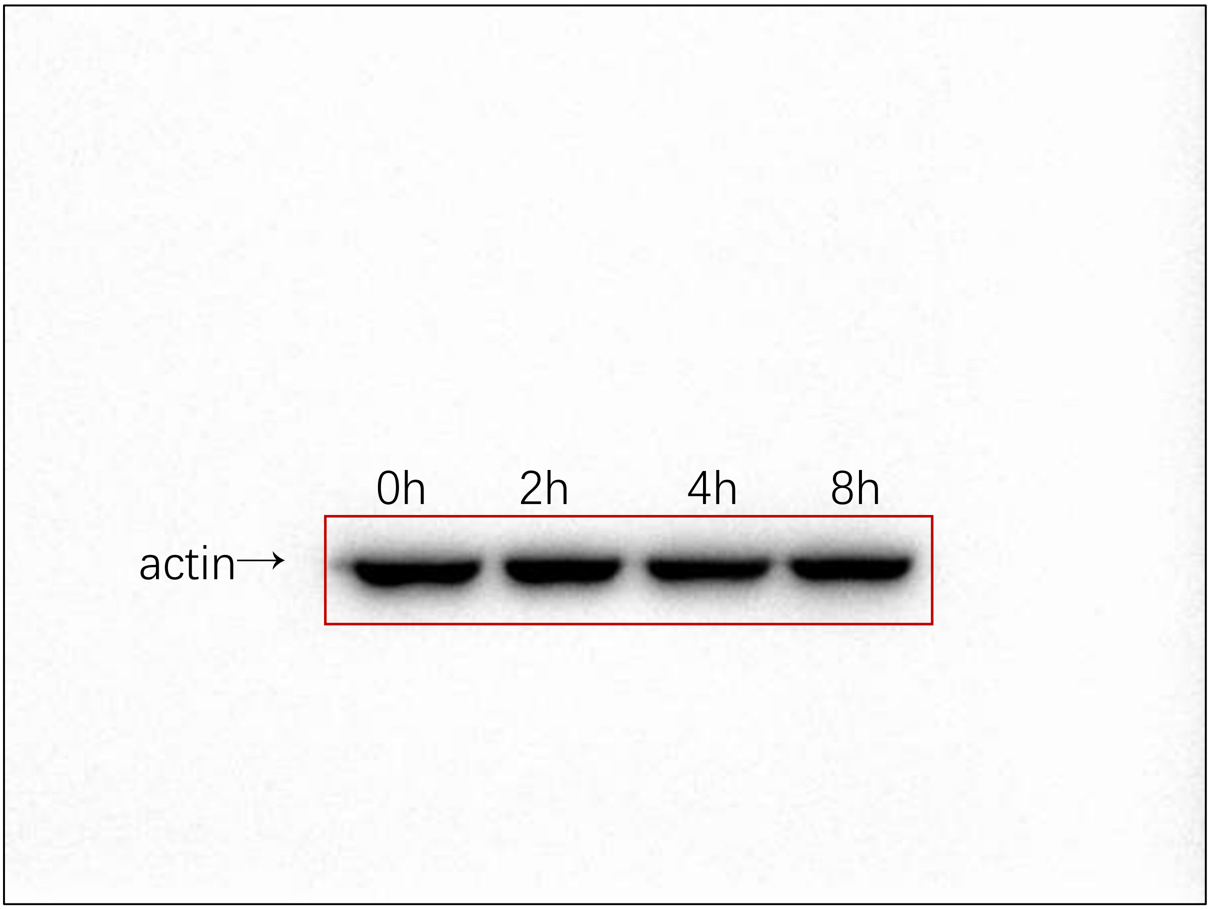

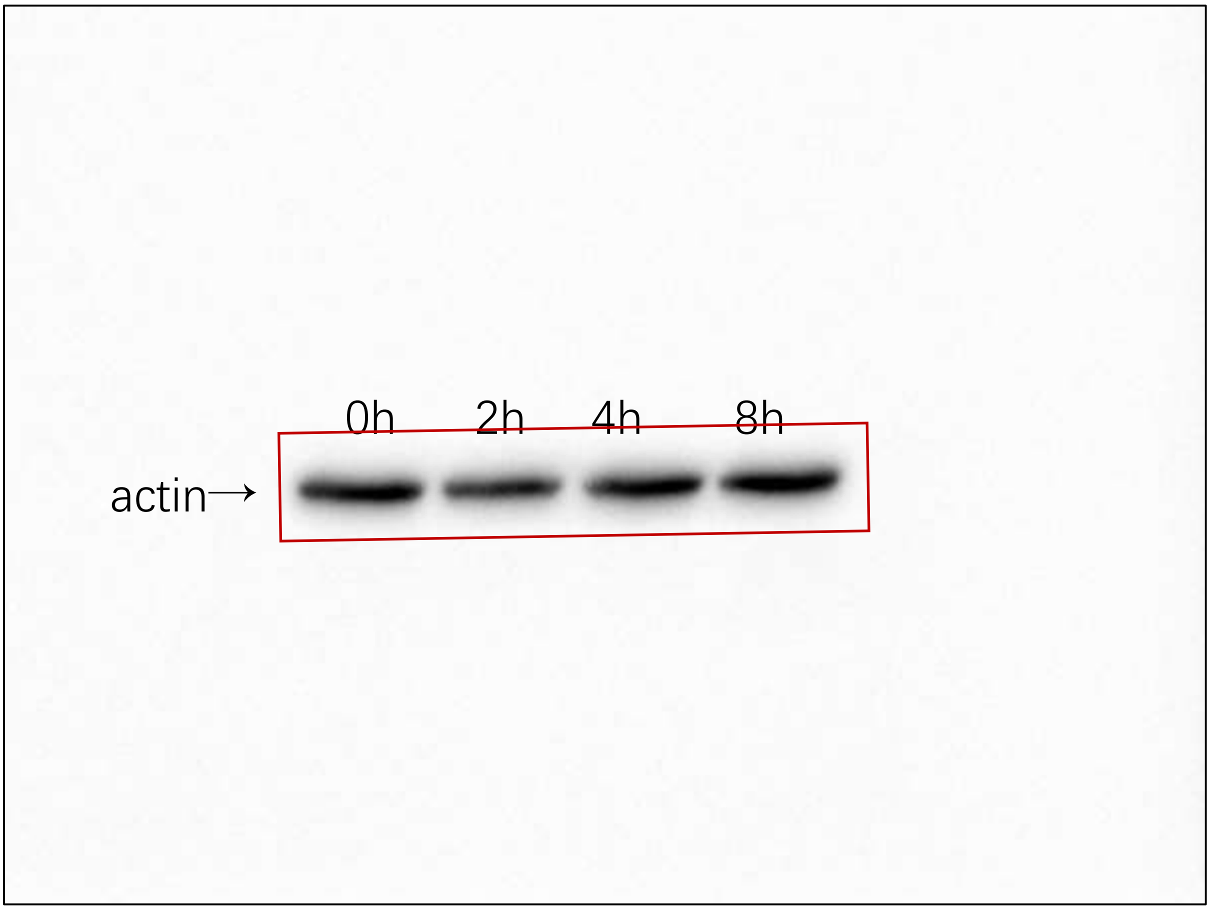

Original images of western blots of D1A FLAG in figure 4e CDC20 & CDH1

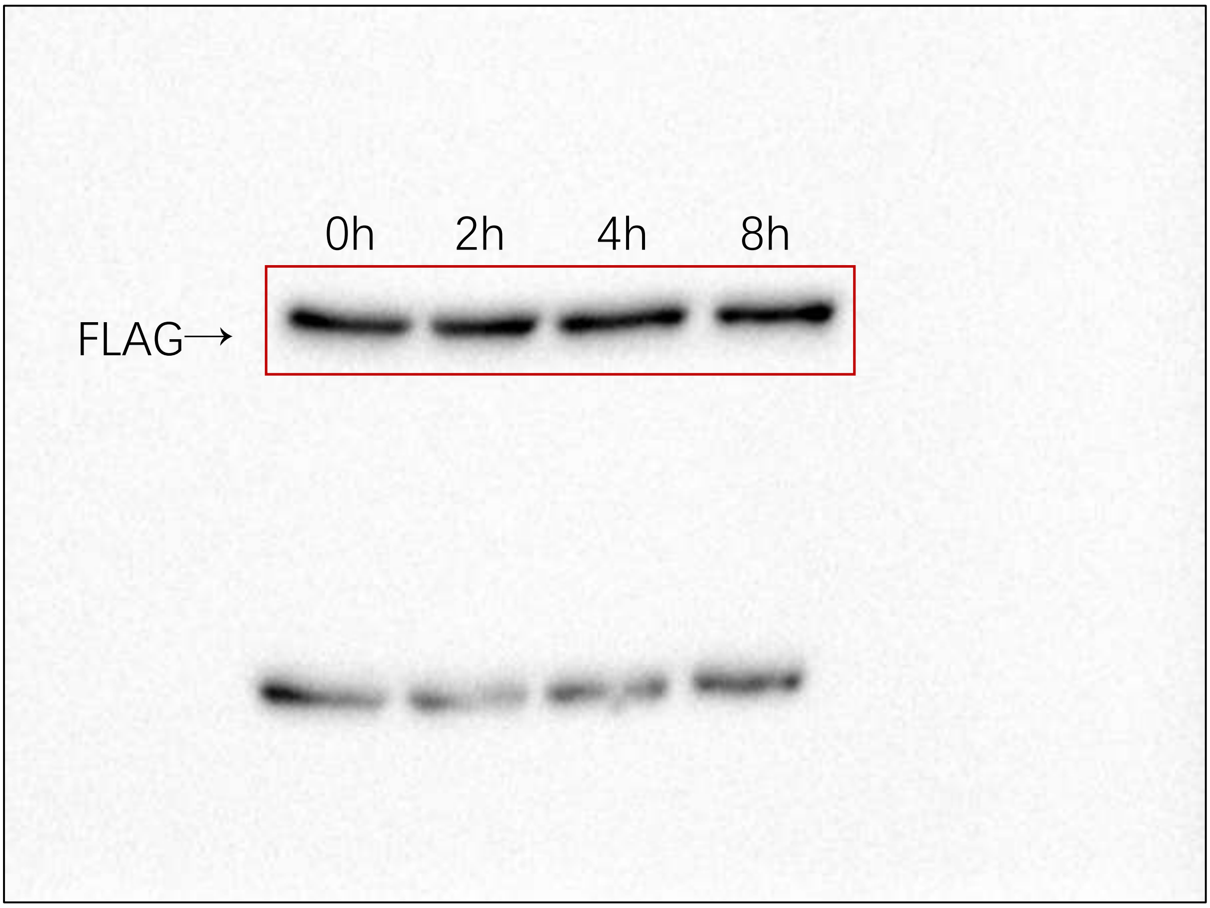

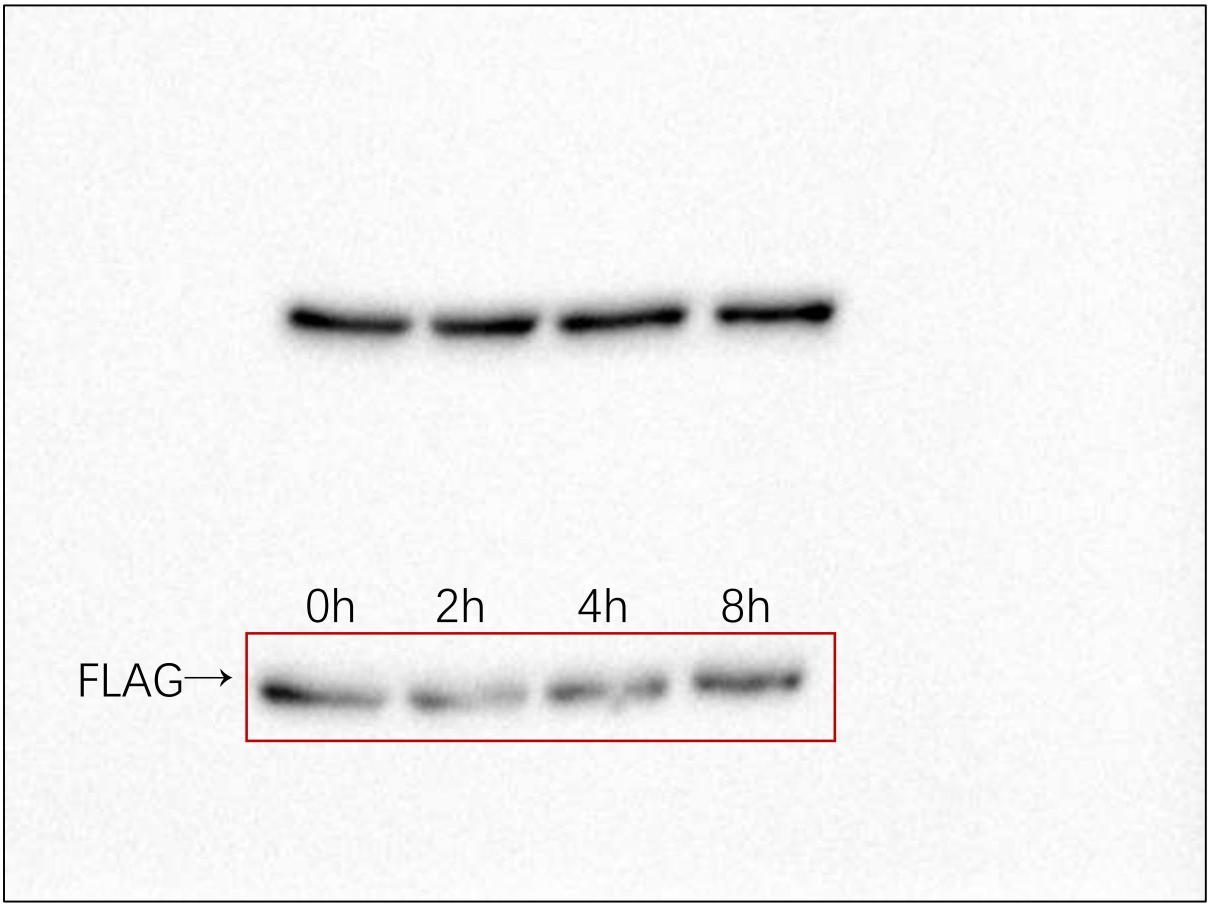

Original images of western blots of D1A actin in figure 4e CDC20 & CDH1

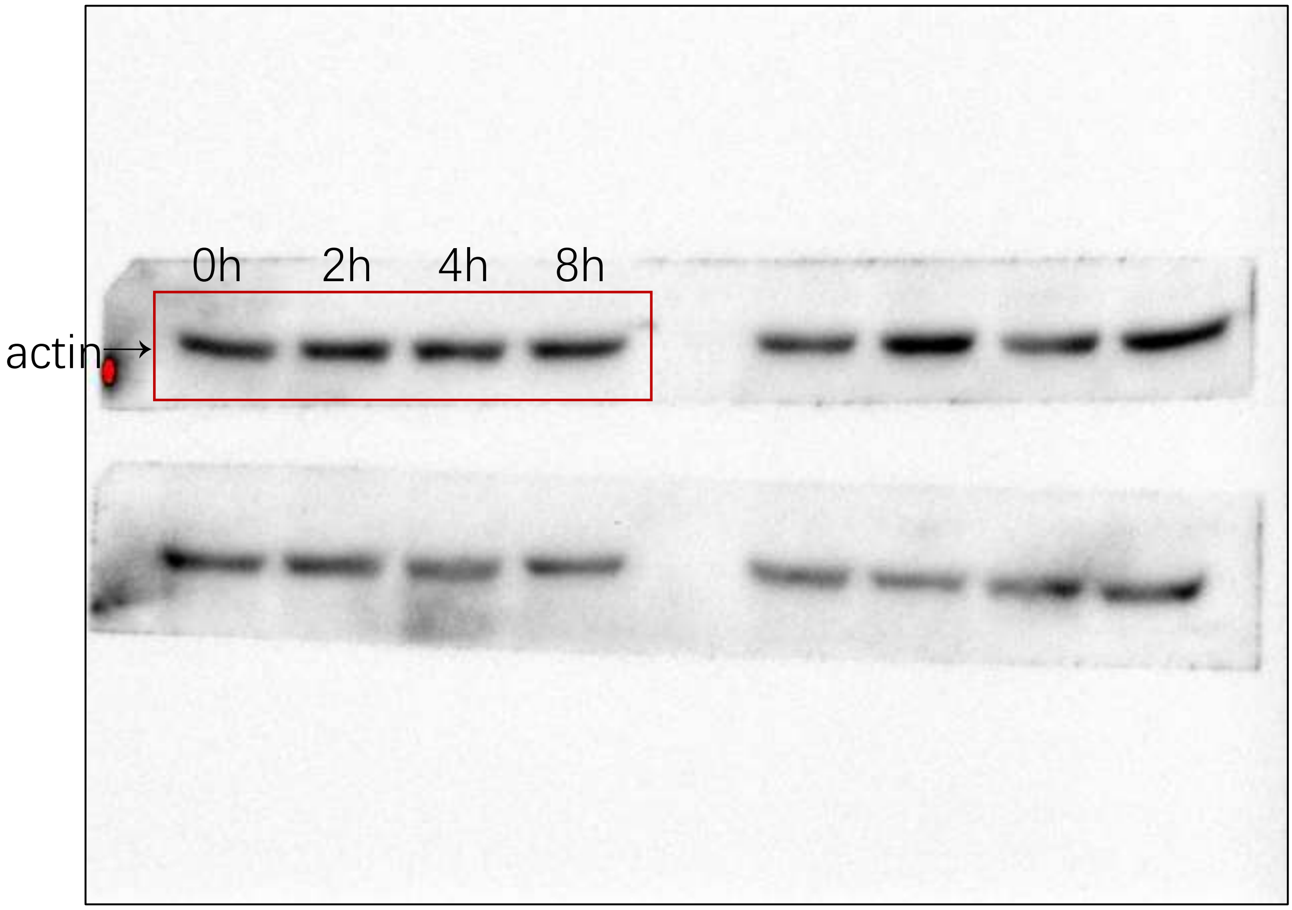

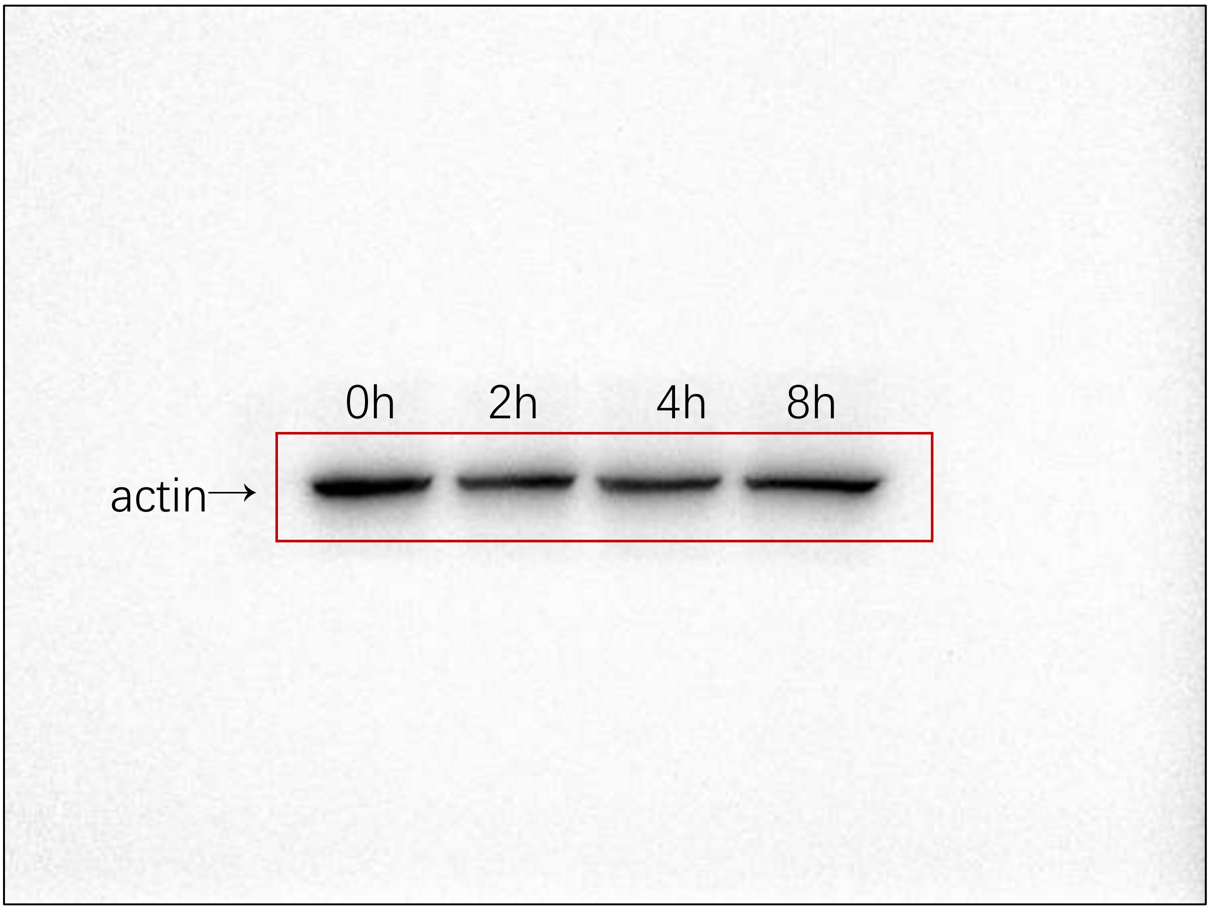

Original images of western blots of D2A FLAG in figure 4e CDC20 & CDH1

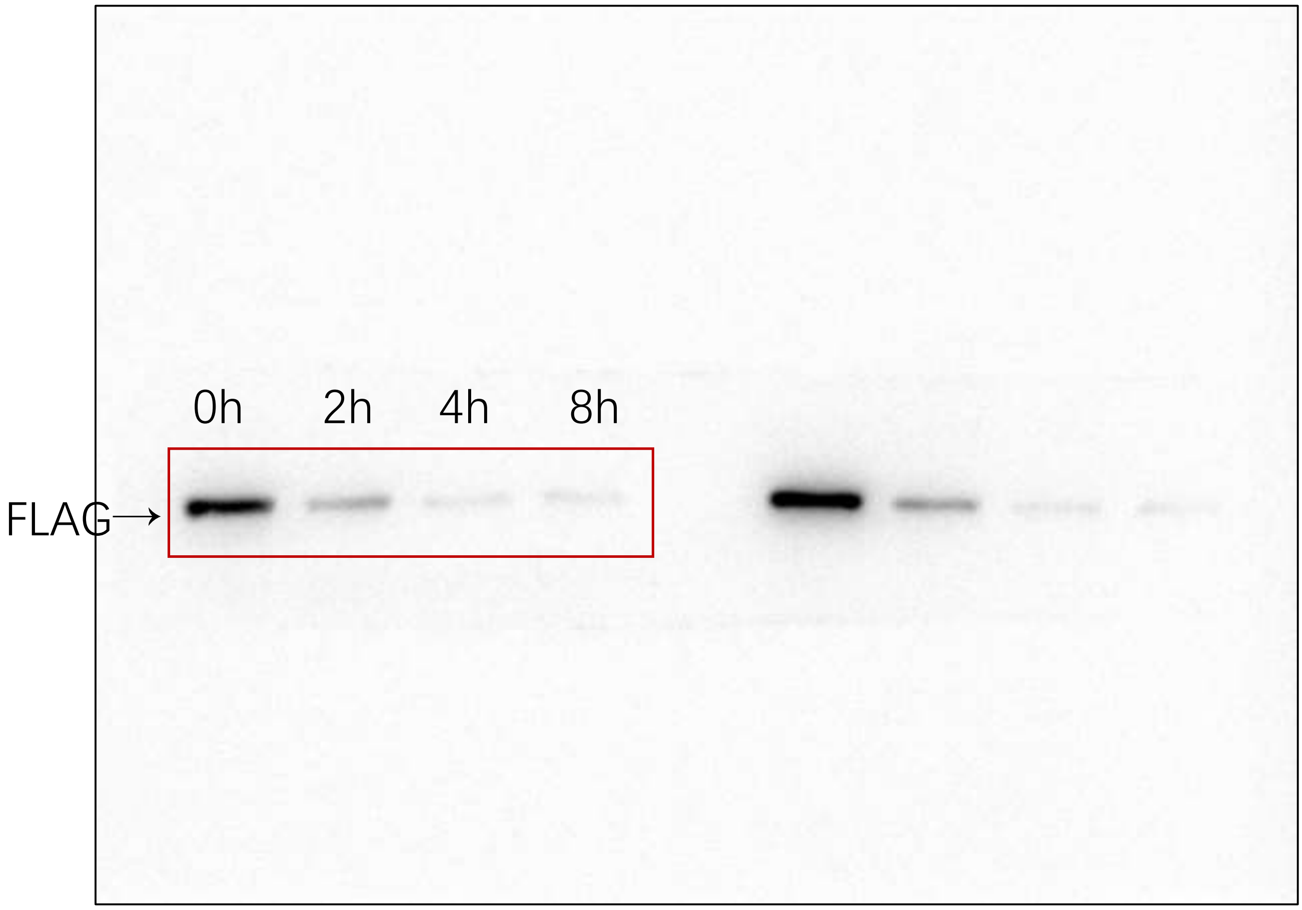

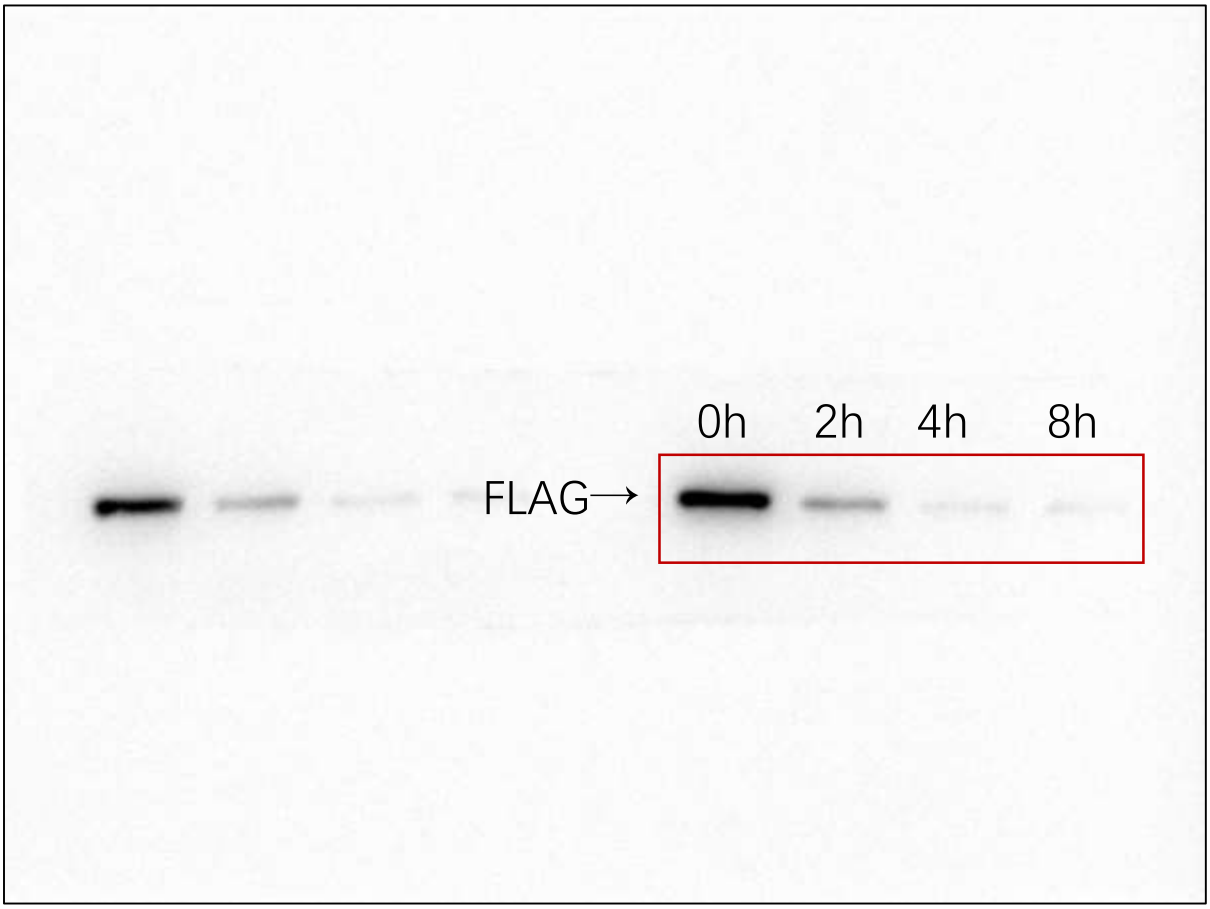

Original images of western blots of D2A actin in figure 4e CDC20 & CDH1

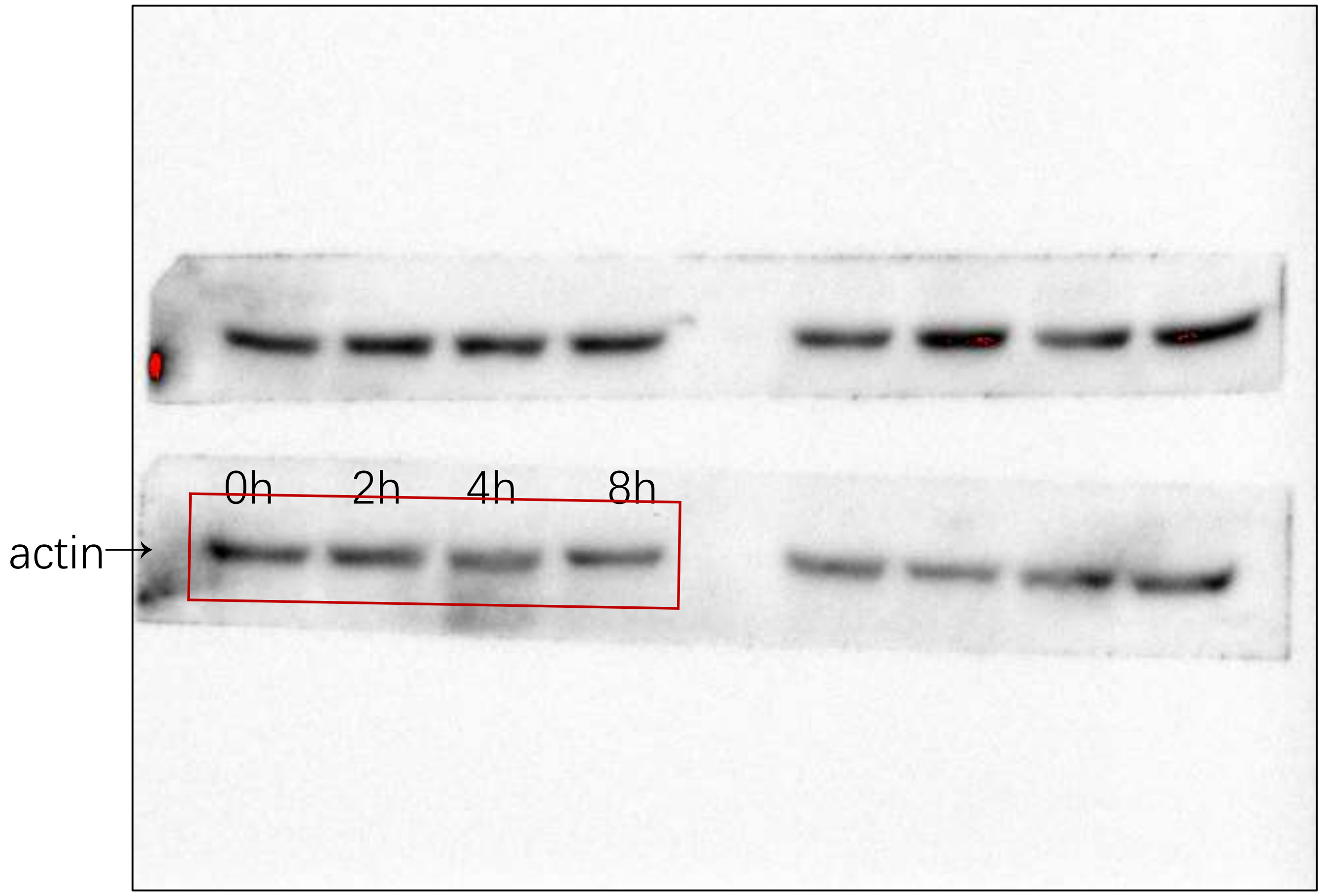

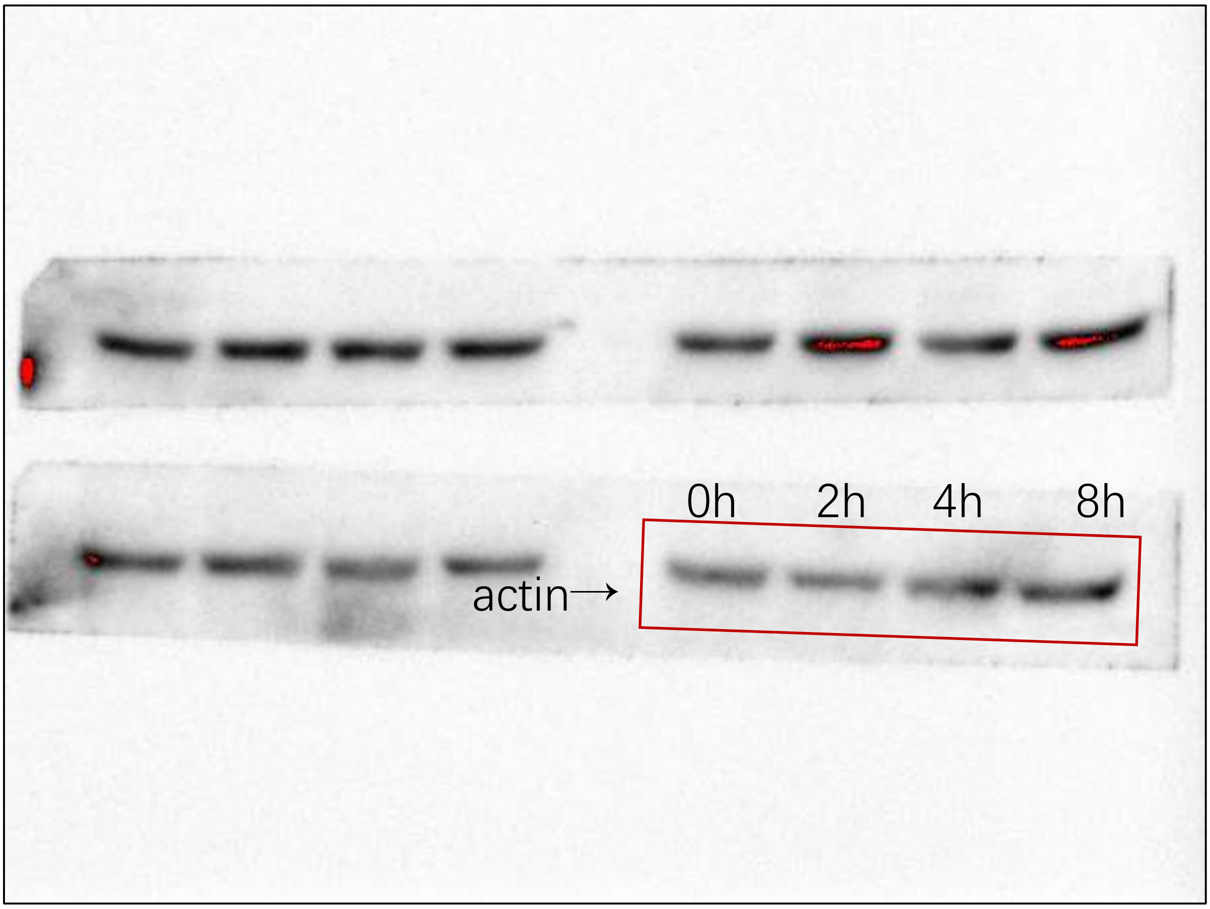

Original images of western blots of D3A FLAG in figure 4e CDC20 & CDH1

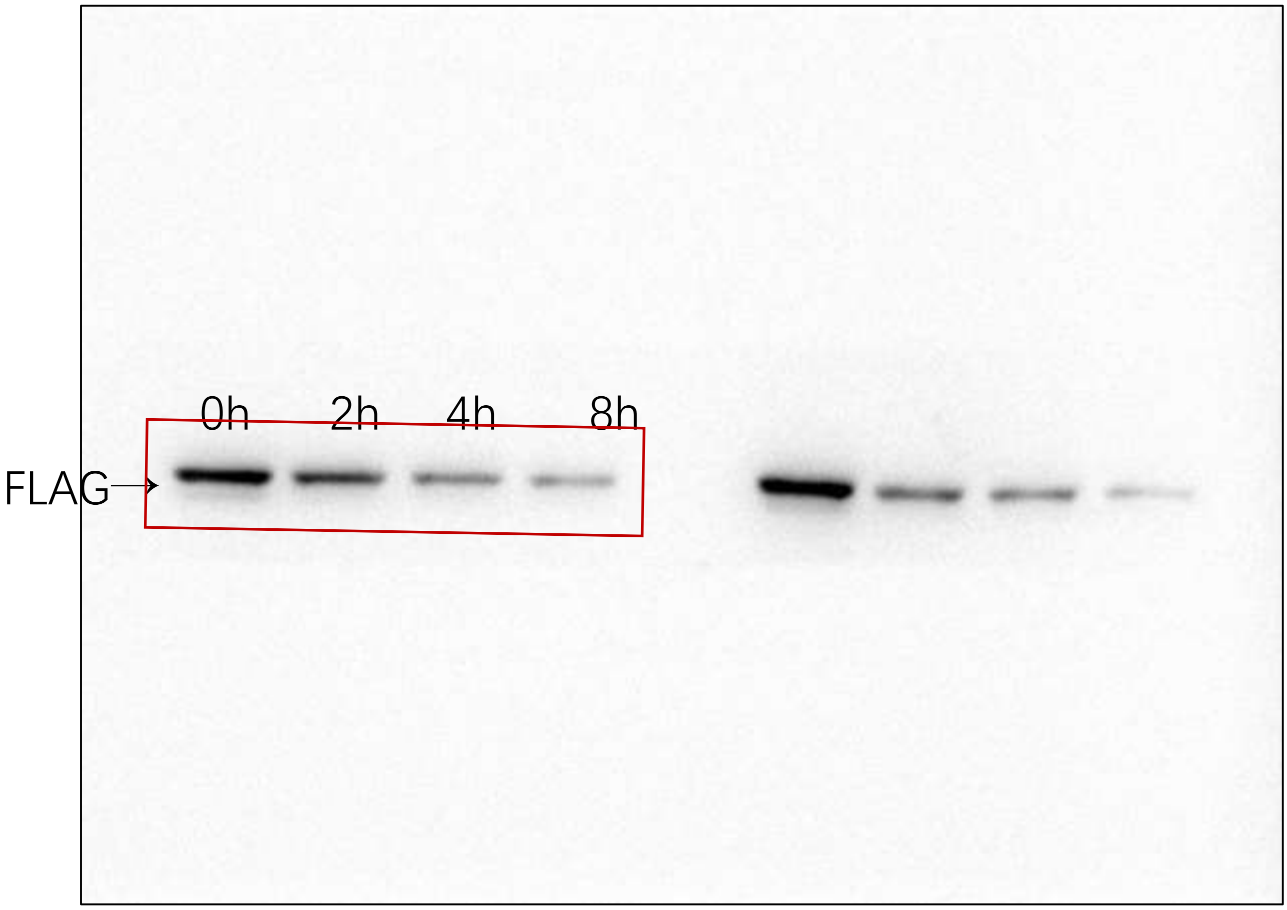

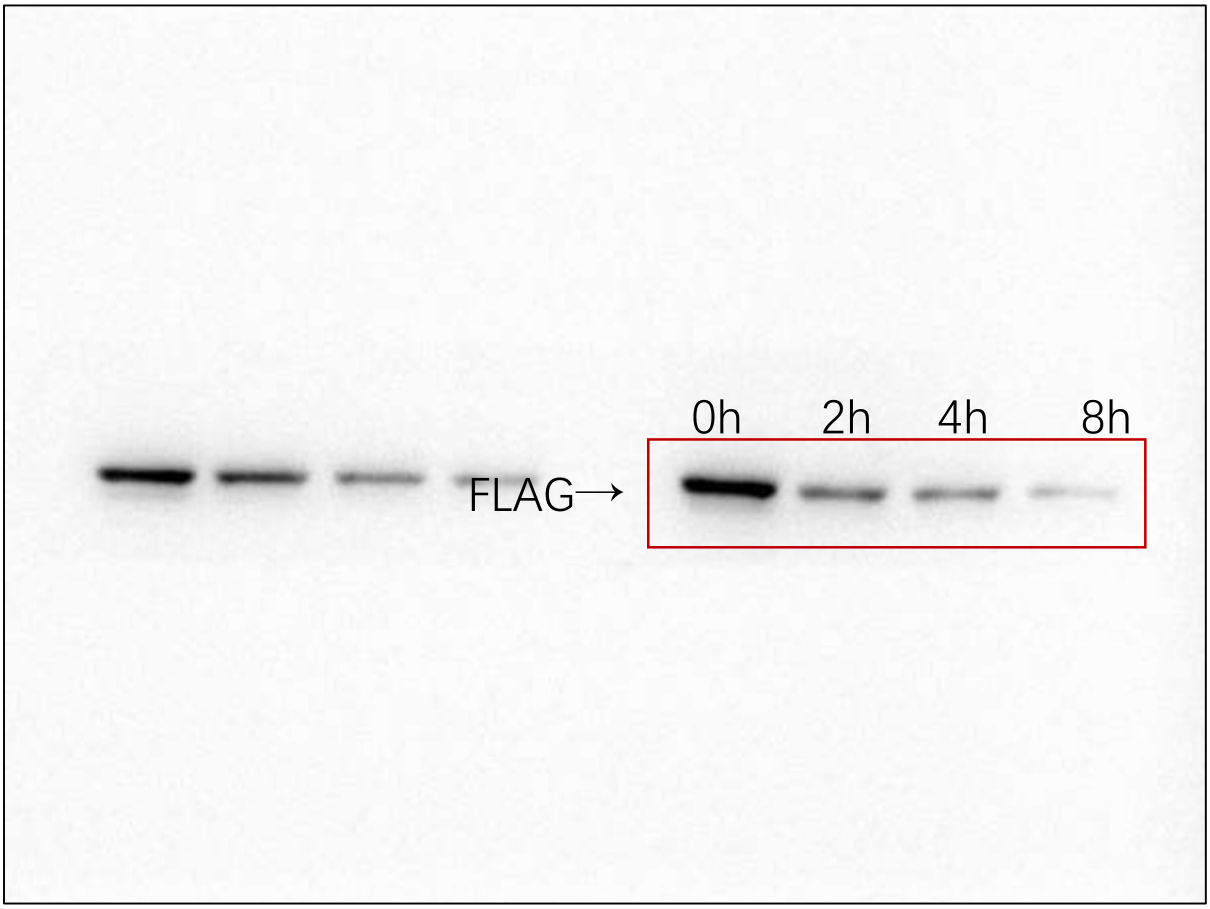

Original images of western blots of D3A actin in figure 4e CDC20 & CDH1

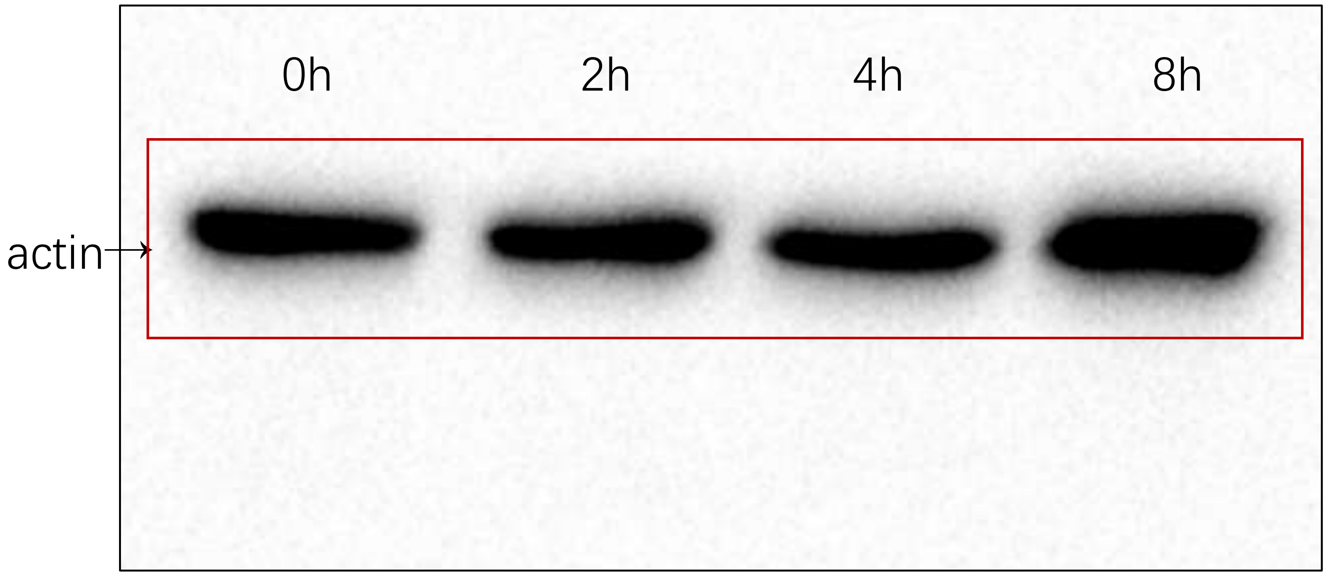

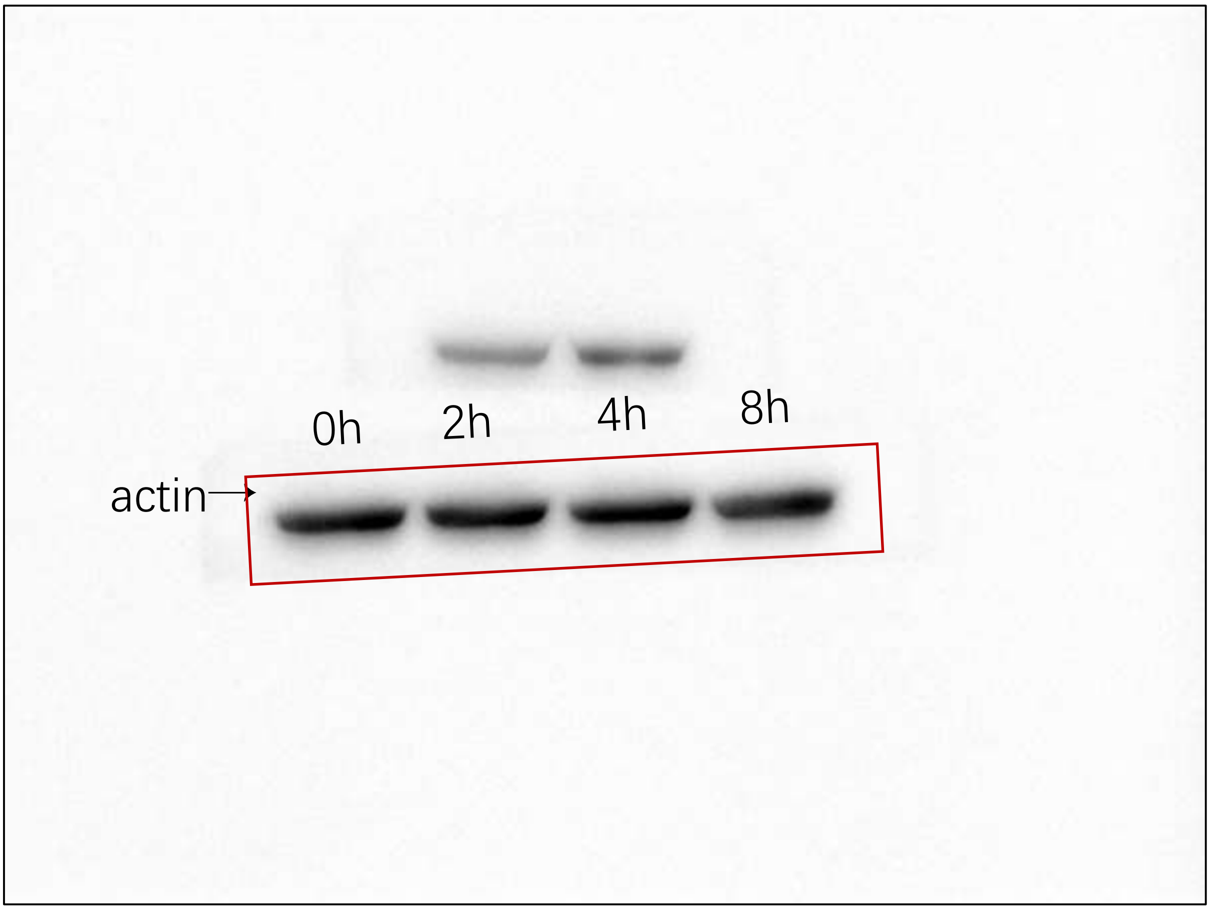

Original images of western blots of SIRT6-FLAG and CDH1 in figure 5a 293T

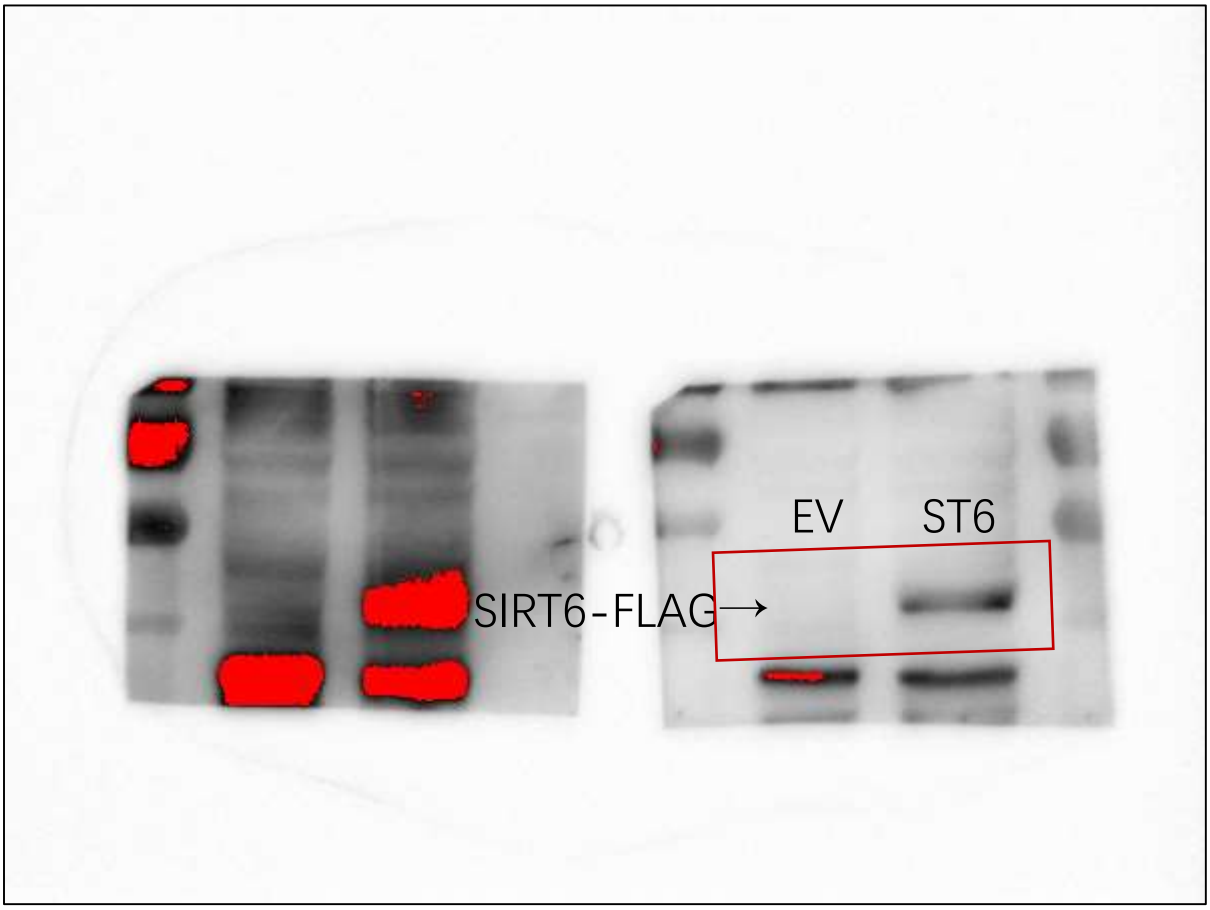

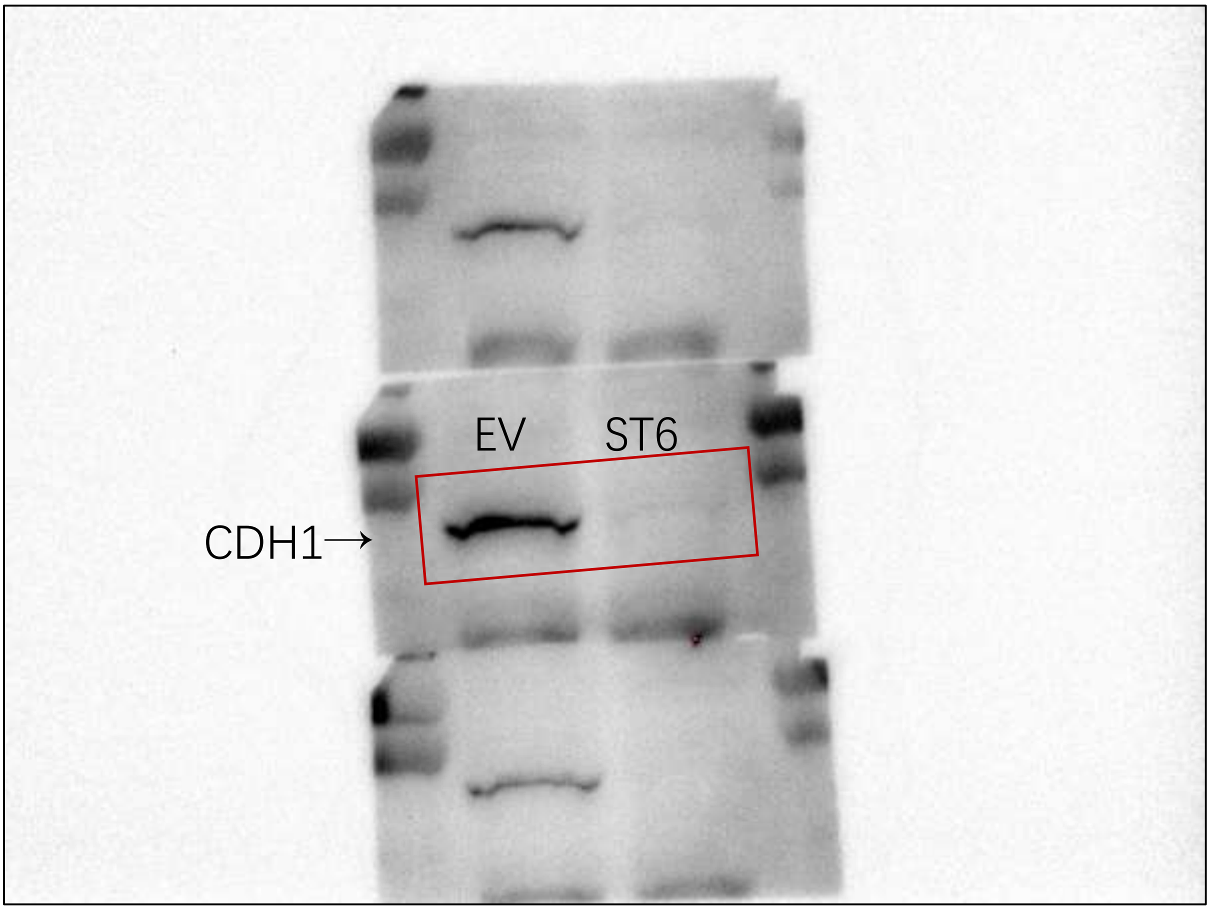

Original images of western blots of HSP70 in figure 5a 293T

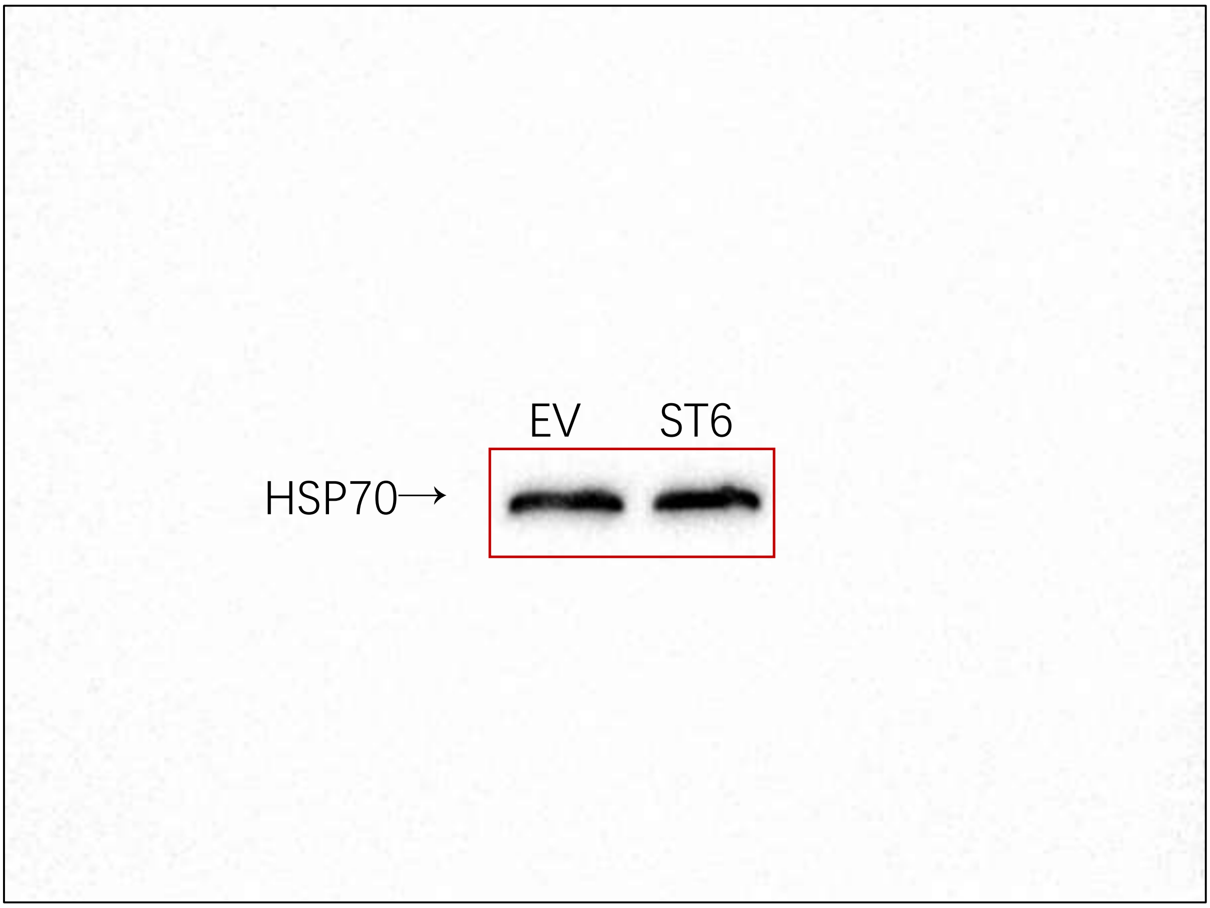

Original images of western blots of SIRT6-FLAG and CDH1 in figure 5a HeLa

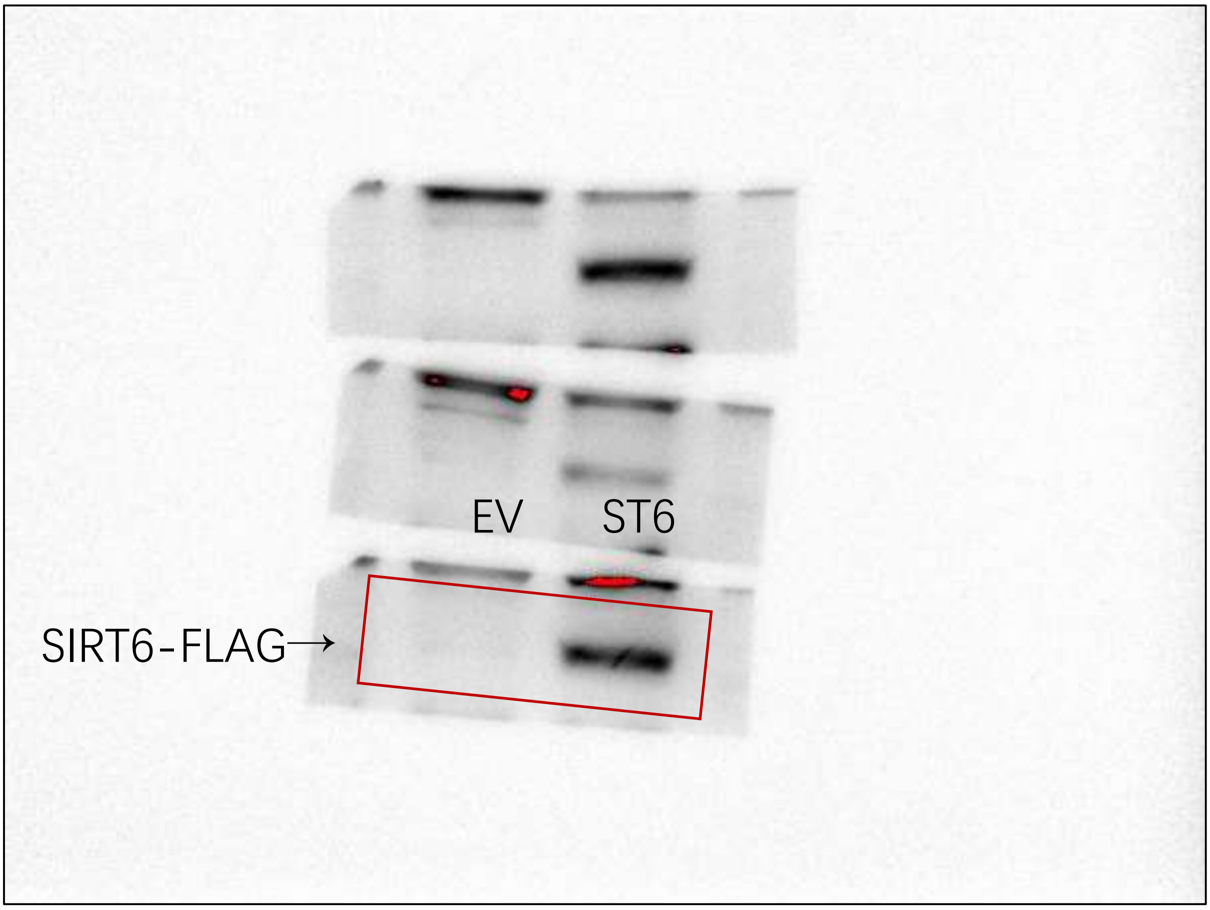

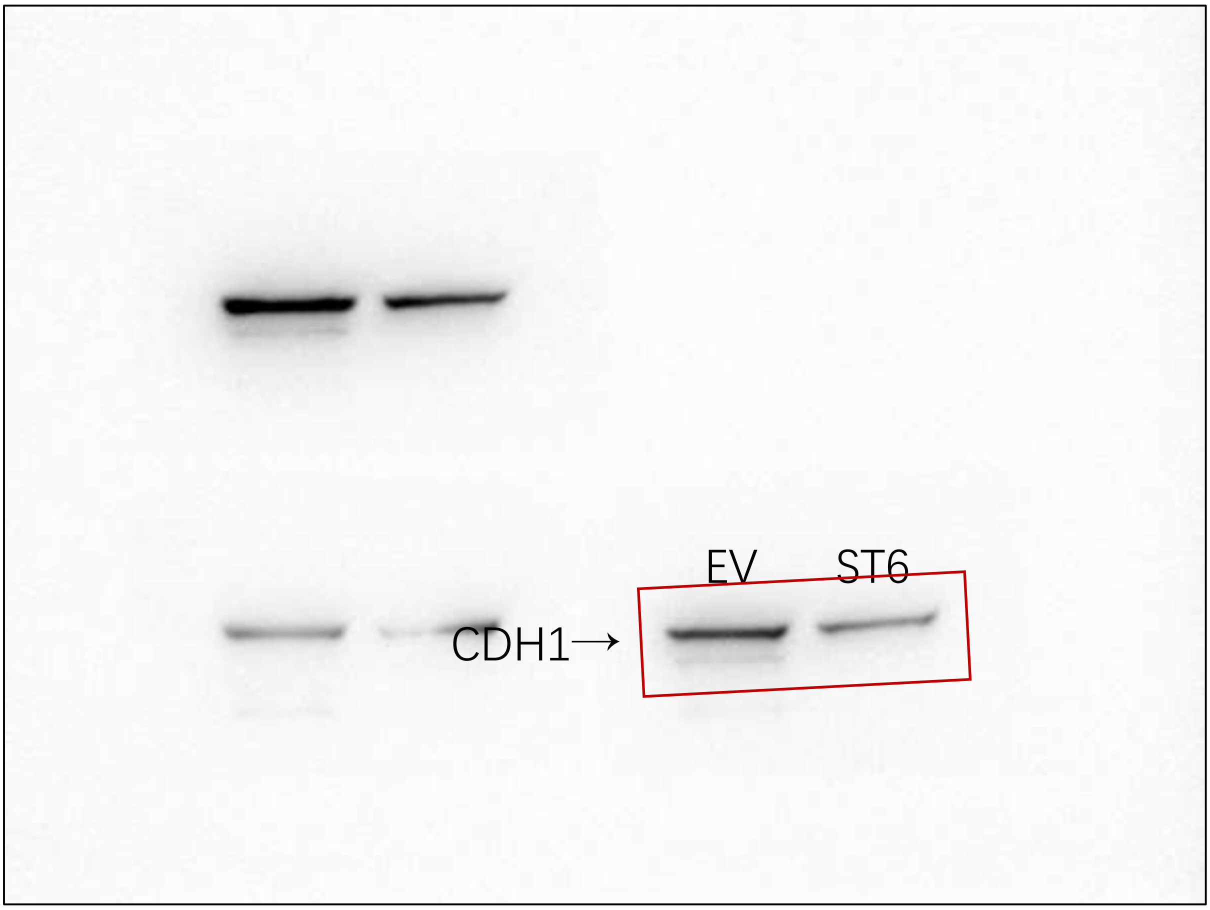

Original images of western blots of HSP70 in figure 5a HeLa

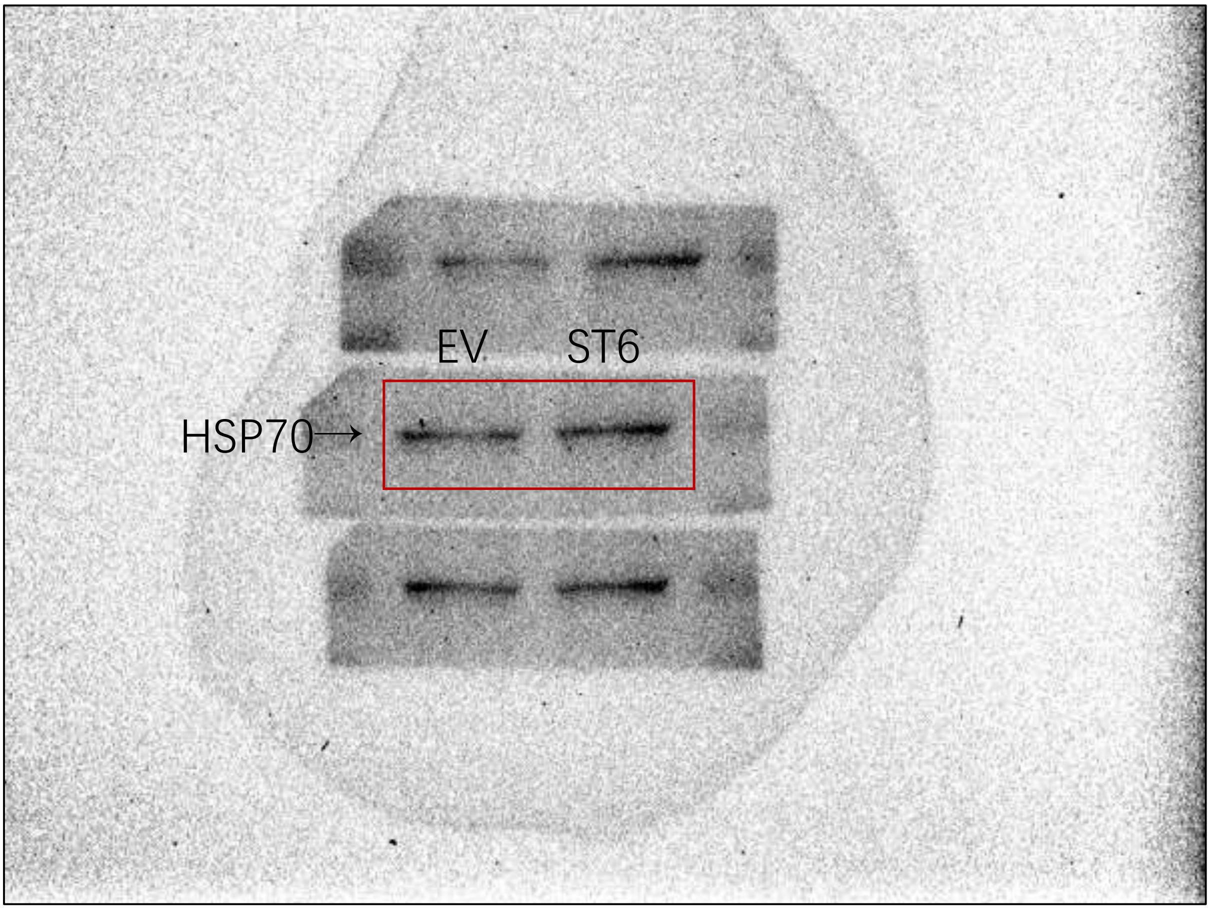

Original images of western blots of SIRT6 and CDH1 in figure 5b 293T

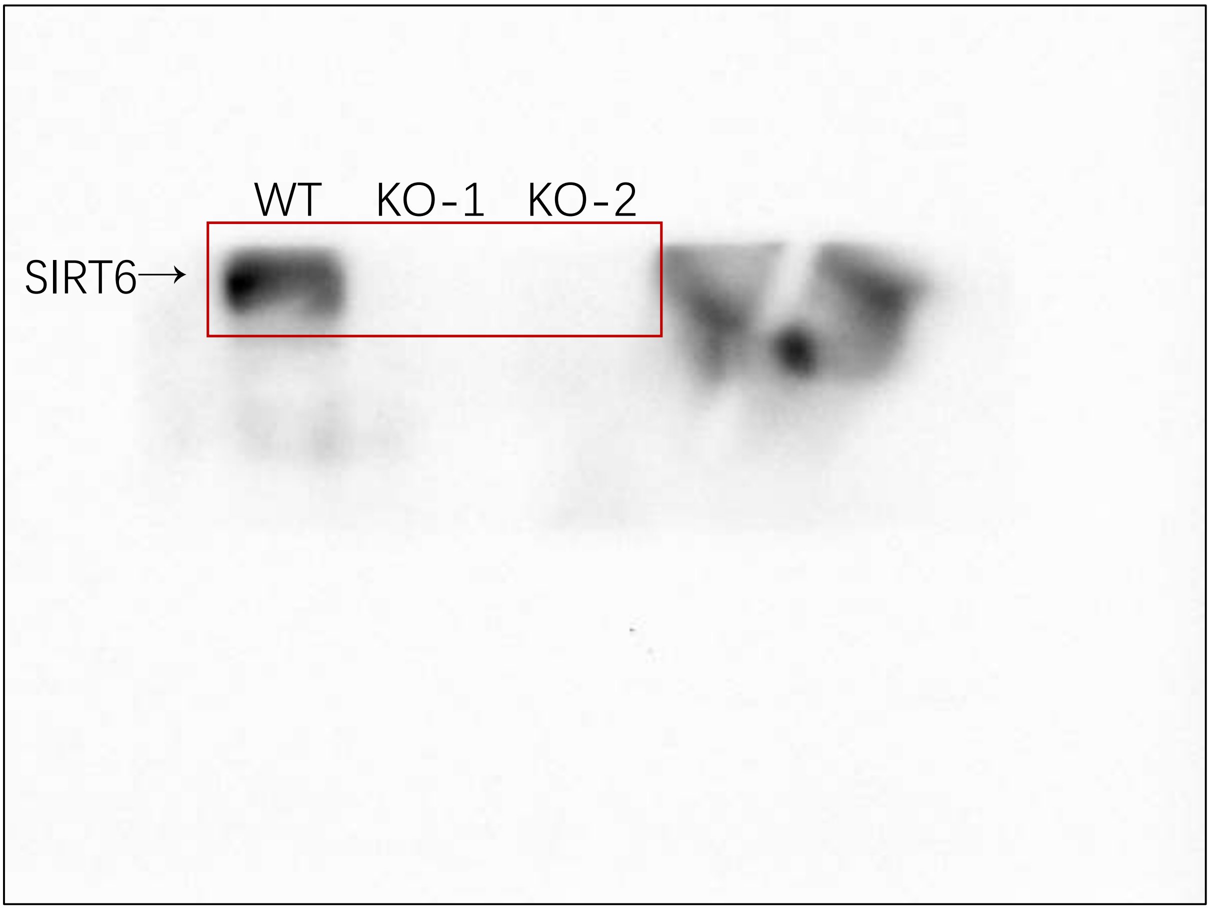

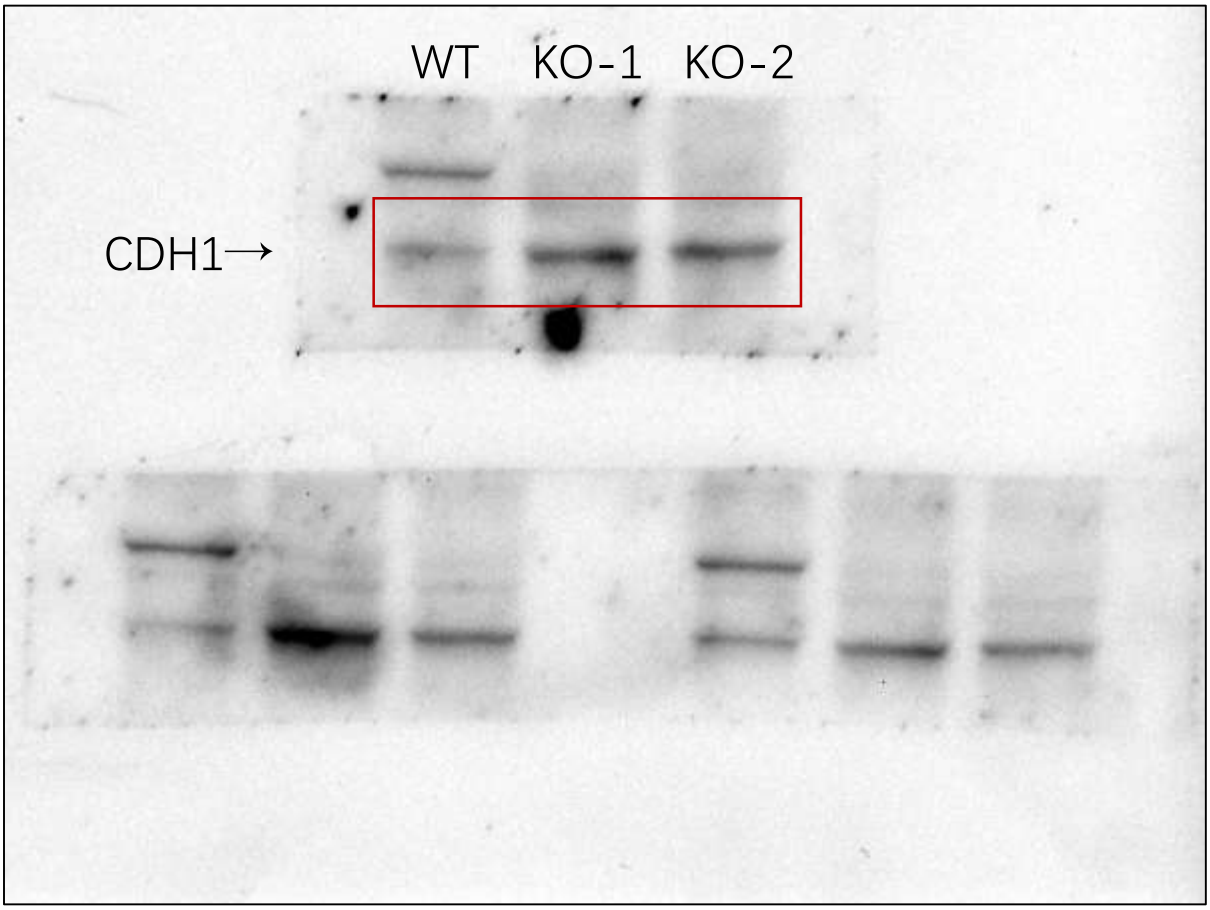

Original images of western blots of GAPDH in figure 5b 293T

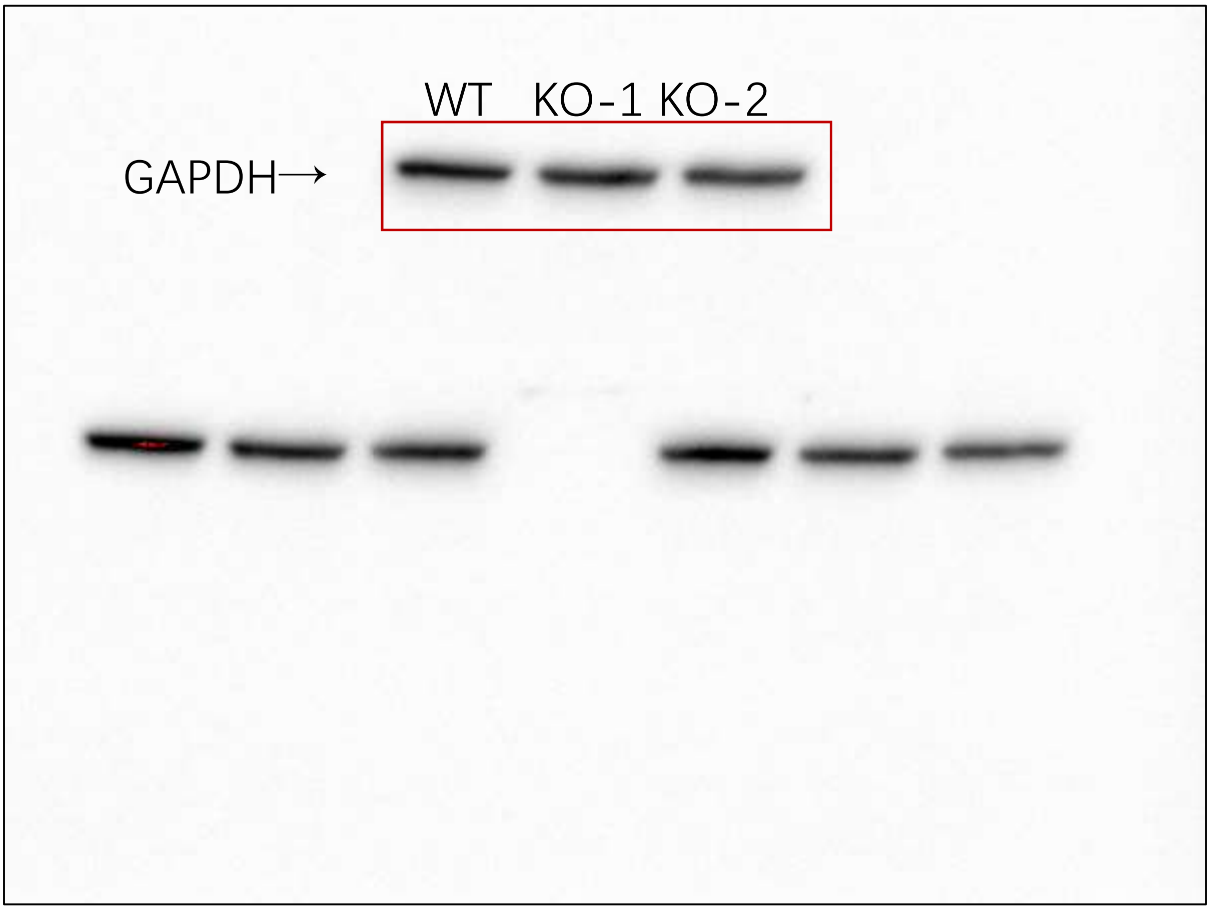

Original images of western blots of SIRT6 and CDH1 in figure 5b HeLa

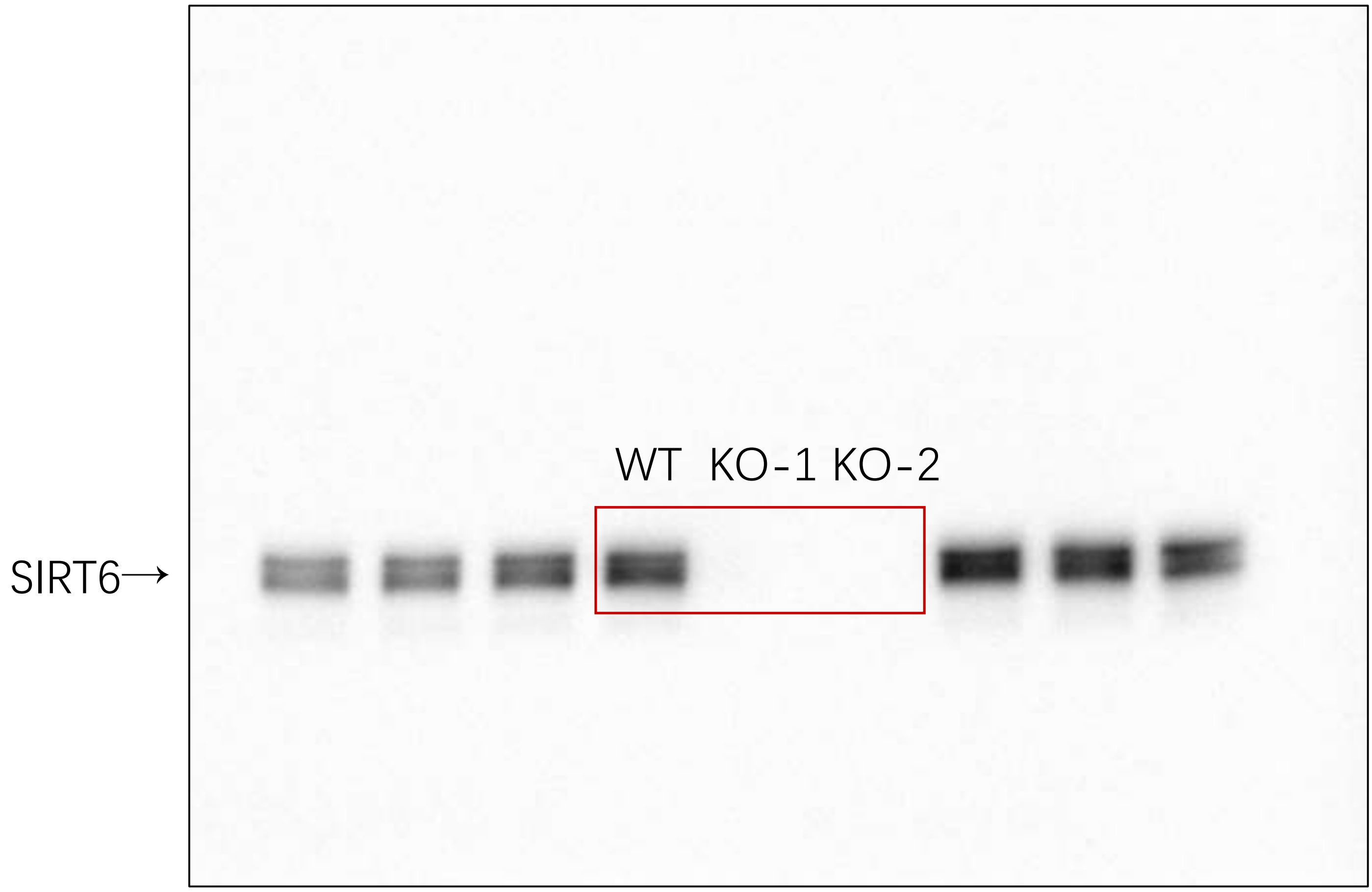

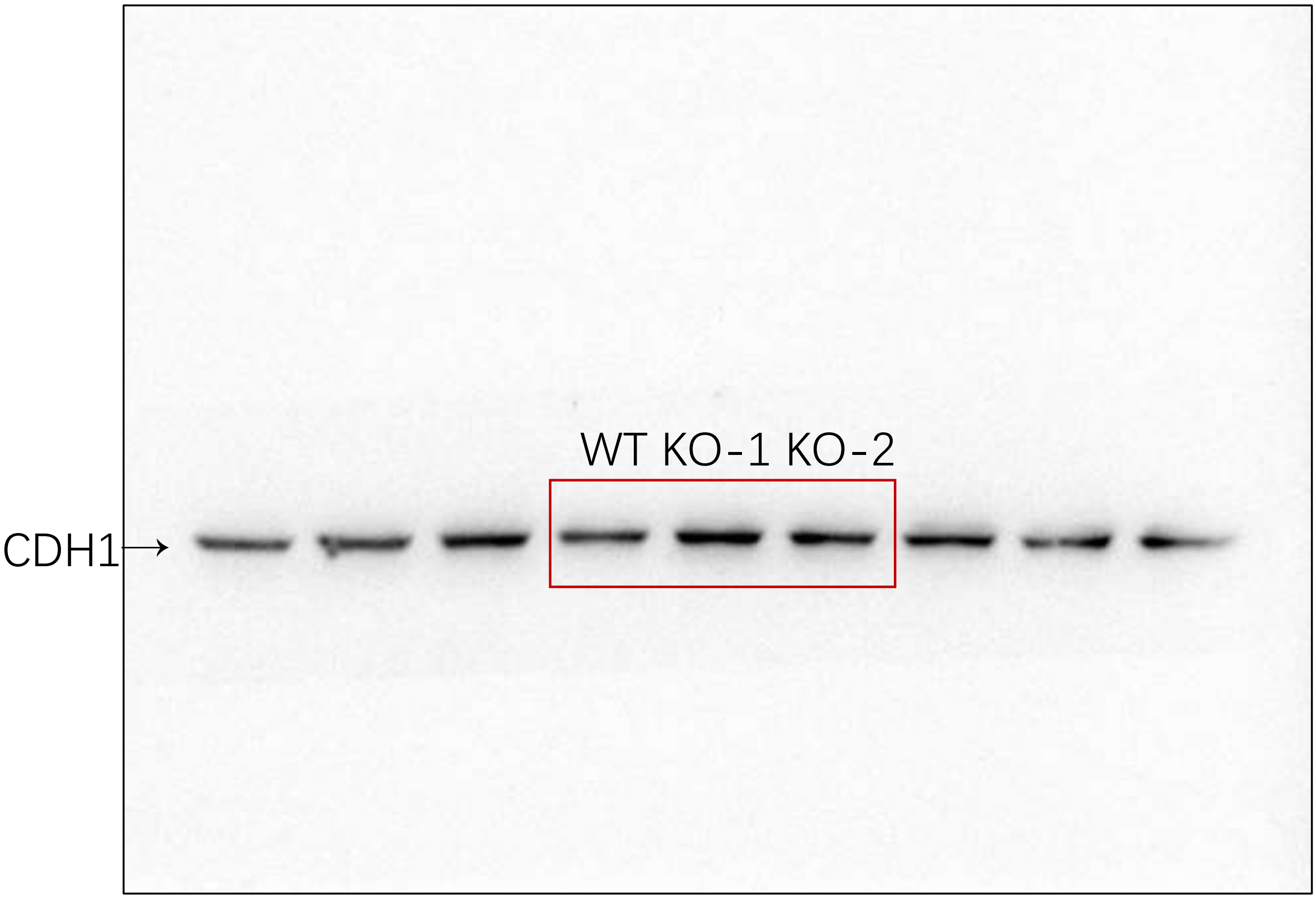

Original images of western blots of GAPDH in figure 5b HeLa

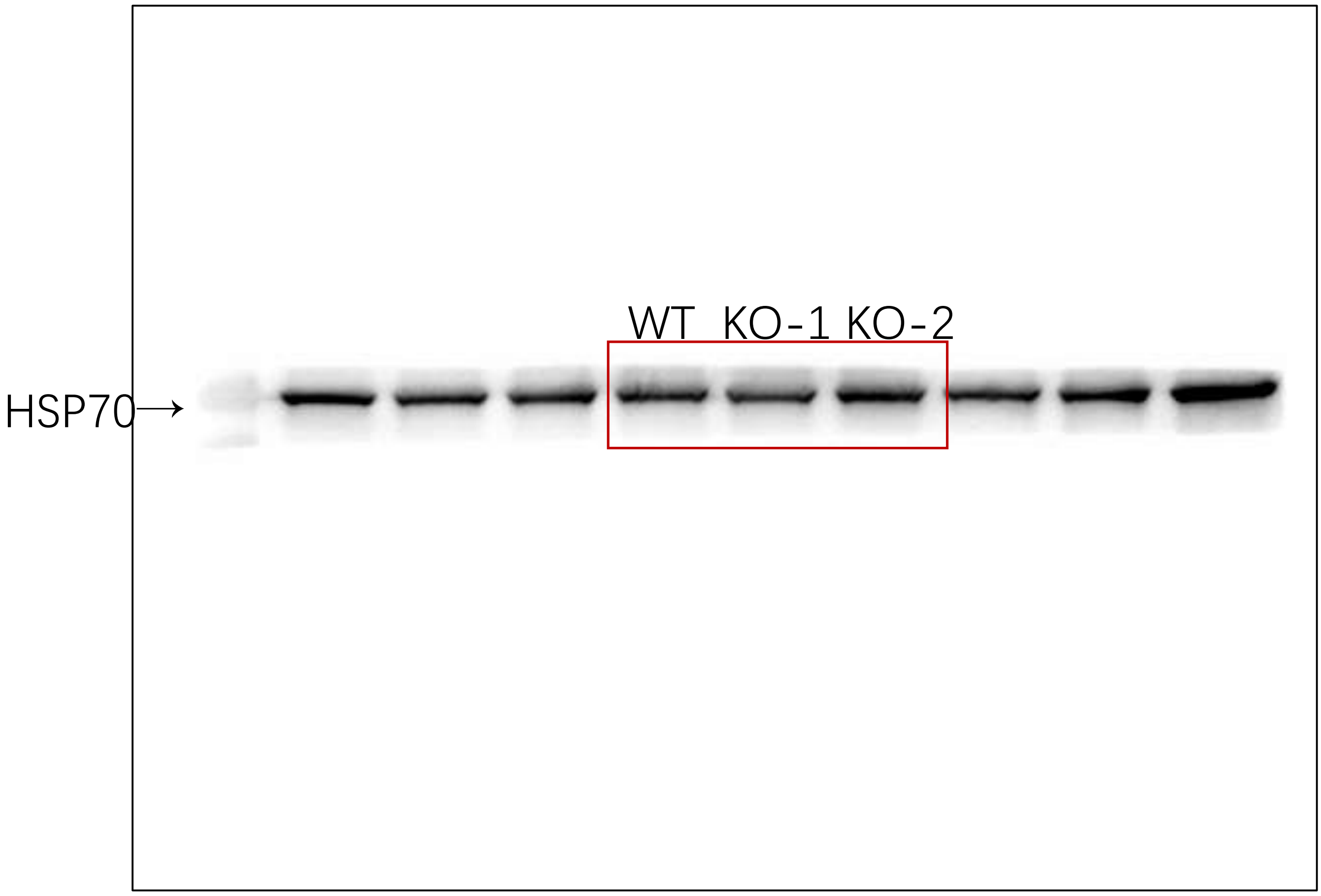

Original images of western blots of CDH1 and FLAG in figure 5c

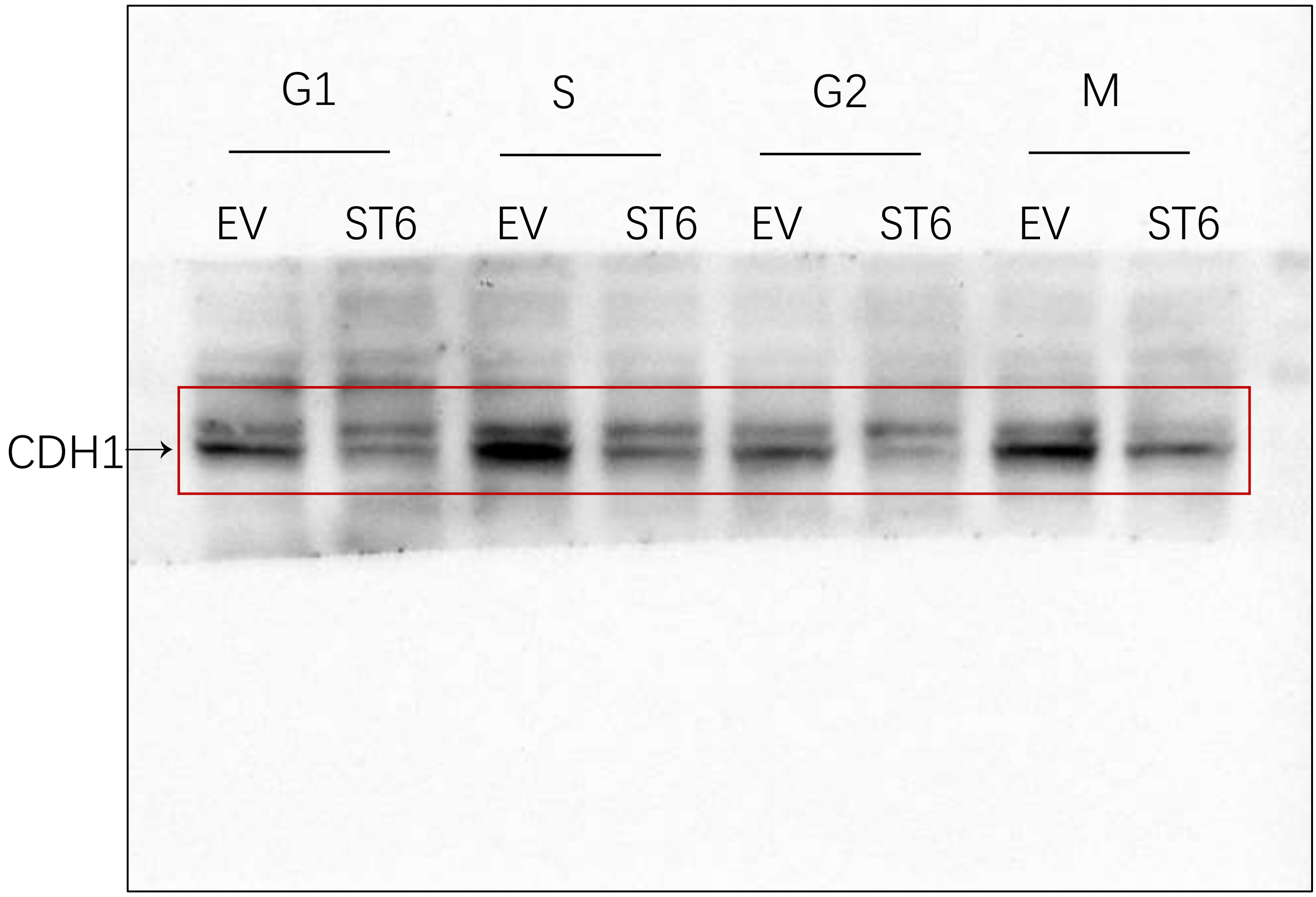

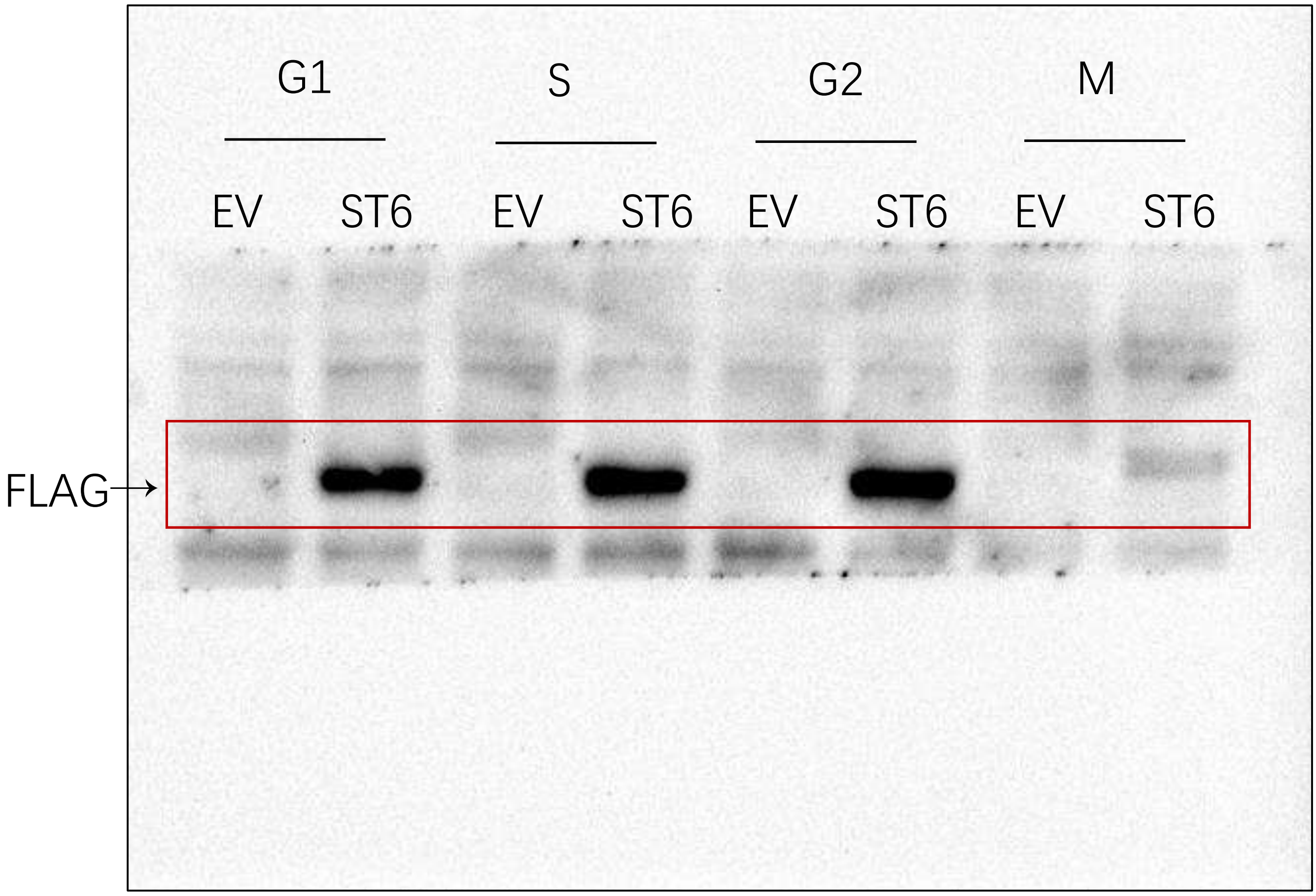

Original images of western blots of Cyc B1 and phoH3@Ser10 in figure 5c

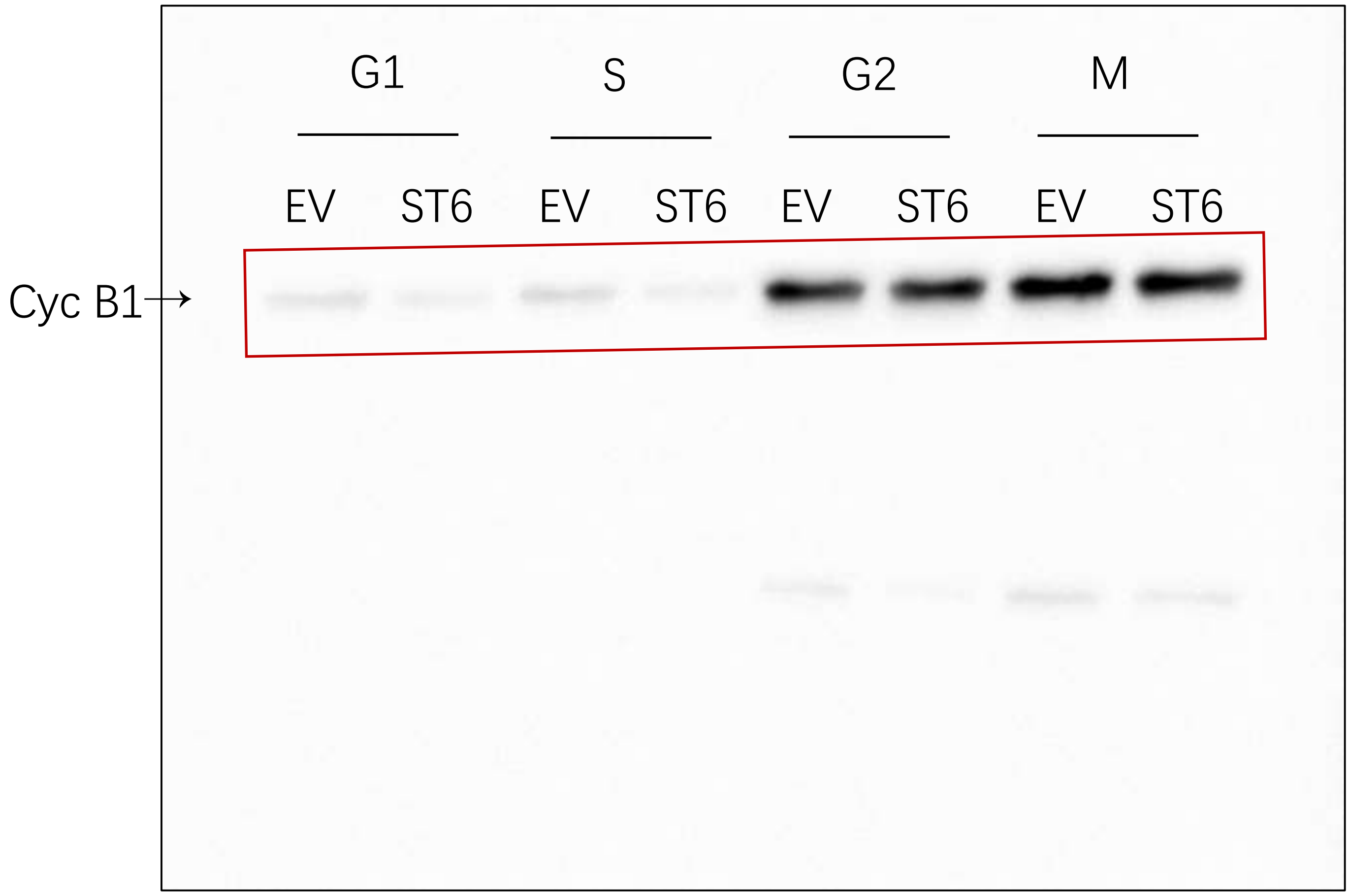

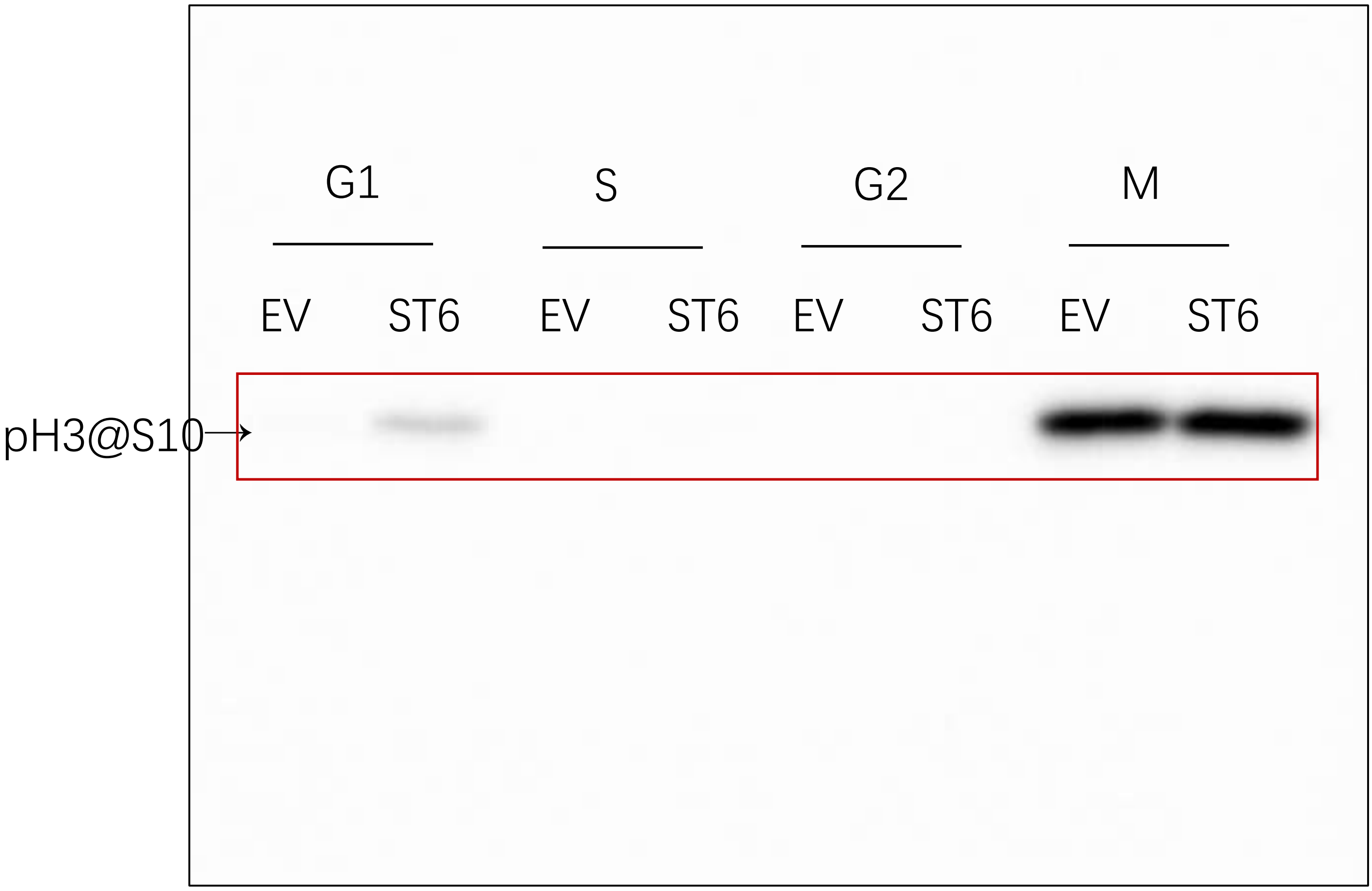

Original images of western blots of GAPDH in figure 5c

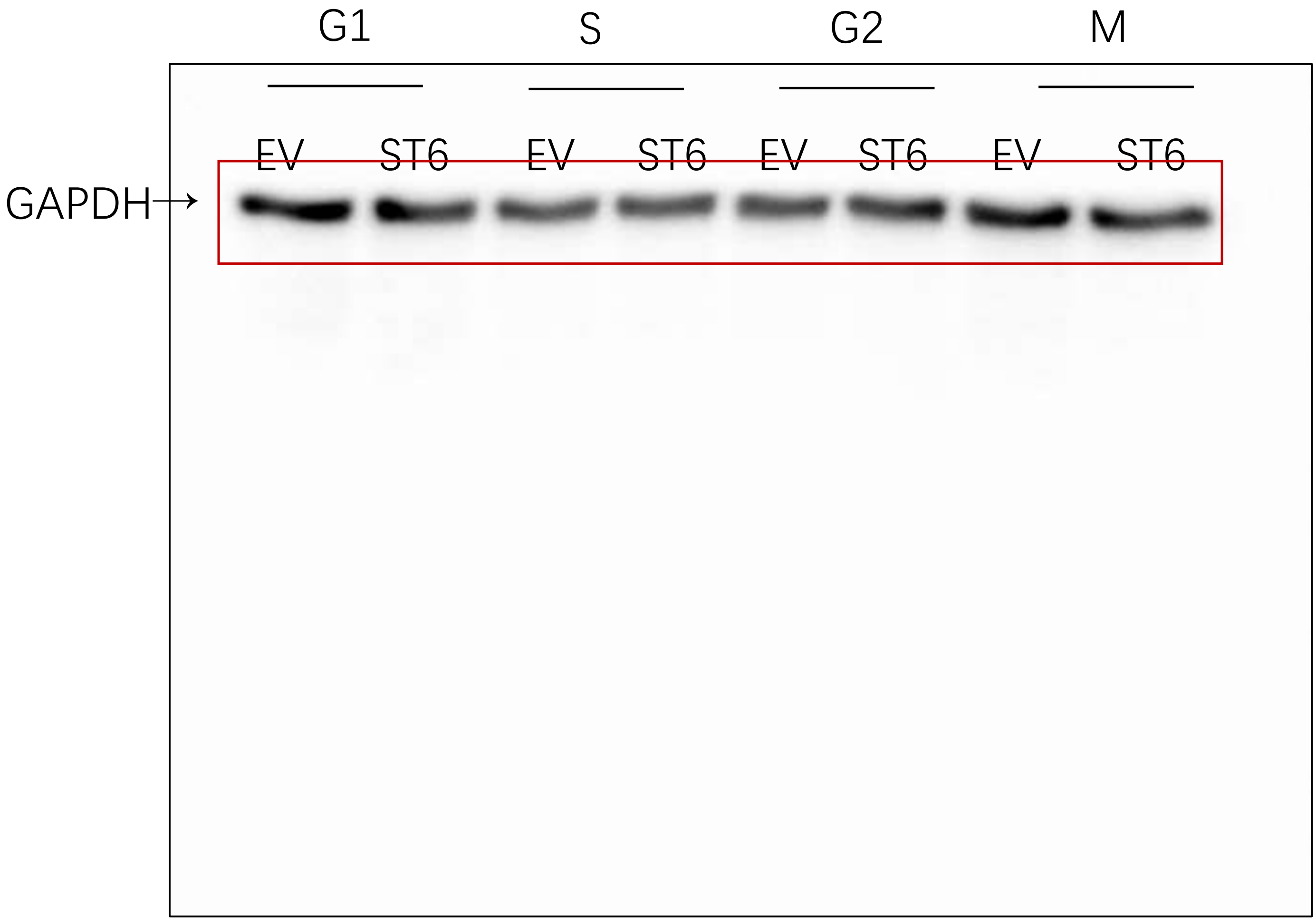

Original images of western blots of CDH1 & actin in figure 5d EV

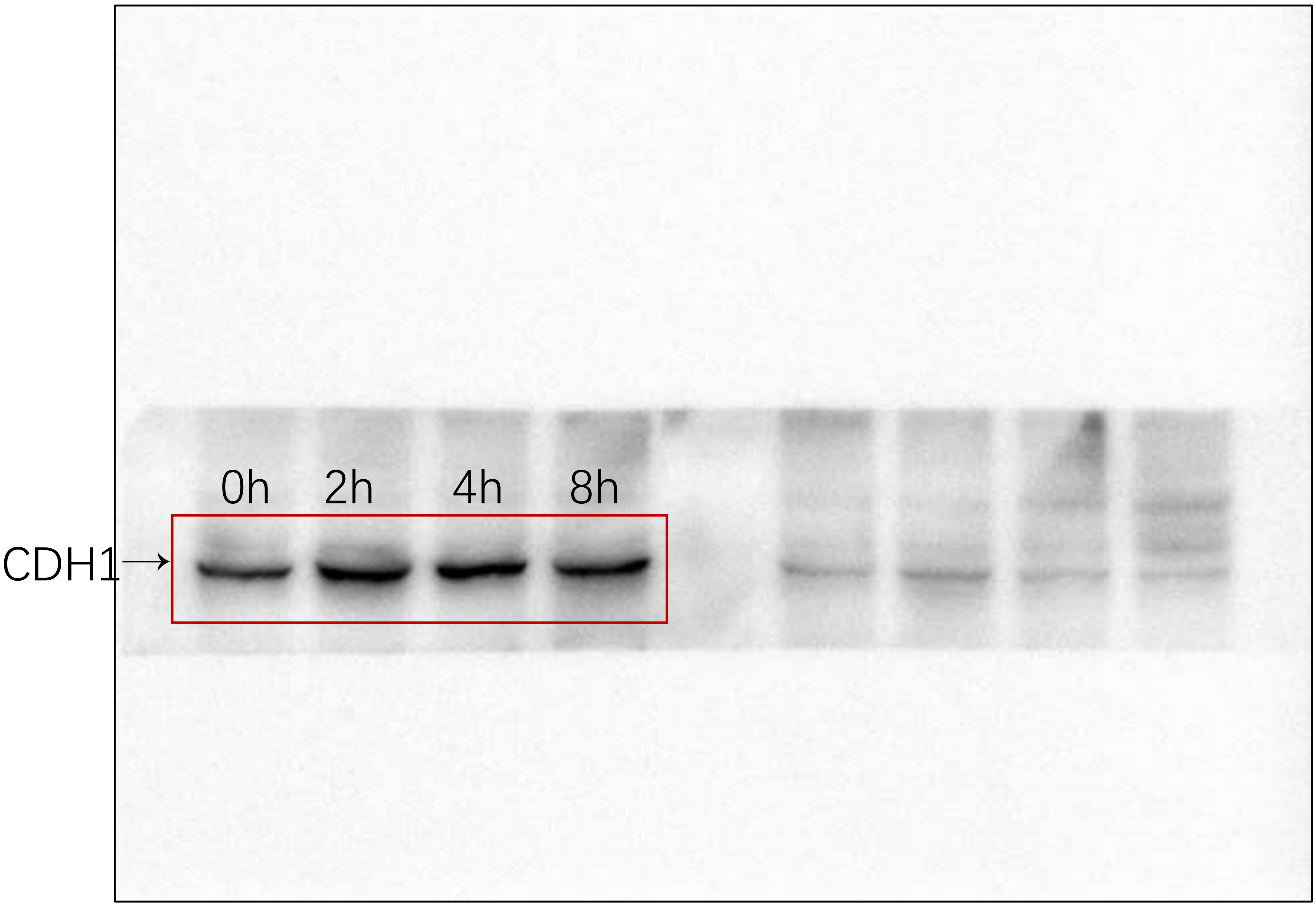

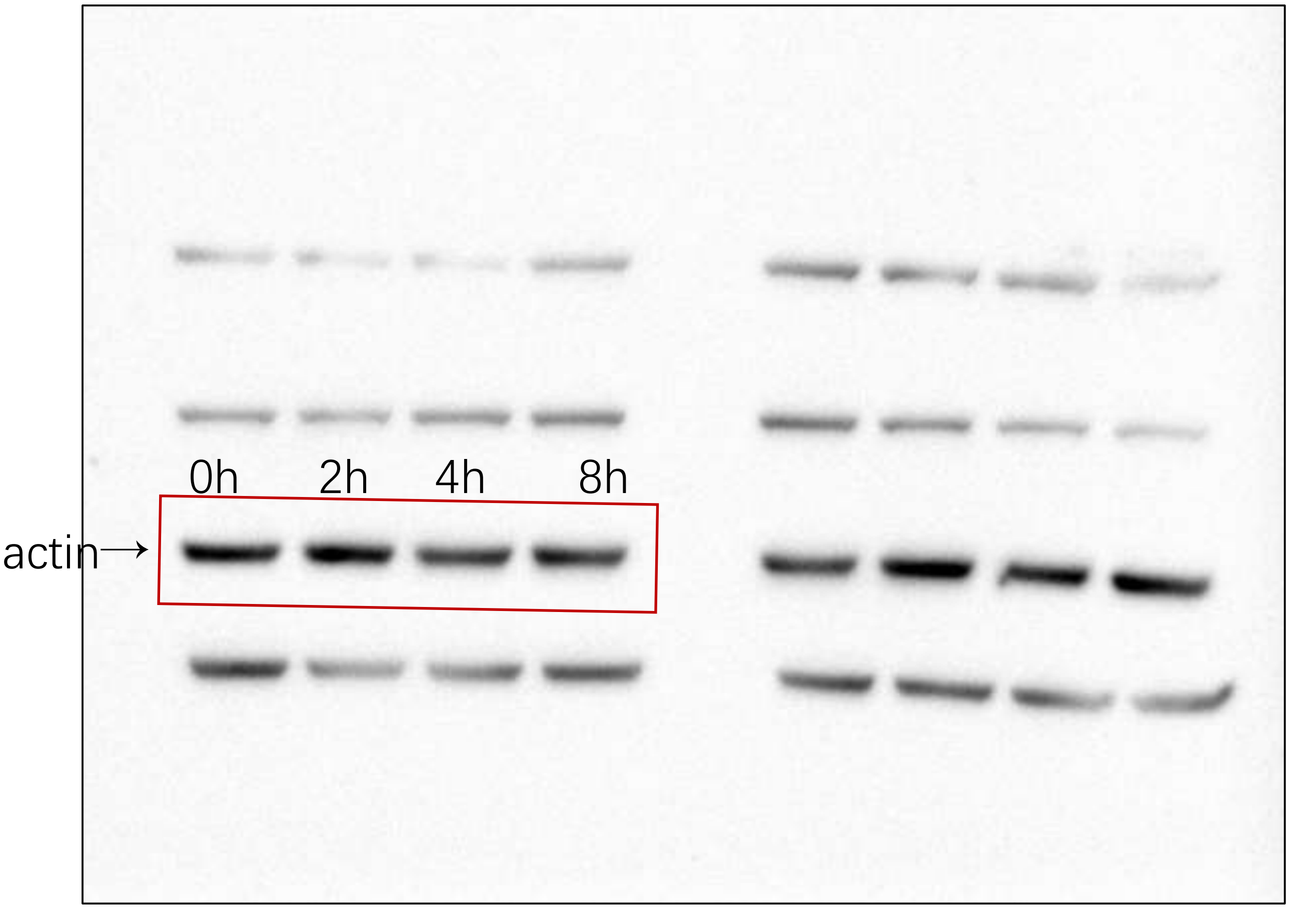

Original images of western blots of CDH1 short & long exposures in figure 5d ST6

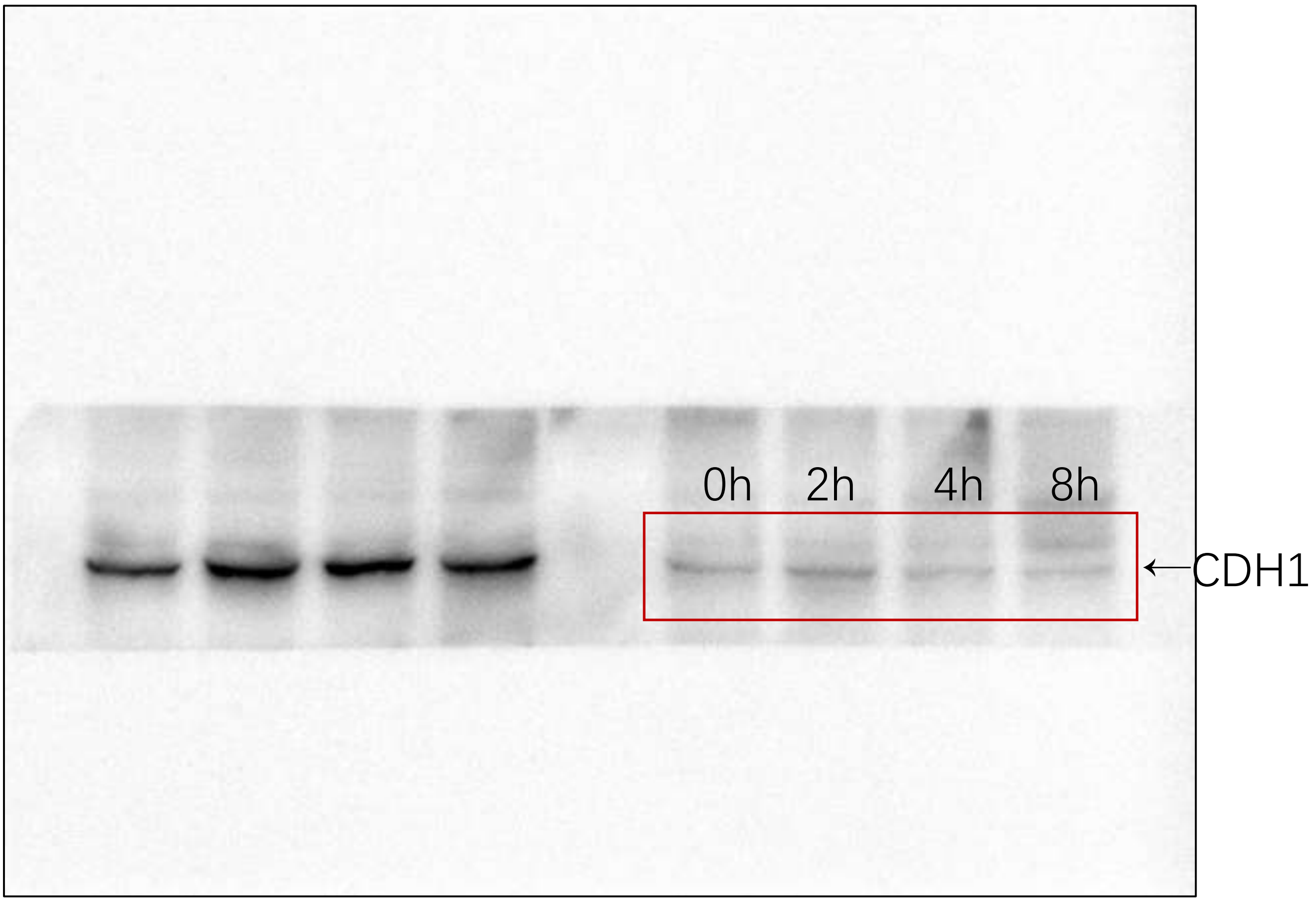

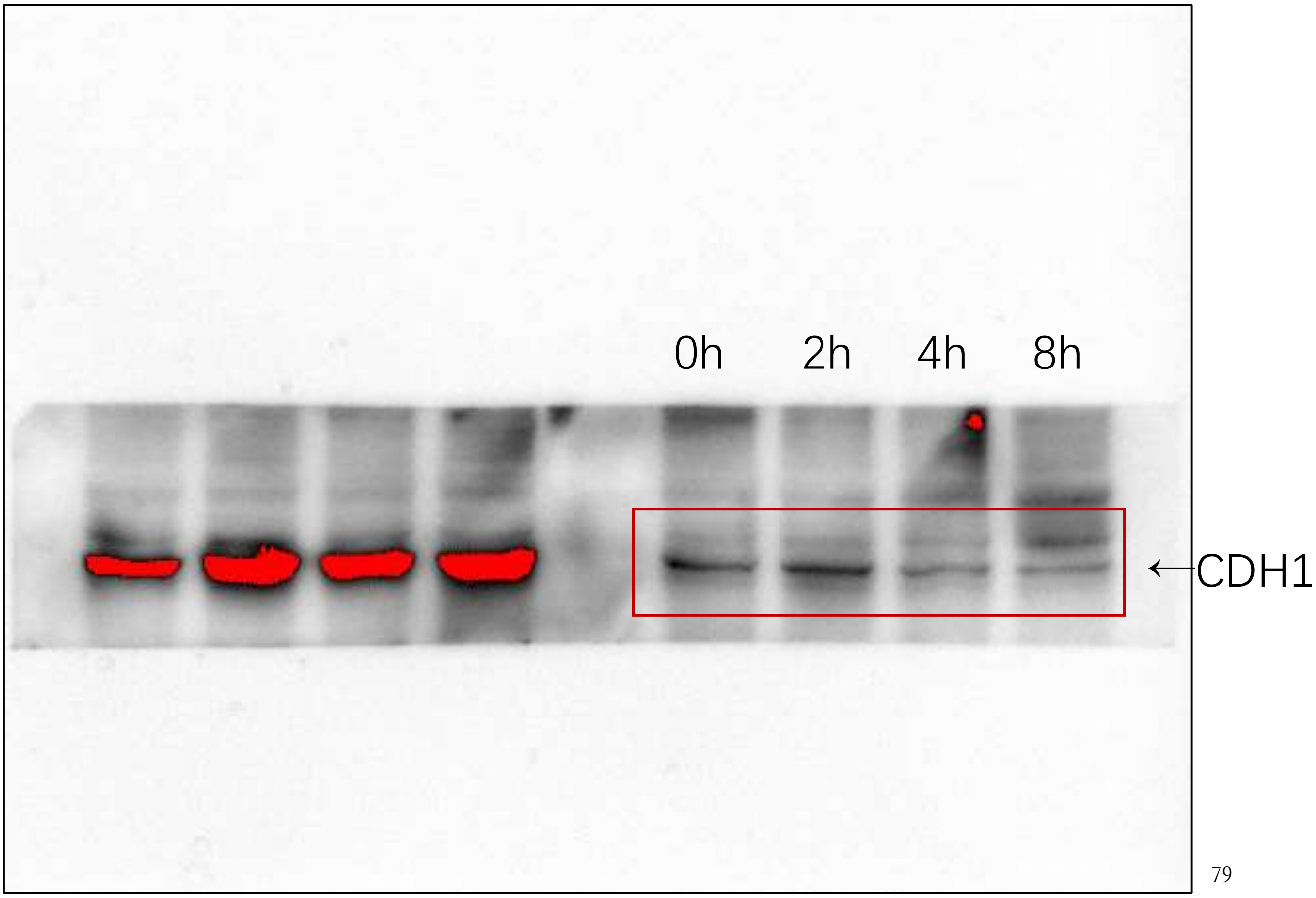

Original images of western blots of actin in figure 5d ST6

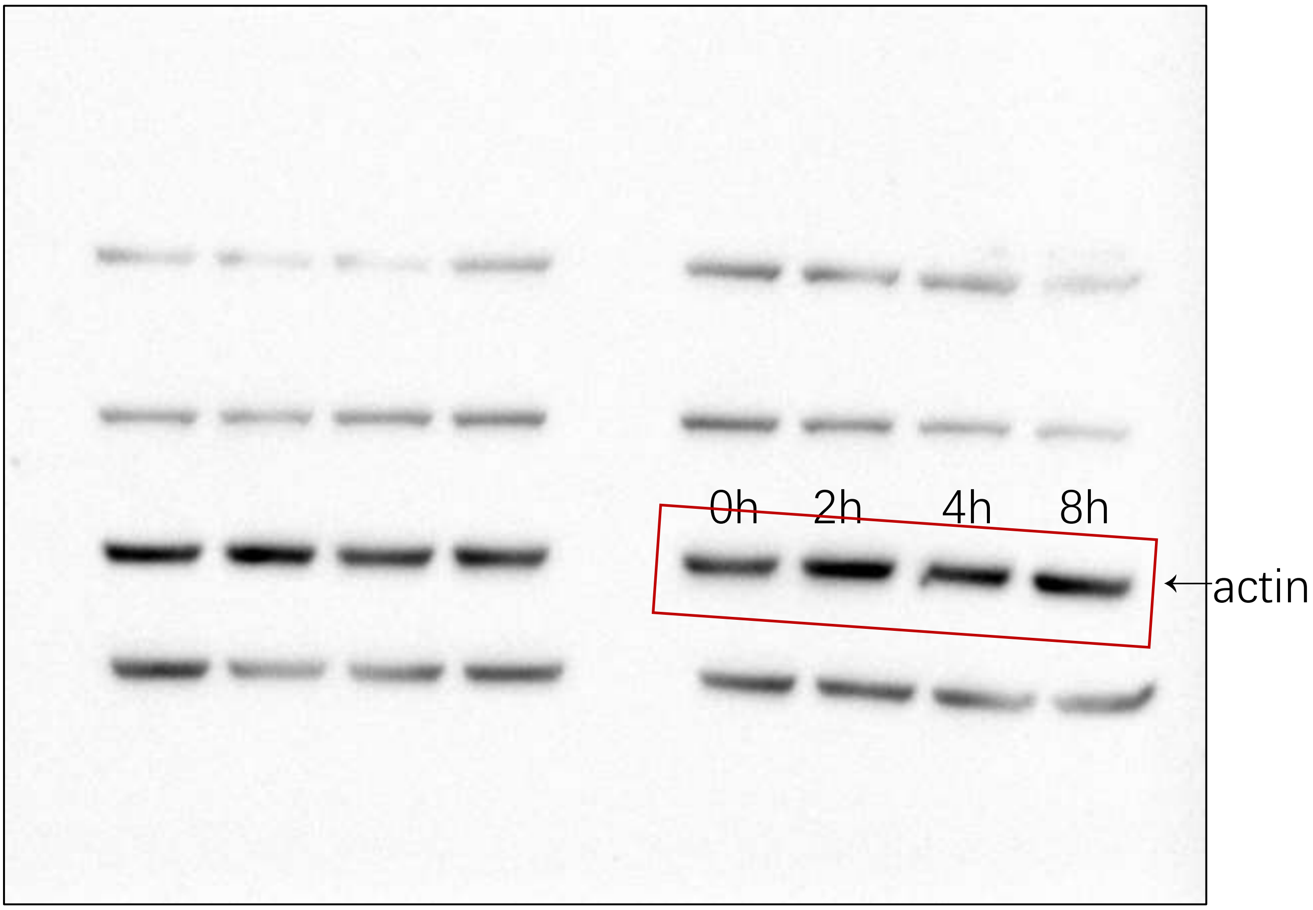

Original images of western blots of CDH1-HA and FLAG in figure 5g

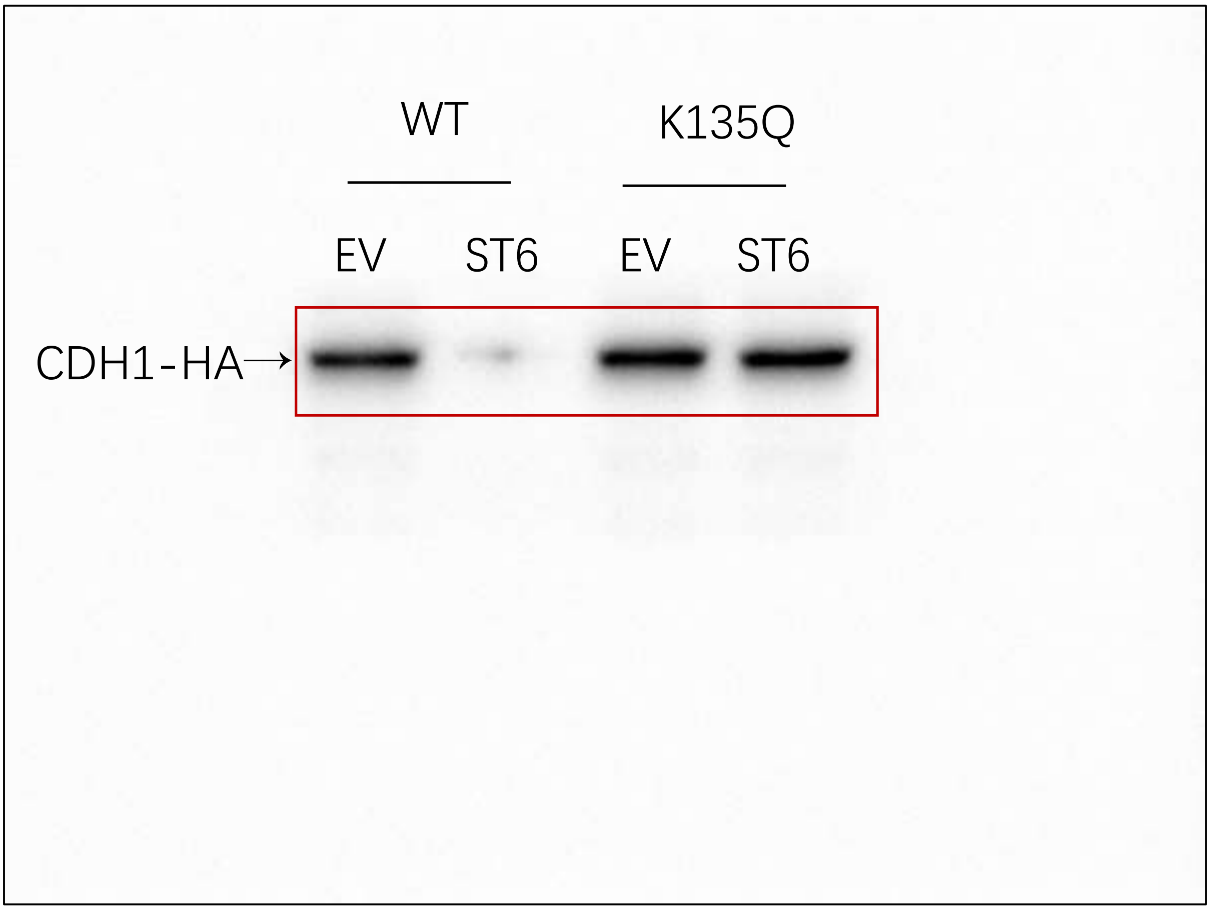

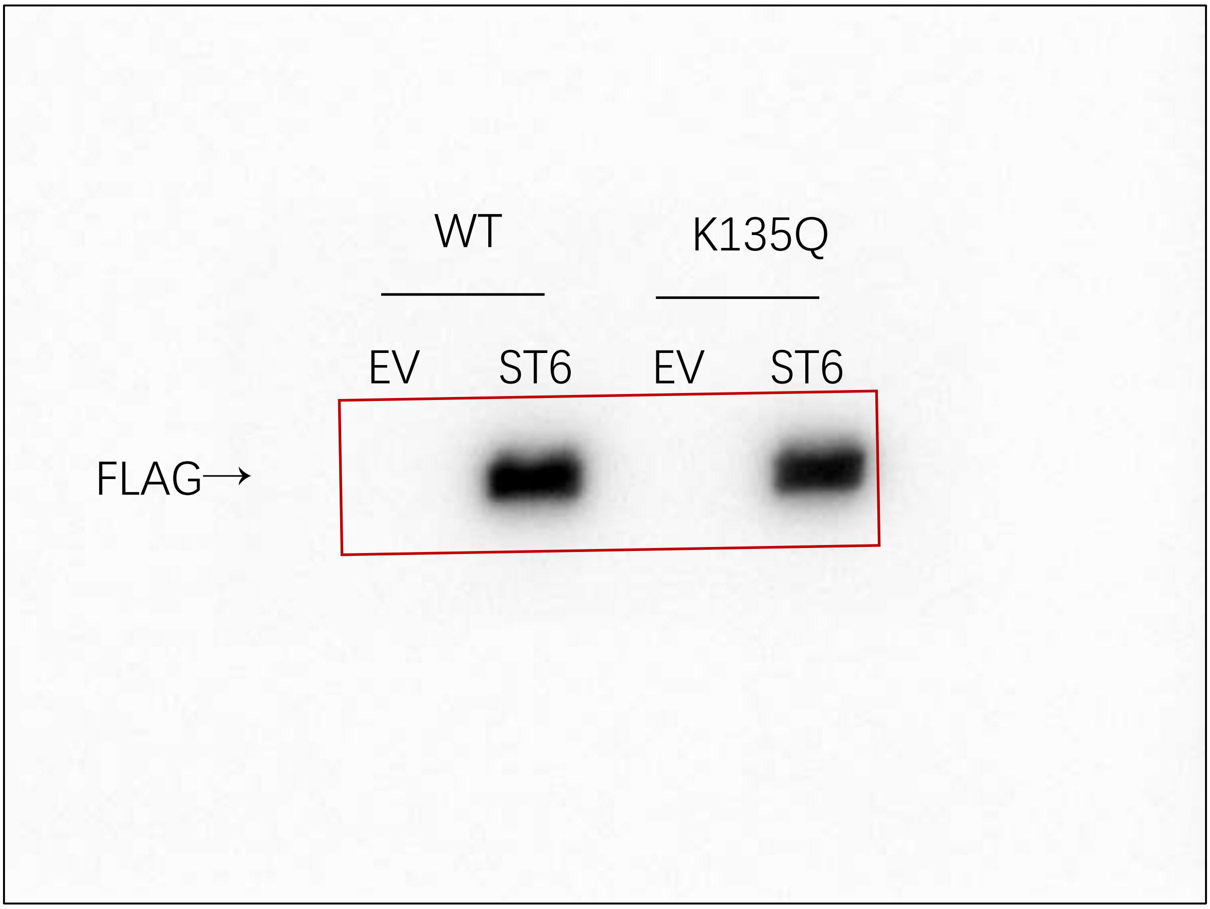

Original images of western blots of actin in figure 5g

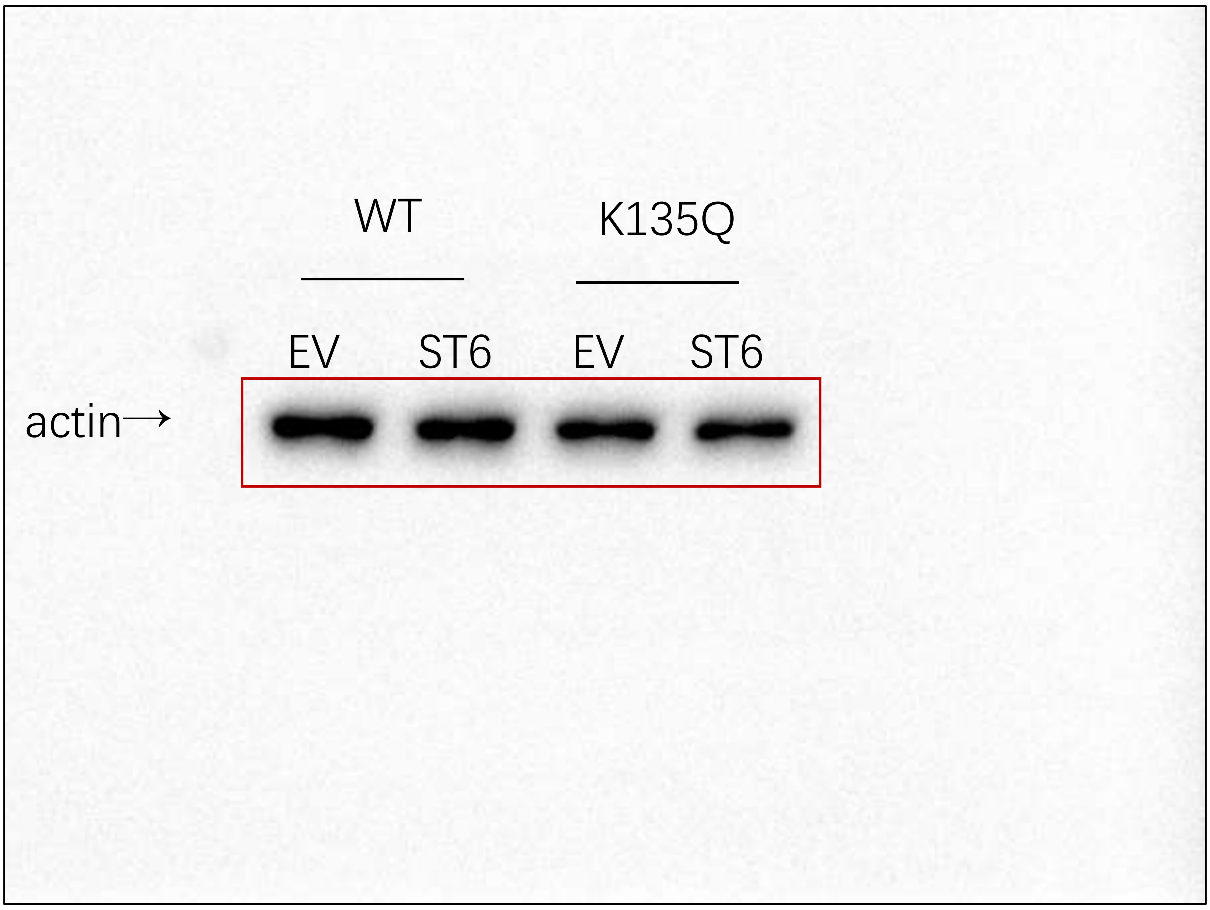

Original images of western blots of TPX2 & Cyc B1 in figure 6a 293T

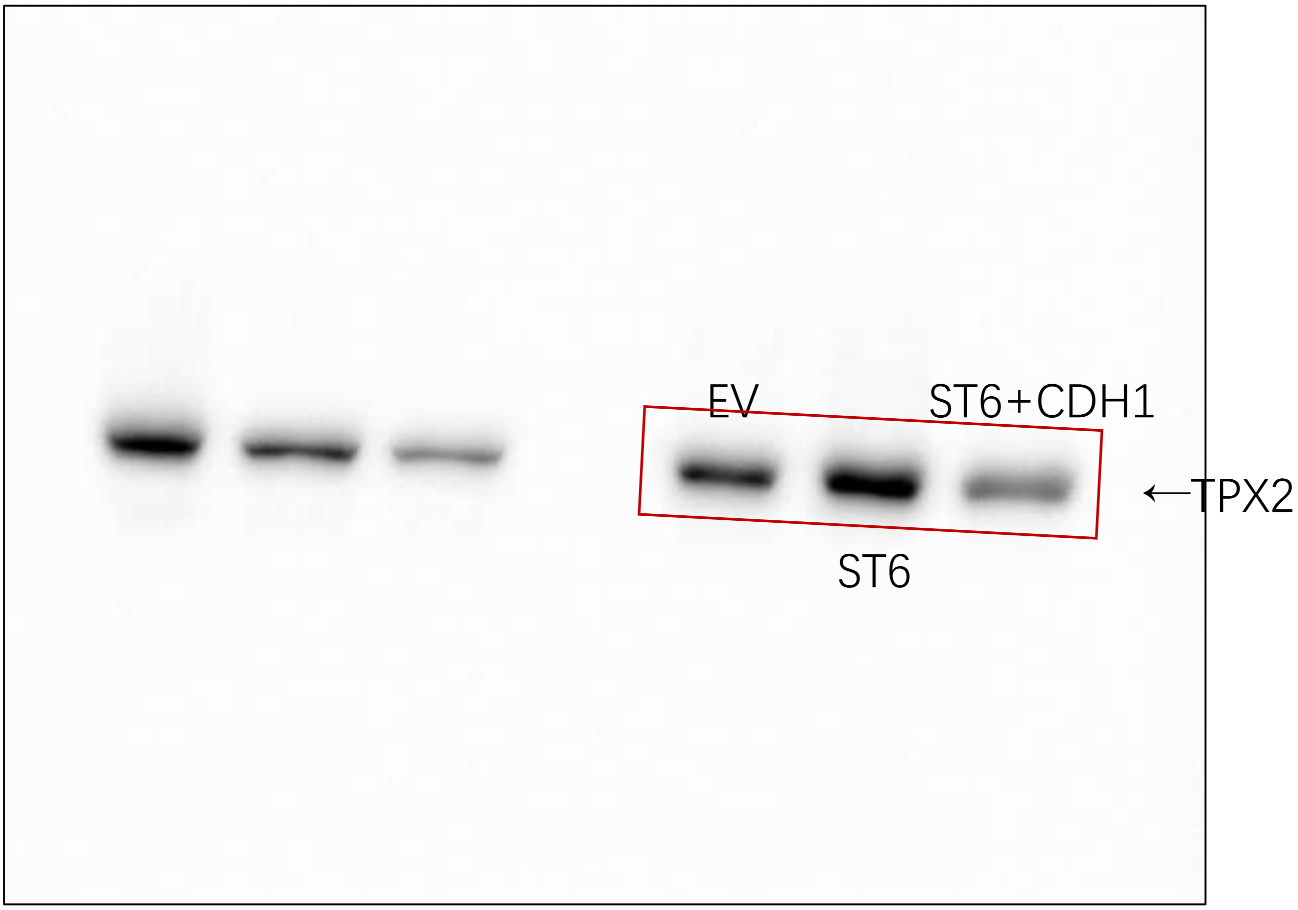

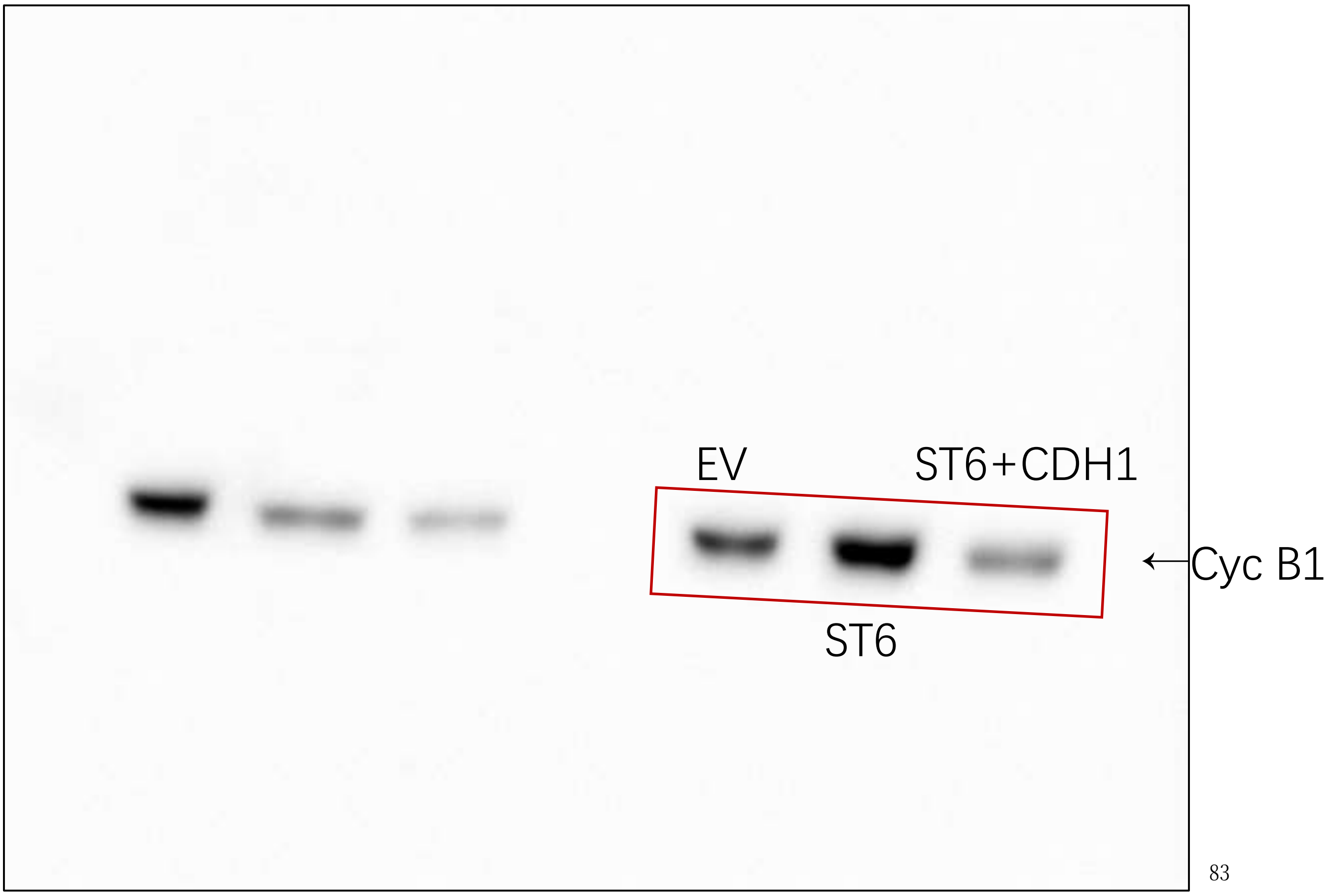

Original images of western blots of AURKA & AURKB in figure 6a 293T

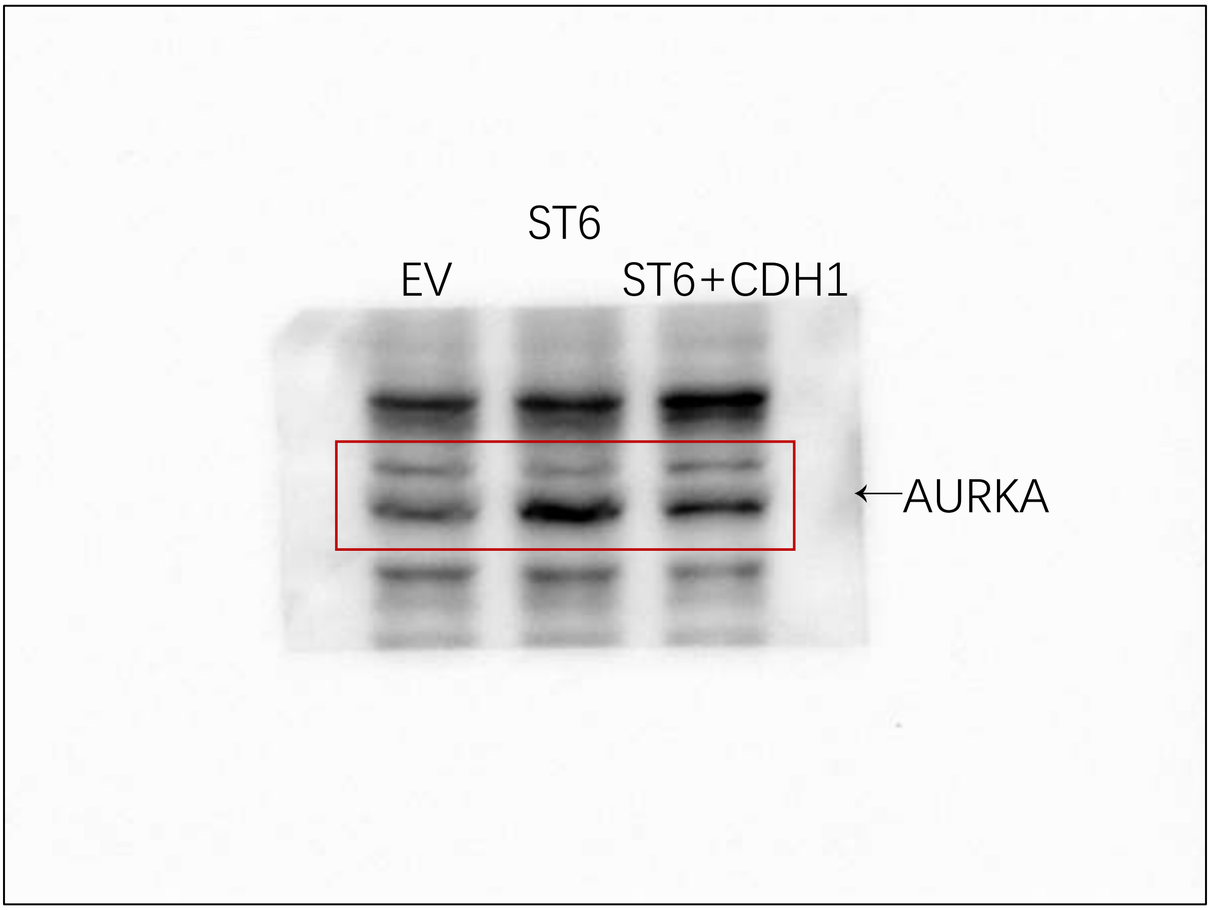

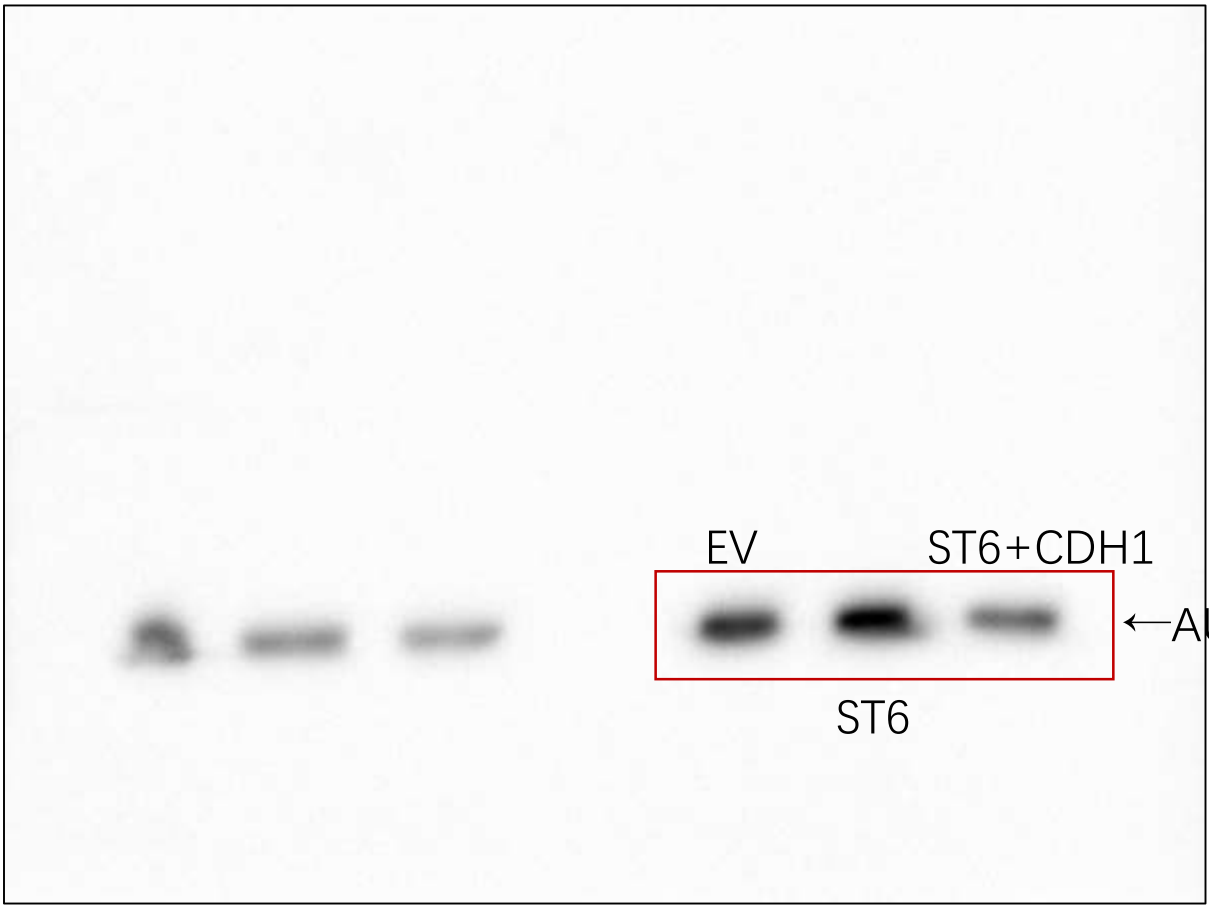

Original images of western blots of securin & FLAG in figure 6a 293T

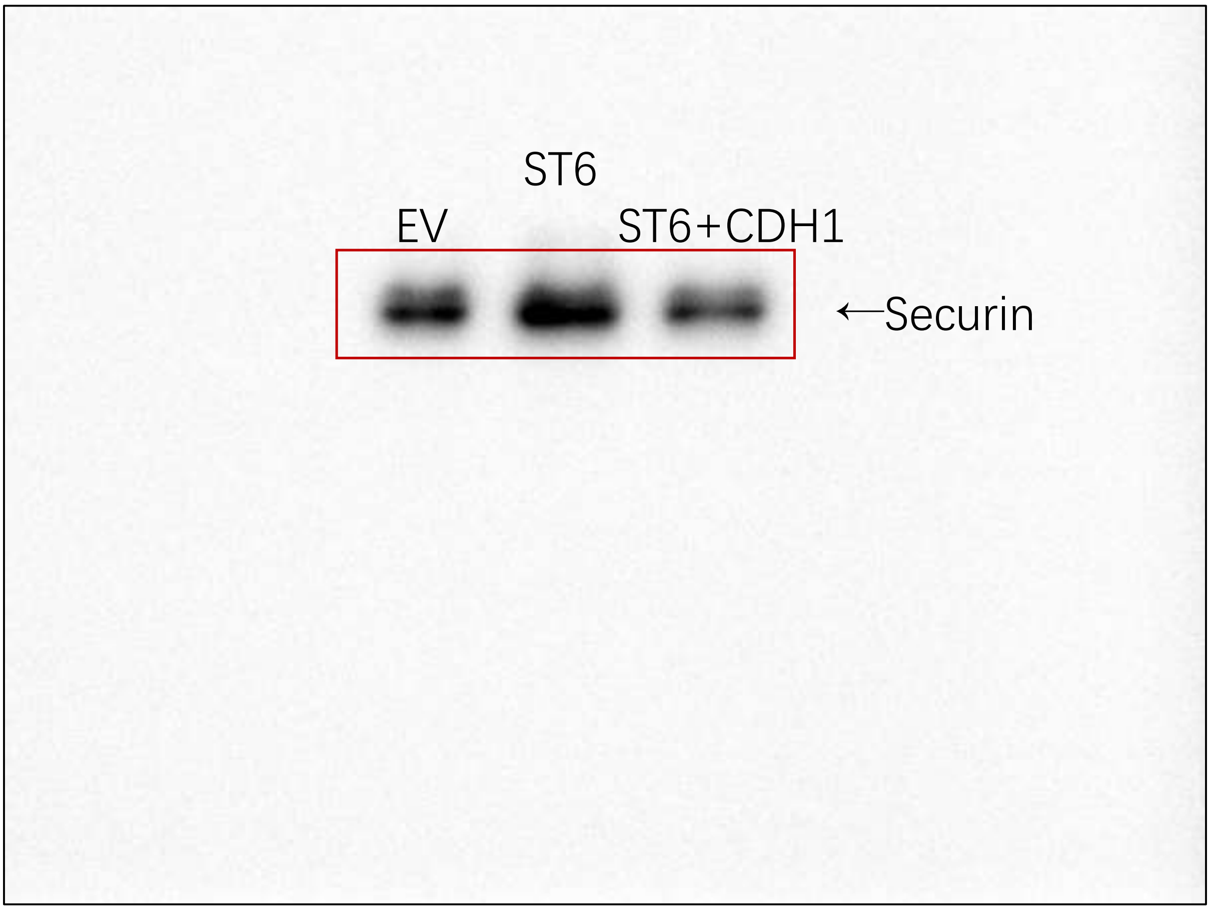

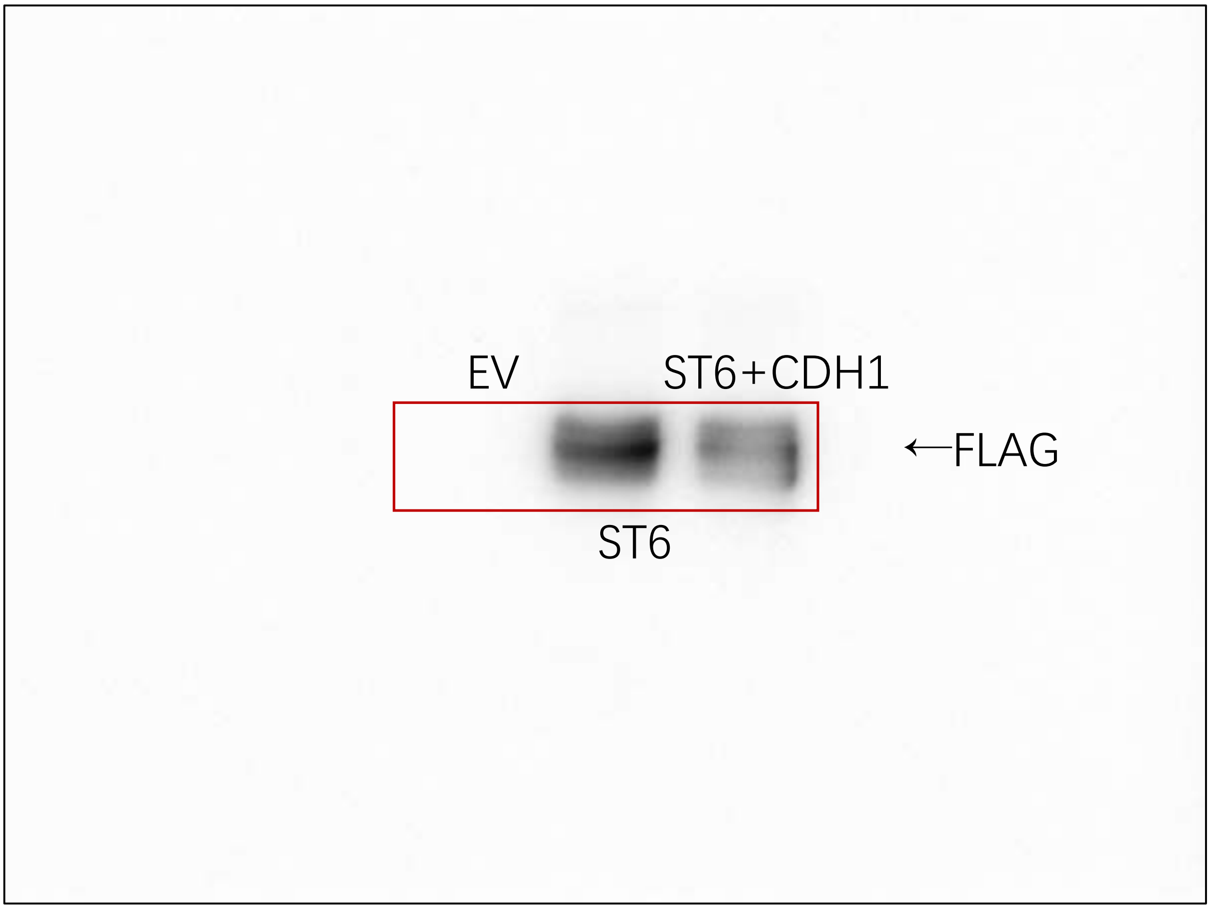

Original images of western blots of CDH1 & actin in figure 6a 293T

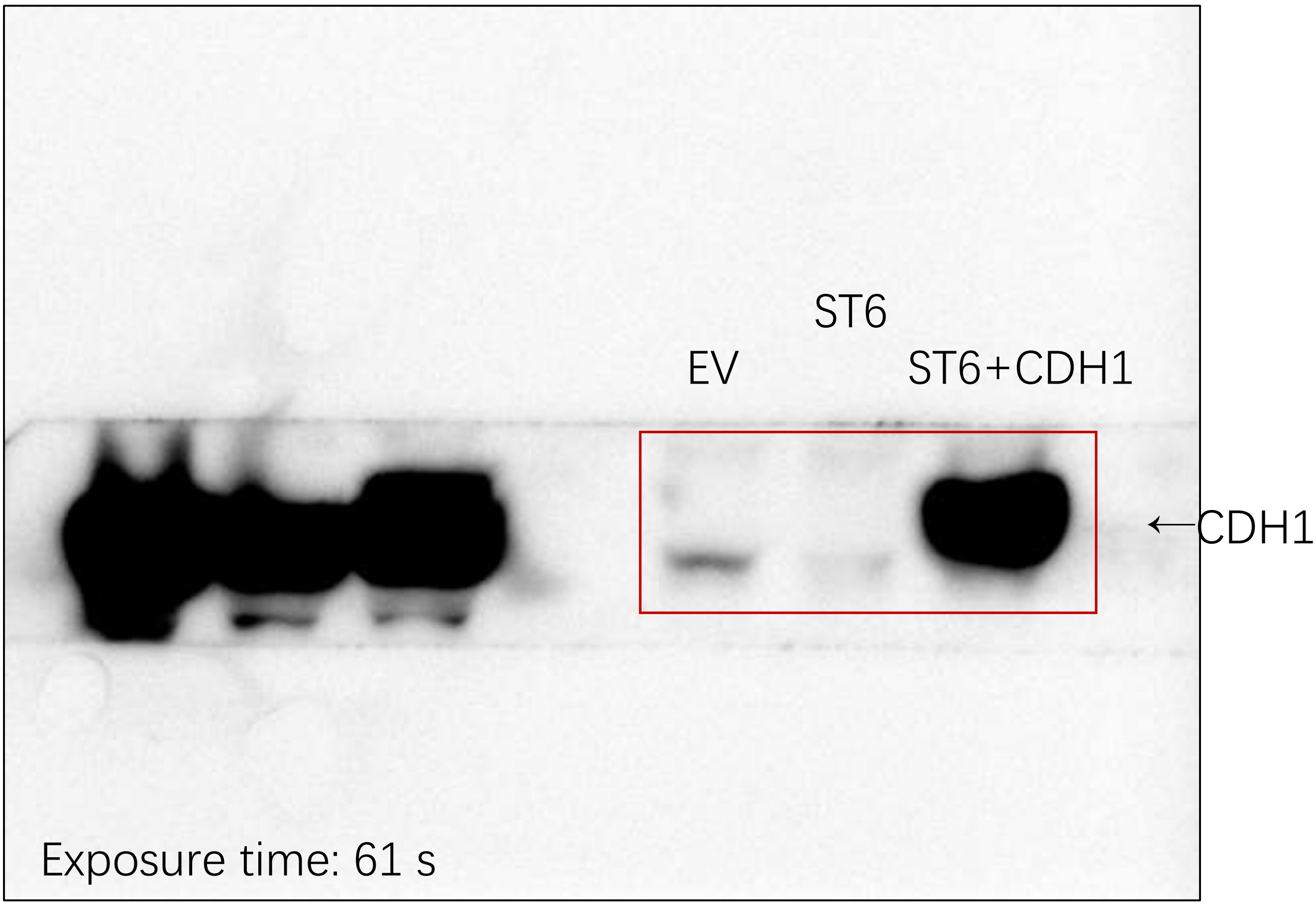

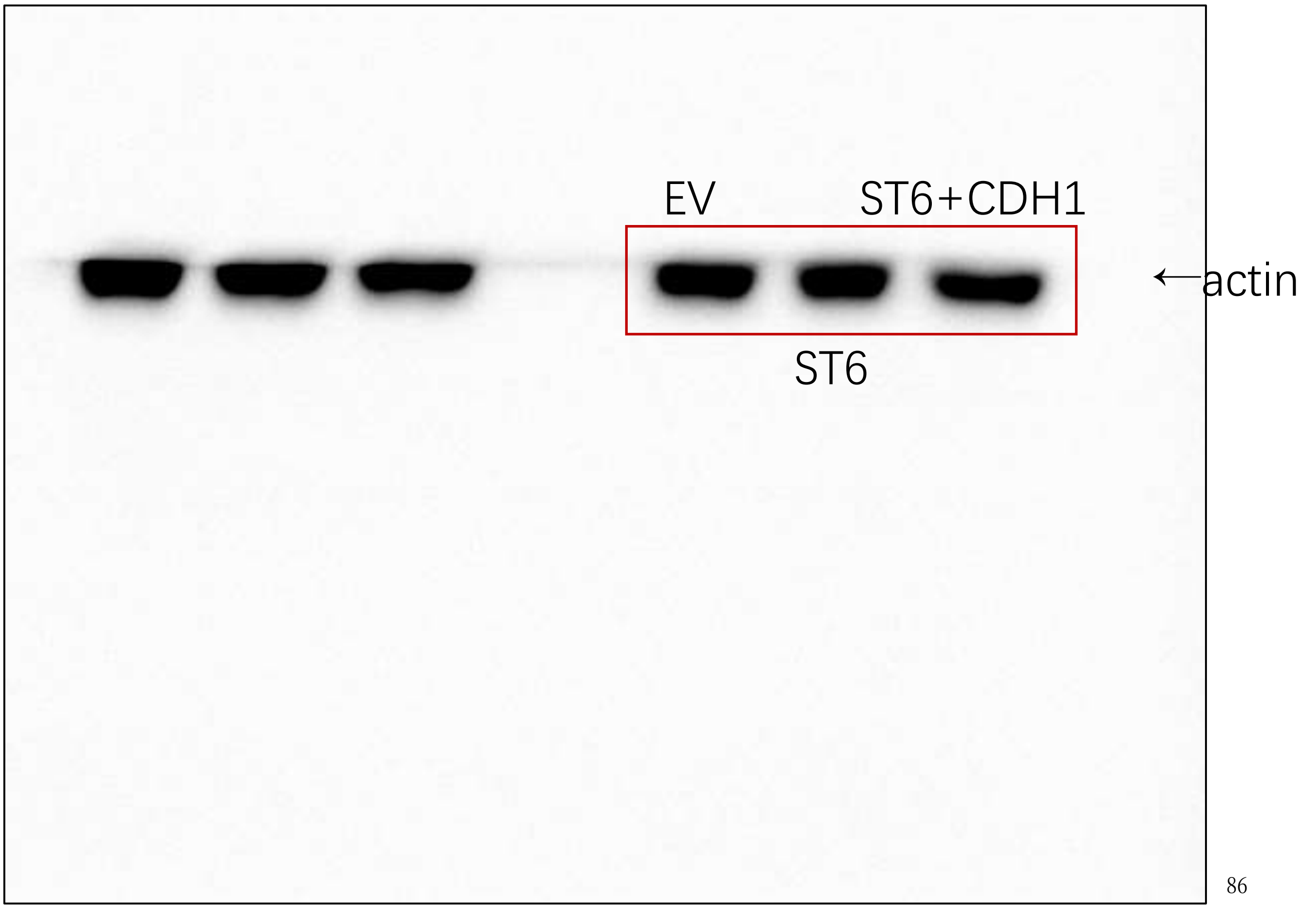

Original images of western blots of CDH1 in figure 6a 293T with short exposure time

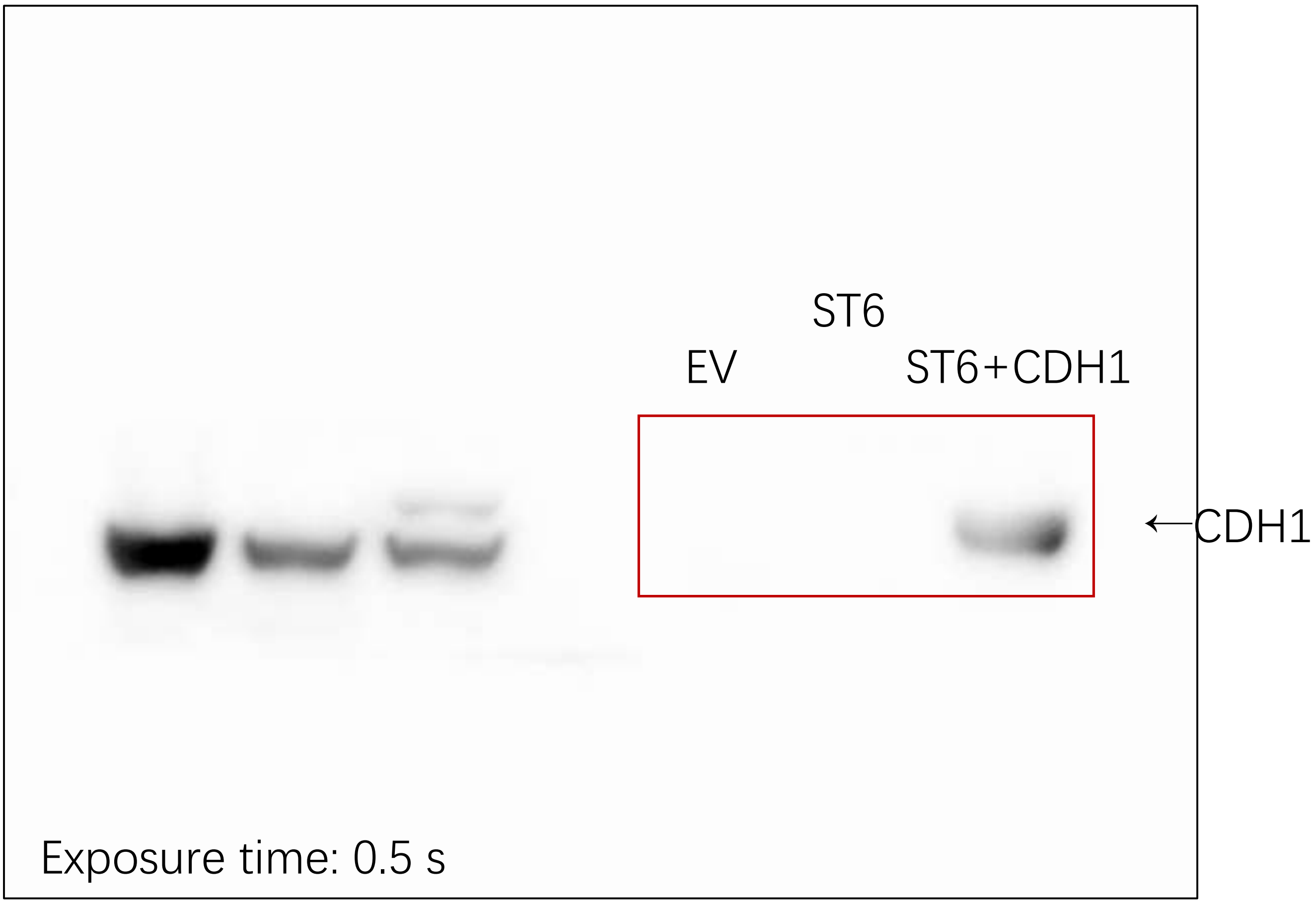

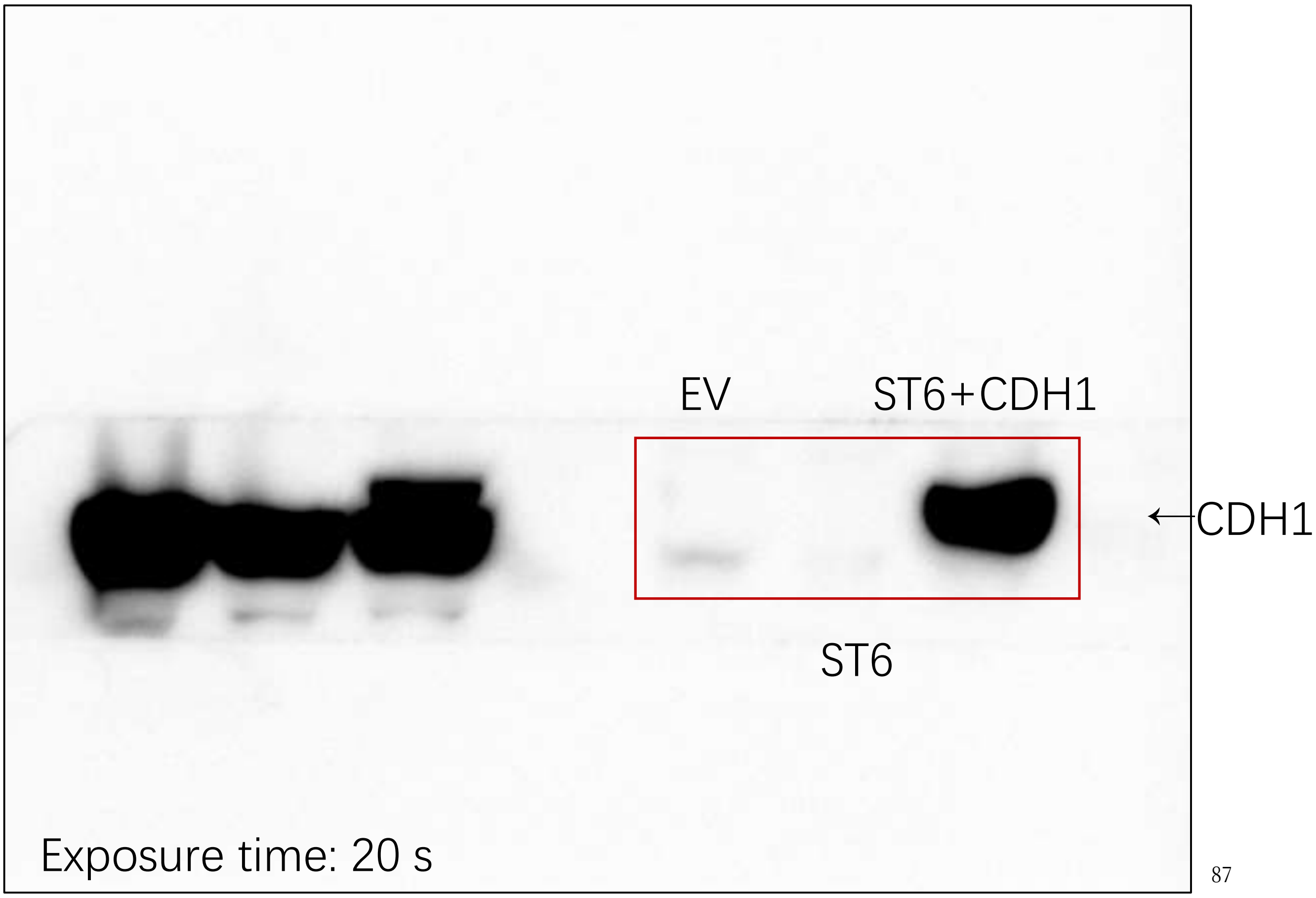

Original images of western blots of TPX2 & Cyc B1 in figure 6a HeLa

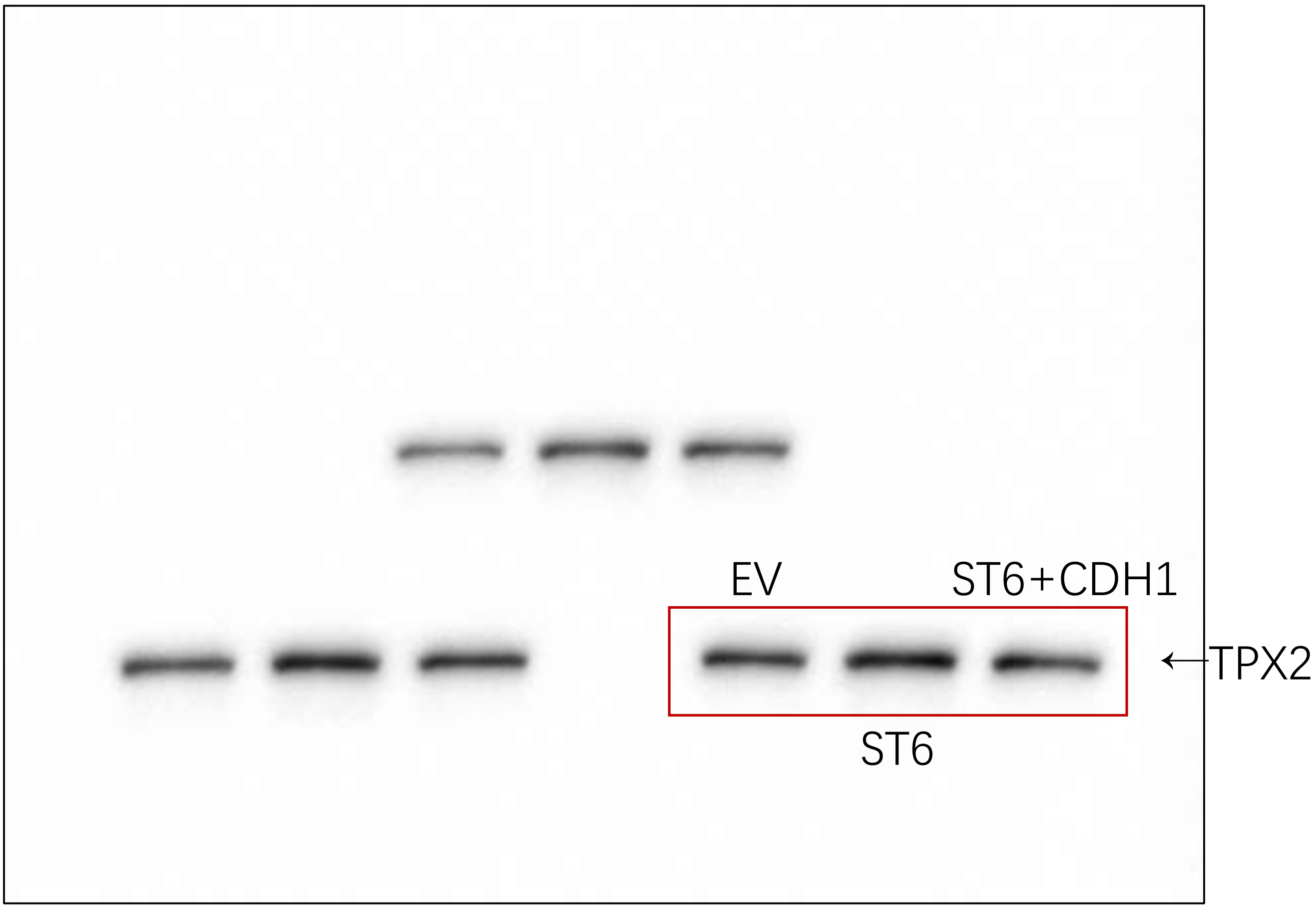

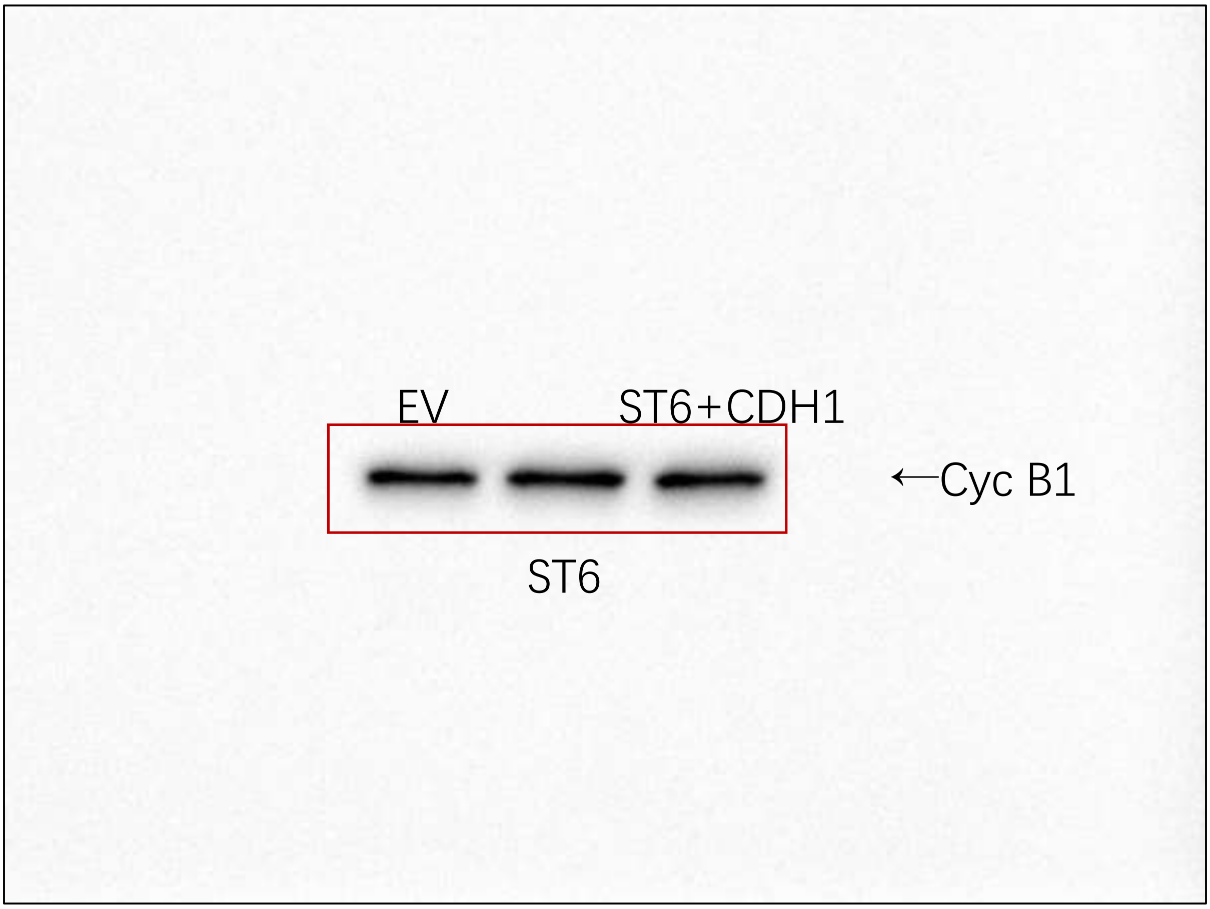

Original images of western blots of AURKA & AURKB in figure 6a HeLa

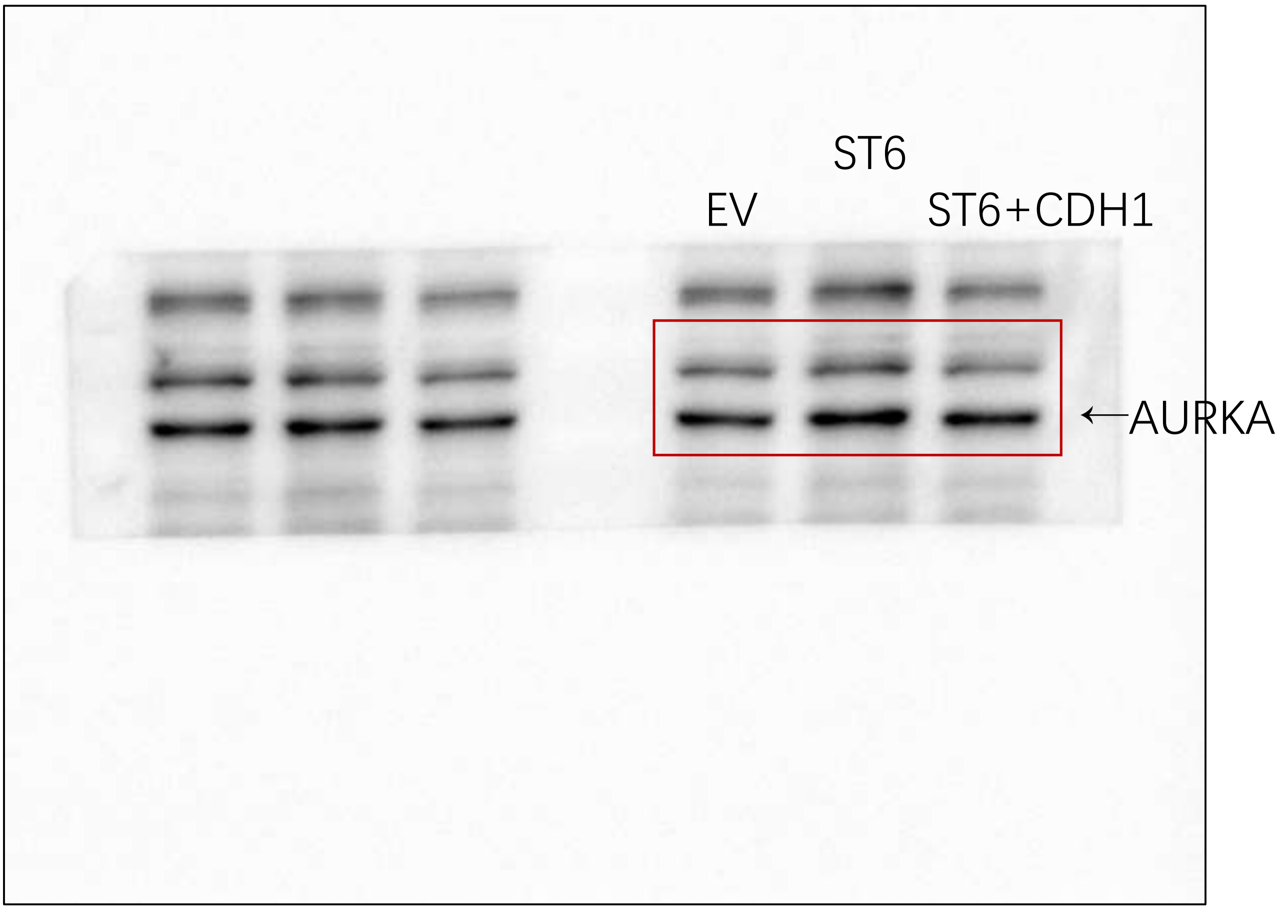

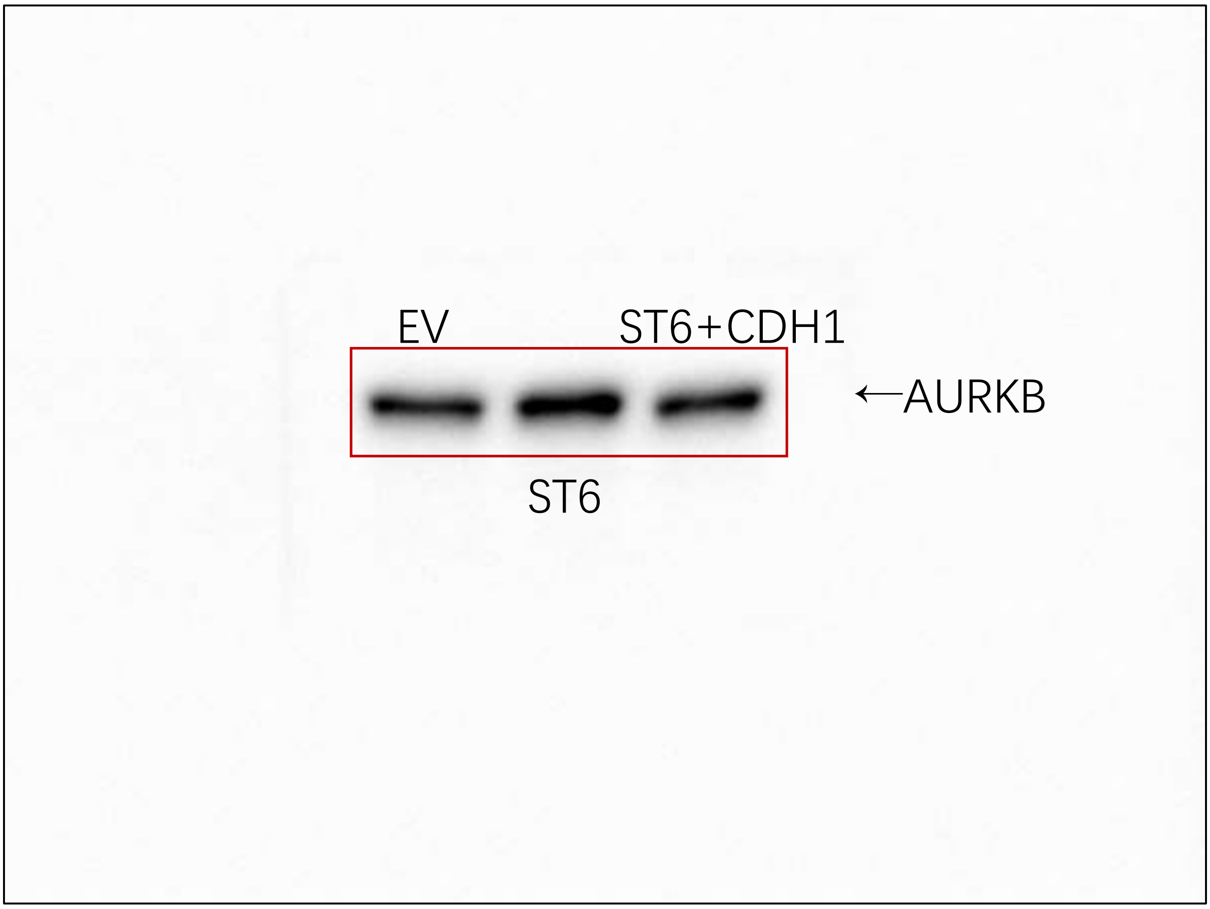

Original images of western blots of securin & FLAG in figure 6a HeLa

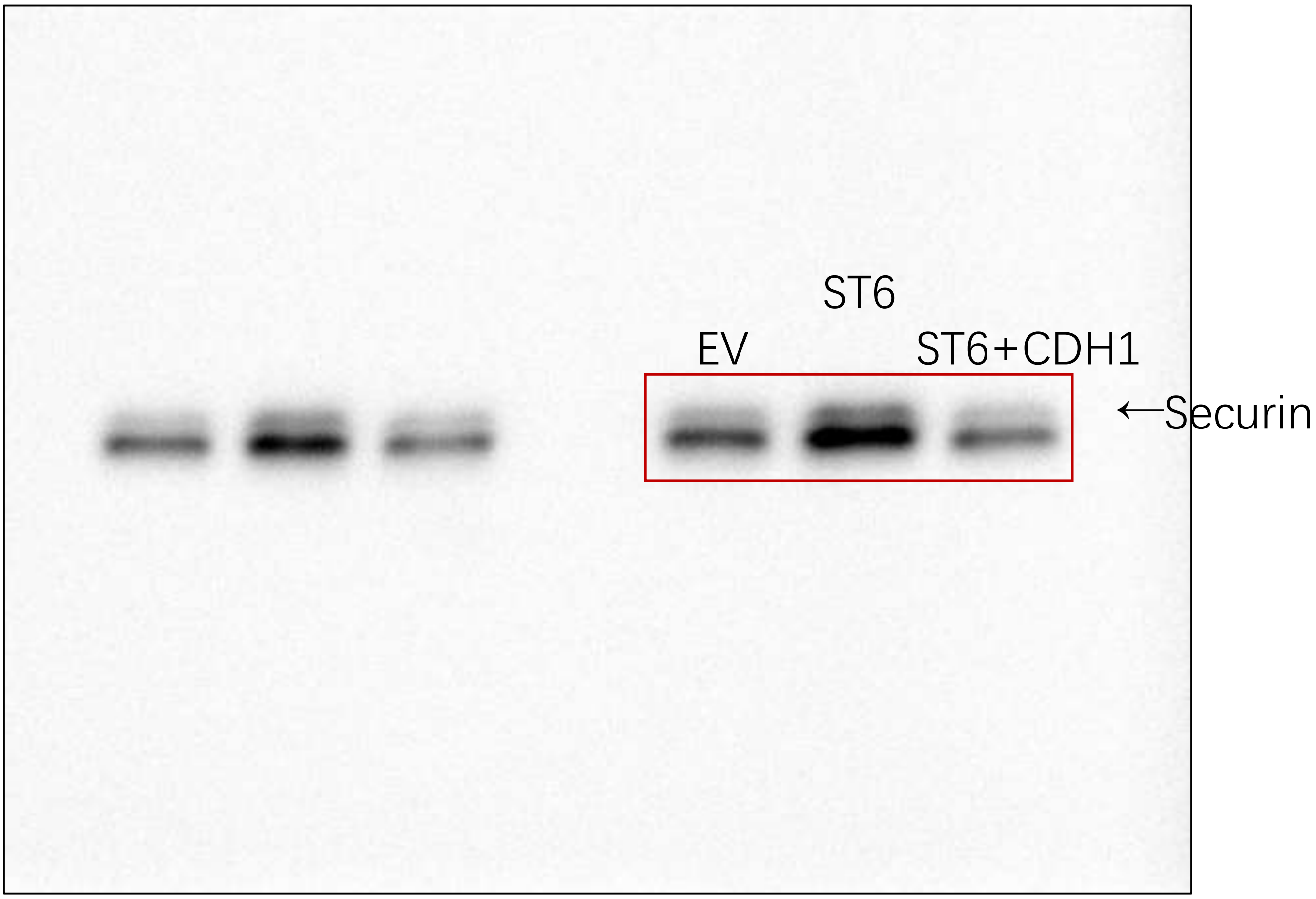

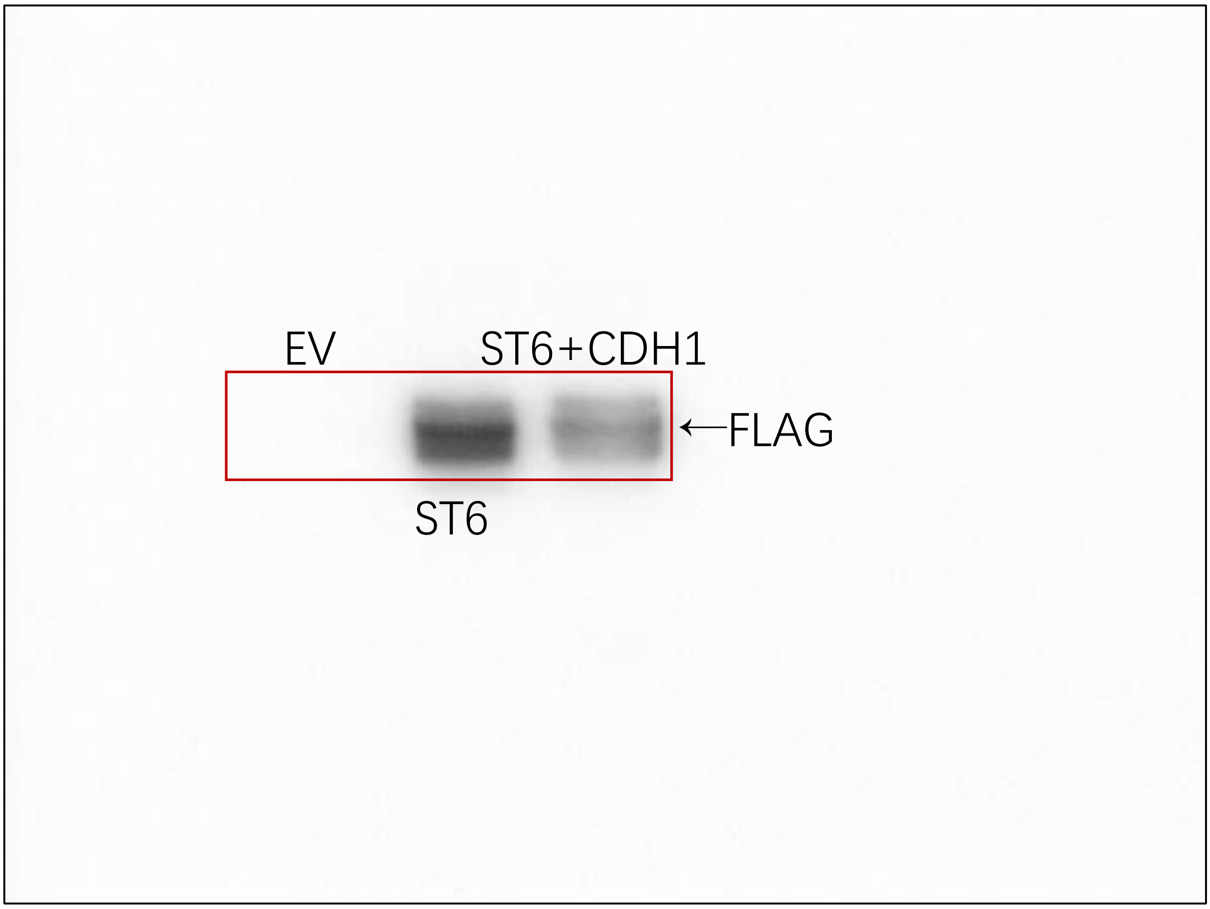

Original images of western blots of CDH1 & actin in figure 6a HeLa

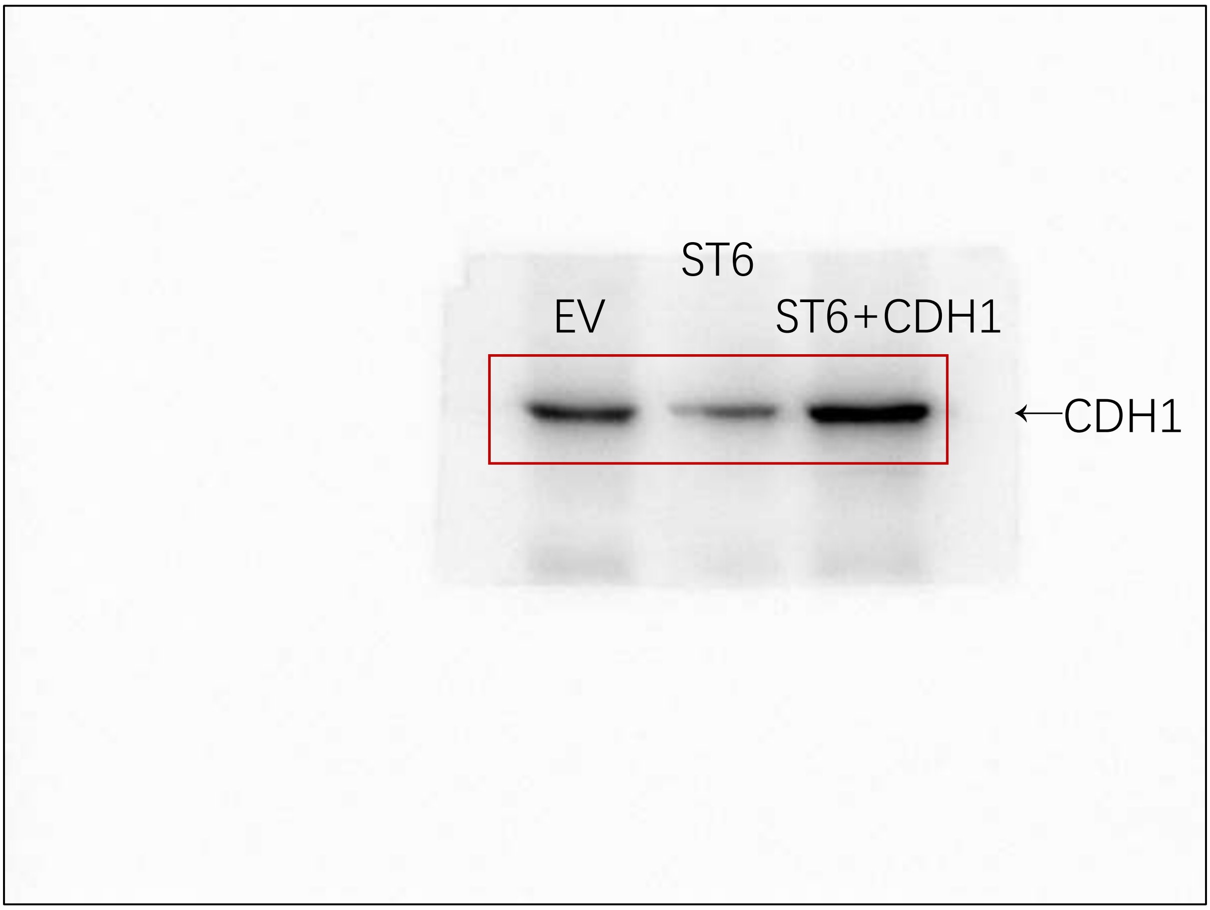

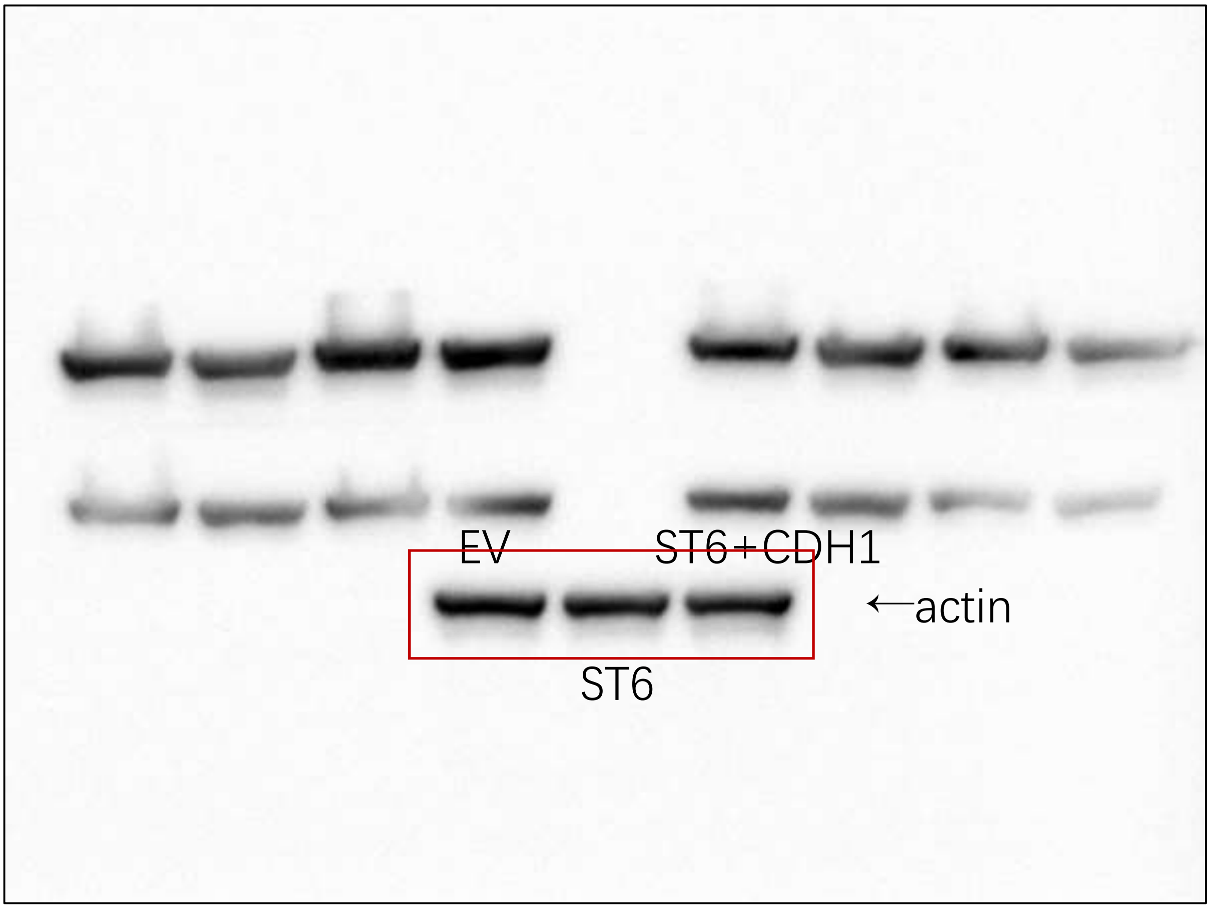

Original images of western blots of SIRT6 & GAPDH in figure S2

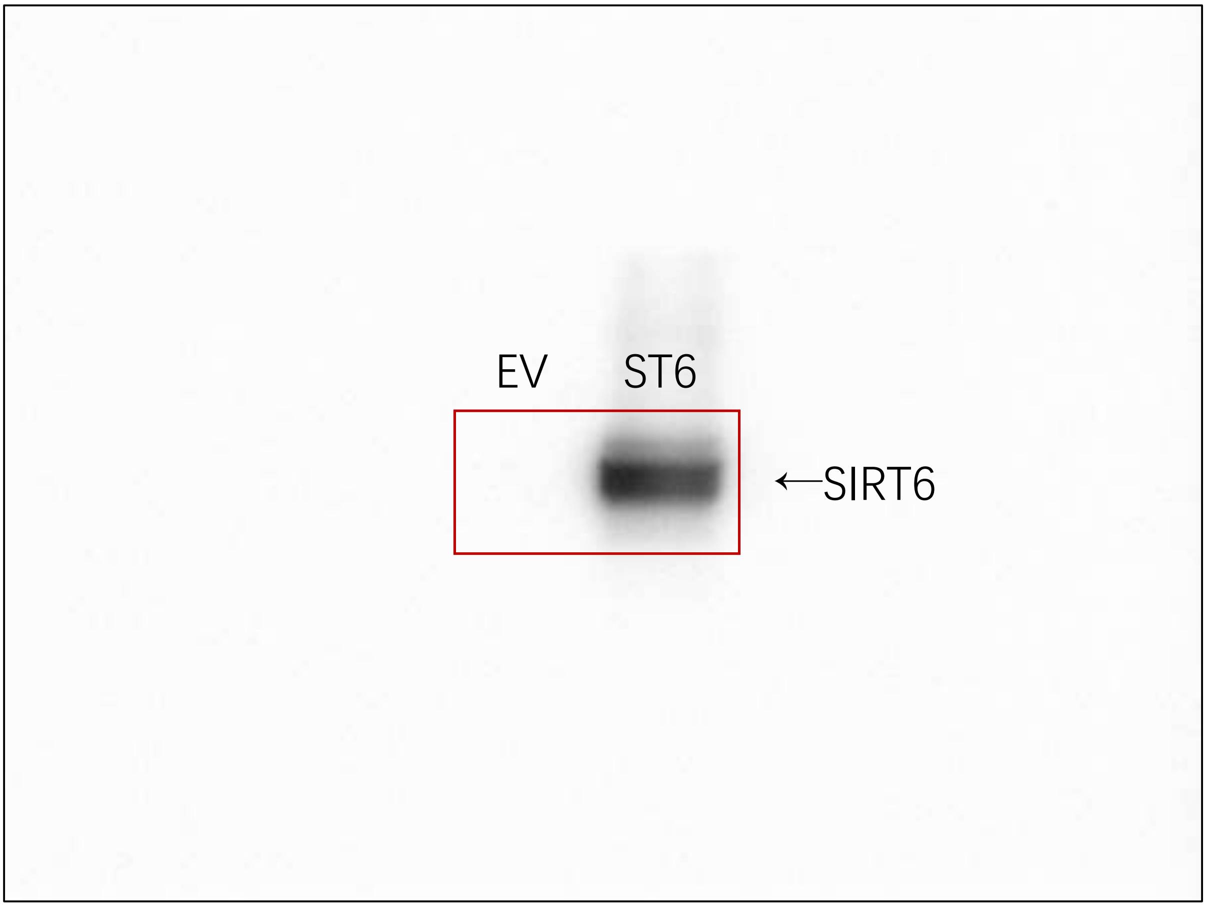

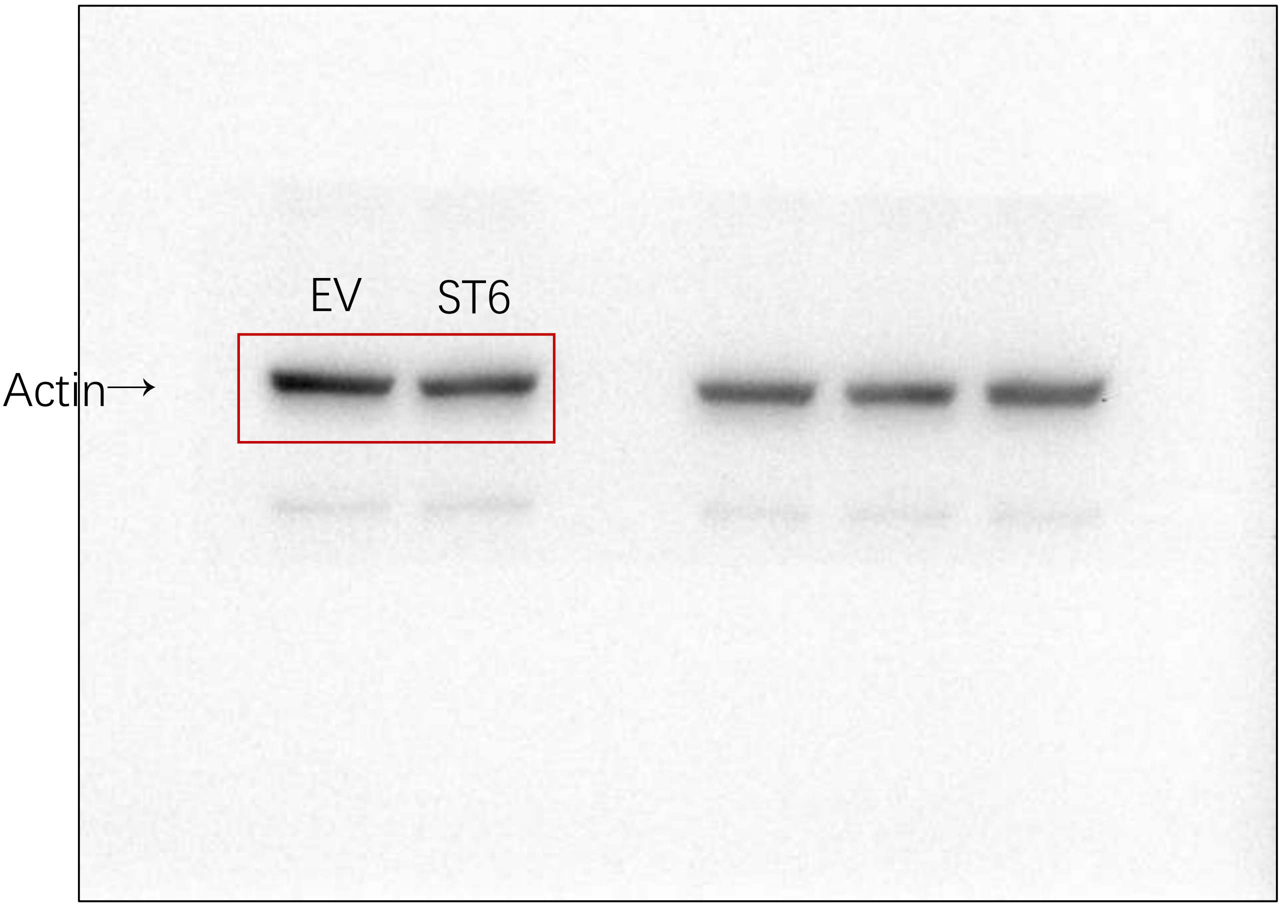

Original images of western blots of CDC20 & FLAG in figure S7 293T

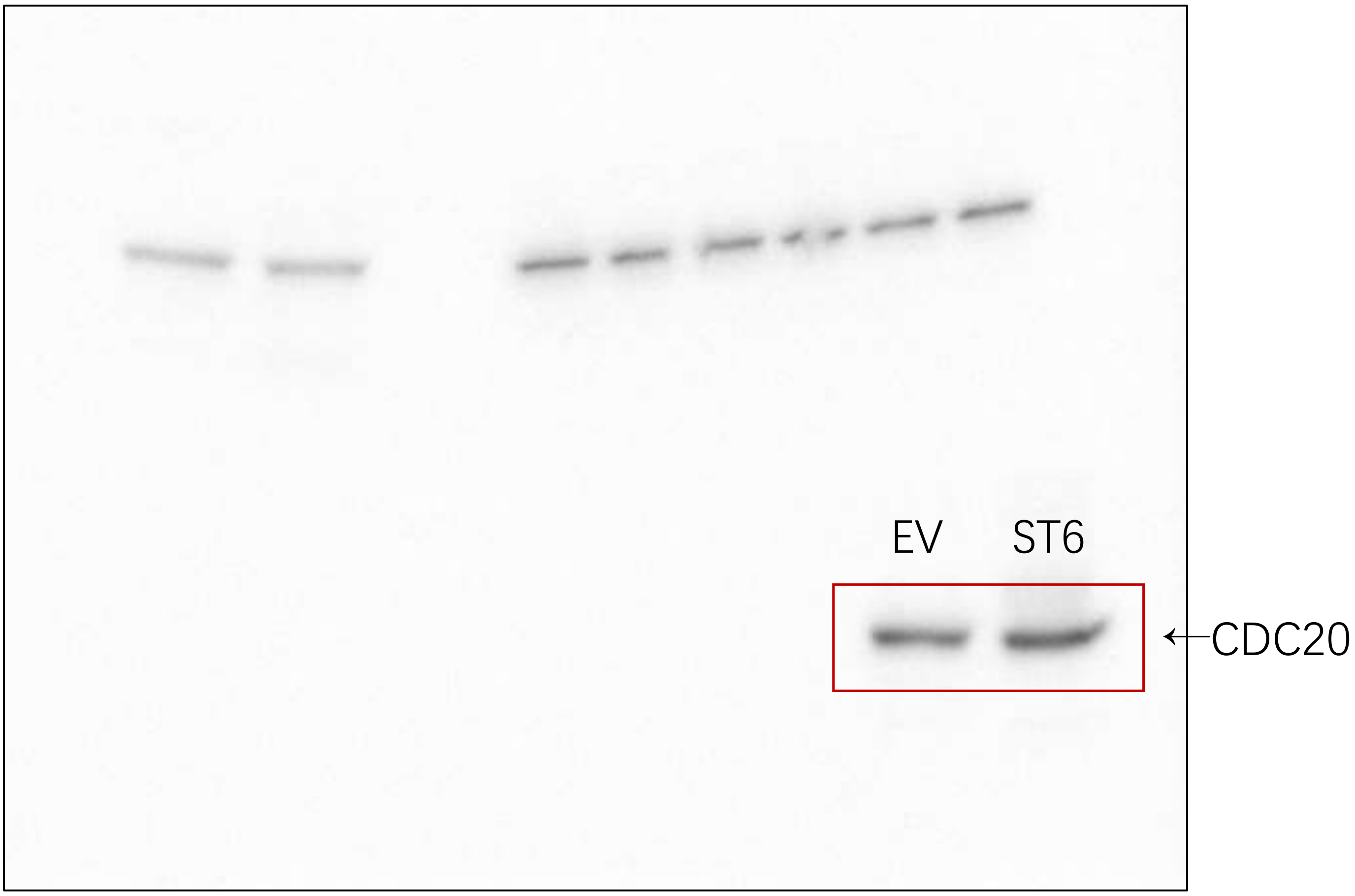

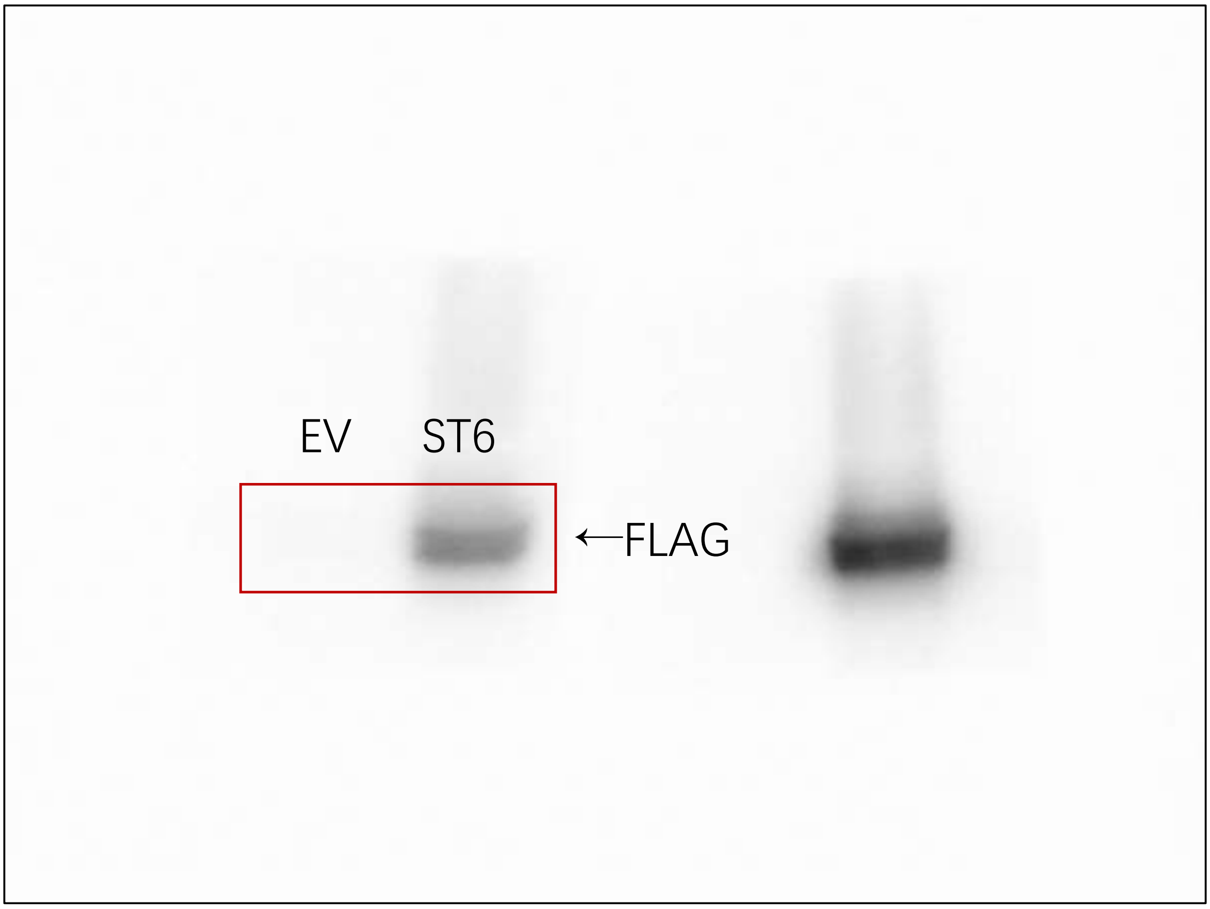

Original images of western blots of actin in figure S7 293T

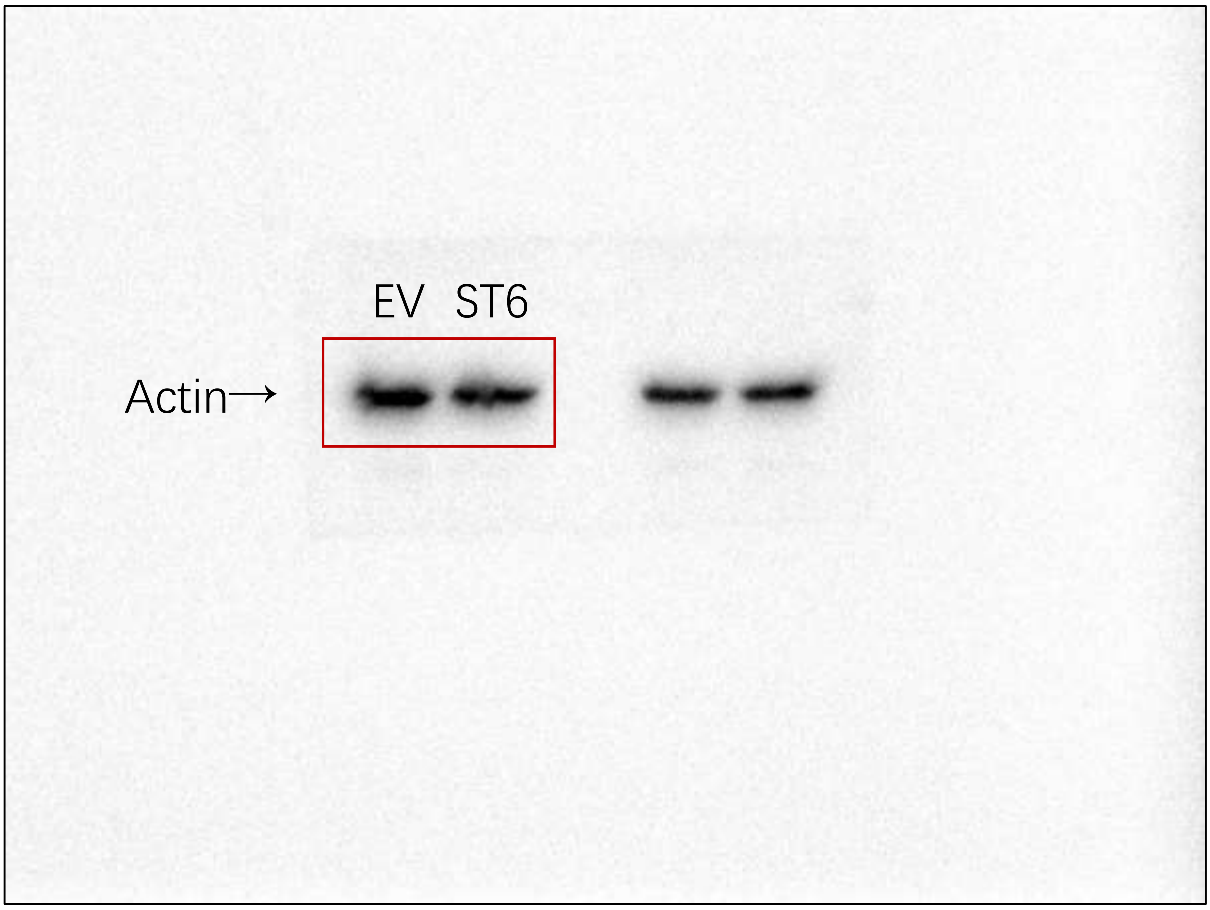

Original images of western blots of CDC20 & FLAG in figure S7 HeLa

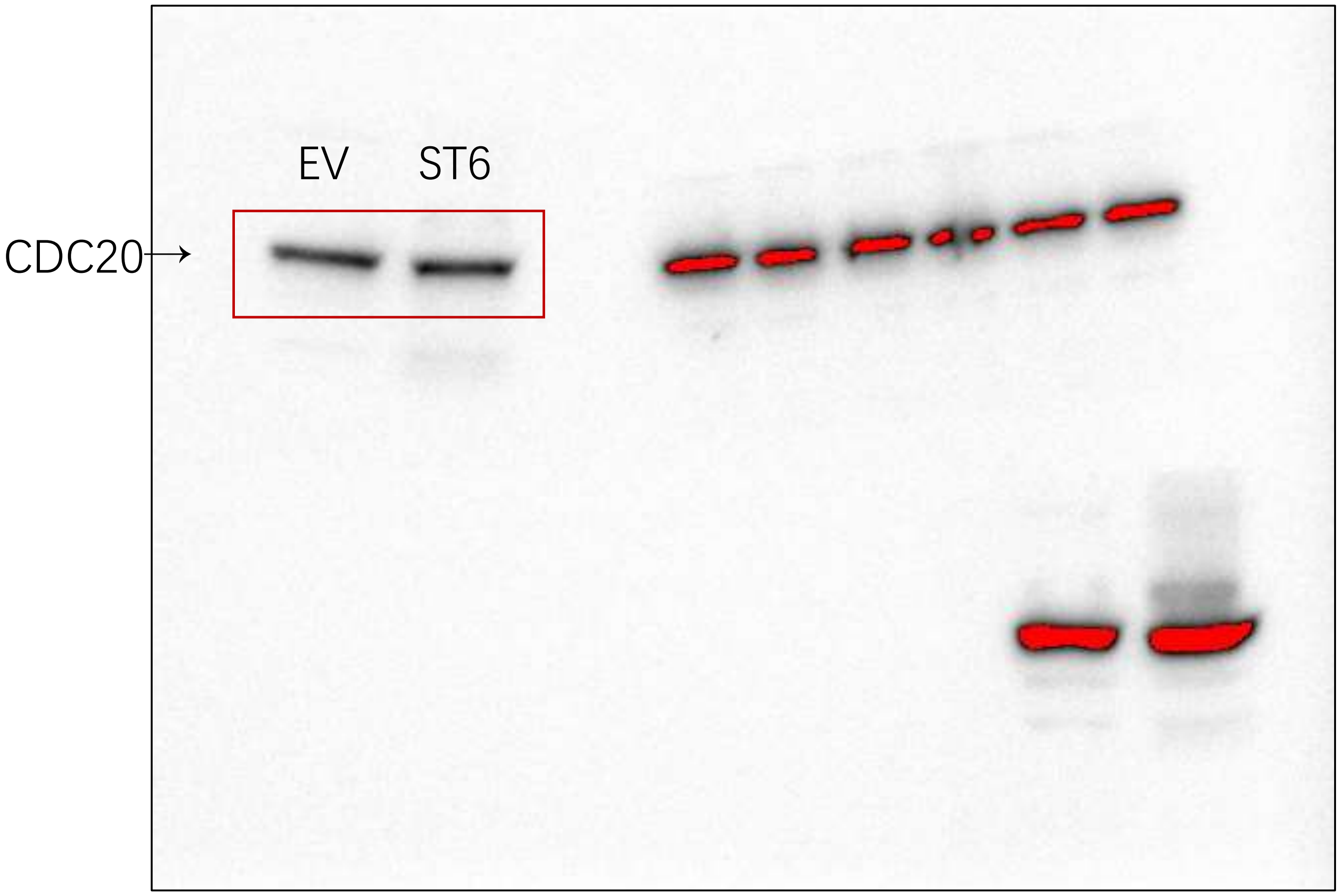

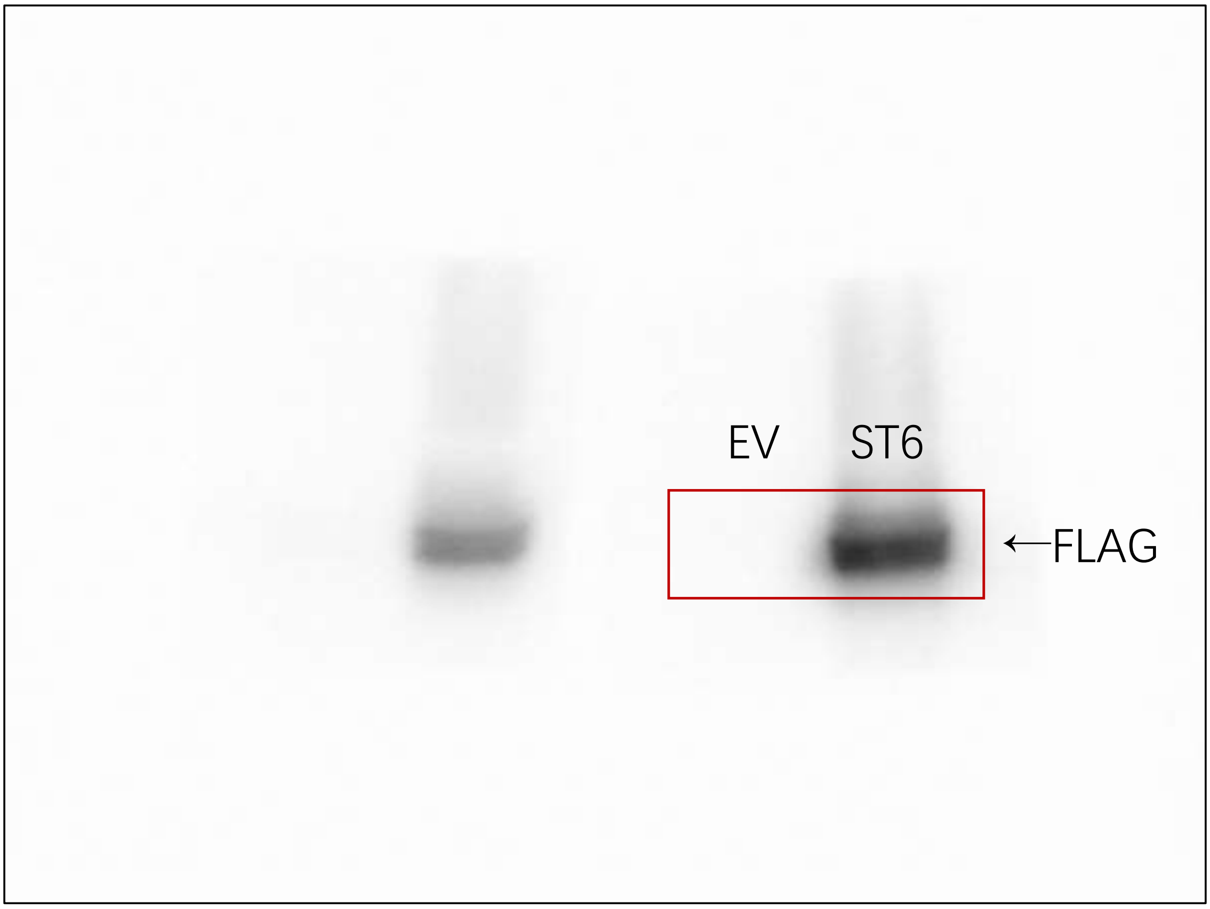

Original images of western blots of Actin in figure S7 HeLa

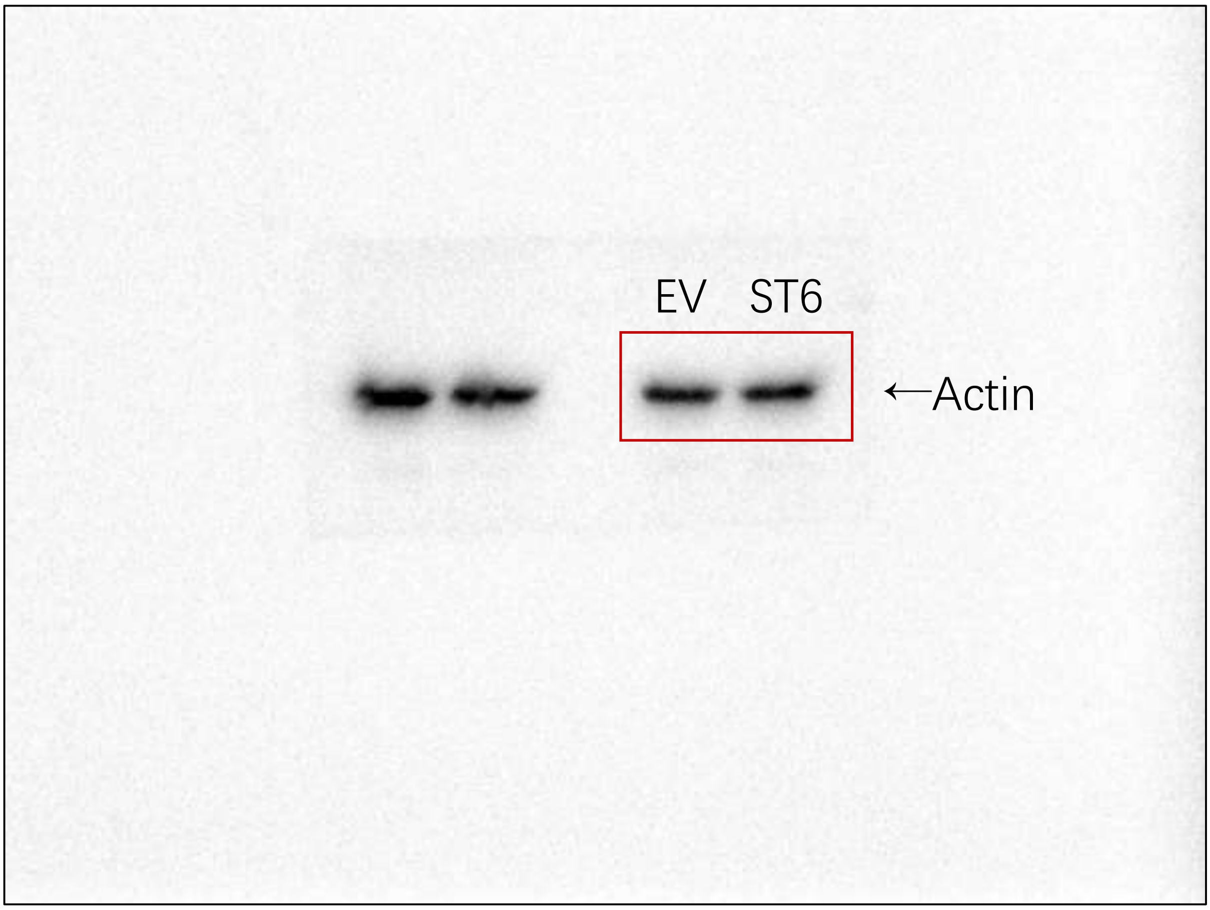

Original images of western blots of CDC20 & SIRT6 in figure S8 293T

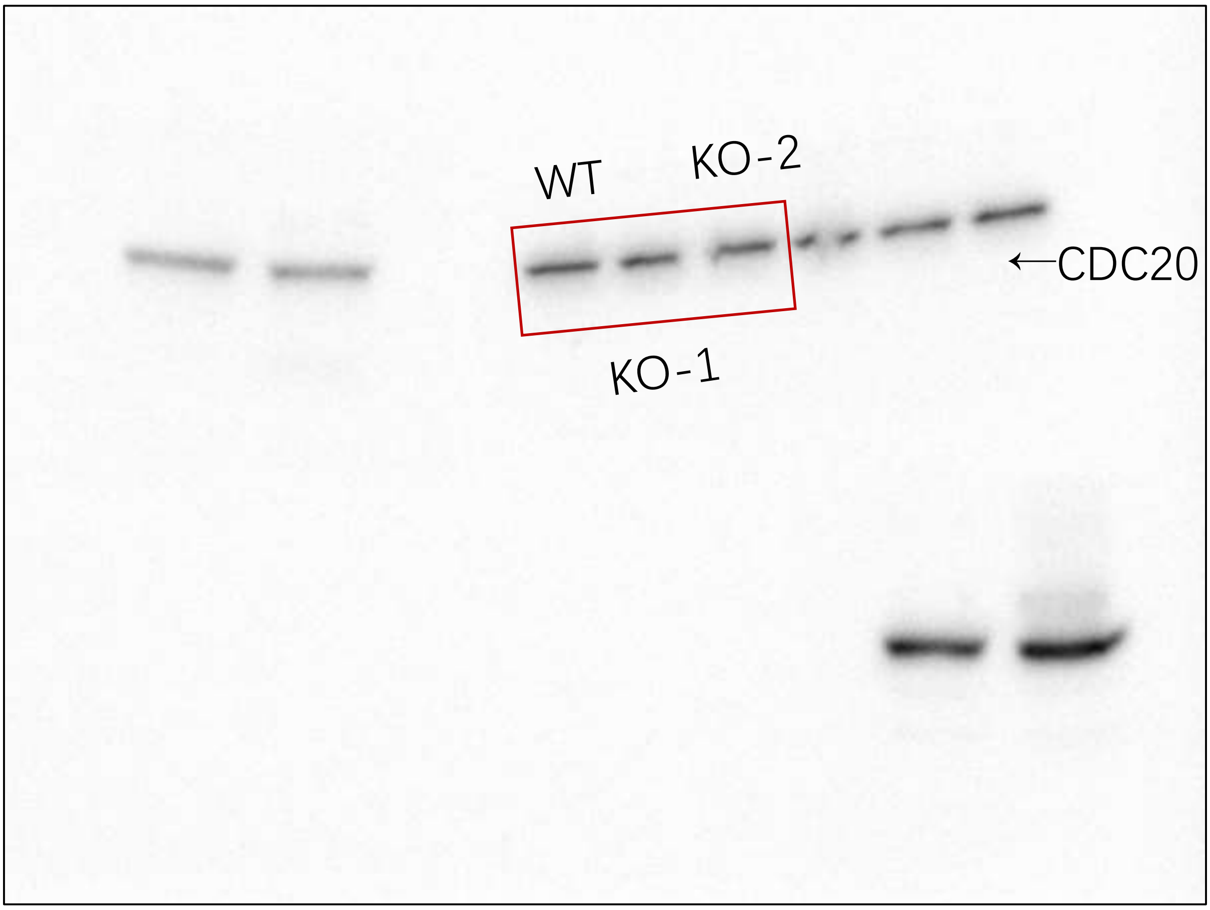

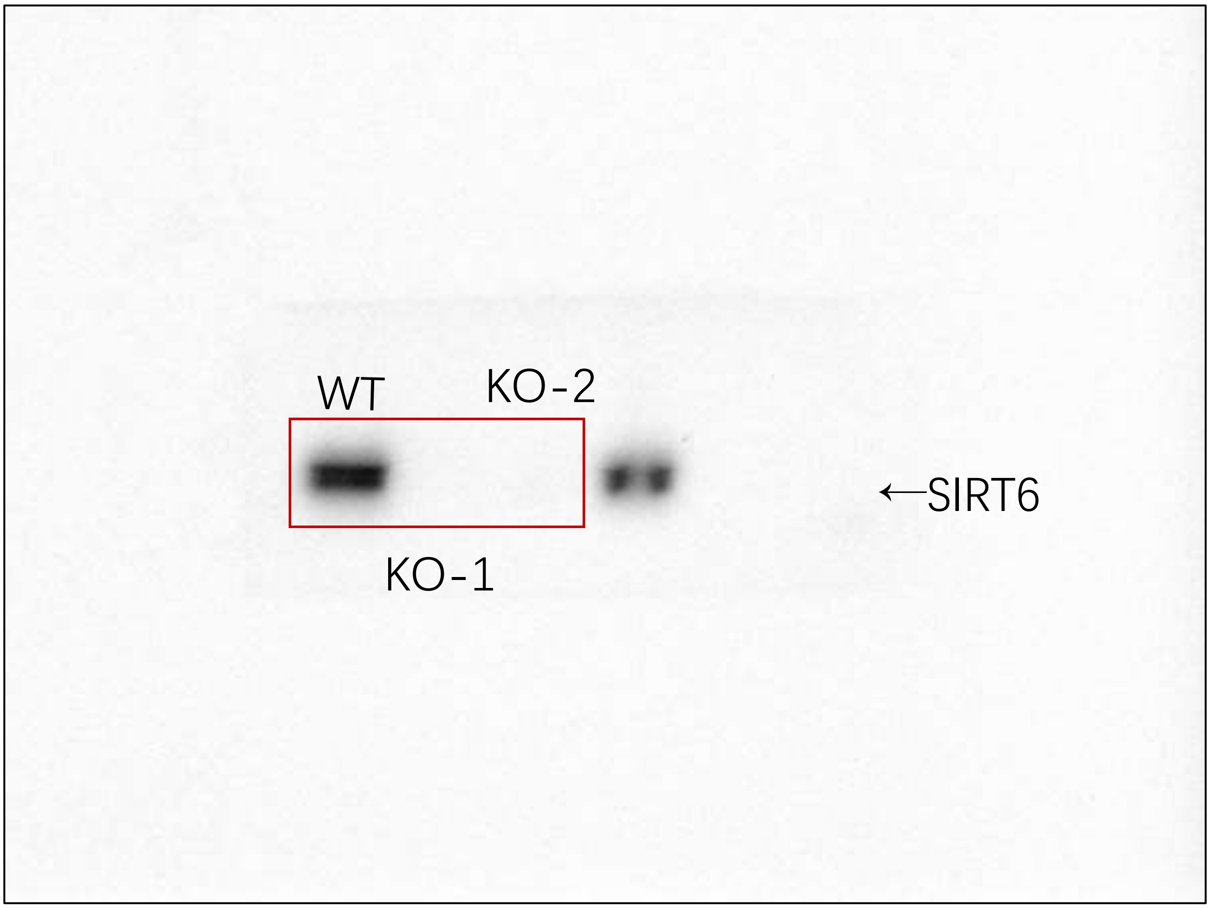

Original images of western blots of actin in figure S8 293T

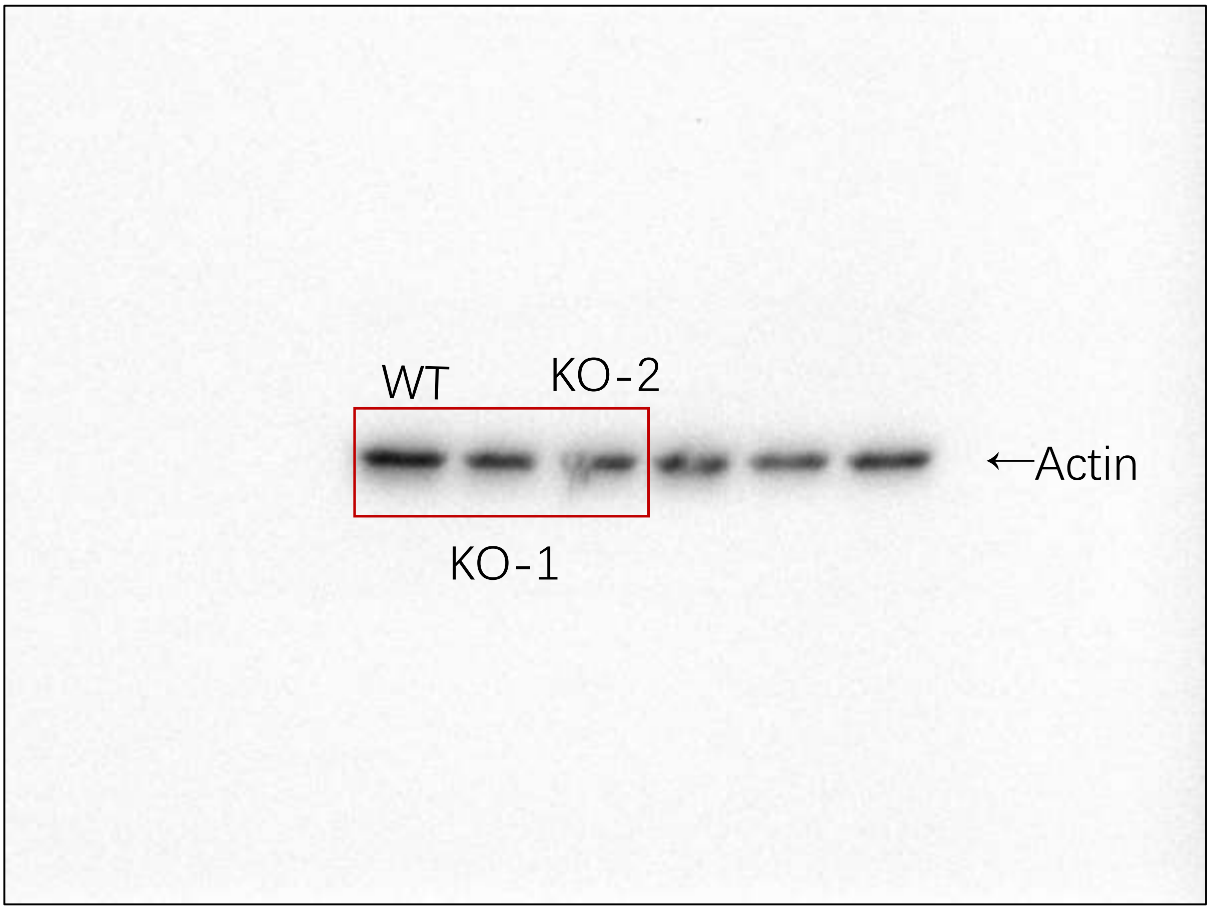

Original images of western blots of CDC20 & SIRT6 in figure S8 HeLa

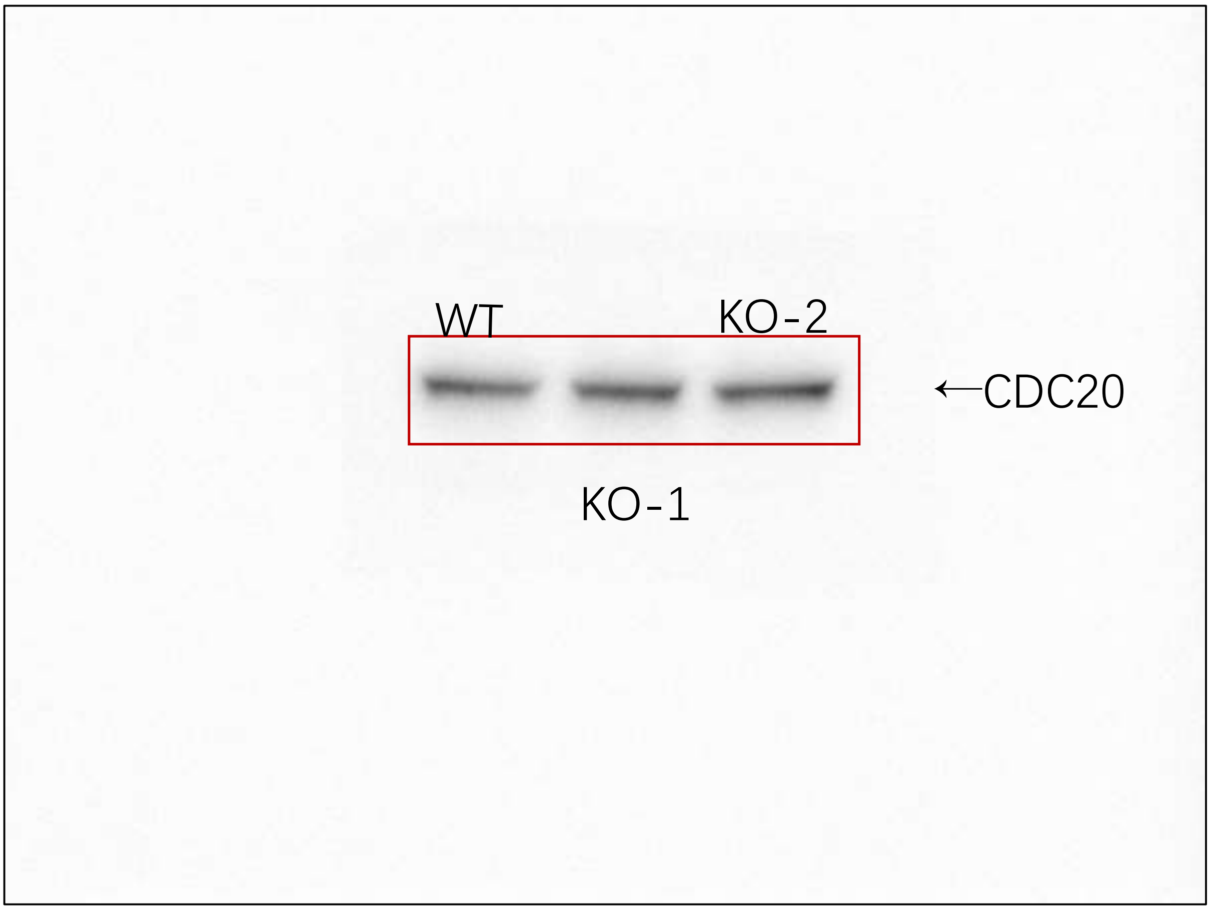

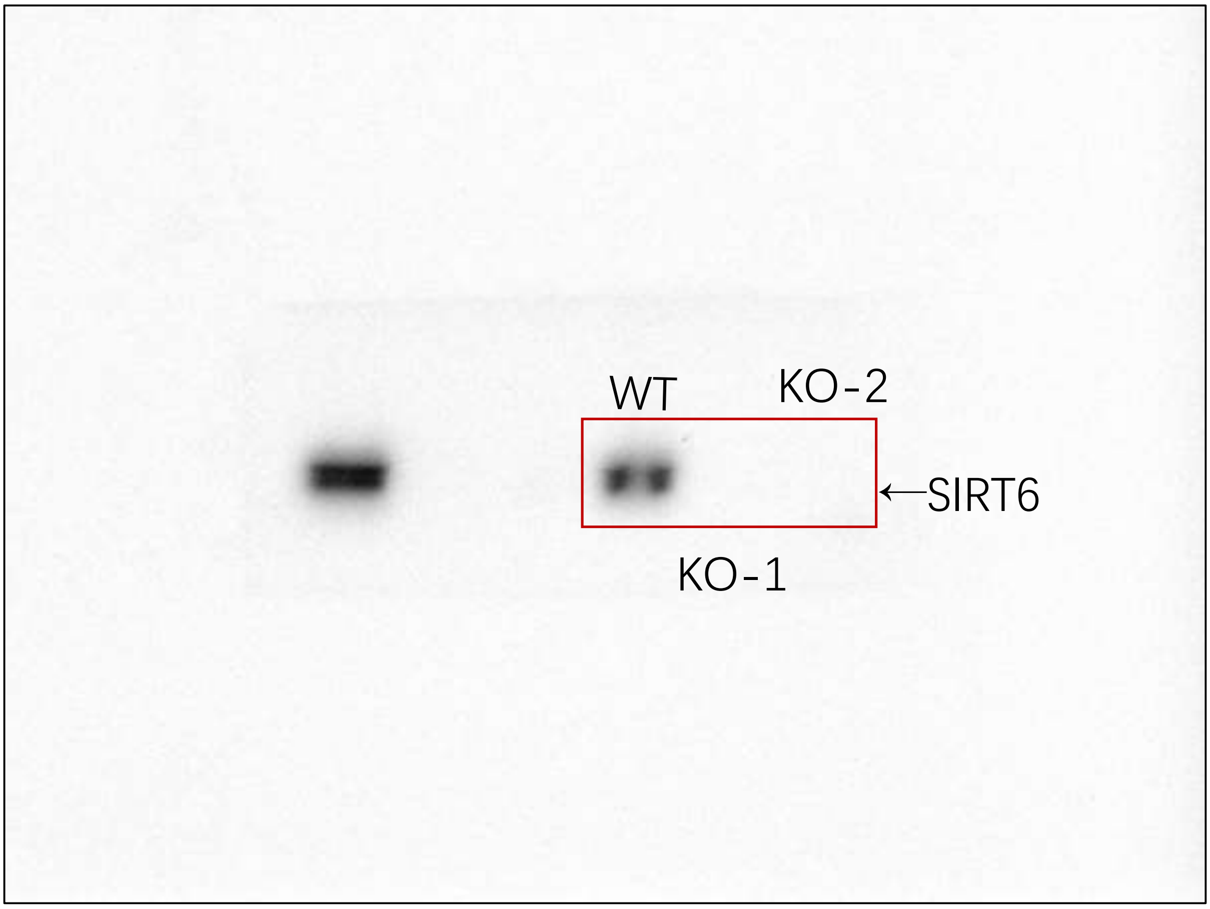

Original images of western blots of actin in figure S8 293T

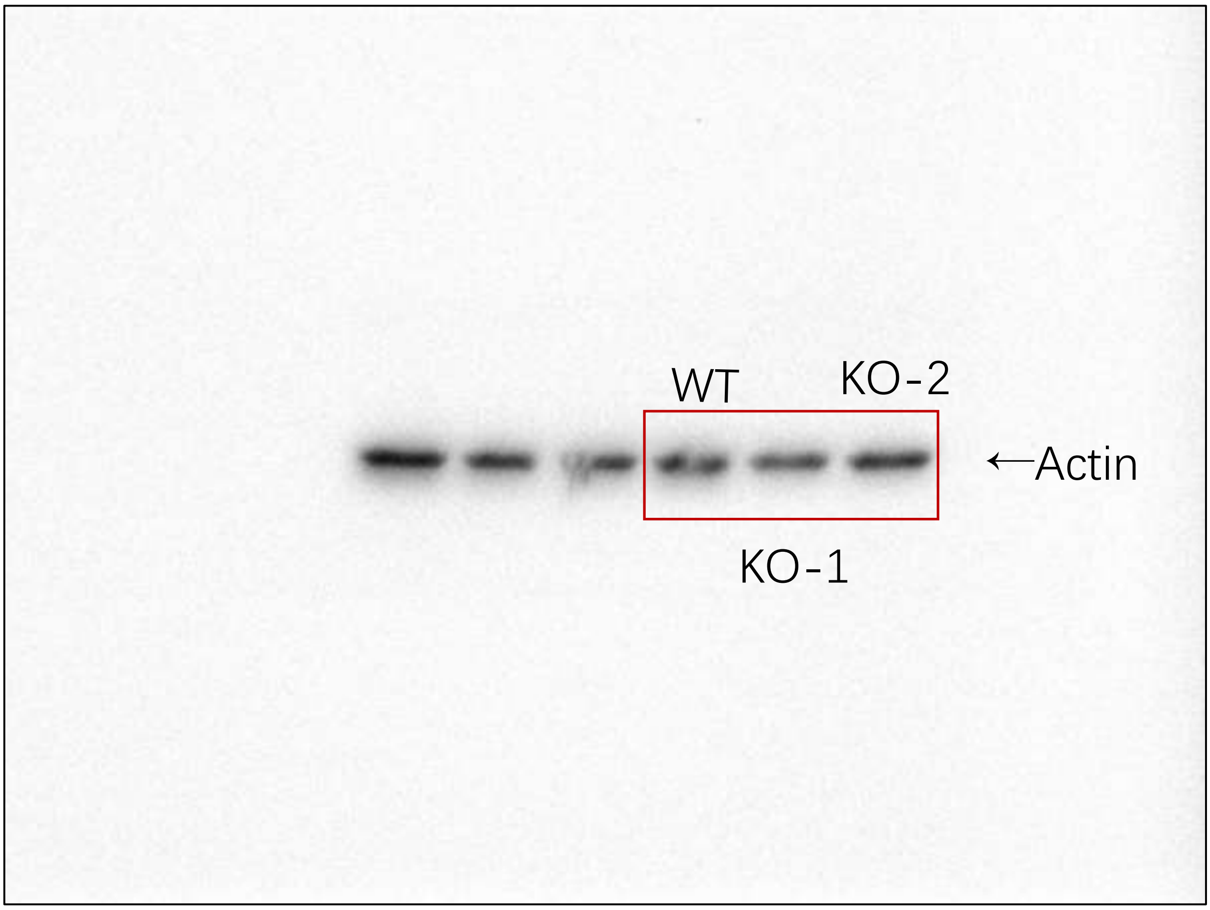

Original images of western blots of CDH1 & FLAG in figure S9

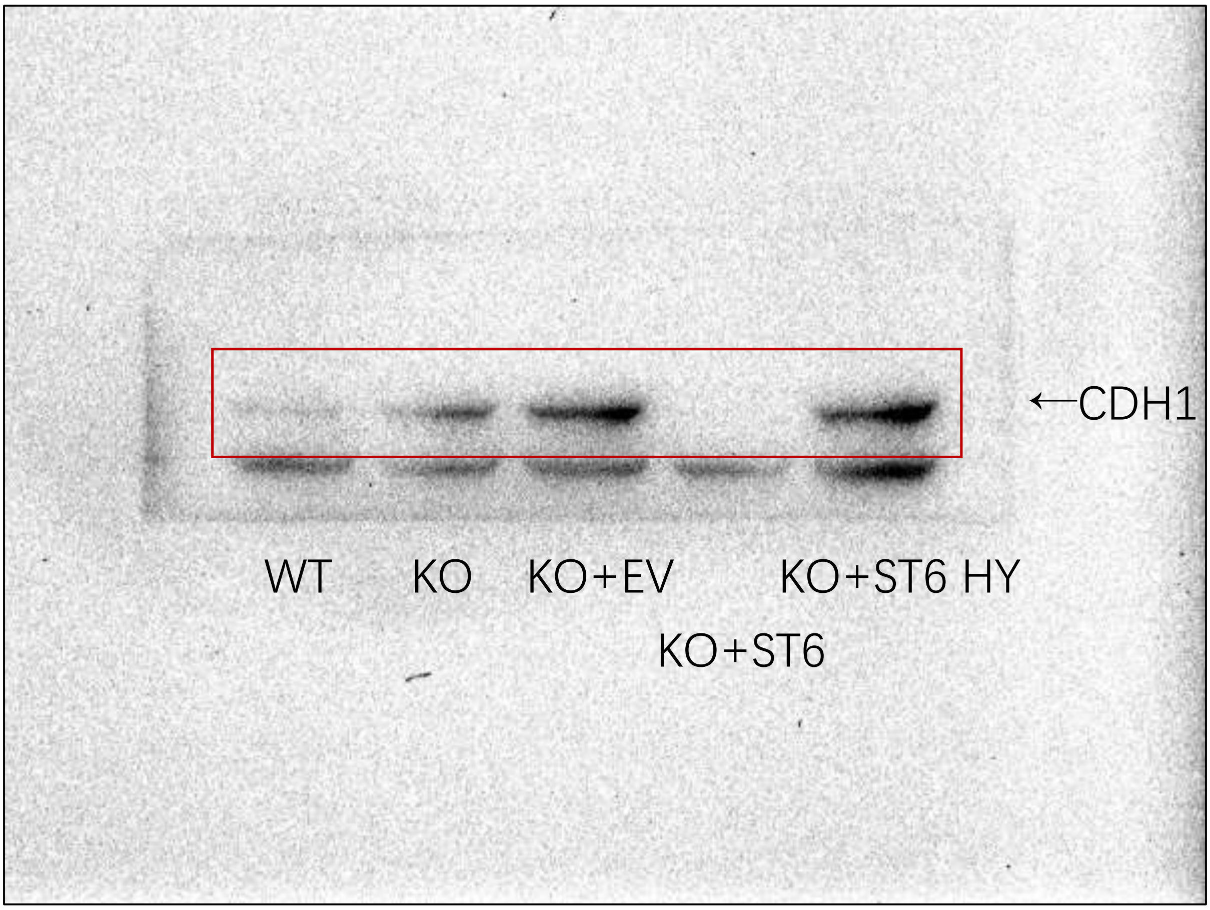

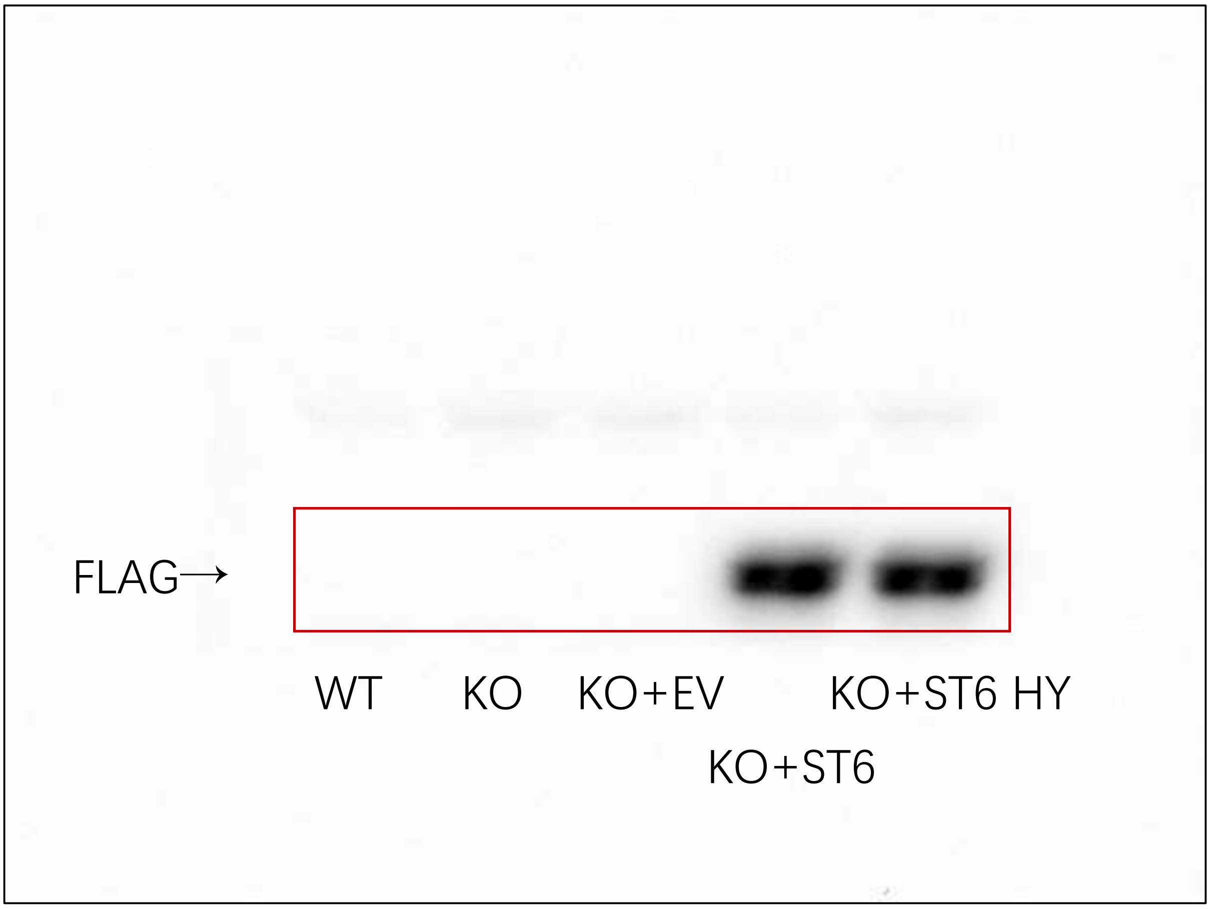

Original images of western blots of CDH1 & FLAG in figure S9

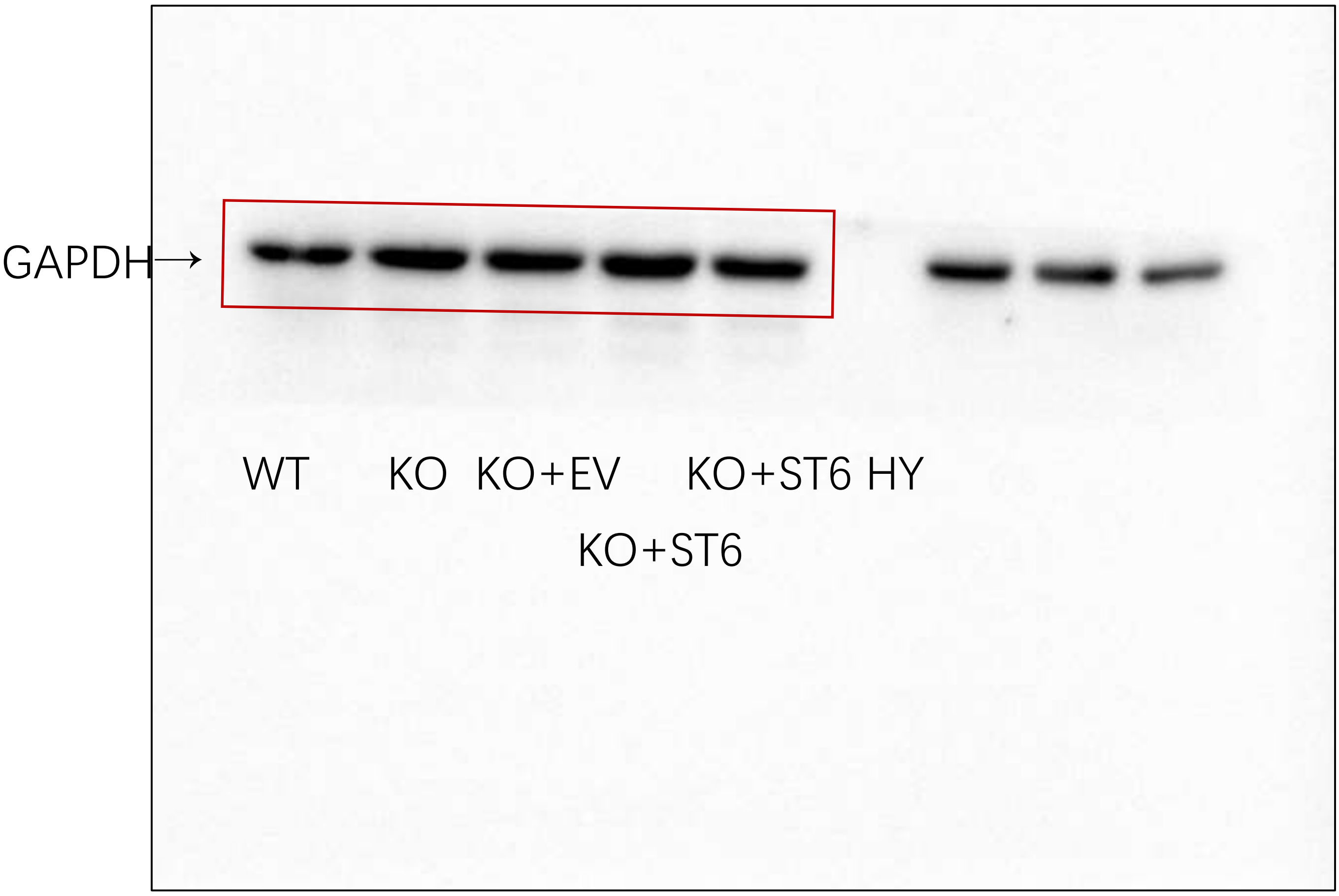

Original images of western blots of HA & FLAG in figure S12

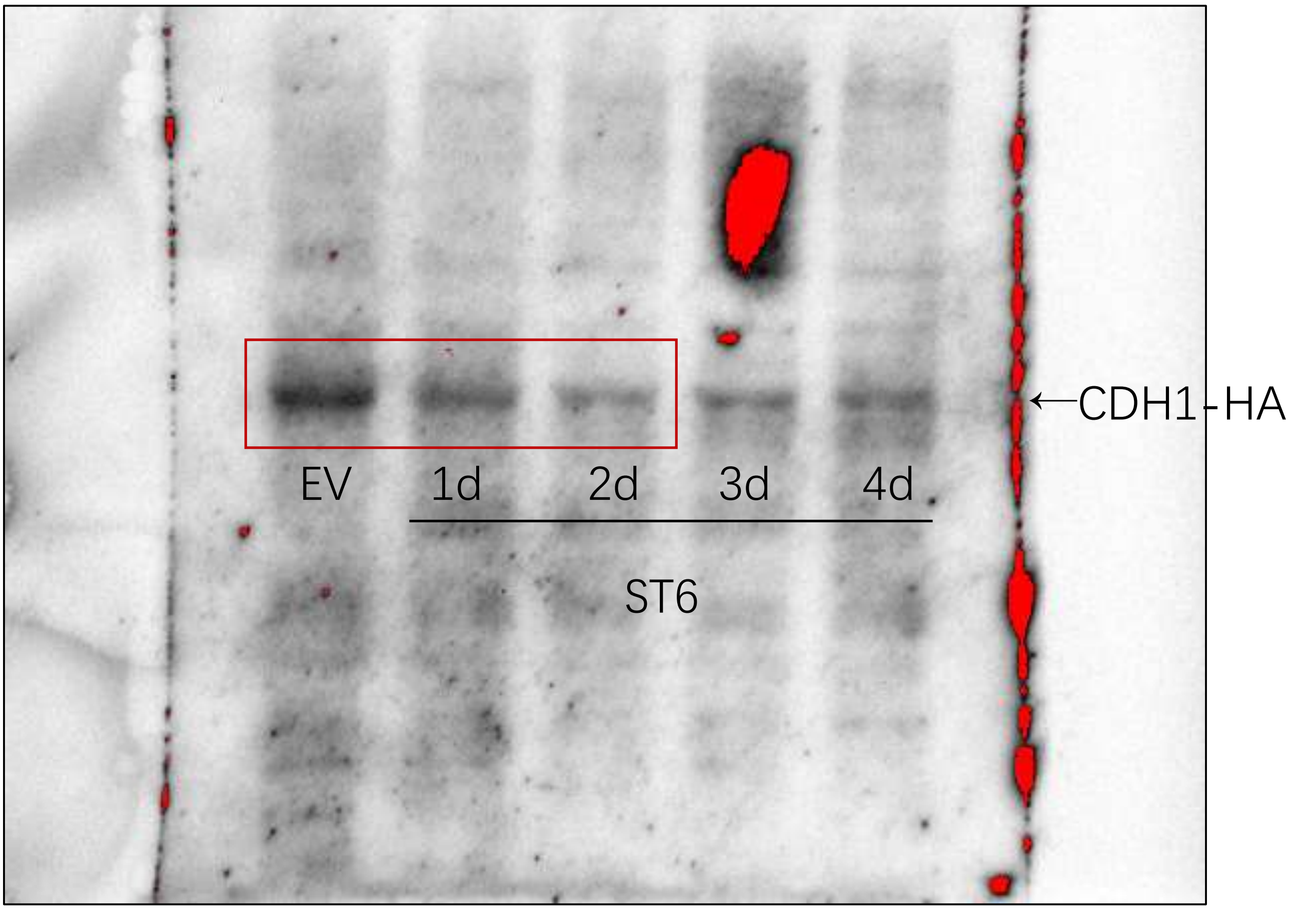

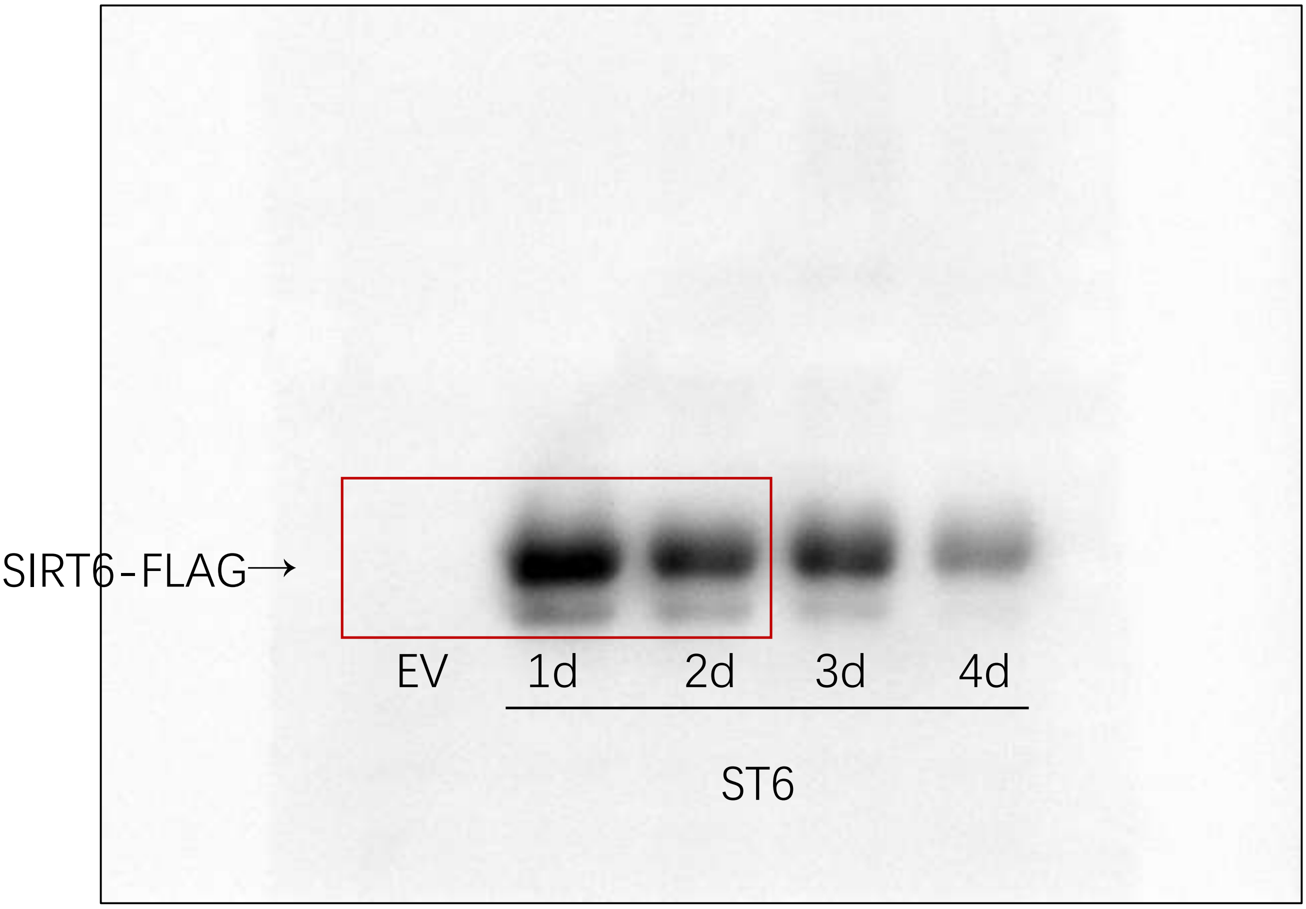

Original images of western blots of actin in figure S12

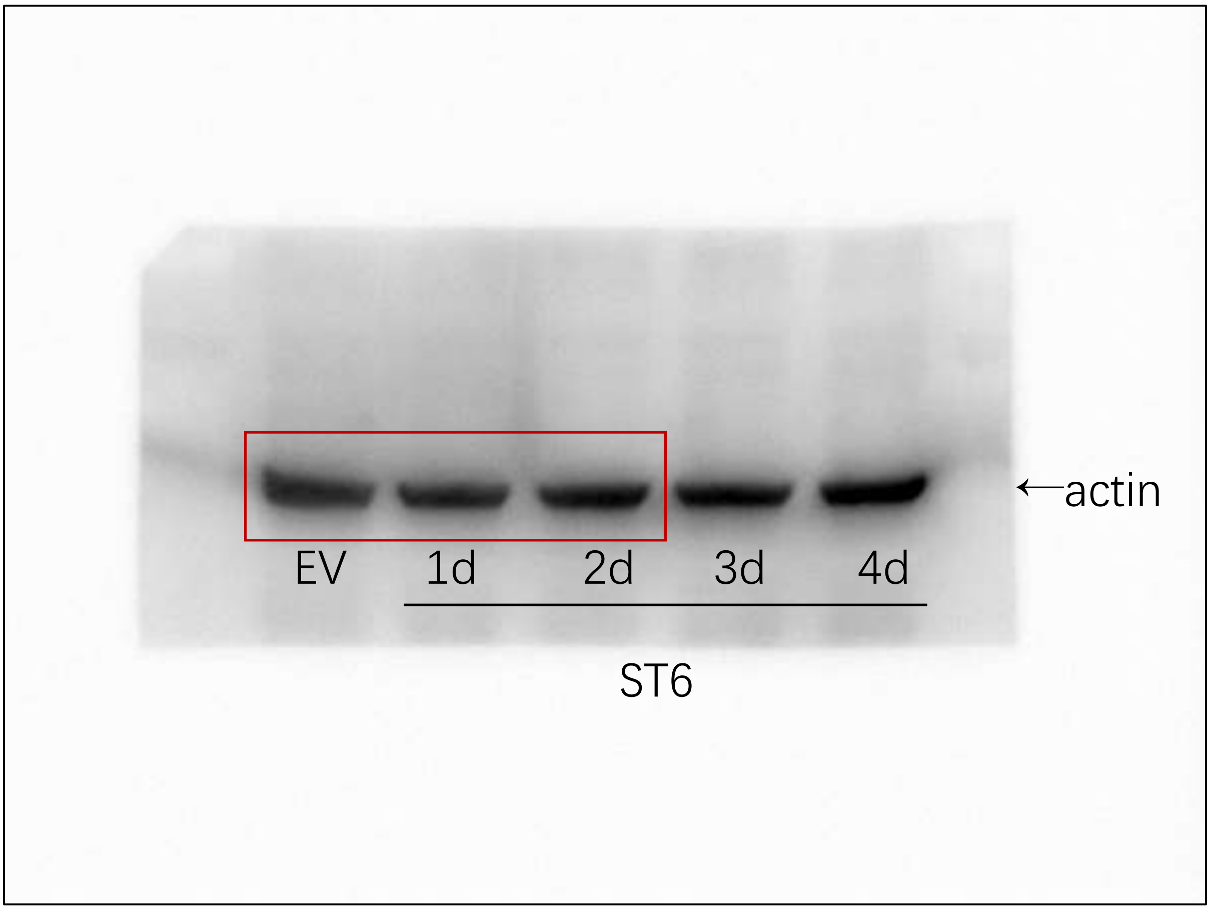

Supplement: Supplementary file 1 — Supplementary Information. [file 41598_2021_93684_MOESM1_ESM.pdf]
